# Supplementary material for: Picornavirus Evolution: Genomes Encoding Multiple 2ANPGP Sequences—Biomedical and Biotechnological Utility
Source: Viruses. 2024 Oct 9;16(10):1587. doi: 10.3390/v16101587 (PMC11512398; doi:10.3390/v16101587)
Supplement: Supplementary file 1 [file viruses-16-01587-s001.zip › viruses-3237076-supplementary.pdf]

Avisivirus Polyprotein Alignment

```
AsV-A1-HUN          MDPISEIAGAVTEVA--KDLAPAPVNQIIEGVSNLTTTPSANSTIQTSAPTVDTSIPHGT
AsV-A1-USA-IN1      MDPISEIAGAVTEVA--KDLAPAPINQIIEGVSNLTTTPSANSTIQTSAPTVDTSIPHGT
AsV-C1-45C          MEQVGAAIAAVSDAA--ASLAENPVQEIIEGVGNLATTVSTNALVQTSNPTVETGIPDST
AsV-C1-HUN          MEQVGAAIAAVSEAA--ASLAENPVQEIIEGVGNLATTVSTNALVQTSNPTVETGLPDST
AsV-C1-             MEQVGAVVTAVSQAA--SNLAENPVQEIIDGVGNLSTTISTNALVQTANPTVETGLPDST
AsV-B1-44C          MEVLAAVNGAVADVNNFATAATEIVTDAVTGVGSIQAAQSDNASVQSSHPVQQVGVADST
                    *: :.    **::.      *   : : : **..: :: * *: :*:: *. :..:...*

AsV-A1-HUN          SQILDSFFSCGTVQETSVLNYEKMILLDQAEWGTTPDVSYCLLKISVPGAFFSDATKPAH
AsV-A1-USA-IN1      SQILDSFFSCGTVQETSVLNYEKMILLDQAEWGTNPDVTHCLLKTSVPGAFFSDASRPAH
AsV-C1-45C          NVISDDYLSCACTVDTDTMNVEKTILFGSDNWSSNQAFGTCISRYDVPDVFLNSDSCPAY
AsV-C1-HUN          NIISDDYLSCACTVDSDTMNVEKTILFGTDDWSSNHTFGTCISRYDVPNAFLDSNSCPAY
AsV-C1-             DTITDDYLSCACTVDTDTMNVEKTILFGTDEWSANHRFGTCISRYDVPDVFFNSDSCPAY
AsV-B1-44C          SGSTDDFLSCSLKVDTAKVNPAKAVLIGTATWTSNDVMYEVIENWDLPNVFFHDSNFPAY
                    .   *.::**.   ::  :*  * :*:.   * :.  .   : . .:*..*: . **:

AsV-A1-HUN          GISKYFRLLRCGYRFTVVLSVPPGACGALAMVFVPPGFTDKITQ-NQPVTA-WDPEAILT
AsV-A1-USA-IN1      GISKYFRLLRCGYRFTVVLSVPPGACGAVAMVFVPPGFNNKMTV-GQTVTK-WDPEAVLT
AsV-C1-45C          GQSSYFRFLRCGFRFQITTNPPPGAGGSLILAYMPPGFQFRVQQKGATITG-FDPEAVLT
AsV-C1-HUN          GQSSYFRYLRCGFRFQITTNPPPGAGGSLILAYVPPGFQFRVQPKGQTITG-FDSEAVLT
AsV-C1-             GQSSYFRFLRCGFRFQVTTNPPPGAGGSLILAYVPPGFQFRVQQKGAQITG-FDPEAVLT
AsV-B1-44C          GQSRFFRFLRCGYRFHLTFNPPPGSQGCLVLSFVPPGYAHCIPAKGTATNWKFDTDALLT
                    * * :** ****:** :. . ***: *.: : ::***:   :   .   .  :*.:*:**

AsV-A1-HUN          LPHVVVDSRTSNTGTLTVPYVNYQSYCNLDQNGNQ-AFVAVLVLGKYNSANGTSSSCDIA
AsV-A1-USA-IN1      LPHVVVDSRTSNAGTLTVPYVNYQSYCNLEQNGNQ-AFVAVLVLGKYNSGTGTSSTCDIA
AsV-C1-45C          LPHVIVDIRSSTHSALTIPYVNHKNYFNYSYSGDHRGTVIVFVLGQYTVGSGTSSNVGVS
AsV-C1-HUN          LPHVIVDIRSSTHSALTIPYVNHKNYFNYAYQQDFRGTVVVFVLGQYTVGSGTSSTVGVS
AsV-C1-             LPHVIIDIRSSTHSSLTIPYVNHKNYFNYSYGNDHRGTVVVFVLGQYTVGTGTSSNVGVS
AsV-B1-44C          LPHVRCDARSTTMSSLVVPYINFNSYVDYTGSGTATAHIIVWVLGRYRCGTGTSTNIDYS
                    ****  * *::. .:*.:**:*.:.* :        . : * ***:*  ..***:. . :

AsV-A1-HUN          LYGELLDTDFQCPRPVS-QG------KRRKAAKPEHNPTT-AMVSIASGPGSANLANSTL
AsV-A1-USA-IN1      LYGELLDTDFQCPRPVS-QG------RRRKASRPEQNPTT-AMVSIASGPGSANLANSTI
AsV-C1-45C          VFGEMLEADFQCPRPYRVQGQNRRKIRRRRAPPPPANPPVGRHVNIGPAPGAVIAANSVL
AsV-C1-HUN          VFGEMLEADFQCPRPYQSQGQNRRRIRRRKAPPPPSNPPVGRHVNIGPAPGAVVAANSVL
AsV-C1-             VFGEMLDADFQCPRPYRVQGQNRRKIRRRKAPPPPDNPPIGRHVNIGPAPGAVVAGNSVL
AsV-B1-44C          VYGEMLDMDLQCPRPWD------------------QGPTR---MVVDPAPGAVMAGNSKI
                    ::**:*: *:*****                     .*.    : : ..**:.  .** :

AsV-A1-HUN          APKLADSLAIANEGTAVDYSTAGCDQSVTDLIELARSWQIAAYGKLDSAQKNS--VVLNI
AsV-A1-USA-IN1      APRLADSLAIANEGTAVDYSTAGCDQSVNDIIELARSWQLAAYGKLNSADKDT--VILNM
AsV-C1-45C          NITTADSLAIGNEGTAVDCTTAGAASAIPDVRELASDWQILHQQSMSWAALNAGDRVWSG
AsV-C1-HUN          NVTTADSLAIGNEGTAVDCTTAGASSAIPDVKELASDWQILHQESRSWAALTAGARVWSG
AsV-C1-             NVTTADSLAIGNEGTAVDCTTAGASSAVPDVKELASDWQILHQESRSWAALTAGQRVWSG
AsV-B1-44C          MN-DCQTVALAGEGTLVDNTTAGAKVAKTSLLSPARHWQIMASFDWTTTGALG-AQIFKA
                        .:::*:..*** ** :***.  :  .: . *  **:    .   :       : .

AsV-A1-HUN          NFDPYSYGNLGLLFDKFQYWRGSLEVQFVMYSNSLASGRYQLCWLPADWS---ADYSLAQ
AsV-A1-USA-IN1      NFVPYSYGNLGLLFDKFQYWRGSLEVQFVMYSNSLASGRYQLCWFPADWSDSSRAYTLAQ
AsV-C1-45C          NFAPYEVGNIGALMDKFMYWRGSFEVQLVVYGSSLTSGRIQLSFYPGMSNN--NGRTLQQ
AsV-C1-HUN          NFAPYEVGNIGALMDKFMYWRGSFEVQLVVYGSSLTSGRIQLSFYPGMPNN--NSRTLQD
AsV-C1-             NFAPYEIGNIGALMDKFMYWRGSFEVQLVVYGSSLTSGRVQLSFYPGLPND--NSRTLQQ
AsV-B1-44C          NFEPFSYGNIGFLFDKFLFWRGSLEIAVLAFGSSLTSGRFQVSWYPDLSAR---DLTVAQ
                    ** *:. **:* *:*** :****:*: .: :..**:*** *:.: *          :: :

AsV-A1-HUN          LRNSIYSTGDVSSAPCTLVLPFTSQNWRRRCDGRYGSIIVRMVNRLAVNGSSTTHFSYAL
AsV-A1-USA-IN1      LRNSIYATGDVSSAPCTLVLPFTNQNWRRRCDSNYGSITVRMVNRLAVNGSSTTHFSYAL
AsV-C1-45C          MRNAFYSTGDISAAPTRLTIPFTNDSWRRRCDQQYGSFYIHIVNRVCVNASASPTMSFVL
AsV-C1-HUN          MRNAFYSTGDISATPTRLTIPFTNDSWRRRCDQQYGSFYIHIVNRVCVNASASPTMSFVL
AsV-C1-             MRNAFYSTGDISATPTRLTIPFTNDSWRRRCDQQYGSFYIHIVNRVCVNASASPTMAFVL
AsV-B1-44C          VRNSIFATGDISSVATRLTIPFTNPNWRRRCDSAYGSIYVHSINRQTVNSTANPAIQMII
                    :**::::***:*:..  *.:***. .******  ***: :: :**  **.::.. :   :
```

```
AsV-A1-HUN          FVRAGQDLQFFAPRYGDYSIL--------QGPIEGETYNQTSTLSTNFEISDVVIHGSKH
AsV-A1-USA-IN1      FVRAGQDLQFFAPRYGDYSIQ--------QGPIEGETYNQASTLSTNFEISDVVIHGSKH
AsV-C1-45C          FVRLGPDFQFFCPRYGDYHIQGPTVEKNPGEPEEGETYSGQPNVFLNFDCVEVPIQGASH
AsV-C1-HUN          FVRLGPDFQLFCPRYGDYHIEGPIVEKTPEESEEGETYSGQPHVFLNFDCVEVPIHGASH
AsV-C1-             FVRLGPDFQFFCPRYGDYHIEGPVVPANSEVTDEGETYVGQPHVFLNFDCVEVPIHGASH
AsV-B1-44C          LVRLGPDVDFFCPRYGDYHIQG-----------DDTELIDEVQSFLNFTIKEVPIQTASH
                    :** * *.::*.****** *             :.           **   :* *: :.*

AsV-A1-HUN          TQIDNFFGRSWVHGFHTSSSADTAMKLPLRTPRHSHGSAMLAFAYWCGEVVITVHNRSEN
AsV-A1-USA-IN1      TQIDNYFGRAWVEGFHTSTAASTAMKLPLQTPRHSHGSAMLGFAYWCGEVVITVHNRSQN
AsV-C1-45C          TLVRNLFGRLWLQ-EHTVSPSSGPHVVKLEVPNESHAAILQCFAYFSGEVIISIRNGGDT
AsV-C1-HUN          TLVRNLFGRLWLQ-EHTVSPSSGTHVVNLEVPNESHAAILQCFAYFSGEIILSIRNGGDT
AsV-C1-             TLARNLFGRLWLQ-EHTVSPATGTHVVNLDVPNESHASILQCFAYYSGELILSVRNGGDT
AsV-B1-44C          TLIPNFFGRAFYYGKYTSPAETSASVIPLKVPQYGHGSLMTMFAYFTGEVVLTVHNRGTG
                    *   * *** :    :* ..   .  : * .*. .*.: :  ***: **::::::* .

AsV-A1-HUN          MLICAHSYDLEEQHSQVNEQSIFSLGAILVPPREVKTFRAPWYSQTPLRRP-LDNDDEPS
AsV-A1-USA-IN1      MLICAHSYDLEEQHSQVNEQSIFSLGAILVPPREVKTFRAPWYSQTPLRRP-LDDPNEPS
AsV-C1-45C          TVIAAHTYIPEEQHNPVDEFSIMSLGAVIIPPLEIKIIRVPFYSPSPLRMIRRHRDFEPT
AsV-C1-HUN          TVIAAHTYVPEEQHDPTNEFSIMSLGAVVIPPLEIKTIRVPFYSPSPLRMIRRHNTFEPT
AsV-C1-             TVIAAHTYVPEEQHDPTNEFSIMSLGAVVIPPLEIKTIRVPFYSPSPLRIVRRGNGFEPT
AsV-B1-44C          LLVLAHTYIIEEQHNPSDESTIFSLGAVLVPPGEVKTFACPYYAHTPLRPLRLEDT--PA
                     :: **:*  ****.  :* :*:****:::** *:* :  *:*: :***         *:

AsV-A1-HUN          MGFLYVTSEGSSNFTVYLALHKPKFFFPLPCPRFTSNSAKVAPKGTGMSIAERKIQLTSV
AsV-A1-USA-IN1      MGFLYVSSEGTSDFTVYLALHKPKFFFPLPCPMFTSNQSREAPRQP-KSIAERKIELSSV
AsV-C1-45C          FGYLYLCSPSTSNVTVYMGLANPNLFFKLPCPQYTVNSR------------------AAI
AsV-C1-HUN          FGYLYLCSPSTTNVTVYMGLANPNLFFKLPCPRYTTNTR------------------SRS
AsV-C1-             FGYLYLCSPSTTNVTIYMGLANPNLFFKLPCPRYTINTR------------------AIS
AsV-B1-44C          FGYLYASGEGAIPFTVYISLRDPKFFLDMPQPAFTSNTRAAGARVG-----RQGYVRSAI
                    :*:**  . .:  .*:*:.* .*::*: :* * :* *                    :

AsV-A1-HUN          ARRTLEWARREVGAYDEVDHRDILMGGDIEENPGPQRYQPLHKPEPSSNLLRAVSRRAIM
AsV-A1-USA-IN1      ARRTLEWARREVGAIDETDHKDILLGGDIEENPGP-------------------------
AsV-C1-45C          TSAGPRFFR------EDDDHYNILLGGDIEENPGP-------------------------
AsV-C1-HUN          ARSAPRFFR------EDDDHYNILLGGDIEENPGP-------------------------
AsV-C1-             TRSAPRFFR------EDDDHYNILLGGDIEENPGP-------------------------
AsV-B1-44C          RLPLPQFEK------ERSAHEDVLLGGDVESNPGPVELQNGQQACVGFAPGPISGKEYKM
                         .: :      :   * ::*:***:*.****

AsV-A1-HUN          SMVEENIQLT--PITRKAKVKVLDWVRREMGVFDETDHRDILLGGDIEENPGPQSIYFLG
AsV-A1-USA-IN1      -----------------------------------------------------QSVYLLG
AsV-C1-45C          -------------------------------------------------------VVVCG
AsV-C1-HUN          -------------------------------------------------------VVVCG
AsV-C1-             -------------------------------------------------------VVVCG
AsV-B1-44C          QDYQHSAVLAGEAALVTHQGRDALYWFRSESVQYLEPQIDICVCGDVERNPGPK-IVVVG
                                                                **:*.****  : . *


AsV-A1-HUN          LSGCGKSRLVNAIAGHPLCDSRLSPTPIHTETHSHQLMG----YEVFEQVGIPPAGGKYV
AsV-A1-USA-IN1      LSGCGKSRLINAIAGHPLCDSRLSPNPIHTETHSHQLMG----YEIFEQVGMPPAGGKYV
AsV-C1-45C          PSKGGKTRLLCAMAGHPIVPS--FPGPHVANMLGEEFT------EIPEDMKLPLNKKILV
AsV-C1-HUN          PSKGGKTRLLCAMAGHPIVPN--FPGPHVANMLGEEFT------EIPEDMKLPLNKKILV
AsV-C1-             PSKGGKTRLLCAMAGHPIVPS--FPGPHVANMLGEEFT------EIPEDMKLPLNKKILV
AsV-B1-44C          RSGSGKSRLCNMILGHDYFPSRLSSTPVTTKMRTATLPCGTQIVDTPERFSIQEEIKGFI
                     *  **:**   : **    .   . *  ::     :       :  * . :       :

AsV-A1-HUN          YLQEATRFDKEHVDFIREMDQQHPGWRRHAVLYVNRLGDTKLSQYLRGVPELAGFKEVTD
AsV-A1-USA-IN1      YLQEATRFDKEHVDYIREMDKLHPGWRRHAVLYVNRLGDTKLSQYLRGVPELAGFKEATD
AsV-C1-45C          VLGEDN---DANANYIQWLSEEYPSWNHRAVVFC-WPGCQLQGNNFR----------ICR
AsV-C1-HUN          VLGEDN---DANANYIQWLSEEYPSWNHRAVVFC-WPGCQLTGNNFR----------ICR
AsV-C1-             VLGEDN---DANANYIQWLSEEYPSWNHRAVVFC-WPGCQLQGNNFR----------ICR
AsV-B1-44C          WVLEEGRWTQENKDFLAYMDTSYPGWRRHCVIYHTRHEDPGSNFPQFLKDAGLSSFQWSK
                     : *     . : :::  :.  :*.*.::.*::         .
```

```
AsV-A1-HUN          NPLSVIPLLTTINSYEGTGVQLVCKNRGVYKHFGVREGDKVYHINTENLVKTCLDGQVAV
AsV-A1-USA-IN1      NPLNVIPLLVSIDTYVGTGVQLVCKNRGVYKHFGVREGDKVYHINTENLVKTALDGEVAV
AsV-C1-45C          NPIAVSALLAQSLPYNTRGTQLVYQDRGLYRHYGVLFDNKVFHLDSQDILKSGLKGSAVV
AsV-C1-HUN          NPIAVSALLAQSLPYNTRGTQLVYQDRGLYRHYGVLLDNKVFHLDSQDILKSGLKGSAVV
AsV-C1-             NPIAVSALLAQSLPYNTRGTQLVYQDRGLYRHYGVLFDNKVFHLDSQDILKSGLKGSAVV
AsV-B1-44C          NPLDLTSRFLMIPPYRNCLVQLVFKDRGLYKHYGARIGARIFEVNSDNLLSALTKKEVPI
                    **: : . :    .*    .*** ::**:*:*:*.  . :::.:::::::.:  . .. :

AsV-A1-HUN          MVEDYSAGWIPCSHEEKVGAVSFVQTGTLDGVTFSCDFNCETWAKIFVPSEGETQGQRLK
AsV-A1-USA-IN1      MVEDYSDGWIPCSPEEKIGAVSFVQTGTLDGVTFSCDFNCETWAKIFVPSEGETQGQRLK
AsV-C1-45C          SVDDP-LEWVPCSATDLAGSLDLANSGTIE-IDFNINSNCETWAKGVIGDLSPTQSDRLK
AsV-C1-HUN          SVDDP-LEWVPCSATDLAGSLDLANSGTIE-IDFNINSNCETWAKGVIGDLSPTQSDRLK
AsV-C1-             SVDDP-LEWVPCSATDLAGSLDLANSGTIE-IDFNINSNCETWAKGVIGDLSPTQSDRLK
AsV-B1-44C          VSTPDDGSWQVAEDLFTPEAQRLAKNLELEKVKYGFDANCETWAKDVLGVATPCQSHVVR
                            *  ..      :  :.:.  :: : :. : ******* .:      *.. ::

AsV-A1-HUN          KVMAIAAGAAFVYGLPRGEGFDFMQCVTKVMMTLFSKQVKTIVVKMVIKFFCRLCCYLVL
AsV-A1-USA-IN1      KVMAIAAGAAFVYGLPRGEGFDFMQCVTKVMMTLFSKQVKTIVVKMVIKFFCRLCCYLVL
AsV-C1-45C          KVLVVAAAAGFLYCLPHDQ-SGFMDGVTKCLINLFSKQVKSAMIRMAIKFVCRLVCYLIL
AsV-C1-HUN          KVLVVAAAAGFLYCLPHDQ-SGFMDGVTKCLINLFSKQVKSAMIRMAIKFVCRLVCYLIL
AsV-C1-             KVLVVAAAAGFLYCLPHDQ-SGFMDGVTKCLINLFSKQVKSAMIRMAIKFVCRLVCYLIL
AsV-B1-44C          KACCIAVLASLGVLTLSSQDVNVMGSITSVIVSLFSKQIKTAVVRLAIKFVLRLVCYLVL
                    *.  :*. *.:      .:  ..*  :*. ::.*****:*: ::::.***. ** ***:*

AsV-A1-HUN          YCHSPNLVNTAMLTILLTLDVFDTEIDEISGKVAKALVSGDFKAAGRAMMEAADRKCEDF
AsV-A1-USA-IN1      YCHSPNLVNTAMLTILLTLDVFDTEIDEISGKVAKALVSGDFKAAGRAMMEAADRKCEDF
AsV-C1-45C          YCHSPNILNTGVLTTLLLMDVFELEVDEGLDKLAHALIEGDFKGLGKFLKKRTGRDCDDF
AsV-C1-HUN          YCHSPNILNTGVLTTLLLMDVFELEVDEGLDKLAHALIEGDFKGLGKFLKKRTGRDCDDF
AsV-C1-             YCHSPNILNTGVLTTLLLMDVFELEVDEGLDKLAHALIEGDFKGLGKFLKKRTGRDCDDF
AsV-B1-44C          YCHSPNLINTAMLSALLVMDVFDNELDEAISKFAQLCIKGDFKGLGRWVKGKVGSDCQDF
                    ******::**.:*: ** :***: *:**  .*.*:  :.****. *: :   .. .*:**

AsV-A1-HUN          K--CEAKELFKSEGPDATGKSFNTWTLVAKNLEWWVDKLKQFINWIRTKLFPSDAKDKIE
AsV-A1-USA-IN1      K--CEGKRTLQSEGPDATGKSFNTWTLVAKNLEWWVDKLKQFINWIRTKLFPSDARDKIE
AsV-C1-45C          EPGDDHRPIFRAEGPDDLPKTFNSWSLMAKNVEWWITKFADFCKWLKEKVFPENHEDQIE
AsV-C1-HUN          EPGDDHRPIFRAEGPDDLPKTFNSWSLMAKNVEWWITKFADFCKWLKEKVFPENHEDQIE
AsV-C1-             EPGDDHRPIFRAEGPDDLPKTFNSWSLMAKNVEWWITKFADFCKWLKEKVFPENHEDQIE
AsV-B1-44C          D-CPENRPIFNSEGP----KDFNDWSLVAKNVKWWLEQFKSFFSWMRDKVFGGDS-DQVD
                    .   : :  :.:***    * ** *:*:***::**: :: .* .*:: *:*  :  *:::

AsV-A1-HUN          AMESVRDRMVMSLAAADKHLVTLKADKAYATSKAARDYHLKITNEIIDLNAMDLGPDFRD
AsV-A1-USA-IN1      AMESVRDRMVLSLAAADKHLVTLKADKAYATSKAARDYHLKITNEIIDLNAMDLGPDFRD
AsV-C1-45C          QVEKLKDTLALTICQADKHLVAMRTDRDYATSYEANIYHQNLMVKLVDLNAKDWGPDFRE
AsV-C1-HUN          QVEKLKDTLALTICQADKHLVAMRTDRDYATSYEANIYHQNLMVKLVDLNAKDWGPDFRE
AsV-C1-             QVEKLKDTLALTICQADKHLVAMRTDRDYATSYEANIYHQNLMVKLVDLNAKDWGPDFRE
AsV-B1-44C          EVESRFEQITTTLAQCDAHLVAMATDKAYATGKSARQYHEVLSARLRDLSLLPLGGPLSQ
                     :*.  : :. ::. .* ***:: :*: ***.  *. **  :  .: **.    *  : :

AsV-A1-HUN          LGTKIGQILNRLQSVTFDSVDAGSMRQEPLGIWISGEPGCGKSFLSHLIIKHLKEKKGFS
AsV-A1-USA-IN1      LGTKIGQILNRLQSVTFDSVDAGSMRQEPLGIWISGEPGCGKSFLSHLIIKHLKEKKGFS
AsV-C1-45C          LSIKLGQVLQRMQAVNFESSNMNGLRAEPVGIWISGGPGCGKSFLAQILIKHLRVAHGFS
AsV-C1-HUN          LSIKLGQVLQRMQAVNFESSNMNGLRAEPVGIWISGGPGCGKSFLAQILIKHLRLAHGFS
AsV-C1-             LSIKLGQVLQRMQAVNFESSNMNGLRAEPVGIWISGGPGCGKSFLAQILIKHLRVAHGFS
AsV-B1-44C          VSQKINYMQGRMGKVTFDCVEAGSARMEPLGIWISGGPGAGKSFLAQRIMKELKTSLGYD
                    :. *:. :  *:  *.*:. : .. * **:****** **.*****:: ::*.*:   *:.

AsV-A1-HUN          VFCNPSGSDHMDGYNGQEIHYFDDLGQIREEADIKLMCQLISSQQFIVPKADLTSKGTLY
AsV-A1-USA-IN1      VFCNPSGSDHMDGYNGQEIHYFDDLGQIREEADIKLMCQLISSQQFIVPKADLTSKGTLY
AsV-C1-45C          TYNHPTGSEHMDGYTGQEVHYIDDMGQIREEEDMKLLCQLISSQPFIVPKAELLSKGTQY
AsV-C1-HUN          TYNHPTGSEHMDGYTGQEVHYIDDMGQIREEEDMKLLCQLISSQPFIVPKAELLSKGTQY
AsV-C1-             TYNHPTGSEHMDGYTGQEVHYIDDMGQIREEEDMKLLCQLISSQPFIVPKAELLSKGTQY
AsV-B1-44C          VYYHPTGSKHMDGYNGQEIHYIDDLGQLREEGDVALLCQMISSASFIVPKADLNSKGTLY
                    .: :*:**.*****.***:**:**:**:*** *: *:**:***  ******:* **** *
```

```
AsV-A1-HUN         NAKVVIATTNKNEFDSTVLNDSGALRRRFPIRLHVRPHSFYTTQDGRLDLNRAMKDGDID
AsV-A1-USA-IN1     NAKVVIATTNKNEFDSTVLNDSGALRRRFPIRLHVRPHSFYSTQDGRLDLNRAMKDGDID
AsV-C1-45C         RAKIVIATTNRTSFDTMVLSDTGALQRRFPIRLKIRAHSFYTKADGTLDVALAMERKAFE
AsV-C1-HUN         RAKIVIATTNRTSFDTMVLSDTGALQRRFPIRLKIRAHSFYTKADGTLDVALAMERKAFE
AsV-C1-            RAKIVIATTNRTSFDTMVLSDTGALQRRFPIRLKIRAHSFYTKADGTLDVALAMERKAFE
AsV-B1-44C         NSKVVIATTNRTSFDTQVLTTPDALRRRFPIELSIRPHAFYCTLDGRLDMHKVMVEKAWD
                   .:*:******:..**: **. ..**:*****.* :*.*:** . ** **:  .*     :

AsV-A1-HUN         PG-CWEIDVGS-GRSCWQTLNWDILIHEIEDELINREHINKFFSQGA--IFESDEVEVVP
AsV-A1-USA-IN1     PG-CWEINVGT-GRSCWQTLNWDILIHEVEDELINRENINKFFSQGA--IFESDEVEVIP
AsV-C1-45C         DGSCWEINVGNDSRPCWQTLNWEVLTDEIDRMVATRSSIASLFNQGARCDLESDEVELIP
AsV-C1-HUN         DGSCWEINVGNDSRPCWQTLNWEVLTDEIDRMVATRSSIASLFNQGARCDLESDEVELIP
AsV-C1-            DGSCWEINVGNDSRPCWQTLNWEVLTDEIDRMVATRSSIASLFNQGARCDLESDEVELIP
AsV-B1-44C         DCSCWEVNVSKDGRPCWQTINWDVLKDEISKQLATRMSVLTFFSQGP------SLFECEE
                      ***::*.. .*.****:**::* .*:.  : .*  : .:*.**.       . .*

AsV-A1-HUN         EQGPGSVNKSTVTKLKNWINSLIDRAKSFFERNKCWFYLGSALATLATLVTTALPAARSY
AsV-A1-USA-IN1     EQGPGSVNKSTVTKLKNWINSLIDRAKSFFERNKCWFYLGSALATLATLVTTALPAARNY
AsV-C1-45C         ESGPGSVNPKTMDKVKNWLNSLLSDALSWWERNKQWLLLVSALSTLASLAIGAIPAYRAI
AsV-C1-HUN         ETGPGSVNPKTMDKVKNWLNSLLTDALSWWERNKQWLLLVSALSTLASLAIGAIPAYRAI
AsV-C1-            ETGPGSVNPKTMDKVKNWLNSLLSDALSWWERNKQWLLLVSALSTLASLAIGAIPAYRAI
AsV-B1-44C         EEDEAPYSEQTKVRVSSWLRQLLDKARGFFERNKCWLVGISAVATLLSVAVSVAPKLFGN
                   * . .. . .*  ::..*:..*:  * .::**** *:   **::** ::.  . *

AsV-A1-HUN         LSNLYSGEPTRAKVTRVTREFQSEGPSYYSLKDRLVEVGETGSTGLALGGKVVLSFGHND
AsV-A1-USA-IN1     LSNLYSGEPTRAKVTRVTREFQSEGPSYYSLKDRLVEVGETGSTGLALGGKVVLSFGHND
AsV-C1-45C         QNQLYHGEPSAKPKDRVKRDFKPEGPNFHSLKDRMVEIGTSHSTGLLLCDKKVLTFGHNT
AsV-C1-HUN         QNQLYHGEPSAKPKDRVKRDFKPEGPNFHSLKDRMVEIGTSHSTGLLLCDKKVLTFGHNT
AsV-C1-            QNQLYHGEPSAKPKDRVKRDFKPEGPNFHSLKDRMVEIGTSHSTGLLLCDKKVLTFGHNT
AsV-B1-44C         SS-VYEGSP-QQLKPKVYREFRSEGPNLFHLKDRLVEVGSSGSTGLILGGKQVLTYGHNM
                    . :* *.*      :* *:*:.***. . ****:**:* : **** * .* **::***


AsV-A1-HUN         DSKFIIYKDQEHPVVKEENISVNNSPQDLALLTVQTPYQFKELRRKIYADVYRGDGFLLF
AsV-A1-USA-IN1     DSKFITYKDQEHPVVKEENISVNNSPQDLALLTVQTPYQFKEIRRKIYADVYSGDGFLLF
AsV-C1-45C         DCGFITHKDQTFKVTSETYISVSGCDQDLKILEVETPYQFKNCSHKIYSGNYKGDGNLIF
AsV-C1-HUN         DCGFITHKDQTFKVTSETYISVSGCDQDLKILEVETPYQFKNCSHKIYSGNYKGDGNLIF
AsV-C1-            DCGFITHKDQTFKVTSETYISVSGCDQDLKILEVETPYQFKNCSHKIYSGNYKGDGNLIF
AsV-B1-44C         DRSFIKHKDIVYSVTKVEWIKVNDSEQDLAILTIDTNLQFKQLVNKVYSGEYHGDGYLLY
                   *  ** :**  . *..   *.*... *** :* ::*  ***:  .*:*:. * *** *::

AsV-A1-HUN         LKKGTLIAHQVKRITPCDNIMTQQGHQTQFAYRYQVNSASGWCGGVLVGIVGGNPMILGM
AsV-A1-USA-IN1     LKKGTLIAHQVKRITPCDNIMTQQGHQTQFAYRYQVNSASGWCGGVLVGIVGGNPMILGM
AsV-C1-45C         LRNNQLIIKDVFRIREKQGIGTIDGTYTHSAYAYSARTGSGSCGGILVGYVSGNPIILGM
AsV-C1-HUN         LRNNQLIIKDVFRIREKQGIGTIDGTYTHSAYAYSARTGSGSCGGILVGYVSGNPIILGM
AsV-C1-            LRNNQLIIKDVFRIREKQGIGTIDGTYTHSAYAYSARTGSGSCGGILVGYVSGNPIILGM
AsV-B1-44C         FRDNSLLATQVTGIRPFDPIATQEGHITCRTYCYHAKTARGSCGGVLVGMVGGNPMVLGL
                   ::.. *:  :*  *   : * * :*  *  :* * ..:. * ***:*** *.***::**:

AsV-A1-HUN         HVAGNGSHGIAARIFPNFSQGIVTQRMPN----TELYFQPRRSEIYPSPANDGTSNVEPP
AsV-A1-USA-IN1     HVAGNGSHGIAARIFPNFSQGIVTQRMPN----TELYFQPRRSEIYPSPANDGTSSVEPP
AsV-C1-45C         HVAGNGDTGIAARLYPCFAQGVTMHKWKQEQMFDTNYHQPRRSKFSPSCFFD-TGAQEPA
AsV-C1-HUN         HVAGNGDTGIAARLYPCFAQGVTMHKWKQEQMFDTNYHQPRRSKFSPSCFFD-TGAQEPA
AsV-C1-            HVAGNGDTGIAARLYPCFAQGVTMHKWKQEQMFDTNYHQPRRSKFSPSCFFD-TGAQEPA
AsV-B1-44C         HVAGNGHQGIAARVERYLWQSQGTVVKIEP---GTVYHQPRRSRIVPSPVYC-DSALAPA
                   ******  *****:   : *.       :       *.*****.: **      .   *.

AsV-A1-HUN         VLSNRDRRLETPIDDITKHNADRHKMNRFNPPMDAFQVAKSNVISELASIVEPCYHMTYD
AsV-A1-USA-IN1     VLSNRDKRLEEPIDDITKHNADRHKMNRFNPPMDAFQVAKANVISELASIVAPCYHMTYD
AsV-C1-45C         ILSNRDPRNPG-VADITKHNADKLTGNVFDPPEDAFALAKSRLIGSMSAHIEPEGQATFE
AsV-C1-HUN         ILSNRDPRNPG-IEDITKHNADKLTGNVFDPPEDAFALAKSRLIGSMSVHVEPEGQATFE
AsV-C1-            ILSNRDPRNPG-IEDITKHNADKLTGNVFDPPEDAFALAKSRLIGSMSAHIEPEGQATFE
AsV-B1-44C         VLSRADPRLEVPVEDITKRAAAKYVGNIFKPPEDCFIAAKAHVTRLLSTVVPPVGSLEYR
                   :**. * *    : ****: * :   * *.** *.*  **:.:   ::  : *     :
```

```
AsV-A1-HUN          QAVDSTLLPIVWTTSPGLEFKGKTKRQLVDDPGFKERVMKLYRSFAGG-NSAPPQVKYTT
AsV-A1-USA-IN1      QVVDSSLLPIDWTTSPGLEFKGKTKKQLIDDPAFKERVMKLYKSFAGG-NSAPPQVKYTT
AsV-C1-45C          EAVSSELLPIDWGTSPGDKYRGKTKAELVDDKKFRADVHNLVKRFNGDPNREPVDVYFTC
AsV-C1-HUN          EAVSSELLPIDWGTSPGDKYRGKTKAELVDDKKFRADVYNLVKRFNGDPDREPVDVYFTC
AsV-C1-             EAVSSELLPIDWGTSPGDKYRGKTKAELVDDKKFRADVYNLVKRFNGDPNREPVDVYFTC
AsV-B1-44C          EAIDNSILPIDWSKSPGIKYKGMSKRQCVQDQSFKRDVLHLLL-------AQNPEVEFVT
                    :.:.. :*** * .*** :::* :* : ::*  *:  * :*             :* :.

AsV-A1-HUN          YLKDEVRIKEKVKKGATRTITASSFDYTIACRMIFGNIFRQLFGNGLPAGFAPGMNPYTQ
AsV-A1-USA-IN1      YLKDEVRSKEKVKKGATRTITASSFDYTIACRMIFGNIFRQLFGNGLPAGFAPGMNPYTQ
AsV-C1-45C          YLKDELRPKEKARACKTRVISAANWDYTIATRMVAGPILRQLYAWGREFGFGPGLNPYSH
AsV-C1-HUN          YLKDELRPKEKARACKTRVISAANWDYTIATRMVAGPILRQLYAWGREFGFGPGLNPYSH
AsV-C1-             YLKDELRPKEKARACKTRVISAANWDYTIATRMVAGPILRQLYAWGREFGFGPGLNPYSH
AsV-B1-44C          YLKDELRKLEKIKQGKTRSIEAASFDYTIACRMLFGQIMMHLFVKGREVGFGPGINPYTE
                    *****:*  ** :   ** * *:.:***** **: * *: :*:  *   **.**:***:.

AsV-A1-HUN          FDELYDSCWLNVICLDYSKFDASLSKDLMEHAIEVVSCFSEDPMSVIRAFQPTLISQERV
AsV-A1-USA-IN1      FDELYDSCRLNVICLDYSKFDASLSKELMEHAIEVVACFSEDPMSVIRAFQPTLISQERV
AsV-C1-45C          FDDLYDKILPFVICLDFKGFDGSLSSDLMFEAAQVIACFSTKPEAIMASAELTIGSTERV
AsV-C1-HUN          FDDLYDKILPFVICLDFKGFDGSLSSDLMFEAAQVIACFSTKPEAIMASAELTIGSTERV
AsV-C1-             FDDLYDKILPFVICLDFKGFDGSLSSDLMFEAAQVIACFSTKPEAIMASAELTIGSTERV
AsV-B1-44C          FDELFDRLHPHCLEIDYSGFDGSLSRELMIHCLDVLVSFHESPETCRKLAMLTIDSVERV
                    **:*:*      : :*:. **.*** :** .. :*: .*  .* :       *: * ***

AsV-A1-HUN          SDELWEVRGSMPSGSPWTTMINTICNLLMCKTYLLDMGHDLTKTYVVCYGDDCVISVDQC
AsV-A1-USA-IN1      SDELWEVRGSMPSGSPWTTMINTICNLLMCKTYLLDMGHDITKTYVVCYGDDCVISVDQC
AsV-C1-45C          SDEVWYNYGGMPSGSPWTTTLNTICNLLMCYTYLLDMGHCWSETFVVAYGDDVVISANIK
AsV-C1-HUN          SDEVWYNYGGMPSGSPWTTTLNTICNLLMCYTYLLDMGHCWSDTYVVAYGDDVVISANIK
AsV-C1-             SDEVWYNYGGMPSGSPWTTTLNTICNLLMCYTYLLDMGHCWSETFVVAYGDDVVISANIK
AsV-B1-44C          SDEVWHVSGGMPSGSPLTTLMNTVCNMLMCYTWAFYQGYSCEEVFVAAYGDDVIISAKKK
                    ***:*   *.****** ** :**:**:*** *: :  *:   ..:*..**** :**..

AsV-A1-HUN          HKLEGIEQWFMDKFGATVTPEDKSGKINWRFKNKLKFLKRTPMQLDWIPKIVGALDIDSM
AsV-A1-USA-IN1      HRLEGIEQWFMDKFGATVTPEDKSGKIKWRFKNKLKFLKRTPMQLDWLPKIVGALDIDSM
AsV-C1-45C          HNLEGIENWFKTKFGATVTSSDKQSKITWTTKNNMEFLKRRPKELEFLPKIVGALDLDNM
AsV-C1-HUN          HNLEGIENWFKTKFGATVTSSDKQSKITWTTKNNMEFLKRRPKELEFLPKIVGALDLDNM
AsV-C1-             HNLEGIENWFKTKFGATVTSSDKQSKITWTTKNNMEFLKRRPKELEFLPKIVGALDLDNM
AsV-B1-44C          SNITDIVQCFKSWFGASITPAIKEGDISWAPKHQVVFLKRRPKQLDFAPKIVGALDLQNM
                     .: .* : *   ***::*.  *...*.*  *::: **** * :*:: ********::.*

AsV-A1-HUN          MDRIQWTKGHFQEQLNCFYYELALHGEDTYNEARKSIAFRCPELVHPTYQCALQTIKPMV
AsV-A1-USA-IN1      MDRIQWTKGHFQEQLNCFYYELALHGEDTYNEARRSIAFRCPELVHPTYHCALETIKPMV
AsV-C1-45C          LQHLEWTKGHIQDQLNSFYLELALHGREKYEEIRAKLAPRAPQLVHPTYACAKATITPMV
AsV-C1-HUN          LQHLEWTKGHIQDQLNSFYLELALHGREKYEEIRAKLAPRAPQLVHPTYACARATITPMV
AsV-C1-             LQHLEWTKGHIQDQLNSFYLELALHGREKYEEIRAKLAPRAPQLVHPTYACAKATITPMV
AsV-B1-44C          LDRIQWTTGDFQSQLNSFYIELALHGRETYNSVRAFLANKAPHCVHPTYDTAVLTVQPIV
                    ::::**.*.:*.***.** ******.:.*:. *  :* :.*. *****  *  *: *:*

AsV-A1-HUN          SLM
AsV-A1-USA-IN1      SLM
AsV-C1-45C          AIL
AsV-C1-HUN          AIL
AsV-C1-             AIL
AsV-B1-44C          GFL
                    .::
```

Grusopivirus Polyprotein Alignment

```
Grusopi-A1-yc5        -MRMETLGNVITTKVSESLSTALESVVQDTPGIGSTVEFKSANAIVATSEPTVGTVPPLS
Grusopi-A2-yc3        ---MENLMNKVETSITDVLSTAVESIEESIPGMGQTLNHQSANAVMTTEDPTVTTAPMNA
Grusopi-C-LoPV-1      -MQMETIAKEVTSEVTSVLSTAVETMVEEVPGLGKTLNHQSDNAIMTTEDPTIAVAPLKS
Grusopi-B1-yc6        MMASETLGNVVENSITKALSNVEEVAKEVVPGLGDTINHPSVNAVIATSAPTVDGTLPEP
YC-4                  ---MESIGNIVMNEVTHALSNASEVVKEVVPGLGDTLNHPSTNAMMATDQPTVDAAPPDA
                          *.: : : ..::  **.. *   :  **:*.*::. * **:::*. **:  .   .

Grusopi-A1-yc5        TDGSADDYLSCAYTVDTNVQNIEKMILL--GSYRIGNNAARHSIIFKLPMPDSFFSDFTH
Grusopi-A2-yc3        TDGSADDYLSCAYRIDAGDDNVQKMVLLKTGSLQTSTSTKIGTTAAKLHFPHCMFQDYTY
Grusopi-C-LoPV-1      TSGSADDYYSCSYVVDAGESNVQKMILL--ETGQITTNKDPYTLVTQLRFPQCFFDHSSK
Grusopi-B1-yc6        TDGSADDFLSCAYQIDAGDGNVQKMVLLANGRTQVSTMN-PATPIINIEVPHTFFNTSTC
YC-4                  TDGSTDDFLSCAYKVDAGDSNVQKMVWLYGGEQTVGSS---WNMLFKIPVPHQFFASSSY
                      *.**:**: **:* :*:.  *::**: *          .     .    :: .*. :*    :

Grusopi-A1-yc5        PAYGQSRYFHCLRSGYNFKLLIAGAFGAQGSIILFYLPPVPRGVLQILPHNMNQSGITHK
Grusopi-A2-yc3        PAAGQSHYFCYLRAGAHFKICINAAPGSVGLLAMVYMPPGYEHYFHVNEQ---------Q
Grusopi-C-LoPV-1      PAQGQSQYFDLVRCGCHIKLCVHATAGSLGGLVLQFLPPGFENYQEHSLS--------YD
Grusopi-B1-yc6        PAAGQAKYFGLCRCGYKITFQVNLPRGSSGAVVLWYAPPYYSHYMPGDTTNR--------
YC-4                  PAFGNSRYFTLVRCGIKVMLEIDLPAGSSGAVIMFYLPPPYGSLIT-SAQDT--------
                      ** *:::**   *.* :. : :  . *: * : : : **

Grusopi-A1-yc5        LNNYFDPDTILYGPHVLMDVARNTEVTLSLPYISYKNYEHIYGDSRRGYLVCAVYSDVYL
Grusopi-A2-yc3        ADDRVDLKTVLYLPHVLIDVSKNSQMSLTVPYMNYKNYFDFRNTAVYGQLLLLYLTPYHP
Grusopi-C-LoPV-1      KKPTYDPDTMLYAPTAILDISRNSEVSITVPYVHFQNYADFRLSRTYGQINVYMLTKASL
Grusopi-B1-yc6        ---KFDYDTILNAPHVIMDVALNTQVTLVVPYVSFLNYTNHHLINSGSTFHVTVIQPIRS
YC-4                  ---KFNLDTMMYNPHTIIDVARNTQCTLVVPYINFTNYINYRDLNSSGWIVAIELMPVRT
                           : .*::  * .::*:: *:: :: :**: : ** .        . :

Grusopi-A1-yc5        PQTS--HIDVSLYGELLDMDLQVPRKVLES-----GFLNQGRKFRPRRTQPRMPPVQKDT
Grusopi-A2-yc3        PQSGNNKVAYAIYGEFLDLDFQCPRFLPTAP---TNLSDQGRKVYPRRKHPKKPKSSPDV
Grusopi-C-LoPV-1      SNNAVNHIPYAIFGELLDLDFQCPR----------FFKNQGRKVLPKRRDPQPPKGHKDV
Grusopi-B1-yc6        PLSSATHVDWAVFGELLDLDFQAPKFFS-----------QGRRIYLKRKPPKPEKSLPDV
YC-4                  IQTSMNKYVWNVYGELLDLDFQAPHLVQDNWALPSTLQDQGRKIVPKRKPPPVPPSKPDV
                        ..  :    ::**:**:*:* *:              ***:.  :*  *        *.

Grusopi-A1-yc5        STVIINPGPGACNLASHDVIARTESLSLAGETTGVDFRTAGCAAAIPSLRTILRRWTHIA
Grusopi-A2-yc3        PHVLIQPGYGTMNCSNAVAANRAESIALDNDNSAMDLRTSGCASVSHSLMDVLRKWTVVR
Grusopi-C-LoPV-1      NHVLVQPGYGALNMSNAVAVDRAESLSLCNDTSGVDFRTAGAKASIISVREVARRWTIYH
Grusopi-B1-yc6        NHVLVQPGYGALNTSNSVAAGRAESLGLCNDISSIDFRTSGCASASTHLSDLYRKWNICY
YC-4                  KHVLVQPGYGALNSSNAVACDRAESISLCNDVSAMDLRTAGCASAMTSVNDLARRWTIYY
                        *:::** *: * :.   .  *:**:.* .: ::*:**:*. :    :  : *:*.

Grusopi-A1-yc5        QTSYAISSTAGSRLWEENVVFNHGIFRQLADKFLSWRGSLEFRVLVYSSTLHRGKLALSW
Grusopi-A2-yc3        RISWNSSAGVGVCINTIPVDFDTGIWNCVKDCFAYFRGGLEFKMVIASSVFHQGMIQLSW
Grusopi-C-LoPV-1      FFDITESMTTGETVEVVPLQLNKGIFKYLADSFAYFKGSLEIKLMVFASTFHKGKIQMTW
Grusopi-B1-yc6        SGTWLASYTSGQVLRSSFLQFNFGIFRYLADSFAFFRGSLEYKVLVFTSKFQTGKYQLGW
YC-4                  RGTWNANSTRGQVIHRQKFRLDKGIFKYLADSFAFFRGDLELKVMVFTSKFQKGKYQLSW
                            .   *  :    . :: **:. : * *  ::*.** :::: :* :: *    : *

Grusopi-A1-yc5        YP-MDVTGSQSTNYPYYRNAISMVFDVTDASPVLVAPFSSESWRASTAATFGVLSLHALT
Grusopi-A2-yc3        YP-GGSN--SDPNLYHMRNAIYITQDVRSSGPILLAPFTCTSWRRRFNGGYGNLHINVVN
Grusopi-C-LoPV-1      YPRTCPAAKGDVTYPYTRNGIFITQDMTSVGPILTLPFTSTTWRRSMVK-YGSITINVVN
Grusopi-B1-yc6        YPYG-PANDGPRNLSTMRNSLFMVGDATSTGPILTLPYTCDTWRRPFTT-YGYLTLCVVN
YC-4                  YPYGNVMGVSVLAQESTRNSVFLTSDASTTGPILKIPFTSASWRRGFQD-YGTLVISVVN
                      **                **.: :. *    .*:*  *::. :**      :* : : .:.

Grusopi-A1-yc5        PLSATHSTVAKTAVSVHVLMRVGKDFQAFCPRSGTVRFKTLVDSTTSTPSLFLSTSVNDG
Grusopi-A2-yc3        PLTYNNATTS--HVYILLMMRAAKDFELMSPTYGALSFGTNSYSPSMPARLVEQVSQNE-
Grusopi-C-LoPV-1      PLTRNGNCTP--TVHCVLMLRAGPDFTVMVPSFGSQKWVAPPCVDFQDDFTLLKEERRLL
Grusopi-B1-yc6        PLTYNSNTFS--GAYYVIMARAGPDFKVMCPHFGTYKWDSTSVGDSIVNYPRGLNPTRDE
YC-4                  PMTYNGNTFS--SAYFIILLRASDNFEVLCPFYGGYQWVGSSYLGDQDEYPMLISGNRAR
                      *:: .    .   .   :: *.. :*  : *  *    :                     .
```

```
Grusopi-A1-yc5        NV-------------------DDSTSVDNDNLDNQGNP-DIQMALEQRGESGNDEDQFDI
Grusopi-A2-yc3        -------------------------EEETMTLSDQGN-----------DNNPWEED----
Grusopi-C-LoPV-1      G----------------------VYTPEEELTDQGN--------EEVVENPWDSE----
Grusopi-B1-yc6        IG------------------LSRAIEEHEAKIAAELDTNLVHDSQGLHDSQGNP--WDDE
YC-4                  LQPVYHDVEHFQRTGKIVPSKATRLAAREARLECGCDIGPECPTDDECEEHENQSLWEED
                                                     :    :            .

Grusopi-A1-yc5        DPTLAINFETTFIPIQSVDHLDFKYLFSRASYVGRVAGSTGNTKHRILNLAPPLKGILSL
Grusopi-A2-yc3        EPTPFVNFETDKIATLSVNHTNLEACFGRMMYSISLTKPHG-GNFVNTEIPIPLHGVQKL
Grusopi-C-LoPV-1      EPVPFLNFERKEIATLSDDHADLSNCLGRMTFVTSLRPSSS-DHFVFKKLPMPAGGVQRL
Grusopi-B1-yc6        EPTNFINFEVMNVETMSEDHGELRHCLGRMTYATTIDSPSN-TRFAVKPLPIPLYGVARI
YC-4                  EPTPFINYETLNVKTISDQHADLRNCLGRMTYTTTITKPQS-TNFVARRLPCPITGVARI
                      :*.  :*:*   :   * :* ::   :.*  :   :  . .  ..    :. *  *:  :

Grusopi-A1-yc5        TRVAVYFTGELMITVSNISETSILVSHSWYNSYHYSSVDQYLTDGAIVIPPHEIKQFRVP
Grusopi-A2-yc3        ARCFAYMTGELIIGIVNRSTTSALMAHSYFEESVFNHWNQVFTTGALVIPPGENKVFKVP
Grusopi-C-LoPV-1      SRGFAYFSGEIIIGVHNQSSKSAMVTHSYWERTKFTHWDQLCTSGAIIIPPGESKVFKCP
Grusopi-B1-yc6        IRGFAYWSGNLIMGIHNRSTKSAIVAHSYYDSGLYNHWNQLFTTGAVIVPPNESKVVKIP
YC-4                  TRGFAFWSGEVTFSVYNRSTAAVLVGHSFVKENVFTHWNQIFSSGAIIVPPKEAKVFKVP
                       *  .: :*:: : : * *  : :: **: .   :.  :*  : **:::** * * .: *

Grusopi-A1-yc5        YYAKNPLSTLTDPYAFGFLHVYVDTDVGQADVFLSLVNPNLFQLTPLP--LTTTAAR---
Grusopi-A2-yc3        YYAETPFRVTAAANACGMLHGYLTD--GQLDVLISLRNVQFLVPICMP--LCAGRVN---
Grusopi-C-LoPV-1      FYSEEPLRPTNVNEALGYVHAYVVD--EQIDIFVSLRHVDFFFIMPLPKSEATIAVK---
Grusopi-B1-yc6        YYCQYPFMPTAAPDACGHVHTYQMD--GSIDIFLSFREANFFFPIPLPLTTTMLTTGDTD
YC-4                  FYSPTPFRPTASRDSLGTFHAYLVD--GNLDVFISFRNVQFFQPMMLPNDT--AVEGNLL
                      :*.  *:       : * .* *      . *:::*: . :::    :*

Grusopi-A1-yc5        --------------EISDYAISEVEEG----VDDLEDQGNTCEELHLTVRRKQCIASVEL
Grusopi-A2-yc3        --------------TLTNLPFPP---------CALENQGNGCEEMKITMRRKHCIGSVEV
Grusopi-C-LoPV-1      --------------DQQEKRKSNSKQTGDLFLCGMEDQGLGCGEMKATHKRPECLHSLEQ
Grusopi-B1-yc6        AKDGEIIQPLSLRLDGIDISNTSRDPAEMLELLSMDSQG-----FDYCRNPRKAIRSCGH
YC-4                  ITDG---QDVLSTTEGHELLET----AQLLEKLQMDNQGP-CAETRIVVCRTKCKRDIPT
                                       :   .            ::.**             ..  .

Grusopi-A1-yc5        HKSCRLHYFQPRCCKCQEWEYKEFIGPEPICLGPYKDVRAKFYCQCKKGIHHFQCGDHFG
Grusopi-A2-yc3        HKSCRLHYFQPRCCKCQEWDYKDFIGPEPICLGPYKDVRPKFYCQCKKGIYHFQCDEHFG
Grusopi-C-LoPV-1      HRSCHRHYFIPRCCKCQEWEFKKYIEAPGLCMEPFKEIRPKFYCVCKKGIHHFQCEDHYG
Grusopi-B1-yc6        EKTCDVHMFSKLCCDCSPPVPRTKLED---CRGNLLVCRPRYFCSGQ---NHESCTAHFR
YC-4                  HKACRLHEYCDACCVCEPQRRVLEDAP---CSESSVIVRPRYYCTLKG--DHSQCDVHKG
                      .::*  * :   ** *.             *       *.:::*  :    * .*  *

Grusopi-A1-yc5        RQMCCGCAHIYGWLDQAEKDYFEKHVKPWRSQEDLSKEGIEPNPGPFQYVACDLTAPVFK
Grusopi-A2-yc3        RQMCCGCAHIYGWLDQTERDFFEKHGKPWKSQEDLSKEGIEPNPGPFQYVACDLTAPVFK
Grusopi-C-LoPV-1      RQMCCGCAHTHGWLDEFERQYFEER-SPHPTQKELGQFGVETNPGPHH--SCKMDGIWIS
Grusopi-B1-yc6        RQICCTCSEKFGQLDYFEMKYFSSE-----------------------------------
YC-4                  RQICCQCARYTGWLDIPERQYFSPK-----------------------------------
                      **:** *:.  * **  * .:*. .


Grusopi-A1-yc5        CQPHRFCDFKSQSHQNCPQHNSPDSIMCCLCSARLRITDNRYKETDAKWLSRYGVEMNPG
Grusopi-A2-yc3        CQPHRFCDHKSQSHQNCPQHHSPDSFMCCLCSARLRITDNRYKETDAKWLSRYGVEMNPG
Grusopi-C-LoPV-1      CLPHSWCDKKNVIHQNCQKHVWADVSMCCLCSHHQRENNSNYSERDAKHLSRYGIEMNPG
Grusopi-B1-yc6        ------------------------------------------------------------
YC-4                  ------------------------------------------------------------


Grusopi-A1-yc5        PVLTSQEAVAHFCATLLDPCDQKPEPMVRVSAKPKCWIMRFHFDCENHQHLAKEMCCLCA
Grusopi-A2-yc3        PVLTSQEAVAHFCATLLDPCQQRPEVMVRVSAKPKCWTMRFHFDCENHQHLAKEMCCLCA
Grusopi-C-LoPV-1      PVLTTRESVAHFTATVLNPCDKRKPQMIRVKQKPKCAEMEYHFCCEDHVSLSRELCCVCT
Grusopi-B1-yc6        ------------------------------------------------------------
YC-4                  ------------------------------------------------------------
```

```
Grusopi-A1-yc5        AGSRVTQDLYAATNQDQLSNQGIESNPGPTLVYKDRGMYKHYGVQVGDKIFHINTENILD
Grusopi-A2-yc3        AGSRVTQDLYAATNQDQLSNQGIESNPGPTLVYKDRGLYKHYGVQIGDKIFHINTENILD
Grusopi-C-LoPV-1      R--------WSPTMQSELGKYGIEKNPGPTLVCKDRGLYKHYGVQIGDVILHVSTENVLD
Grusopi-B1-yc6        -------------VEESCDTLSGNLRENSSLFVSDGDDRYYAFRYQGNYFGFAATE-AIE
YC-4                  -------------AKEELSKYGIEPNPGPITVCCNLLDKKRVGFRFGHYIYELSDN-PLE
                                    :.. .. . : . ..  .  :           *. :     :  ::

Grusopi-A1-yc5        AVISGKANVMMEDNIMGWETVGDEYVTVAEKYLQAGSMPDFKFNVDQNCETWARHLLGDY
Grusopi-A2-yc3        AVISGKANVIMEDNIMGWETVGDEYVTVAERYLQAGSMPDFKFNVDQNCETWAKHLLGDY
Grusopi-C-LoPV-1      AIISGEAKILEDQDVMGWQAVSDEYITVAEKYIQAGSMPNFKFNVDNNCETWARHLLGDY
Grusopi-B1-yc6        AITRGTIELR-VLNDHSWRPIK----TIPEDWLLAGVEEVR--CVNSDGWNSKVHPL---
YC-4                  AVYQNIVNFRRTTASTVWTPIS----EEIREQLTNLIHTIDPMIVHLDGEVSPFNGP---
                      *:  .  :.        * .:        .  :           *. :      :

Grusopi-A1-yc5        GPTQGEILKHRLAQCVALGMLAQFAMSSQGLGSTVQGMMTKLTDLIFGNLENQIVKLVIR
Grusopi-A2-yc3        GPTQGEILKHRLAQCVALGMLAQFAMSSQGLGSTVQGMMTKLTDLIFGNLENQIVKLVIR
Grusopi-C-LoPV-1      GPTQGEILKRRLVQCVALGMLAQFAMSSQGLGDTIQGLMTKLTDLIFGNLENQIVKLVVR
Grusopi-B1-yc6        ----------LAQALFDS-------FSNQGFADGATALMSKLSSLVFGELEHQVMRMVIR
YC-4                  ----------NFQEAVKISILYGMQLEDQAIGGMLTGFLHKLADLIFGNLEETILKLVIR
                                     .         :..*.:..   .:: **:.*:**:**. ::::*:*

Grusopi-A1-yc5        AVVRILCYLILYIHSPNLMTTGVLAALLVLDATSITVDDPLKVMTKALINGDFTEFCAAV
Grusopi-A2-yc3        AVVRILCYLILYIHSPNLMTTGVLAALLVLDATSITVDDPLKVMTKALINGDFTEFCAAV
Grusopi-C-LoPV-1      AVVRILCYLILYIHSPNLMTTGVLAALLVLDATSISVDDPLKVMTKALINGDFSEFCAAV
Grusopi-B1-yc6        TVVRIVAYLILLAMNPTMITTGILATLLYLDSTTTQIDSPLKILCESLINGDFLAFCEAI
YC-4                  VVVRILCYVVLYMSNPTAINTGIVGTLVILDLTSISVDDPVRTMSKALINGDFHEFCEAL
                      .****:.*::*   .*. :.**::.:*: ** *:  :*.*:: : ::******  ** *:

Grusopi-A1-yc5        LEKCDIVDADALADTIPTFTSFVDQS--MEDEGPNPKPFNDWTTCAKNIQWWMEGIVKAF
Grusopi-A2-yc3        LEKCDTVDADALADTIPTFTSFVDQG--MEDEGPNPKPFNDWTTCAKNIQWWMDGVIKAF
Grusopi-C-LoPV-1      LEKCDTVDSEALMETIPTFTTFVNQG--LNDESPNPKPFNDWTTCAKNVQWWLDGIIKAF
Grusopi-B1-yc6        IERTNVDDPAILRSTIPEFTTMLG----QESQGANSK-FTVWTNTCKSIGWWLESLSRVI
YC-4                  LENCQQVDKATLTTTIPAFSAMAKGDLTFADEGKARD-FNDVTTAFKNITWWLDGLLKVI
                      :*. :  *   *  *** *:::        .:.   . *.  *.  *.: **::.: :.:

Grusopi-A1-yc5        KWIKDKLFPSDLNSLAKWMEENEDNIATTIALADEHLVLLKTDKEYATSSQTKKKHVALC
Grusopi-A2-yc3        RWIKEKLFPSDLNSLAKWMEENEDNIATTIALADEHLVLLKTDKEYATSSQTKKKHVALC
Grusopi-C-LoPV-1      KWIKEKLFPTELSSLAKWMEENEDNIATTIALADEHLVLLKTDKEYATSSQTRKKHLALC
Grusopi-B1-yc6        TWLREKVFPVEISKTARWLEENQEKLVLTMALADEHLLLMKTDKSYSLSSKAKEKHIALV
YC-4                  DWLSGKIFPKNLPKATEWLEENKEQLTLSLAYADEHLILMKTDKTYSVSDEARKKHVRLA
                       *:  *:** :: . :.*:***::::. ::* *****:*:**** *: *.::::**: *

Grusopi-A1-yc5        DTISGMMLKMKDDPRLNPMISRLNILMGKLQSINFEPEAEWTHRPEPLGMWINGAPGVGK
Grusopi-A2-yc3        DTISGMMLKMKDDPRLNSMISRLNILMGKLQSINFEPEAEWTHRPEPLGMWINGAPGVGK
Grusopi-C-LoPV-1      DTISGMMIKMKDDPRLFQMINRLNTLLGKLQSINFEPEAEWTHRPEPLGIWINGAPGVGK
Grusopi-B1-yc6        EMLVGAQIQIGTDTRVRDLNSKISYLISKLQAINFEPVTNWHHRSEPLGIWIYGDPGVGK
YC-4                  ELFTGAILRLEGLPSNNELLRRIHHVMAKLQSINFEPEANWTCRPEPLGIWVSGAPGVGK
                      : : *  :::   .    :  ::  ::.***:***** ::*  *.****:*: * *****

Grusopi-A1-yc5        SFLTNFITKRLKEKYGWNIYANATGSNHMDGYTDQEVHVFDDFGQQREETDYTLMCNLIS
Grusopi-A2-yc3        SFLTNFITKRLKEKYGWNIYANATGSNHMDGYTDQEVHVFDDFGQQREETDYTLMCNLIS
Grusopi-C-LoPV-1      SFLTNFITKRLKEKYNWKVYANATGSNHMDGYTDQEVHVFDDFGQQREETDYTLMCNLIS
Grusopi-B1-yc6        SFFVNLVTKFISRKFGWGVYANPTGSNHMDGYTDQEIHIFDDFGQLRSEEDYSLICNLIS
YC-4                  SFLVNYISKNVAKAKDWKIYANPTGSKHMDGYTDQQVHIFDDFGQVKDEQDYTLICNLIS
                      **:.* ::* : .  .* :***.***:********::*:****** :.* **:*:*****

Grusopi-A1-yc5        SVPFIVPKAEVTAKGTTYKGRAVLVTTNRRDFSSVALFDAEALERRFPFKYQIRPKPKYE
Grusopi-A2-yc3        SVPFIVPKAEVTAKGTTYKGRAVLVTTNRRDFSSVALFDAEALERRFPFKYQIRPKPKYE
Grusopi-C-LoPV-1      SVPFIVPKAEVTAKGTTYKGRAVLVTTNRRDFTSVALFDAEALERRFPFKYQVRPKPKYE
Grusopi-B1-yc6        SVPFLIPKAEVTAKGTPYNGKLVMVTTNRRDFSSVSLLDSDALARRFPIKLQIRPSRDYS
YC-4                  SVPYIVPKAEVTTKGTLYNGRLVLATTNRRDFSSCTLWDPEALERRFPYRYTIRPRAQYR
                      ***:::******:*** *:*: *:.*******:* :* *.:** **** :  :**  .*
```

```
Grusopi-A1-yc5        LNGKLNVALAMRDGALLSGNAWERNLGIAGLDKWEPLDGDTLMEEICTELMIRENIAKFM
Grusopi-A2-yc3        LNGKLNVALAMRDGALLSGNAWERNLGIAGLDKWEPLDGDTLMEEICTELMIRENIAKFM
Grusopi-C-LoPV-1      LNGKLNVALAMRDGMLLTGNAWERNLGIAGLDKWEPLDGDTLMEEICTELMIRENIAKFM
Grusopi-B1-yc6        IGGKLDVPKALRDGALLSGKAWEVNYGISGLDAWIQMEADVFLEDVCLQLRTRENVARMM
YC-4                  LGERLDVSAAMRDGALVNGTAWERNLGFPGVDKWEPLDGPTLLGEILDDLDGRRNIAAMM
                      :. :*:*. *:*** *:.*.*** * *:.*:* *  ::. .:: ::  :*  *.*:* :*

Grusopi-A1-yc5        NQGNSD---PLFAILEQSKIDFDWQSLEEQVAMFTRRPPKGKIQKFKVWVSDSVAKIKSF
Grusopi-A2-yc3        NQGNSD---PLFAILEQSKIDFDWQSLEEQAAMFTRRPPKGKIQKFKVWVSDSVAKIKSF
Grusopi-C-LoPV-1      NQGNGD---QLFAILEQSKLDFDWQSFEEQAAMFTRKPPKSKIQKFKSWVTESVNKIKSF
Grusopi-B1-yc6        NQSPENIPMNVFRHLEQQNFGFDSE-DDTQEPLFPQTSTSSVMTKGKKWIGDTLKKMKSF
YC-4                  NQGPD----KIFEGIEMTTMGFDFD-ELENGPQLFSPPPEQKLQKYRQWIVSAIKSGKSF
                      **.       :*  :*  .:.** :    : . :   ...  : * : *: .:: . ***

Grusopi-A1-yc5        VEKNRTWFLALGAIGSIISVCSFLIPRVSMFTQSFYEGSTKVAKLSKNFKVAAAQHNAKF
Grusopi-A2-yc3        VEKNRTWFLALGAIGSIISVCSFLIPRVSMFTQSFYEGSTKVAKLSKNFKVAAAQHNAKF
Grusopi-C-LoPV-1      VDKNRTWFIAMGAVGSIISVCSFLVPRLNLFSQSFYEGGVKVGKLAKNFKVEAARHNAKF
Grusopi-B1-yc6        ISKFGVFIGAGASLGVLLALIKLIKPKFID--NGFYDGTPNVRLVPKDFLVDVEKHNQKL
YC-4                  FQKFKKYIEAAGVLASVIGIVKFLRNHFAHMIHNVYDGQPVQAMTTKDFKIHVAEHNKKL
                      ..*   :: * . :. ::.: .::  :.    :..*:*       .*:* : . .** *:

Grusopi-A1-yc5        EDQ---------GLMDMRHICQRLVNLRGPKGTATGLALGGKTIITYGHEEFHSLEFVKD
Grusopi-A2-yc3        EDQ---------GLMDMRHICQRLVNLRGPKGTATGLALGGKTIITYGHEEFHSLEFVKD
Grusopi-C-LoPV-1      DDQ---------GLLDLRHICQRLVNLEGPHGEATALALGNKTVITYGHEEFTKLTYVKD
Grusopi-B1-yc6        REK--QKPMESQGFADLRPICNRLVNLKSDRGEATGLALFGKTVITYAHNNFTHCTYHQN
YC-4                  KEKEKQQPHEDQGLADIRHICTRLVNVRGQVGTVTGLAVGGKSIITYAHNNFEHMVHHGE
                       ::         *: *:* ** ****:..  * .*.**: .*::***.*::*    .  :

Grusopi-A1-yc5        TQVNANLSPPVAVRISDEPTDLALYQADTPFQFKNAYHLIHDEDYRGKGYLIWKNQTEYM
Grusopi-A2-yc3        TQVNANLSPPIAVRISDEPTDLALYQADTSFQFKNAFHLIHDDDYRGKGYLIWKNQTEYM
Grusopi-C-LoPV-1      TAMECELKEAVHVQVSGEPTDLAMYECKTSFQFKSASHLIYDQDYRGKGYLIWKHHGEYM
Grusopi-B1-yc6        KELNAPLISGIKVAYQGSTTDLAMYNVDLKYQFKNSMNLIATEDYHGRGYLVWKNHDTYT
YC-4                  SECEAQLLNPIRITYDMEQTDLAIYNVDMKYQFKDVDRLIHGEDYRGDGYLVWKHGRDYM
                      .  :. *   : :  . . ****:*: .  :***.  .**  :**:* ***:**:   *

Grusopi-A1-yc5        ILAVDNIRPAHPITTNQGTISSRVYMYNAKTGAGTCGGILVGLVNGNPKILGIHTSGNGV
Grusopi-A2-yc3        ILAVDNIRPAHPITTNQGTISSRVYMYNAKTGAGTCGGILVGLVNGNPKILGIHTSGNGV
Grusopi-C-LoPV-1      LLAVDNIRPAHPITTTQGTISSRVYMYNAKTGSGTCGGVLVGIVNGNPKILGIHTSGNGV
Grusopi-B1-yc6        MLAVEDIRPGPQITTIQGVVSSRTYIYKANTQRGTCGGVLIGFENGNPKILGIHTSGNGI
YC-4                  VMAVSDIRPGNTIVTKQGTVSSRVYVYKARTGPGSCGGVLIGTPNGNPKILGIHTSGNGI
                      ::**.:***.  *.* **.:***.*:*:*.*  *:***:*:*  ***************:

Grusopi-A1-yc5        TGAANRLYSFFDQGQVTKKISEDKPKYFQPRKSAYVPSPVHVYTDVGPPVLSKNDPRLEV
Grusopi-A2-yc3        TGAANRLYSFFDQGQVTKRISEDKPKYFQPRKSAYVPSPVHVHTDVGPPVLSKNDPRLEV
Grusopi-C-LoPV-1      TGAANRLYSFFNQGKVVKKEIEPRPKYFQPRKSAYVKSPVYQDSDVGPPVLSKNDKRLEV
Grusopi-B1-yc6        TGAANRLYNNLDQGLVVEKKYVG-NSYHQPRKTKFRPSPFYVNPRVSPAVLSNRDPRLID
YC-4                  TGAANRLYPHMNQGQVVRKTDGA-PVYHQPRRTKYSQSPVYTSSGVAPAVLSRNDPRLTK
                      ********  ::** *..:       *.***:: :  **.:  . *.*.***..* **

Grusopi-A1-yc5        EVDDITVRAAEKYIGNTFDPPPSIFEHAKARLAENLSKVLEYKPPPLTYDEATSTEILDI
Grusopi-A2-yc3        EVDDITVRAAEKYIGNTFDPPPSIFEHAKARLAENLSKVLEYKPPPLTYDEATSSEILDI
Grusopi-C-LoPV-1      EVDDVTVRAAEKYVGNTFDPPPTIFEHAKIRLAENLSKVLEYKPKMLTYEEATSTDILDI
Grusopi-B1-yc6        PIEDITKKAAMKYRGNTFDPPGFAFELAKIALFSRLYKVLP-RAKQISFEKATDSSYLAI
YC-4                  AIDDITVEAASKYVGNVFQPPDRAFKLAQTRFATNAYKVID-KHETQTYDWAIDPSNLPL
                       ::*:* .** ** **.*:**   *: *:  :  .  **:  :    ::: * ... * :

Grusopi-A1-yc5        DWTTSPGEKYKGKTKKELVVSESFKTDVLTQLANPNTYFVTYLKDELRSNEKIRNGNTRA
Grusopi-A2-yc3        DWTTSPGEKYKGKTKRELVASDSFKTDVLAQLANPNTYFVTYLKDELRSNEKIKNGNTRA
Grusopi-C-LoPV-1      DWTTSPGEKYKGKTKKELIAMPDFKQDVIDQLENPNTYFVTYLKDELRSSDKIKSGNTRA
Grusopi-B1-yc6        DWQTSPGHKYQGCTKKQLIDDEKFVADVAQQLNDPDTYFTTYLKDELRPNEKVAMGKTRA
YC-4                  DWTTSPGLKYTGKTKASLVFDTKFKEDVIQQLREPATYFTTYLKDELRPLEKIQEGKTRA
                      ** **** ** * ** .*:   .*  **  ** :* ***.********. :*:  *:***
```

```
Grusopi-A1-yc5        IEACNFDYTVAFRMVMGHHYKNIMNDVEQLSEICVGINPYVYFDTIVDSLYDYNLCLDYK
Grusopi-A2-yc3        IEACNFDYTVAFRMVMGHHYKNIMNDVEQLSEICVGINPYVYFDTIVDSLYDYNLCLDYK
Grusopi-C-LoPV-1      IEACNFDYTIAFRMVMGNHYRNIMDDVEQLSEICVGINPYVYFDTIVESLNEYNLCLDYK
Grusopi-B1-yc6        IEASNFDYVIAYRMVMGEIYKAIIEDVQCVSGIAVGMNPYEDFDELYYGLYDNNLCLDFS
YC-4                  IEACNFDYVIAYRQVMGPIYSDIYADVECISEIAVGMNPYTDFDTLIHSLYPYNLCLDYK
                      ***.****.:*:* ***  *  *  **: :* *.**:***  ** :  .*   *****:.

Grusopi-A1-yc5        KFDGSLSPQVMEAAVEVFSWFASNPDLVKAIHRPTIYSTNWVSNQVWTVEGGMCSGSPCT
Grusopi-A2-yc3        KFDGSLSPQVMEAAVEVFSWFANNPDLVKAIHRPTIYSTNWVSNQVWTVEGGMCSGSPCT
Grusopi-C-LoPV-1      KFDGSLSPQVMEAAVEVFSWFARDPDMVRKIHHPTIYSTNWVSNQVWTVDGGMCSGSPCT
Grusopi-B1-yc6        GFDGSLPPQLMEAAVEVLSHFHVEPDLVVKIHQPVIKSTNLVGDELWKVDGGMCSGAPCT
YC-4                  GFDGSLSPQLMRAAVDVLAYFHSDPEMVRKIHEPTIVSTNYVSGQIWEVDGGMCSGSPCT
                       *****.**:*.***:*:: *  :*::*  **.*.* *** *..::* *:******:***

Grusopi-A1-yc5        SILNSAVNILVISTMMMSYGYDPKELRLLTYGDDCVISVPYKVDISDFKKRLKNYFGMTV
Grusopi-A2-yc3        SILNSAVNILVISTMMMSYGYDPKELRLLTYGDD-----LYLIHI---------------
Grusopi-C-LoPV-1      SVLNSAVNVLVLSTMMMSYGYDPKEIRLLTYGDDCVISVPYKVDVSDFKWRLKNYFGMTV
Grusopi-B1-yc6        SVLNSICNYLAIATVLISYGYKDDDFKIYTYGDDCVVSVKEKTSMEDLEWRFAAYFGMTV
YC-4                  SVLNSACNILAISTVIISYGIEPSDFRCVTYGDDCIISLKEKICLDDLAWRFKAYFGMTV
                      *:***  * *.::*:::*** . .:::  *****          :

Grusopi-A1-yc5        TNFDKTEEFKWLSRGEISFLKRTPAILEGTTKLVGALDLDSMREKIQWTRSLNDFSSQLE
Grusopi-A2-yc3        ------------------------------------------------------------
Grusopi-C-LoPV-1      TNFDKTEEFKWLGRGEISFLKRTPTILDGTTKLVGALDLSSMKEKIQWTRSLNDFSSQLE
Grusopi-B1-yc6        TNFDKSSEISFLPPNQITFLKRSPLELYDTGKIVGALDLSDSHEKIQWMKSPETFEQQLD
YC-4                  TNFDKKSQIQFLQPMEVS------------------------------------------


Grusopi-A1-yc5        SFQLELALHGRGVYEKEIQELRKISPGSAWMPFDVALHRMKGICDIL
Grusopi-A2-yc3        -----------------------------------------------
Grusopi-C-LoPV-1      SFQLELALHGKEIYEREIQELRKLSPGSAWMPFKVALQRMKGVCDIL
Grusopi-B1-yc6        SYLLEVAIHGEQIYNQTVEAMREIAPSLDYPPFTYMKMRILVITGLM
YC-4                  -----------------------------------------------
```

Kunsagivirus Polyprotein Alignment

```
Kunsagi-A       MDPLSLTAGLVGDVVGGVAGAITGGGDPVGSLLSSGLSLLGLGSSSSTSSPTPSRDGGGP
Kunsagi-B       MDPFSFATIAGGELISSLLGSEAATG--VASAIDSGVDMLTSTLSNTSVSAPDTPSG---
Kunsagi-C       MDPFSLVTELAIGTLTESVPLLFGGG----VSEAAAASTTAAEVSPELTLPSNISAG---
                ***:*:.:      :        . *        :. .      *     ..    *

Kunsagi-A       SSIGPQLSAPDLPQPPPSPGLPLSAGGDPQPHGVTSSEPPTPAPTHTQAGSSQSTATPFG
Kunsagi-B       ---VPQNSAPISTAPTGGGGETISA---PPPS---VAVPAVDTTVTTPTASSQSLGAATG
Kunsagi-C       ----PDPSAPAPVETTPSSMDTTPD---PVQN----TGQAQPATITTNAGSSNAQNPSSG
                    *: ***    .. .   . .    *       :  .  :.  * :.**::  .. *

Kunsagi-A       GASSGHTPTFEDACFANCLAPDAQASLTGKLTRVSTFGYSNSARSFASINVVHLPRNYYV
Kunsagi-B       GANAPQEVNFEDQSFANTLPPDLQADLTRKLTRVSTTTYTANSKPFEDLVTIHLPRDYYV
Kunsagi-C       SSTVAQPLTFNDLAFANTMSPNLQADLTHKLTRLAYFTYQSSG-MFTDILVLDLPSALYV
                .:.  :  .*:* .*** :.*: **.** ****::   *  ..  * .: .:.**   **

Kunsagi-A       TYTQAPPHGSALFMRLVRTRFLVEIQSSAPLGAAGIVVFYFAPADIPES-EGHMGSSVFN
Kunsagi-B       SYPQAPPFATAMFLRYVKTKFIAEVQVNAPMGCAGIVVAYYLPPFVNEQ-YDHT--TVFN
Kunsagi-C       NSSYAPPSATALFLRNVRTKFVFEIQVNAPMGAAGIMVVYFLPRTVSDSGYDHS--TVFN
                . . *** .:*:*:* *:*:*: *:* .**:*.***:* *: *  : :.  .*   :***

Kunsagi-A       LSPCDFDVARMSTAKFVIPYAATTPFVPVDSHDMGRFVCMALTPYRAPSGAQSELTFTIY
Kunsagi-B       SPHVIIDVARQSTARFIIPYASTTPFVPVDSTDMGSIRLMPLTPFRSPTGTSATVTFVTY
Kunsagi-C       SPHVIVDVARTSTARLVVPYAATTPYVPLSSNQMGSLRIMVLTPYRPPPNTSSTLTGVVY
                 .   .**** ***::::***:***:**:.* :** :  * ***:*.*..:.: :* . *

Kunsagi-A       MAAIESELTCPRPRRQ-------MFSTHSQLQEVVITSTPGSLTIGNSSLANLAPTLSLA
Kunsagi-B       LAAVESVLSCPRPRRQ-------IFSTIASNPEYVIPITPGSLNIANSQVSSHAQSLSLA
Kunsagi-C       CAALESELALPMPRRQAPPPGVTWFSAHSQLQEMQIVNTPGSINMGNSRTANNAPTLSIA
                 **:** *: * ****        **: :.  *  *  ****:.:.**  :. * :**:*

Kunsagi-A       GEGYRIDEITPGGQRPVDDFIWLALRPSTRYEGWSFDWHPTDPAEAQLFAWDLDLGMSTS
Kunsagi-B       SEGFRIDHTTPGGQHPVRDFKWLAMRPGSRTDSWWFEWPATTVAESRIFAYNIDIGRSVG
Kunsagi-C       GEGFKIDSMTPGGARPFSDFAWVAMRPGTREKDFQLTWTPSQVAGTQLMAYNLDLGQSTA
                .**::**  **** :*. ** *:*:**.:* ..: : * .:  * ::::*:::*:* *..

Kunsagi-A       NLGILANSFAYARGSLQITLVAALSVFNRGRLRLCFEMAGDDRYDHQASMAVNFTVIDFS
Kunsagi-B       TLGMIANSFAYARGALNITVMVASSVFNQGRLRIAAEPSGDDNFDNQSSMAVLFTILDIS
Kunsagi-C       PLGIMANSFAYARGSLVVTLLCATSVFHKGRLRVCFEMDGTDEYNDQTSMGVFYTVMDFS
                 **::*********:* :*:: * ***::****:. *  * *.::.*:**.* :*::*:*

Kunsagi-A       NSNTGSLVVPYTSPSWFRPTTADHWGRIVVFVNTPLTASAACIDVVSVQVFVSFAPDCKF
Kunsagi-B       QSNTATLTVPYTSQSWLRRLTGSFWVRLSIFVNNPLTFNASSIDSVRVGIFLSFGNDVEV
Kunsagi-C       STNTGSLVVPYVSNTWYKPTSGSYWGRLKIFVNNPLSRNAAAFESVTVQVFLSFGADVKF
                .:**.:*.***.* :* :  :...* *: :***.**: .*:.:: * * :*:**. * :.

Kunsagi-A       YVPHSNGMTYQAPPMPDQVSATQCSDPFGPQVTVVDFSQPLVAAETSPSPHAMFSSAHTN
Kunsagi-B       YVPHSNDMLYHAPPVP-----SQVADPFGPQMVTTDLTQGVSTPQPITPEHPMFSVSHSS
Kunsagi-C       YVPRSNNMLYQAPPTS--FSQVQVTDPFAPNVTAIDFSRGLLGPDGSGEPHPMFTESHSR
                ***:**.* *:*** .      * :***.*::.. *::: :  .:     *.**: :*:

Kunsagi-A       VRHLLGRFWHLTDEFEATHVLSSHPIPFPANSHAAIARSCAFWNGEPVFVVINHTAGPIE
Kunsagi-B       VDRLLGRFWQVTDVLMAQHTIELFPVPFPANTHAAIARSMAFWNGEPVFAVVNPNPFPIE
Kunsagi-C       VENLLGRFWHVSDQVPASGTPTHFPVPFPANTHAALARTAAFFNGEPVFCVVNTTSHTLE
                * .******:::* . *  .   .*:*****:***:**: **:****** *:* .. .:*

Kunsagi-A       VSHFWEDAYVA-VTHALSSRGSILIPSKGQAVFSVPFYSPTPVRSTDANASLGNLLVRTL
Kunsagi-B       VTHFWDETPVM-VPSAMASRGSIIVPAQGNTMFSVPFYSATPVRSTDSNATLGSIVARTM
Kunsagi-C       VAHYWDDSILTGVTHAAASHGAILIPPNGHSVFSVPFYSQTPVRSTDSDSSLGILVLRAL
                *:*:*::: :  *. * :*:*:*::*.:*:::******* *******::::** :: *::
```

```
Kunsagi-A       ETAVSGRFSVFVAIRNPKLFFPRAIPDWVIPGRSVAR--TYRSALAALTGAASARATTSR
Kunsagi-B       NADHRGQVTLYAALRSANFFFPRPVPDWVIPGSSASLS-SYRATLGMLTARGLNAESAPR
Kunsagi-C       GSSTTEHVTLYAAIRSPNLFFPRPIPDWVLPTDLVNGSPSYRTALARLTLMRSSDPTLAP
                 :    :.:::.*:*..::****.:****:*   .    :**::*. **       : .

Kunsagi-A       ESVARIMAAAAALQGPGCFPFGRHSAP---------------------------------
Kunsagi-B       TAISRVMAAAASAAAQ--------------------------------------------
Kunsagi-C       AVTSQIIAAASAQGWQRDLTQDGDVESNPGPFSLLIQLLEVYKPFVVGLVGGSILLGIVI
                   ::::***::

Kunsagi-A       SLLHFLIGRPRPRVPPSP-----------------------------------------V
Kunsagi-B       -----------------------------------------------------------G
Kunsagi-C       SDSVWQRDLPREGVEENPGPVSYSFIFFKMLKSPLLTGLFPPLLCAQALIALQRWLAVRS
                         *.      ..                **:*

Kunsagi-A       SHSSVASSPRSLLLSGDVEPNPGPVTMVYDPIECKYYLEVGGHLLGMNPPRGWPAKTKIN
Kunsagi-B       GPNCDPDLSSLLLLSGDVERNPGPCVIVHDPLNNEYMLEVGGMLLAITWPRDVEKLKLKA
Kunsagi-C       YDPLAPSQWCRDLTCEGIEPNPGPVRVIKNPLTEEYYLESQGVILKLVFS---------A
                                .:* ****  :: :*:  :* **  * :* :  .

Kunsagi-A       KKVNTTCRYPKTHKCPRWSLFIAQRQPWFISGPIVWDWIMVDYFPLSPEAILCSHGICQI
Kunsagi-B       ARGKSLCKWPRAERCPRFSLRSPPLREWHIYGPTIYDWILADYFPCKPELTLCVHGLTTI
Kunsagi-C       RKGKSKCSGTPCCKATRYKFEVVKPAPYLLAGPVIWDWTIVEYFSSCCKHDLCVHALIDA
                 : :: *  .   :..*:.:       : : ** ::** :.:**.   :  ** *.:

Kunsagi-A       ENDDVHLLMDGVFLPLSRLARMDCAGLISWLFLKMPTWFHCFPFSRPSCPVYLPP-----
Kunsagi-B       EDPKVHVILDGFILPVSRLARVDTIMMATWALAMAPRWFAGLPFSVPRAPPLLPPXLRQT
Kunsagi-C       TSPEVNFFLDNWALPVSKLSRTSVFAIVGWMFKEAPNFFRSPVFCQPTAPQAEYPD----
                 . .*:.::*.  **:*:*:* .   :  * :   * :*    *. * .*    *

Kunsagi-A       -----EARAGVTMTAIPQGPFDTILSFFDGYLMKSAFRHYTPVVLRCIITLYSIHVANDP
Kunsagi-B       IAQRMADIAGSASSAIPQGPFDWISAWFDTYALKATLRNYAPVLIKAAIHLYAIHVAKDP
Kunsagi-C       -----QKYWPDTSGALPQGPFS-VFSLFDSYVLKSTLKAYAPFLIQSVINLYVMSVANNP
                           :  *:*****. : : ** * :*:::: *:*.:::. * ** : **::*

Kunsagi-A       IVTLLLGGLATYDIMVTKPPAALVVIIDAVSCATYDSFKAVMAPVFGDITATAWRRIKEK
Kunsagi-B       VVTLLLGGMVMYDMSTHKAPPAIAVLIEALACGTWDAFKAATTPLFLELPSVAWNRIHRA
Kunsagi-C       VVTLLLGGVTMYNAVTLKPPPLLVTLIEAFSCGTYDAVCAAAAPLLEGAPLRLFNTMKNK
                :*******:. *:  . *.*. :..:*:*.:*.*:*:. *. :*::   .   :. ::.

Kunsagi-A       FLRLCARAQDFKKTTDTAKAVVWWISSIAKMMHYVWINWLNPPVQNRQVQLAVAQLLVDC
Kunsagi-B       FTRLCARAQDFKKFTDIARSVAWWISAIGRLLQYVWFNWINPPTQDPDVQLAIADVLINA
Kunsagi-C       IKGIFARPQNFASITTAAKHFVWWIKTVITFFQWIWVNLVNPPAQNEQIQMTVSDLVYQA
                :  : **.*:* . *  *: ..***.::  :::::*.* :***.*: ::*::::::: :.

Kunsagi-A       NAFLSDLSLDPSTLGDRPRRQR-LLVYLQRALQMPGNPDNVQRLLQQSFNKLVSLSPIPP
Kunsagi-B       NGFLAEATTSQVKTQELAKKKKDLLVKLHYVLKTPGLPAEANRLAQNAFNKVSSVQTAPP
Kunsagi-C       NSLLIRYNTEPDHRHELDRLKERLLGKLHVASKIPGLPERAQKLVCASIEKLCRTSPTVP
                *.:*   . .     :  : :. **  *: . : ** * ..::*   :::*:   ..  *

Kunsagi-A       SPPSTRVEPVGVWLSGEPGTGKSLLMSALSADIAKHYNWTVYNHPTGSDYFDLYTNQQVH
Kunsagi-B       PRAPLRMEPVGVWIAGEPGTGKSLLMSALSVDLAKHFNWGVYSHPTGSEYFDLYTDQEIH
Kunsagi-C       EPVDKRPEPLGVWISGPPGVGKSLLMSKLAEDVMAIKKWKVYYHPVASEHYDGYTGQYVH
                     * **:***::* **.******* *: *:    :* ** **..*:::* **.* :*

Kunsagi-A       CIDDLGQGKAEEDLKVLCQCISTVPFVVPGAAIEDKKHYYNGKVVIATTNRTDFRTYTLT
Kunsagi-B       MIDDLGQNKEEKDLKILCQCISTVPFVVPIADLEGKGTHYNGKVVLATTNRLDFTTYTLT
Kunsagi-C       CFDDLGQSKNELDLACICQAISCVPFIPPRAALEDKGGYYAGKLVIATTNRIDFDTYTLT
                 :*****.* * **  :**.** ***: * * :*.*  :* **:*:***** ** *****

Kunsagi-A       TPGALERRFPIRVRVLQSKWPLTKETLRDRTYFNLVSASEAAHPITYSCLLDSIIQAYDS
Kunsagi-B       TPGALQRRFPITVEVLKSPWTLNKESFDNRSFFRIQGP--NKQPVRYDTLLQSIISMVEA
Kunsagi-C       DPGALKRRFPIKLSIGPPKFPFSKQTIADGSSFNISNFD-TGAPVRYETLLHTILEDLDR
                 ****:***** : :  . :.:.*::: : : *.: .      *: *. **.:*:.  :
```

```
Kunsagi-A       RLALLQGPPSPNPFHLLADPTFPPPPPEDEPQSCDDDHLAAIEALEVTPDSNDQKWLEAV
Kunsagi-B       RESFAQ---SPSPVRAVSDS--VEELLEGTSVDPDLDAVDEMSMEMDSPAHHDAWWRRVI
Kunsagi-C       RQAFAQ--------------------------------NPEEEVPCSPDAHDDLWITRL
                * :: *                                    .    :*  :*  *   :

Kunsagi-A       GGSQAADPDLPMLSSAPADPSLLERVTALADRLYGSLRKRTVPEWALMAFGSLGLFAVLA
Kunsagi-B       GGVIAADPVLPFLSDAQRD----AHTQDLAARLFAAVPRRTIPEWVSLAFGAAGAFCAIR
Kunsagi-C       EAFKVHEPLLPMLSDDEIT----PEVVRIRESIFRECPQRRAPEWAVMLFGAGGLAGLLL
                 .  . :* **:**.         ..  :   ::    :*  ***. : **: *    :

Kunsagi-A       GIVRKIFSFLQP------RPTPPQGPYNPATAGVRISARELARRADPQSPWQPQFNHCFK
Kunsagi-B       AFVSTISNWLGSSGAAKEEKAPSQGPYNPTGTAVRVSARELARKADPQGPWSAPCHHLFK
Kunsagi-C       YAIPKLSSLVSGLLPKSETLPEDQGPYNPTGSATRISARELARRASPQSPFSAPFTHLFK
                  : .: . :          .  ******: :..*:*******:*.**.*:..   * **

Kunsagi-A       NCVFIEAD-GFTWYAVMFGRVLVVNKHYLDCWSGPVVVSTAVSSFSADLTVPPQFVEGDL
Kunsagi-B       HTGFIQLESGTVYFCAISSRTVIINTHMFRDLPENFTLTTLLGEFKISKSKLQLRSDGDI
Kunsagi-C       NCAYLTCG-DRWAHALVSGRNLIINKHMSKDFSGIVKIATAYGEYSG--RLLHIRDEGDV
                :  ::    .   .. : .* :::*.*     .  . ::*  ..:.          :**:

Kunsagi-A       AYFHLPSAPPLKAAPKHYTVPEPASGTQAMLLYAGRDGTYAVTTHTNSYYTFTNGSFFCG
Kunsagi-B       TYAKCMQIPPHRTIQP---VPEIVQGSQTMLLFSTPSGNYIQTVEK--CASFQNVKFWHG
Kunsagi-C       SVFLLPPGTPIYKELKFR-IPSLPFNPPAFLLYMTSDSTFAQQVKDLKHIPISN--YWHG
                :       .*         :*.   .. ::**:   ...:   ..     .: *  :: *

Kunsagi-A       IVPSVHSYEVPTQAGMCGGLLVLQVGGNWIPYAVHFAGLPHRGYAQGLNLDWIAAVNAPA
Kunsagi-B       TQTIAYAYNTATRPGMCGGLLVCLHEGNWVPVGIHMAGTPSQGFSAGFVDPYLFES----
Kunsagi-C       AQKDSFSYSTHTRSGMCGGLLITQIDGNWVPLGIHMAGLPTTGFAASPIHALP-------
                     .:*.. *:.*******:    ***:* .:*:** *  *:: .

Kunsagi-A       HPLPPLPDDALPAPQGIITKIEPTSNFRLGFCPYTKYSPSPVSLVIRSELEPAVLSAHDN
Kunsagi-B       -----------ARAQGKIVTVKEVPRFTLGFCPKTKYMPSPVSLVVESDLAPAPLSAFDS
Kunsagi-C       -----------PLPQGIITQVREG-QLRIHRPSHTKLRPSPVAAIVESELAPAVLSAHDR
                           . .** *. :.   .: :   . **  ****: ::.*:* ** ***.*

Kunsagi-A       RLEVKRESNAMFLLEKTQKYDTNVTVPRPLLLQTLATEYGTHLRNLMSTLASPASIEEAV
Kunsagi-B       RLEVKRESNALFLLEKFKKYDRDVSCKSPELLTAVTDEYFTKLQVLFTRPARPVSIETAV
Kunsagi-C       RLDIQRVSNEEFLMEKCKKYCSDQVCNHPDLLQAVVDELEMAITRHTELVCEPVTLEEAA
                **:::* **  **:** :**  :     * ** ::. *    :       . *.::* *.

Kunsagi-A       FDTVCPMDHRASAGPHYPGVKRSELIDFQRRTISDRLREDVVALRAAFARGDNVY--LPF
Kunsagi-B       FDTVTPMDHKASAGPKYPGIKRSELIDFQHRIIHDTLRADVHTLTQDLEQGKFDG--VVF
Kunsagi-C       FDTVTPLDHTSSPGYKYAGTKRRDLIDFENKIISPRLRNDVANLELQFRGTSTGAGEVKF
                **** *:** :*.* :*.* ** :****:.: *   ** **  *   :   .     : *

Kunsagi-A       SSFLKDELRPKPKVRNGDTRVVECSSLHYTVAFRMQFLSVLRMMYGSDPNQTGLAPGMNV
Kunsagi-B       SSFFKDELRSWDKIRQGETRVVECSSLDYTVAFRMQFLEVLTVLYGSDMVETGLGPGINV
Kunsagi-C       ASFLKDELRPLSKIASGDTRVVEACSLDYTLLMRMYLLRFFQMCYQSDPTLFGMAPGMNV
                :**:*****.  *: .*:*****..**.**: :** :* .: : * **    *:.**:**

Kunsagi-A       YLEFSTMVSNLYPNNLCLDFKKYDSRLPSDVMSVAADLFASLTEDPVVSRRYFDPIIDSI
Kunsagi-B       YTQLYPAFAQLYERNLCLDFRKYDSRLSTEVMIQGARLLSNLTSDPEVSMNFFWPIINSN
Kunsagi-C       YTDMLPLCTSLFDYNYCFDYSKFDSRLPLQVMHRVAQMISNLTPEPQFVMRLFQPILIST
                * :: .  :.*:  * *:*: *:****. :**   * :::.** :* .  . * **: *

Kunsagi-A       HEVGPYRVEVHGGMPSGCAVTTLLNSVCNVLMCSYAVLLQDPDMDFQVVAYGDDNIVSTA
Kunsagi-B       HQVACYDVVVGGGMPSGCPITAVYNSVCNVIMMSYALLKCNPDVHFITFAYGDDNVVSVD
Kunsagi-C       HIVGSNEIVVEGGMPSGCPITTIMNTLCNVVMTHYCMLLLDPNSDFWPVAYGDDLILSTR
                * *.   : * *******.:*:: *::***:*  *.:*  :*: .*  .***** ::*.
```

```
Kunsagi-A       EPLDVPAFRSVLASDFGMVTTSADKSEECYQVPPEQVDFLKRRLRWTSDFPVPVPVLPLD
Kunsagi-B       QEISLEKFTSILREEFGMEPTAPDKTLNYSFVPPSEVTFLKRTLRATPEYPLPVPVLPLD
Kunsagi-C       KPIDTELYCKIMNEEFGMILTGADKTTTVQAVPPMSVDFLKRRMHYTPEFPLPVPVLPLD
                : :.   : .:: .:***  *..**:     *** .* **** :: *.::*:********

Kunsagi-A       SMLSRLCWCKGPHEF---------------------------------------------
Kunsagi-B       SMLSRICWCKGKREFIDQLRSFVTELALFGRETYTTVQAALLPAANLPPWDFAYRSAASV
Kunsagi-C       SLLSRICWCRGETEFKQQLESFSYEVALYGQSVYERIRVQLMPTVTMMSWPVAHRTVLTM
                *:***:***:*  **

Kunsagi-A       ----------------------------------------------
Kunsagi-B       LGLEDMTHSPTFSFYCPLPPSVSTDDALIALTEMTRSLHFDDGIPQ
Kunsagi-C       LGCY------------------------------------------
```

Limnipivirus Polyprotein Alignment

```
Limnipi-B1-F37/06          -----MDIMTSLTTSFASTIVDSAQDAVAGLMKDDPAPVEAPNVVISDQS
Limnipi-B2-XQTMC45688      -----MESLDALTTAFSATLLDTATDAIGGLLKDDTAPVEAPNVVISDQS
Limnipi-C-20               ----MASIIENLTTTFASSMLGTAEDAVGSLMDAAVRPVEAPNVIISDQS
Limnipi-C-FHMPV-1          ----MASIIENLTTTFASSMLGTAEDAVGSLMDAAVRPVEAPNVIISDQS
Limnipi-A                  -----MELLNDLVAPVMTKVADVAEVAVGGLLDDVAHPVETPNVILTDQS
ClownFish                  MLSTTMSVLSDLVQPVLSKVVETSEEAIGSLLGDSVAPVETPNVIISDQS
Limnipi-D-GDDSYC43605      --MEALSLVNTIETAVAG--VAEVAGKVSGLLPSEDKPIEAPNVVISDQA
                                 . :  :  ..           :..*:     *:*:***:::**:

Limnipi-B1-F37/06          AVAESQIYDVSETPPALTASTVEQEVGQMNIQRYLRLDADTWATSSTAET
Limnipi-B2-XQTMC45688      AVAESQIYDVNDTPPALSASTVAQEVGQMNIQRFLRLDAASWSTSTVAEK
Limnipi-C-20               AVAESEIYETGTVPPALTASTVEQEVGQMNIQRFVRLDAAEWSTSSAAEV
Limnipi-C-FHMPV-1          AVAESEIYETGTVPPALTASTVEQEVGQMNIQRFVRLDAAEWSTSSAAEV
Limnipi-A                  AVSESELYAAGEDPPALTASSVQQEVGQVNVQRYMRLQGGTWDSTQVAET
ClownFish                  AVSESEVYETNQVPPALSASSVEQEVGQMNIQRYVRIQSGNWPGSILAEA
Limnipi-D-GDDSYC43605      ATAESEVYSGG-AAPDITASTVEPEVGQLNLQRFVRLSTAEWPTTTEAES
                           *.:**::*  .  .* ::**:*  ****:*:**::*:.   *  :  **

Limnipi-B1-F37/06          KLKEWKLPDIFNSSKYPTYNLMKNYHWMRSDYEFNLVVNANMGFSGALVL
Limnipi-B2-XQTMC45688      LIKEWKLPDIFNSTKYPTYNLMKNYHWMRSDYEFNLIVNANMGFSGALVL
Limnipi-C-20               KLKEWKLPDVFYDSKYPTYNLMKNYHWMRSDFEFNLIVNANMGFAGALVL
Limnipi-C-FHMPV-1          KLKEWKLPDVFYDSKYPTYNLMKNYHWMRSDFEFNLIVNANMGFAGALVL
Limnipi-A                  AIAEWTLPDVFNSPRLPTTNLMRNYLWMRSDFEFNLIVSANMGFAGALVL
ClownFish                  ELARWELPDVFNQSKFPTSNLMKNYIWMRSDYEFNLIVNANMGFAGALVL
Limnipi-D-GDDSYC43605      LMLTLKLPDAFIDPKFPTYNLAKNYQWARSDFEFSVIVNTNASFAGALMM
                            :    *** * ..: ** ** :** * ***:**.::*.:* .*:***::

Limnipi-B1-F37/06          VYIPQGAHFQFKTFRNFPHVMINCSLNTSARLKIPYINLTQYCGITGDDD
Limnipi-B2-XQTMC45688      VYRPQGSYFDFKTFRNFPNVMINCSLNTSARLKIPYVNWTQYCGITGDTD
Limnipi-C-20               VYVPDGAHFQFDTFRNFPHAMINCSLNTSASLKIPYINMTQYCGIKDDED
Limnipi-C-FHMPV-1          VYVPDGAHFQFDTFRNFPHAMINCSLNTSASLKIPYINMTQYCGIKDDED
Limnipi-A                  VYVPNNAYYQFKTFRNFPHAIINCGLNTSARLKIPYINQTQYCGISGDTD
ClownFish                  VYVPNDAHFEFKTFRNFPHAIINCALNTSARLKIPYINRTQYCGIQSDPD
Limnipi-D-GDDSYC43605      VYVPEGAYFQFDSFMNFPYVIINCSLNTGARLRVPYINRKQYVKTIDDPQ
                           ** *:.::::*.:* *** .:***.***.* *::**:* .**    .* :

Limnipi-B1-F37/06          NNGTIALYVLGQLRVPASGGQEANWTLYGKLLSPEFQAFNVLESPP----
Limnipi-B2-XQTMC45688      NNGVLALYVLGQLRVPPSGGQEANWTLYGKLLSPEFQAFNILETPSN---
Limnipi-C-20               NNGTLRLYVLAQLKVPSSGGQVSNWTLYGKLVTPEFQAFSVLQGPGSN--
Limnipi-C-FHMPV-1          NNGTLRLYVLAQLKVPSSGGQVSNWTLYGKLVTPEFQAFSVLQGPGSN--
Limnipi-A                  NCGKLKLISLATLKTPSAGGQQANWTLYGRLVAPEFQAFAVLEGK-----
ClownFish                  NCGTIRLYVLASLKVPTTGGQSANWTIYGKLLAPEFQAFAVLEGNG----
Limnipi-D-GDDSYC43605      NAGEIRLYCLAKLRVPTSASTYGTYTVYGRLVGPEFQAFKVLEGNRRRRT
                           * * : *  *. *:.*.:..  ..:*:**:*: ****** :*:

Limnipi-B1-F37/06          ----GVIRVEEAAGSMFLASRHHTALRPRMGLTSEQSKSDVAVVGDSAFR
Limnipi-B2-XQTMC45688      ----GAVRVEEAAGSMFLASRHHTALRPRMGLTSEKSVSDSAVVGDSAFR
Limnipi-C-20               ---NAAVRVEQGEGAMFMASRHHTALRPRMGLTSERSSSDAQVVGDSAFR
Limnipi-C-FHMPV-1          ---NAAVRVEQGEGAMFMASRHHTALRPRMGLTSERSSSDAQVVGDSAFR
Limnipi-A                  ---DSCVRVEEAMGSMYLASRHHTALRPRMGLTSEHADPDQRVAGDSAYT
ClownFish                  ---NEAVRVEEAAGGMFLASRHHTAIRPRMGLTSEHGEPDERVVGDSAYT
Limnipi-D-GDDSYC43605      ARGGEVVRVEQGQGAMFLASRHHTALRPRMGIASEHARPDSRVAGNSSIR
                                 :***:. *.*::*******:*****::**:. .*  *.*:*:
```

```
Limnipi-B1-F37/06           SLREVTNVESFWMGLDWAYTDTIGKRLLITNIKLA-ADEATGGISVKTNL
Limnipi-B2-XQTMC45688       SLKEVTNVESLWQAVDWTYTDAIGKRLTISNIRLE-PSKDAGGIAVNTNL
Limnipi-C-20                SLKQVTNIESLWMALDWAYTDEIGKQLLVTNVIVK-SDSNVGSIKNTTNL
Limnipi-C-FHMPV-1           SLKQVTNIESLWMALDWAYTDEIGKQLLVTNVIVK-SDSNVGSIKNTTNL
Limnipi-A                   SLKEVVAIESFWQALDWTYIDEVGKRLTISNVVVD-SSELTGGISRKTNL
ClownFish                   SLKQVTSIESLWMALDWSYTDEVGKRLLITNVKLQ-SSEQVGGISNKTNL
Limnipi-D-GDDSYC43605       SLFRVVNIPSYWMGLDWDQSRAIGDVLLQTNVAVEGTGSQTGETKDTMNI
                            ** .*. : * * .:**     :*. *  :*: :  ... .*    . *:

Limnipi-B1-F37/06           GFLSNFYFGYKGDLTVTIQTISSRLNQGKLLVVFFPGEDNQDADLKIENI
Limnipi-B2-XQTMC45688       GFLSSFYYGYKGDLTVTVQTISSRLNQGKLLIVFFPGEDNANADITIDKA
Limnipi-C-20                GFLSNFYHGYKGDLCVSIQSVASRLNQGKLLIVFYPGEDNDGADLSIDNI
Limnipi-C-FHMPV-1           GFLSNFYHGYKGDLCVSIQSVASRLNQGKLLIVFYPGEDNDGADLSIDNI
Limnipi-A                   GFLSNFYLAYQGDLVVTIQAVASRLNQGKLLVVFYPGEDNKDKDVTMDKV
ClownFish                   GFLGNFYLAYQGDLVVTIQAVASRLNQGKLLVVFYPGDDNAGKDVTMEKT
Limnipi-D-GDDSYC43605       GFLSRFFYGYQGDLLVTVQAVASKLNQGKLLIAFFPGPDVKDHEMTIDKV
                            ***. *: .*:*** *::*:::*:*******:.*:** *  . ::.:::

Limnipi-B1-F37/06           NNGFTQVLDLGGKTTVRFTLPYVCQQTYRPINGVFGRFAVFVLNPLTYTP
Limnipi-B2-XQTMC45688       NNGFTQVLDLGGKTTVRFTLPYVCQQTYRPVTGIFGRFAVFVLNPLTYTP
Limnipi-C-20                NNAFTQILDLGGKTTVRFTLPYVCQQTYRPMHGVFGRFAVFVLNPLTYTP
Limnipi-C-FHMPV-1           NNAFTQILDLGGKTTVRFTLPYVCQQTYRPMHGVFGRFAVFVLNPLTYTP
Limnipi-A                   NNAFTQILDLGSKTTVRFTLPFVNQQPYRPMSNVHGRFAVFVLNPLTYTP
ClownFish                   NNSFTQILDLGGKTTVRFTLPYVCQQTYRPVSGTHGRFAVFVLNPLTYVS
Limnipi-D-GDDSYC43605       NNAFTSVLDLGDRTTVQFTLPYVSQTTYRDTAEIHGRFAVFVLNRLTYTA
                            **.**.:****.:***:****:* * .**     .********* ***..

Limnipi-B1-F37/06           ACVSTVRVLFYIGGSESFSFVYPRHGAVGFEA---------GSDD-----
Limnipi-B2-XQTMC45688       ACVSTVRVLFYIGGSESFSFVYPRHGRSKFEG---------DDDVT----
Limnipi-C-20                ACPSAVRVLFYIGTSSSFSFVYPKEGTAKFQG---------DDDDVIVEE
Limnipi-C-FHMPV-1           ACPSAVRVIFYIGTSSSFSFVYPKEGTAKFQG---------DDDDVIIEE
Limnipi-A                   ACPSAVRILFYIGAGSSFNFVYPKQSAVKFQAP--------ERNQVGDAT
ClownFish                   SSPSSVRILFYIGTGSSFNYVYPKQSAVKFQGP--------GQQGEMEKA
Limnipi-D-GDDSYC43605       AAPQTVRMLFYLSAGSSFRFVYPKAPMKVFEGLSDDEWDTMSESEEVDQG
                            :. .:**::**:. ..** :***:     *:.           .

Limnipi-B1-F37/06           -EVEPPVTNLEDEASTAHRAKRPVFRNVRVRIPDQIKSDHMLLSNLFGRA
Limnipi-B2-XQTMC45688       -EIEPPVTNLEDESSTAHRAKRPVFRNVRVKIPDQIKADHMMLSNLFGRA
Limnipi-C-20                EYIEEPVTNIEDEASTSTRIKRPVFRNVRVSLPDQIKSDHMLLANLFGRA
Limnipi-C-FHMPV-1           EYIEEPVTNIEDEASTSTRIKRPVFRNVRVSLPDQIKSDHMLLANLFGRA
Limnipi-A                   SADGAPITNIEDETSNTRVQKRPVFRNVKATLPRQIKSNHMLLSNLVGRA
ClownFish                   QQGSSPVTNIEDETTNTRVVKRPVFRNVKVSLPRQIRSDHMLLDNLKGRA
Limnipi-D-GDDSYC43605       TEVDGATTNLEDLGTDIARMRRPVFRNVRVQIPNQVKADHMDLKLLKGRA
                                 . **:**  :     :*******:. :* *::::** *  * ***

Limnipi-B1-F37/06           HALKPMTA-TVKDNTYKLIQTEKSFLSVFKLFRYRSGDMTLHVVHTSDGP
Limnipi-B2-XQTMC45688       HALKPHTA-TTTDTTYKLVQTEKSFLSVFKLFRYRSGDMTLHIVHTSDGP
Limnipi-C-20                HALKPTTS-TTADKTAQLVPTAKSFLNVFKLFRYRSGDMTVHIMHTGTGP
Limnipi-C-FHMPV-1           HALKPTTS-TTADKTAQLVPTAKSFLNVFKLFRYRSGDMTVHIMHTATGP
Limnipi-A                   FALEPYTSTSTGGQNILLKENNLSFLKVFKLLLYRSGDMTLHVSHTAVSP
ClownFish                   FPLQAWTSGTTGAETVTLTENDLSFFKVFKLFMYRSGDMTIHLSHTAEAP
Limnipi-D-GDDSYC43605       FPVLNTTCKNKFAR-SELMALSESFGVLYRIFRYRKGDMTLHISSTSTSH
                            ..:   *. .       *     **  ::::: **.****:*:  *. .
```

```
Limnipi-B1-F37/06          LFAAHSYLAPDLG-TNAEDNYSEVLSQGAVCMLPKTAASICVPFYVLTPF
Limnipi-B2-XQTMC45688      VFAAHSYLADDLG-ADAEDNYSEILSQGAVCMLPKTAASICVPFYVLTPF
Limnipi-C-20               LVVAHSYISDDLG-TVAEDNYSRVLSQGSVAMRPASNASLCVPFYVLTPF
Limnipi-C-FHMPV-1          LVVAHSYISDDLG-TVAEDNYSRILSQGSVAMKPASNASLCVPFYVLTPF
Limnipi-A                  LYMAHSYIQTAIAGTTSADRLSQIMSQGSVCVMSNSAASLCVPFYVMTPF
ClownFish                  VYMAHSYIQTTMTGTSENDRLAQILSQGAVCVMPTSALSMCVPYYVMTPF
Limnipi-D-GDDSYC43605      IYVAHSYFLNSTP-TTESEQLSLILGMGAVAVPPNGMASMCVPYYVFSPF
                           :  ****:      :   :. : ::. *:*.: .    *:***:**::**

Limnipi-B1-F37/06          VSTSDYGKILLSSPDDSSYTVRSSVTFDSNTRFYFLQSPP-------ASK
Limnipi-B2-XQTMC45688      VATTELGKLLVSSHNDATYNLRASVTFDSNTRFYFLQSPP-------AVK
Limnipi-C-20               VESMELGKLLMSSLDDTTYSVRLALTFDRNTRFYFLQSPPD------VVA
Limnipi-C-FHMPV-1          VESMQLGKLLMSSLDDTTYSVRLALTFDRNTRFYFLQSPPD------VVA
Limnipi-A                  VKTADFGRLFVEGQNDSTWIMWIGATFDTDTRFYFLQSPPDIALGENEKE
ClownFish                  IKVDDFGTLFVQSQNDTTYKIWPSLTFDSDTKFYFLQSPP--SVESATVE
Limnipi-D-GDDSYC43605      IGADFLGNVFSLSVDDSTYALYVSVTFDADTEFFFLQSPPK------AKP
                           :     * ::  . :*::: :  . *** :*.*:******

Limnipi-B1-F37/06          TDRDD-DFDDDADFS-------FRAARSPMD--------VVDDYPF----
Limnipi-B2-XQTMC45688      STAPPGLYLDDEDFCSIELLTPARALARAME--------RRSGLNFPEFQ
Limnipi-C-20               ADFFGNDVDELEGPSDWSRLNLLERTLDRLA--------VETTYPVKPVS
Limnipi-C-FHMPV-1          ADFSDDDVDDFEGPSDWSRLDLLERTLDRLA--------IETTYPVKPVS
Limnipi-A                  EPEDLEWV-EEEGHR----QTEVETIQRFLEGRGLFPVHKTCDINDRDGM
ClownFish                  EDTDLEWFPEEEGVRGSVWQMEISTIQRYLTNTEWDHSYRSGEIRGEDQE
Limnipi-D-GDDSYC43605      SRSARSIDSDDEEEVDWGVGRMRLKMSGDVE------------ENPGPN
                                    :                   :

Limnipi-B1-F37/06          ---------------------------------------KR-----DLT
Limnipi-B2-XQTMC45688      ITTVGNRRQYLCWLDGVVYKSF--CPDHLRSKIIRKMTRNAR-----DLL
Limnipi-C-20               YAGRFLHFHSKAEVDGVPFYAQGKTKRECRLKLLEQILAYKR-----DLT
Limnipi-C-FHMPV-1          YAGRFLHFHSKAEVDGVPFYAQGKTKRECRLKLLEQILAYKR-----DLT
Limnipi-A                  YMTCYSEVTIG----DKTFRANSFWRTKSLLKAYRMCKEFVRESDNQELL
ClownFish                  FIMWFGELTLRKGDVEKKFRANSYYNSKTQLNTYRQAKTYVR-----SLV
Limnipi-D-GDDSYC43605      ATVQRSQRQMIEELLATEGEKIALQIMCVIIVAVHLLVTWMR----RRLT
                                                                    *      *

Limnipi-B1-F37/06          RDGDVESNPGPWFVLRSDGGLAIARN--GRVMVPVYDREGGFEWAIQPQ-
Limnipi-B2-XQTMC45688      VCGDVESNPGPIMMMGLNGILTMRRSDGGGVWTLTHSRRG-VEWAVSPS-
Limnipi-C-20               ACGDVESNPGPVLAISSTFGWSLIDEG-VPITIPALNLAN-AAVVSSPL-
Limnipi-C-FHMPV-1          ACGDVESNPGPVLAISSTFGWSLIDEG-VPITIPALNLAN-AAVVSSPL-
Limnipi-A                  KCGDVESNPGPDIWRNRITGCMAVQEGAALYYSPNKQIVDNIYVLPVQN-
ClownFish                  ADGDVESNPGPAIFRHQLSHALGVKERGWIYHCPNKNFQDGRRICVMQER
Limnipi-D-GDDSYC43605      LSGDIESNPGPTFEEELMTLVNVAKAP-----------------------
                             **:****** :

Limnipi-B1-F37/06          -----RLFWRVIYVRDSFLDSYSSAIPALHHLVQDSTMESDEEYKNAILS
Limnipi-B2-XQTMC45688      -----SVFIPVCYFTNQFMAGMVQSLPLSEDLAIDASMGSHSHEQRVALN
Limnipi-C-20               -----DSWWQICEIEGDFLRNFNTALSANFAPDIAIADDVPQTYTNTAIF
Limnipi-C-FHMPV-1          -----DSWWQICEIEGDFLRNFNTALSANFAPDIAIADDVPQTYTNTAIF
Limnipi-A                  ---HIEDWERYSHFTMACLEPIWT-AQMPRLQHTNTIAVNNPAPNTVAAA
ClownFish                  RPEEIQLWKFYCNTRGEDISLIWNRLNMDALEDLLELQIPFGSPDTNRTC
Limnipi-D-GDDSYC43605      -----------TYNYIGFVNEYKQKQSSFSLPALEVLREGTPNEPIWITW
                                             :
```

```
Limnipi-B1-F37/06          VTIVSMRPGWKRLRTFSLISLIGFFSAIDL-VQAAYSRMRLLLSGDVEQN
Limnipi-B2-XQTMC45688      LMVATSGP-RRWLCTLVIVPVVGIASISTI-LLSFISRALLLLAGDVERN
Limnipi-C-20               LQIMSRGFVRRIVITP-FISMFMLAGFSRW-IHARFARLRLLLSGDVEQN
Limnipi-C-FHMPV-1          LQIMSRGLVRRIVVTP-FISMFMLAGFSRW-IRARFARLRLLLSGDVEQN
Limnipi-A                  ITLMAMRVNGPRWLVKFIFPFLHAANMWDL-STGWFHFFRLLRSGDVEQN
ClownFish                  LVMTHLKNTIPAWLFRPMLIFLRFCTYWDR-STNYMNFMRLCLCGDVEEN
Limnipi-D-GDDSYC43605      YPGKTARVGHRLIKMSKQKSALALMVETDLRAVKSFIESQLMRAGDVERN
                                                .                  *  .****.*

Limnipi-B1-F37/06          PGPRSQ--------VFLGPDRTIYLQVADQLLF----LNDFSISEERLTV
Limnipi-B2-XQTMC45688      PGPCS----------FYFVDGMVRVSAGGFCFG----FEKFSTALDTLNF
Limnipi-C-20               PGPVFFYKE-----TDLGTHFLCPLNSANTRFLGIYNIDNFIFSKEIKIT
Limnipi-C-FHMPV-1          PGPVFFYKE-----TDLGTHFLCPLNSANNRFLGIYNIDNFIFSKEIRIT
Limnipi-A                  PGPINIR--------YCTDEEHIRYALKVHNLKSIEIVNRDFFQEIGEKT
ClownFish                  PGPTFKY--------FMTGEDRAKFSIKTKSGVPIQISASDTFSAIGSGV
Limnipi-D-GDDSYC43605      PGPSLLRKDKGVYHHFGVQVGDHVLHMSSDNVLSVLGSGEVEIVNTPYVP
                           ***

Limnipi-B1-F37/06          SSTEPLSFPHNKGFWMSSQNIRALFRTGATQMSVGFFYLDNQTNFSDAFI
Limnipi-B2-XQTMC45688      VVVP---TPTTGEFVLTSANIRKLFLIGNEQMRAGPLFVDNATLFSSRFL
Limnipi-C-20               SRWS--AVPTSNTIVITERQFTRLTILGEHALDG----HDVRFVFDDQTL
Limnipi-C-FHMPV-1          SRWS--SVPPANTIVITERQFTRLTVLGEHALDG----HDVRFVFDDQTL
Limnipi-A                  IHLQIVDTKHWRNLTLTDSEEKTLMKIGKDLITND----MKLTTPVGLGR
ClownFish                  INVDRASPESWRSMRFSNAVSKRLTLHGGELMQG-----CTITAPVG---
Limnipi-D-GDDSYC43605      SEWSLVCSIDEDEDKLMSRAYRYARDMGHFSVRNN--CQTFVNYLIGEDV
                                          : .         *   :              .

Limnipi-B1-F37/06          ES-ILPMMYALNTCALM---DAEIGFETMMQWLLGINPDSVANARAMSAV
Limnipi-B2-XQTMC45688      ES-VVPMLYALQTCAID---DAELPFENVIQWILGIENEALENAKLAGKV
Limnipi-C-20               EDTPLPCLYALVAFCLARVVDAESPISGVVQWMLGVDASAIANAQLASTI
Limnipi-C-FHMPV-1          EDTPLPCLYALVAFCMARVVDAESPISGVVQWMLGVDASAIANAQLASTL
Limnipi-A                  VEHLIEKTHLLTGLRFLKIVNPEGRISDTLAWFLGWEKSGLERVHVLTTM
ClownFish                  VDVTEEEVALLIGLSFVHIVYPEGIFTDTFCWLFGMKKSGVERMQIATEL
Limnipi-D-GDDSYC43605      ALQSKGLMFVAYVLLGLSVMGVMVPEGSLLERFTGVSEDNMTRLNGMMNV
                                                        .  : * . . : . .    :

Limnipi-B1-F37/06          LEDSNVRRMLKDAGEGIAASLLRGLLRVLLYCMILSRDCSATTVMAITGL
Limnipi-B2-XQTMC45688      LSDANIRRMLSDIGGGAAAVLVKGFLRVLLYCLILSADTSAMTILSVTGL
Limnipi-C-20               FTDDNLRRVLGDAWSEASSLLLKGFFKVLMFAFILSQSSSSLTLLSVSCL
Limnipi-C-FHMPV-1          FTDDNLRRVLGDAWSEASSLLLKGFFKVLMFAFILSQSSSSLTLLSVSCL
Limnipi-A                  MADGNITRIVDGITDSIARTCVKGMIRAVLYTLIFSLTPNAVARGAIAAL
ClownFish                  LADGNISKIVDGISGDLMKACMKGLIRVVIYTLILSISDNAVVQASVAAL
Limnipi-D-GDDSYC43605      LNPQTLSSLVADIKNKVLSSFLKGMIRLVCYSMIVAHNPTTATILSVTIL
                           :   .:  :: .         ::*::: : : :*.:   .: .  ::: *

Limnipi-B1-F37/06          VAIDAMSVLWTAFQHSNIVSLVNFLLPGDSQIAINHRRRMVELLDSD---
Limnipi-B2-XQTMC45688      VAIDSIGALWTAFQTANIQGLVDFLLPGECPQLCAERRRLIAILTKEG--
Limnipi-C-20               VAADVSSAVFQIFQSCNVSSLINYLLPGEDVIGCCERARMIELAHDIF--
Limnipi-C-FHMPV-1          VAADVSSAVFQIFQSCNVSSLINYLLPGEDVIGCCERARMVGLAHDIF--
Limnipi-A                  AAFDLTTSILDAFRNMSLKRLAEYLIPEMENKQEYTRERTNLLAKVNKVM
ClownFish                  AAMDVGSTLLSAFQNMSLRRLLEYLIPENREDGDYTRERTNLLAQVT---
Limnipi-D-GDDSYC43605      AGMDVMGCSGFFDDVAGAIHKIVGDSAKEEEISEEDQELVQSQLQKKTN-
                           .. *            .         .         :
```

```
Limnipi-B1-F37/06          --------------------APMFTEGLFDDVTKVARGLEWWMKQIFKLA
Limnipi-B2-XQTMC45688      -------------------HDEMQAEGLT-DITFVMKNLEWWMTKLVKIS
Limnipi-C-20               -------------------KDFSDTAEDFTSMTQLARNLDWWVQRLLKLA
Limnipi-C-FHMPV-1          -------------------KDFTDTAEDFTSMTQLARNLDWWVQRLLKLA
Limnipi-A                  RHNEMKQDEWFDAVDDTQEVVTEQGQLGFNTFTLVSKNLGWWITKIQELA
ClownFish                  --------ERTNLIDN--NTPTQQGQKEFNTFTTVAKNLTWWIQKIKELA
Limnipi-D-GDDSYC43605      ---------PFLSKLPFMRGKKTEGVKDFNIFSQAAKNFEWWIEKVMVLI
                                                          .:   :.: **: ::  :

Limnipi-B1-F37/06          EMLQKQLNPDRVVRCNTLLDTKLDEIINLVAAVSDLKQRCRKEGKITNQE
Limnipi-B2-XQTMC45688      EYVQKMYNPDQAEMAGGILKMRHDEIVTTIASVSDLLQSTRNAGKVTNQD
Limnipi-C-20               SLISEMYNPDEIQRSSKILKKDEAVVVQTIAGAADLYQDIRTSGSVTNEH
Limnipi-C-FHMPV-1          SLISEMYNPDEIQRSSKILKKDETVVVQTIAGAADLYQDIRTSGSVTNEH
Limnipi-A                  DKLQQWYNPDRVQVAKTTLEEVKEKIIDALTETEALMAKVKVQGVPTQAD
ClownFish                  DFLSGLYNPDRVARAVKELAERSDTLTSDMARVLEMQSLAKVKGVLPIEN
Limnipi-D-GDDSYC43605      RKIADWINPSKYSAAVNYLKYTQDDILADICSVNEVIADSKMDGALTEKL
                             :    **..   .   *      :   :  .  :    :  *  .

Limnipi-B1-F37/06          RDEHDDILAKLVGYQKVALVVPRHPLQLPISQALSQLRSLNYGHRIPDDP
Limnipi-B2-XQTMC45688      RDTHDDLREKLVDFHKLSLLVPRHPMQLPLSQALSTLRRLNYGHRIPSEP
Limnipi-C-20               RNRYDELHEQLVMYQKLANLVPRHPFQIPINQALMTMKSVRYGHRAPEKP
Limnipi-C-FHMPV-1          RNRYDELHEQLVMYQKLANMVPRHPFQIPINQALMTMKSVRYGHRAPEKP
Limnipi-A                  RDEWQQKFDTLTKFYKLSLVVPNCPVSRNIGIVLNNMKRMQLGHQKPKDP
ClownFish                  LDEWKQLSTELGNFHKLSLYTTNTQLSRTIGDSITQLNRIRLPQE-PTEQ
Limnipi-D-GDDSYC43605      RVNWKNLHDRLSTHLSNAVSAIXDRRVQLIQTALSALNRVTLGQKQPEDA
                               .:    *  . . :  .        :   :  :. :   :. * .

Limnipi-B1-F37/06          IRPDPIGIHFYGDPGMGKTVLMTRICNVIAKSLDTEVFTHAPGSDFLDGY
Limnipi-B2-XQTMC45688      IRPDPIGIHFYGEPGAGKTVLMVRITKLIAKALDTEVFVHAPGSEFLDGY
Limnipi-C-20               IRPDPVGIHFCGIPGMGKTVLMTRICNFLAKALKTEVYVHAPGAEFLDGY
Limnipi-C-FHMPV-1          IRPDPVGIHFCGIPGMGKTVLMTRICNYLAKALKTEVYVHAPGAEFLDGY
Limnipi-A                  IRVDPVAIHFCGEPGQGKTVLMTRLTNALAKALNTEVYTHATGSNYFDGY
ClownFish                  IRCDPVAVHFVGKPGQGKTVLMTRLTNLLANQLNTTVYTHATGSRYFDGY
Limnipi-D-GDDSYC43605      FRQEPVAIHFFGEPGCGKTLLINELGGRVGKILDSEVYHHSSGSEYFDNY
                           :* :*:.:** * ** ***:*: .:   :.: *.: *: *:.*: ::*.*

Limnipi-B1-F37/06          SGQQVHYIDEFMAHTQERESMLILQLMGCSQTIVPMAELEDKGMYYKAKV
Limnipi-B2-XQTMC45688      SGQEVHYIDEFMAHTDEKESNLILQLMGCAHSIVPLAGVEDKGMYYKGKV
Limnipi-C-20               SGQKVHYIDEFLAHVDEKEANLILQLMGCSNTIVPMADLDDKGKYYQAEV
Limnipi-C-FHMPV-1          SGQKVHYIDEFLAHVDEKEANLILQLMGCSNTIVPMADLDDKGKYYQAEV
Limnipi-A                  CGQGVHYIDEFMANTDEQEASLALQLMGSSQVILPMADVDQKGMYYKGKV
ClownFish                  TDQGVHYIDEFLASTEEAEADMILQLLGCSQVILPMADLAEKGKFYKGEV
Limnipi-D-GDDSYC43605      FGQKVHVLDEFLMTREENDANLVLQLISSTHVPLPIAACDWKGGYYKGEI
                            .* ** :***:   :* :: : ***:..::  :*:*    ** :*:.::

Limnipi-B1-F37/06          VVTTANTPCVSNSGLKFPKALLRRFQQIKFAAQPYYLKTKNGEQTFDIKK
Limnipi-B2-XQTMC45688      VITTANTPCMSNSKLKFPKAVLRRFTQVNFRAKNFYQKKQNGESVFNIAQ
Limnipi-C-20               VITTSNTLPSSHSKLKFPQAVARRFKVLEFRVNSLYTTVVNGQTVLDGEK
Limnipi-C-FHMPV-1          VITTSNTLPSSHSKLKFPQAVARRFKVLEFKVNSLYTTVVNGQTVLDGEK
Limnipi-A                  LITTSNTSPVSDSKLKAPKALKRRFMVVGFRAATYYQKITNGAMCIDLAK
ClownFish                  MITTSNTNPVSNGKLKSENALKRRFRVVDFRANQKYLCVKNGEVVFNVEY
Limnipi-D-GDDSYC43605      LITASNSRPESETKLVCRQALSRRFIEVEVRPVADYVTTQNGQIVFDARK
                           ::*::*:   *.  *   :*: ***  : .     *    **   ::
```

```
Limnipi-B1-F37/06          AIADKTFEDGSCWNYSLDD----GTTWKTLNFLELAEFVRHKVAVNDKIA
Limnipi-B2-XQTMC45688      AIKDGTFKDGSCWEYSLDN----GVCWKTFDFKEFVQYVLAKVKNNQRIC
Limnipi-C-20               AMRDGSFQSGECWSFCVD-----GLNWKTFSFKKFVAEVMDMIDKNRKFY
Limnipi-C-FHMPV-1          AMRDGSFQSGECWSFCVD-----GLNWKTFSFKKFVAEVMDMIDKNRKFY
Limnipi-A                  ATKTGALQDGSCWECEID-----G-KWRTFSFYDFVDMILAKVKSNQLVH
ClownFish                  AISDGSFTDGNCWEIQEN-----GGKWVTFSFKAFASSILKSVYRNRQVH
Limnipi-D-GDDSYC43605      ATKTKAAETGECWEIKIGSAADTSAKWERLDMDTFEKFVMAKHAEKTEVC
                           *    :   *.**.   .     .  *  :.:  :   :      :  .

Limnipi-B1-F37/06          KDFIDVMKRD------CERLQAEAGED-------EEIDDIIQSLEKPKPP
Limnipi-B2-XQTMC45688      DDFMQTLKDD------EVLLAEGGGDDSEEKPDTEEAQALIDKMTTNEPQ
Limnipi-C-20               ENFSKILDDD------VDELQAPPDTP-------VDSQAILDTL---EIE
Limnipi-C-FHMPV-1          ENFSKILDDD------VDELQAPPETP-------VDSQTILDTL---EIE
Limnipi-A                  QALLEQMKQDEEQMKKYAVLAQGSEDEEKQMTD-DEFDQLLSEMRDLTKE
ClownFish                  KAFLKTLEKD------VATFQGGPGGEE------EDLENIITNL-ELTSE
Limnipi-D-GDDSYC43605      RALNASARRH----------KKVEGEP-----------------------
                             :      .

Limnipi-B1-F37/06          G--------IFDKLRHPIQASKDACKAVKTWVSTMMDGWTRESVVEVSSR
Limnipi-B2-XQTMC45688      G--------LFTPLQRVMTASKKSKELVSDWLRRNSEGWTLESAMGVVLM
Limnipi-C-20               G--------LFAFPRKLVAATRASVQYAKNWITEQCKGWTLDKTIAWVSR
Limnipi-C-FHMPV-1          G--------LFAFPRKLVAATKTSVQYAKNWITEQCKGWTLDKTIAWVSR
Limnipi-A                  MTQMSSDLEIFHEARETAVVPPDIRSKVVKAMQDCWANIVSTDIKTLLER
ClownFish                  KERNSP----FAPIRLVQEASVKTYNQVDRYISQFFSNFKEETFHRFVTR
Limnipi-D-GDDSYC43605      -----------LPMDGELLQSQYMTTVRERWEKMKPTDKTLKWVFGLVSG
                                               .                .

Limnipi-B1-F37/06          VMMVVSVLRTVWGVYSFLKDLGTNKEEAPYDKAMRTDVKKVTVRRVRQVL
Limnipi-B2-XQTMC45688      ISQIVTIARVVWSVYSFLRDIRRTNAEAPYDKATKINPKKVMVKRIQQVK
Limnipi-C-20               VGVAVNVLRIVHSTYTFIRDLTKCDVEAPYDKAIRVDPRKVLVKRAARRN
Limnipi-C-FHMPV-1          VGVAVNVLRIVHSTYTFIRDLTKCDVEAPYDKAIRVDPRKVLVKRAARRN
Limnipi-A                  VSLAVTVIRVSTTIYDMIKGFRATEEQRAYNPTTKPGLTRVLKKTARAGP
ClownFish                  VSSAMMLLKVVHQIYSFFKSTRAPEEQRAYNPSQKLRFAKVIRPRQET-Q
Limnipi-D-GDDSYC43605      IFALSTIICFAWAMITWFKRKPEEEETVEPEGPYSGVHTRPTLKQVSKHL
                           :     :          ::     .     : .      :

Limnipi-B1-F37/06          RNKTQPPPVPEHLTKESPVTQAHEFQHLIDAIVEIKFEAGYKVPLQGLAL
Limnipi-B2-XQTMC45688      KVLTQ--------EQESPVDQAHEFQHILDAMLTVTFEKEQVMPIQGMAY
Limnipi-C-20               VRVDT----------AQAPVECEQYEHFAKPMVFLKFE-DRKHNSRGFAI
Limnipi-C-FHMPV-1          VRVDT----------AQAPVECEQYEHFAKPMVFLKFE-DRKHNSRGFAI
Limnipi-A                  IQVEG-------------GNNSTEYKHILDSMVHLEFE--SGLRSTGFCV
ClownFish                  VEPQG-------------GNKSFEYRHLVEAMVDLQFG--DADPVSALCI
Limnipi-D-GDDSYC43605      KGKVKTP----------EGQVDLMPVHILKNCVIIVWG--NGIQQKGIAL
                                                     *: .  : : :         .:.

Limnipi-B1-F37/06          AGYSLLTYAHGIGSGRMTVQYGNMSLELEEDEYELAVFSTDDG----DTD
Limnipi-B2-XQTMC45688      HDYTLLTYAHGIGSGRLTIQHGNMNVELEEDQYEIMIMSTEDG----DTD
Limnipi-C-20               AKRQIITYAHGLGNGNLIVHYGGIEMNLTPDDYTISVFVVEDG----ETD
Limnipi-C-FHMPV-1          AKRQIITYAHGLGNGNLIVHYGGIEMNLTPEDYTISVFVVEDG----ETD
Limnipi-A                  GQRDIFVYAHGVGEGSMMFEHRSVAYEIPEDMYEVEQFVTGQG----KMD
ClownFish                  GGRSIMTYMHGVGDGPLTFEYKGRNFTVAEDKYHVEVFDLDGT----RMD
Limnipi-D-GDDSYC43605      GDREICTYSHGVGAGPVTLKWRGGSLVLAPQELEFENVQFEIDGKVKKTD
                               : .* **:* * : ..  .    :  :   .  .           *
```

```
Limnipi-B1-F37/06               LCWIRLHKGIGIQFKNLTNSISTPEYGMDCVLVKKIGSNFEIRPMTNVVD
Limnipi-B2-XQTMC45688           LCWIVMDKRIGFQFKNMTKIVASPVYGYDCLLLKKIGHNYEIRQMTNVSE
Limnipi-C-20                    MACIEFVSALGVEFPDFTHLISAPEYGRDARLLTAWGGSFHIRTGENLTK
Limnipi-C-FHMPV-1               MACIEFVSALGVEFPDFTHLISAPEYGRDARLLTAWGGSFHIRTGENLTK
Limnipi-A                       MARIVIDSSIGLEFKGMWKHIAKPIFSRDALLITKMDNLFREREAGTVKD
ClownFish                       LAKICIDPSLGIEFTNMWKHVATPIFHRDGILVRKDGPFFEKMDVGKVTD
Limnipi-D-GDDSYC43605           FVTFQIPKEYGVQFSNIRRHVAPIEETRDGVMFVPIDEFTIER-----KD
                                :  : :    *.:* .: . ::      *  :.   .            .

Limnipi-B1-F37/06               SDYFMMVS-DVSRLSGVRYGNFKYFGKHSRGDCGSLILQKIKGTWKMIGL
Limnipi-B2-XQTMC45688           QDHHVLQ--DRDRVIGMRFGNFKYFGKHAAGDCGSLIIQKIKGCWKVIGM
Limnipi-C-20                    GDYSMLRDPNSGKMWGMRFGHFRYLCKTQPGDCGSIIIQKQNGTWKLIGM
Limnipi-C-FHMPV-1               GDYSMLRDPNSGKMWGMRFGHFRYLCKTQPGDCGSIIIQKQNGTWKLIGM
Limnipi-A                       AGMIVMKD-FDGNIQGMRMNEISYYALTVRGDCGSLLLQKQYGTWKIIAM
ClownFish                       AGVVMLKD-ASGQLVGMRSGQISYYATTSVGYCGSLILQKQYGTWKIVAM
Limnipi-D-GDDSYC43605           VANLIVEDHQWLQSFGDMYGHVAYRAMTRSGQCGSPVFQLQRGTWKLVAM
                                    ::      .  *   ... *      * *** ::*   * **::.:

Limnipi-B1-F37/06               HNSGAAAGQRCAGVRLDIVPIQMVLEGIVVHREAAGLTSFQPGKSSLRPS
Limnipi-B2-XQTMC45688           HNSGAASGPRCAATRLDYVPVEAISEGIVVSREQSPYHTFQPGRTNLKKS
Limnipi-C-20                    HN--AAGQGSAVAFRFDLYPLD-IAQGVIVSKEKSTMRSFMPSKSKLRES
Limnipi-C-FHMPV-1               HN--AAGQGSAVAFRFDLYPLD-IAQGVIVSKEKSTMRSFMPSKSKLRES
Limnipi-A                       HN--GSRQGLAYGVRLDVCIAK--YEGLVTSKTPSDDVFFQPPKSAIHKS
ClownFish                       HN--AGRKGMAYGARLDTCAPQ--QQGVVEEKKEAPCKFFQPTKSNLKKS
Limnipi-D-GDDSYC43605           HN---ASNGRGEACGLNLSKFVLKDPEGLATLVGTHQRIHQPSQSTLKKT
                                **   .      .  ::           :     :    . * :: :: :

Limnipi-B1-F37/06               PFHGAFDVTKEPAVLSKKDRRLTVNVDNLVKDNAGKYRVDRYTANETIMS
Limnipi-B2-XQTMC45688           PFHGAFDVLKEPAVLSKKDRRLTVDIDNLVKDNAGKYRVDRYDANETIMA
Limnipi-C-20                    PFHGAFPVEKEPAVLSSRDTRLIVNIDSLVKTNGEKYRVDRFDPNTTVFA
Limnipi-C-FHMPV-1               PFHGAFPVEKEPAVLSSRDTRLIVNIDSLVKTNGEKYRVDRFDPNTTVFA
Limnipi-A                       PFYGIQEATMQPAPLRATDQRITVPIENLTKEAAEKYRVDQFDVDLNTFA
ClownFish                       PFYNREEHTLEPAPLSTRDKRIEAPIDNLTKNSSNKYRVNKFEPIMNNFH
Limnipi-D-GDDSYC43605           PIHGVFEETKQPAVLTPRDRRLEVQIEDLVKNSSQKYRVDIFDPDTPTFL
                                *::.      :** *   * *: . ::.*.*  . ****: :      :

Limnipi-B1-F37/06               FAMQKIKDRLVSHVSRGRMVSIEKAITGCGCNPIDPTTSPGFKYTKLGMK
Limnipi-B2-XQTMC45688           FAVQRVKDRLHPYISPGRRITIEQAITGLGCNPIDKNTSPGLKYTNLGLK
Limnipi-C-20                    VAAHKVKERFQNHIPLGYMISMEDAIRGGDINPIDKDTSPGYKYVSRGFR
Limnipi-C-FHMPV-1               VAAHKVKERFQNHIPLGYMITMEDAIRGGDINPIDKDTSPGYKYVSRGFR
Limnipi-A                       VAKARVLERIRPHVKVGRSIPMEEAITGAGTNPIDKNTSPGLKYTRDHLK
ClownFish                       ISKMRVKERLQQHVRYGYSIPAEVAITGEGTNPVDQSTSPGLKYTQRNLT
Limnipi-D-GDDSYC43605           IAKSNVKERLKRVVSVGHNVKINDAIRGVDSNPIDPTTSPGLKYRELNLS
                                .:  .: :*:   :  *  :  : ** * . **:*  **** **    :

Limnipi-B1-F37/06               KTDLYRINVDGSVWVSDMLRNDVQAWIDSIDAGETKQTLFNTVCKDELRS
Limnipi-B2-XQTMC45688           KEDLYKVDEHGDVWVSDRLRADVEKWIKNIDSGVCLETVFNTVCKDELRS
Limnipi-C-20                    KCDLYQILPDGTVQISDMLRKDVEVWLTAIKTGKEIDTLFTAHLKDELRS
Limnipi-C-FHMPV-1               KCDLYQILPDGTVQISDMLRKDVEAWLTAIKTGKEIDTLFTAHLKDELRS
Limnipi-A                       KSDLFTIDEKGNAVVSDRLRADVEEQEELLQSGGYPTTTFTACLKDELRA
ClownFish                       KKDLYTVNDDGTVTLAPHFAKDVEEQNQILLSGGYPTTVFTACLKDELRT
Limnipi-D-GDDSYC43605           KKDLFTISPSGDLWISERLQADVAKKWMDLTLTRSIDTTFTAHLKDELRP
                                * **: :   *   ::  :  **      :       * *.:  *****.
```

```
Limnipi-B1-F37/06          LEKVALGKTRVIEAAELDYVVAYRMYMSTIYSDLYESSAEDTGIAVGINP
Limnipi-B2-XQTMC45688      LEKIALGKTRVIEAAELDYVIAYRMIMTTIYSDIYEAAAEDIGLAVGINP
Limnipi-C-20               CEKVELGKTRVIEAAELDFVVAYRMYMSSIYSGFYNTAAHLTGIAAGINP
Limnipi-C-FHMPV-1          CEKVELGKTRVIEAAELDFVVAYRMYMSSIYSGFYNTAAHLTGIAAGINP
Limnipi-A                  DEKVALGKTRVIEAGELDYVILYRMHMNSIYRDLYNAYSGDVGVAAGINP
ClownFish                  KQKVKEGKTRVIEACEFDYVVLYRMHMNSIYSDLYKSSAVFTGIGAGINP
Limnipi-D-GDDSYC43605      VSKVAVGKTRVIEGCELDYVIVYRMVMSTIYRDLYNCPTVSCGVAVGCDP
                            .*:  *******. *:*:*: *** *.:** .:*:  :   *:..* :*

Limnipi-B1-F37/06          PADGHGLFLELNKYHTFMALDYSRFDGSLPAMLMRNAVEILASLHHDEDK
Limnipi-B2-XQTMC45688      PQDGHSLYLELNKYSTFLALDYSRFDGSLPKRLMEKAVDVLASFHVEEEV
Limnipi-C-20               PRDGHELYAELCSYSKFLALDYSRFDGSLPEMLMRKAVEILAELHESPEE
Limnipi-C-FHMPV-1          PRDGHELYAELCSYSKFLALDYSRFDGSLPEMLMRKAVEILAELHESPEE
Limnipi-A                  LAEAARLREDLSQYDSFLALDYSRFDGSLSEKLMRAAVDILADLHEDPDL
ClownFish                  LAEGNHLHEAMSQYDAFLALDYSKFDGSLSESLMRHAVDILADLHEDPGL
Limnipi-D-GDDSYC43605      LVEAHDWHSCLQSFDNIMALDYSGFDGSLSENLMRHAVDVLSSLHEDPAL
                             :.      : .:  ::***** *****.  **. **::*:.:* .

Limnipi-B1-F37/06          VKLLHETVITSKHLVADEFWTVKGGMPSGSPCTTVLNCLCNLLVLEYAFL
Limnipi-B2-XQTMC45688      AKLIHQTVITSKHQVVDEFWCVQGGMPSGSPCTTVLNCLCNLIVLEYAFL
Limnipi-C-20               VARLHETVIVSKHLVLDELWTVKGGMPSGSPCTTILNCICNLLVLEYSFL
Limnipi-C-FHMPV-1          VARLHETVIVSKHLVLDELWTVKGGMPSGSPCTTILNCICNLLVLEYSFL
Limnipi-A                  VRRLHEPVVISKHLVVDEDWIVTGGMPSGSPCTTVLNCICNLLVLDYAML
ClownFish                  VKRLHEPVIMSKHLVVDEFWSVRGGMPSGSPCTTVLNCICNLLVLDYAML
Limnipi-D-GDDSYC43605      VRALHEPVINSTHVVSDEVWFVRGGMPSGSPCTSVLNTVCNLLMLEYALE
                           .  :*:.*: *.* * ** * * **********::** :***::*:*::

Limnipi-B1-F37/06          EIFGLGERDDQFRKHVD-DHLIVVYGDDCIVAHNAEEELGPAFKETIFTS
Limnipi-B2-XQTMC45688      EVFGLEELSMKDHERVNSDYLTVVYGDDCVIAYN-GPDIGLGLKQCIGDS
Limnipi-C-20               DTFGIDYQFHFDGDYTSRDFLTVVYGDDCIVAYN-GPEVGAALAEVVKNA
Limnipi-C-FHMPV-1          DTFGIDYQFHFDGDYTSRDFLTVVYGDDCIVAYN-GPEVGAALAEVVKNA
Limnipi-A                  VHHDVYEDG---VGLPQCDYLSVVYGDDCVVAYN-GMRMGLDFAQTIEDT
ClownFish                  MHHDVHEVE---GALPVCDYLSVVYGDDCVVAYD-GLAMGNEFAETIRSA
Limnipi-D-GDDSYC43605      REVQG-------------AWMTFAYGDDCVVAHDGAQVDADTFVNSMKTA
                                               : ..*****::*::     .  : : :  :

Limnipi-B1-F37/06          FGMEVTPASKVGDVFEVPLDEVEFLKRKFFKIATVH-SDRIAMRLSVDTI
Limnipi-B2-XQTMC45688      FGMEVTPATKTGDEFCVDLDEVEFLKRTFFKLSTAK-YDRYAMRLSLTTI
Limnipi-C-20               FGMEITPASKVGEEYNVEIQDIEFLKRTFFRLRGQR-DDRIALRLSLTTI
Limnipi-C-FHMPV-1          FGMEITPASKVGEEYNVEIQEIEFLKRTFFRLRGQR-DDRIALRLSLTTI
Limnipi-A                  FGMTVTPASKLGDHFNVELHEVEFLKRKFMAFETEEGYKVAILALNENVI
ClownFish                  FGMEVTPASKEGYRFNVELRDVEFLKRKFVDYVTEEGFQIVTLGLDPEVI
Limnipi-D-GDDSYC43605      FGVEVTPADKGEGDIYVPIERVQFLKRTFVFSPEFN---RFVGKLDLDVI
                           **: :*** *      * :  ::****.*.     .        *.  .*

Limnipi-B1-F37/06          RQSLMWMRSSKTFDDQVYSLAIELSAWGEETYDREFAACKRMLEGGSLQV
Limnipi-B2-XQTMC45688      EQSLMWMRSERTFDDQIFSLAVELSAWGKSEYARIFTACKAVMDEG-QKV
Limnipi-C-20               FNSLMWMRNRKTFADQVFSLMVELSAWGREQYDLVVRKCRERLKENREVV
Limnipi-C-FHMPV-1          FNSLMWMRNRKTFADQVFSLMVELSAWGREQYDLVVRKCRERLKENREVV
Limnipi-A                  VQHLMWMRNLTTLPQQIQSLMMEYAAYGKEKYDKLRDTMKRRLAKQNLQI
ClownFish                  KQHLMWMKSASTFNQQIYSLMMEMAVHGQQAYDTLVSWLKDQLKESRAVV
Limnipi-D-GDDSYC43605      KQALMWTRNQHTFDAQMQSLSVELAAHGEEIYNEVRELVNRAMRKSGSQV
                            : *** :.  *:  *: ** :* :. *.. *       .  :      :
```

```
Limnipi-B1-F37/06           NVPFWRAAWETYLGIVDWSVAGAMYPRDLWDPVLEFDDDDDNDVVFFDDR
Limnipi-B2-XQTMC45688       NIPFYDAAWETYLGIVDWPVHGTIVPRDLFDPVLEISDTDSEVEFLYRET
Limnipi-C-20                TIPSYDLAFETYLGIVDWDVVGEVTAEELFPVKLEISDEDD---------
Limnipi-C-FHMPV-1           TIPSYDLAFETYLGIVDWDVVGEVTAEELFPVKLEISDEDD---------
Limnipi-A                   TVPGYDISWTMLNSVVMGDE------------------------------
ClownFish                   NIPEYRASKMLVDGVICDD-------------------------------
Limnipi-D-GDDSYC43605       AMAPYHMSVMCLAMTLQEVLDPGTLWTMQHFVLARTDNG-----------
                             :. :  :       :

Limnipi-B1-F37/06           LA
Limnipi-B2-XQTMC45688       I-
Limnipi-C-20                --
Limnipi-C-FHMPV-1           --
Limnipi-A                   --
ClownFish                   --
Limnipi-D-GDDSYC43605       --
```

Parechovirus Polyprotein Alignment.

```
Parecho-B-LV-1        ------------MAASKMNPVGNLLSTVSSTVG------------------SLLQNP---
Parecho-C             ------------MAANNDTTTSSVLTMAKSAIS------------------TLLQDP---
Parecho-E             ------------MAGQNKTDVGALLSSTASAVG------------------SLLQNP---
RtPV                  ------------MAALMEKAVDATLEKVVENVTGN----------------QAEQGS---
Parecho-A-HPev-1      ---METIKSIADMATGVVSSVDSTINAVNEKVESVGNEIGGNLLTKVADDASNILGPNCF
Parecho-D             -----------------MDTIATLMNKTG----------------------TLLNNP---
Parecho-F             MDTSLASSSSHQCCNCNKQEQKGPLDSIASLIPLVG---------------GLLQKP---
                                              :                               .   

Parecho-B-LV-1        SVEEKEMDSDRVAASTTTNAGNLVQASVAPTMPVKPDFKNTDDFLSMSYRSTTAPTNPTK
Parecho-C             VKEEQETNADRVSASITTNAGNVVQASVAPTMPFAPDFHPGDDYLSMAYTSDTGPTNPTK
Parecho-E             TVEEANTDSDRVAASTTTNAGNLVQSSVAPTMPFAPDFRNKDDFLSMSYSPETAPTNPTK
RtPV                  AGPPPILPEDRVQATTTVNASNLVQNPVAPTMPVKADFKNTDDFLSMSYQTGTAAANPTK
Parecho-A-HPev-1      ATTAEPENKNVVQATTTVNTTNLTQHPSAPTMPFSPDFSNVDNFHSMAYDITTGDKNPSK
Parecho-D             EKEFQEQNSDRMAAASTANAGNLAQAAVKPAAPLDAGFKNSDSFTSMSYSTKTFAQNIAK
Parecho-F             EIEQTEQTSDRISASTSNFAGSIVQAAVKPSAPRQPVYQTQDDFLSMQYTHRSAEVNPTK
                               : : *: :  : .:.* .  *: *  . :   *.: ** *   :   * :*

Parecho-B-LV-1        MVHLAHGTWTTNQHRQALVASITLPQAFWPNQDFPAWGQSRYFAAVRCGFHIQVQLNVNI
Parecho-C             LIDLRHVHWTTTNNRSHEVFRATLPEAFWATTDYPAYGQSRYFAAVRCGFHIQVQLNVNM
Parecho-E             MVLLGRASWSQSQARTTEVFRISLPNSFWAQNTQPAYGQSRYFAAVRCGFHLQVQLNVNM
RtPV                  LVVLGNAQWESTHNRTHEVFRIQLPTAFWANDSMPAYGQSQYFTGVRCGFHFQVQLNVNL
Parecho-A-HPev-1      LVRLETHEWTPSWARGYQITHVELPKVFWDHQDKPAYGQSRYFAAVRCGFHFQVQVNVNQ
Parecho-D             LVPVANANWLNSHGRSTELFAVQLPSGLYRDGTFPAQGISKYFKYVRTAFHFCLQVTVPQ
Parecho-F             LVEIGAGKWEINLSRGDNIFQVPLPLAFWDKPEKPAYGQSRYFAYVRTGFHFQIQVNVQT
                      :: :    *  .  *   :    **  ::     ** * *:**  ** .**: :*:.*  

Parecho-B-LV-1        GSAGCLIAAYMPKTAHDHMNTYTFGSYTNLPHVLMNAATTSQADLYIPYVFNHNYARTDS
Parecho-C             GTAGCLMVVYLPKTCHDNFNTYDFGTFTNFPHVLMNAATTSQADLYIPYINNRNYAKTDS
Parecho-E             GSAGALIVVYMSRTVFVNWESYSFGTFTNSPHIIMKAATTSQADLYIPYVNHHNFARVDT
RtPV                  GTAGALIIVYMPRTVMTNFNTYTFNSFTNLPHIIMNAATTTQADLYIPYTNHHNFARVNT
Parecho-A-HPev-1      GTAGSALVVYEPKPVVTYDSKLEFGAFTNLPHVLMNLAETTQADLCIPYVADTNYVKTDS
Parecho-D             GAAGSLILCYLPR-AAANREPFDFDSYTNLPSVVLNLATGTQADLFIPYTNHKNFAATNS
Parecho-F             GSAGSIIAFYTPMSARDDQENANFDSFLNFPHVILNANTVTQADLFIPYINFNNYAQTDS
                      *:**. :  * .       .   *.:: * * ::::    :**** ***    *:. .::

Parecho-B-LV-1        DDLGGIYIWVWSALTVPSGSPTTVDVTIFGSLLDLDFQCPRPPG-ADTVIYTQGKRT---
Parecho-C             DDLGAVFGFVWSALTVPAGSPTQVDVTVFGSLLDLDFQCPRPYG-QAINIYNQAPR----
Parecho-E             DDLGYVLGYVWSALTIPTGSPTSLDVTIFGSLLDLEFQNPRPFDSSAVQIVLEGPNR---
RtPV                  DDLGYLLGFVWSAMTVPTGSPTTIDITIMGSLLNLEFQGPRPFGSANFTLVDEAGDAKS-
Parecho-A-HPev-1      SDLGQLKVYVWTPLSIPTGSANQVDVTILGSLLQLDFQNPRVFA-QDVNIYDNAPNGKKK
Parecho-D             NDLGTVYCFVWTPLGTPTGAPADVEVNLLACLVNPNFQCPIPTN--------EGPVR---
Parecho-F             NELGYLSVRVWTQLTIPAGSSNTIDVTCYGSLLDLDFQNPRPILAPSFMEAPPAKKR---
                      .:** :   **: :  *:*:.  :::.  ..*:: :** *             .      

Parecho-B-LV-1        ---VRKTKTSKFKWVRNKIDIAEGPGAMNIANVLSTTGGQTIALVGERAFYDPRTAGAAV
Parecho-C             ---KRATKATRFKWTRNNIDIAEGPGSMNMANVLHTTGSQTIALVGERAYYDPRTAGTTT
Parecho-E             ---KRKTKASKFKWTREKIDIAEGPGAMNVANGLSPSGSQSTALVGERAFYDPRTAGAKA
RtPV                  ---KPKKKNSKFKWVREKIDIAEGPGTMNLANVLCTTGSQSVALVGERAYYDPRTAGTKA
Parecho-A-HPev-1      NWKKIMTMSTKYKWTRTKIDIAEGPGSMNMANVLCTTGAQSVALVGERAFYDPRTAGSKS
Parecho-D             ------DPITKFKWTREVRDIAEGPGTMNLANRLETNGARSLALVGERAHYDPYTAGVKH
Parecho-F             -----YSVTTKYKWSRKKIDIAEGPGSMNLANRLSTAGGQSVALVGERAYYDGRTAGTES
                               :::** *   *******:**:** * . *.:: *******.**  ***   

Parecho-B-LV-1        RCKDLMEIARMPSVFLGESTEPDG----RRGYFTWSHTISPVNWVFDDHIYLENMPNLRL
Parecho-C             RVKDLKMISQLYSVFIGNQAVPNN----QYGYFTWNSNVTSENFIFDNDIVPENLSNLGM
Parecho-E             RVKDLMDFARMPSVLRGDGTSATY----RSGYFSWAASTTPGNHVFNYGIWWEDLPNQYL
RtPV                  RLKDFISIAQMFSVSGNDQSTTSNNASTATGYMEWSATQTPGTALRTYSIWMEDFPNLRL
Parecho-A-HPev-1      RFDDLVKIAQLFSVMADSTTPSENHGVDAKGYFKWSATTAPQSIVHRNIVYLRLFPNLNV
Parecho-D             RIVDLMQYARLPSVVN-------------SGIFDWNGTTAPRTSIWKTNIQLAQIPNLKW
Parecho-F             RIGNMLEIYQMPSVIKG-------------GYFSWPNSSTPKTTIFQTNLNLEQIPNLDL
                      *  ::    :: **                * : *  . :. . :    :    :.*   
```

```
Parecho-B-LV-1        FSSCYNYWRGSFVIKLTVYASTFNKGRLRMAFFPNREG-----AYTQDEAQNAIFVVCDI
Parecho-C             MASCYAFWRGSIVCKLTVYASTFNKGRLRMAAFPNLGA-----RPSATACKNAVYMVCDL
Parecho-E             LSSCYTYWRGSIVLKLTIYASTFNKGRLRMALYPNYRASGSDTGYTDAEANNAIYVVCDI
RtPV                  FSSCYNYFRGSIVMKIDVFASSFNRGRLRMCAYPNINS------ATSSQLNNAVYTVCDI
Parecho-A-HPev-1      FVNSYSYFRGSLVLRLSVYASTFNRGRLRMGFFPNATT------DSTSTLDNAIYTICDI
Parecho-D             LSECFQYFRGSLVISMSVYSSMFNRGRLRLCWYPLHAD-----DFSYVSSRNAINVVCDI
Parecho-F             GCACYQFYRGSIVLELTVYNSAFNKGRLKMCFFPNTQT-----LYTYDQANNSQFIIADI
                         .: ::***:*  : :: * **:***::  :*            :     *:   :.*:

Parecho-B-LV-1        GLNNTFEMTIPYTWGNWMRPTRGNSLG-HLRIDVLNRLTYNSSSPNAVNCILQIKMGDDA
Parecho-C             GLNNTFELTVPYTWANWMRPTIGQPIC-RLRIDVMNALTYNSSSPTAVNCILQVRAGDDA
Parecho-E             GLNNTFELTMPFTWGNWMRPTRGIPVA-WCVIDVLNRLTYNSSSPNSVNCILQVRMGDDA
RtPV                  GENNSFELTVPFSWSNWMRHTRGPPIC-DISISVLNRLTYNSSSPNSVNVLFSVRLGDDA
Parecho-A-HPev-1      GSDNSFEITIPYSFSTWMRKTNGHPIG-LFQIEVLNRLTYNSSSPSEVYCIVQGKMGQDA
Parecho-D             GLNNTFELTLPFTSDSWMKHT-GETLG-RITVFNETKLTYNSASVNTVKCVVSMKAGPDF
Parecho-F             GLNSTFQMTLPFAYKDWMRPTRGDSVVGRIQIFVVNRLTFNSSSPSQIWCVLTAKAGNDF
                      * :.:*::*:*::   **: * * .:     :   . **:**:* . :  :.  : * *

Parecho-B-LV-1        MFMVPTTSNLVWQ--GLHSWGSEMDLVDSLDNPDE-IQDNEEI---QTQNVEAAQGEEAA
Parecho-C             EFFIPTGSNFTWQ--GLTSWGSEMDLVDSLDNPTE-IVNN------VESNIESAHGQAPA
Parecho-E             RLMVPCSSPYAWQSDGLRSWGSEMDLVDSLDNPTE-LMDAEET---ESHNVEAAQGEAAA
RtPV                  KFFVPTGSTHVWQ--GLSSWGSLMDLECPLDNPEK-IMDASDNPVDNSNNIEVTTQPEGT
Parecho-A-HPev-1      RFFCPTGSVVTFQ----NSWGSQMDLTDPLC---------------IEDDTENCKQTMSP
Parecho-D             TMMSPKETLHSLQ--APTSWGSEMDLTDPLDDSTDGVKEVEGAAAFQSSTCDYSQADDAA
Parecho-F             MYMVPSTSASIWQ-----SWGSEMDLVDPIDSPEVIQTQQDPKPTFSSPDAEYQQSSKMA
                        : *  :     *     **** ***  .:                    :      .

Parecho-B-LV-1        TEVGLRATENDGSLSEQLNMSQPMFLNFKKHKVNIYAASHTKVDHIFGRAWAVGVFNTET
Parecho-C             VAAGLRSTENDGTLSEQLAVAQPRFLNFQIQDISMFAVSHTLVDHFFGRSWLAGQFNYTS
Parecho-E             TAVGLKATENDGTLSEQLQSNQPMFLNFVKQNVSLFSTSHTKVDHIFGRAWNVTNYAYTT
RtPV                  SALGLRAAENSGSVGEQFNTSQPMFLNFVKQNVDIYTVSHTKVDHIMGRAWRVDSYNYST
Parecho-A-HPev-1      NELGLTSAQDDGPLGQEKPN---YFLNFRSMNVDIFTVSHTKVDNLFGRAWFFMEHTFTN
Parecho-D             EDTGLAAKENAGTLNEVVQAKPPKFINFDTCKRHIYTISHTRVDNFFGRAQRIAEFAWSD
Parecho-F             EDVGLASIENVGTQDQQISQAQPKFLNFKVQKRNIFTVSHTDVDNIFGRAWTVQSASYTS
                         ** : :: *. .:        *:**   .  ::: *** **:::**:

Parecho-B-LV-1        AAIQKFDLHFPTSTHGALSRFFCFWTGELNIHILNVSTTNAFLKVAHTWFGTDSGIARTA
Parecho-C             QNTTTLQIPFPTTQHGAMARMFAYFSGEVTFHITHIG--NCYILVTHTYYGNEEGRHRIY
Parecho-E             GDMHSIFVGFPTTQHGSLARLFAYFSGEVNFHITHTSSTGNFLTVTHTYYGTDSGIPRVS
RtPV                  NNMLSFVISFPKKTHAALTQFMAYFCGEVNFHIVHTSASNSFLEVCHTYVGDREGVERVN
Parecho-A-HPev-1      EGQWRVPLEFPKQGHGSLSLLFAYFTGELNIHVLFLSE-RGFLRVAHTYDTSNDRVN---
Parecho-D             KALKSEPLSWPNHNHQAMARLFAYFAGEINLHLVNES--DKHISVGHTYDLRDGSSDYG-
Parecho-F             STVIKIQCRAPASSHAVQMRFFAFFSGEINFHITNQS--AGELDVVHGFDLPDWMSFP--
                                *   *     ::.:: **:.:*:   .     : * * :

Parecho-B-LV-1        T--LESNGTMIIPPNEQMTLCVPYYSEVPLRCVKGSD----RNSAGLGSLFTQAVGR--T
Parecho-C             D--LASNGAISIPPDTQMSLTVPFYSETPLRTVKGSN----SKTSGLGTLFLRPVGQSGA
Parecho-E             EDGLLSSGAMIIPPNEQMTLCVPFYSEVPLRCVKPTGGAGAEHISGLGTLFLKPTGS-TD
RtPV                  SSNMSSSGSIIIPPNEQMSFCVPYYSPTPLRCVKTTQ---SNKISGLGTLFVRPIGT-TT
Parecho-A-HPev-1      --FLSSNGVITVPAGEQMTLSAPYYSNKPLRTVRDNN--------SLGYLMCKPFLT-GT
Parecho-D             ---VSSSGVMVIPPQTAMSMCCPWYSHTPFRPTRALS---QSGIKPLGTIWFKPEAE---
Parecho-F             ----ESHGVMAIPPMELMTICVPWYSQTPLRPVIDSS------MSLFGTLYLRPQVP---
                           * * : :*.   *::  *:**  *:* .             :* :  :.

Parecho-B-LV-1        ISNRVQIFVSFRCPNFFFPLPAPREATSRSIL----ERVDEANAEELEAVLEARTPDAPL
Parecho-C             AAGRITIYASIRCPNFFFPVPAPKRGTARAVLN---SRPGHFDQSTVEALGMAEDLDSDL
Parecho-E             ANGFVQIFVSLRCPNFFFPVPAPKQATSRSTIR---DMDYITDVCQLEAIGKSKDLDDPL
RtPV                  FDGRLEIFCSLRCPNFFFPVPAPKLATSRSVEE---SLRICHDSEIIQAAAAAEQPDEPL
Parecho-A-HPev-1      STGKIEVYLSLRCPNFFFPLPAPKVTSSRALRG---DMANLTNQSPYGQQPQNR------
Parecho-D             -SGTLIVYLSLRNPNFVFPLPSPKTATASSLSSQDDALFCLYSAERLSDILSALEVSPDE
Parecho-F             -SGRYIVRASLRNPNFFFKMPARKRVTALSILDSEDPLQYLISEDDFSDLMYNLKKDENA
                        .   :  *:* ***.* :*: :  :: :            .
```

```
Parecho-B-LV-1        RLKFNPEDPLKQLREAAKAYFN--------------------------------------
Parecho-C             LLEQRVDDNLAMALMRAKA-----------------------------------------
Parecho-E             EVGTKPKDPLAELKQRAFD--------------------------------------
RtPV                  QITERIVNPLESLYIRAEKWFKNRPETVATFRNESPDCSLIVQCNCCKILISVKDTGTKF
Parecho-A-HPev-1      ------------------------------------------------------------
Parecho-D             PFVPPQNSQDNSLSMRMLRRLG--------------------------------------
Parecho-F             DFERRKEHSRSRLADLIQHARQWYLD----------------------------------


Parecho-B-LV-1        -------------------------------------- ---------------------
Parecho-C             ------------------------------------------------------------
Parecho-E             ---------------------------WFDARTGFKTPLMNPCGDVEENPGPTMIVRVDQ
RtPV                  FLEADGTIKELSPLCPVDRPWINTALMMLDRRMGYRSRILCQCGDVEENPGPWFEVPVLS
Parecho-A-HPev-1      ------------------------------------------------------------
Parecho-D             ------------------------------------------------------------
Parecho-F             ------------------------------------------------------------


Parecho-B-LV-1        ------------------------------------------IMHSDEMDFAGGKFLNQC
Parecho-C             --------------------------------------------FFNRRQGCRSETLLQC
Parecho-E             DTEILITSLNGRLSFKKRVKGSDFCWPLSPNQLEDMHWIHLAQIQIEKRYGYRFWLLMLC
RtPV                  STVLIHFTNLTYKFYLKTVTG--EIEPLSICEERDRNYIFFAIKWFNKRSGYRSRLLSQC
Parecho-A-HPev-1      ------------------------------------------------------------
Parecho-D             ---------------------------------------------------YRRELLKQC
Parecho-F             ------------------------------------------------RKGHRKELLTQC


Parecho-B-LV-1        GDVETNPGPD----------------IELVYKNRGFYKHYGVRFGGHIYHLNSQDILSTA
Parecho-C             GDVESNPGPT----------------MELVYKDRGFYKHYGVRVGNAIYHLDSQDILSTA
Parecho-E             GDVELNPGPD----------------IELVYKNRGFYKHYGVRVGNYIYHLNSHDILSTA
RtPV                  GDVEENPGP-----------------IELVYKPRGFYKHYGVRTENGVYHLDSDDILKTA
Parecho-A-HPev-1      ---------M----------------MKLAYLDRGFYKHYGIIVGDHVYQLDSDDIFKTA
Parecho-D             GDVEENPGPFPNRAGNSQQINPTMHHAWLVYRDRGIYKHYGVQCGDSVYHLETEDIIDAA
Parecho-F             GDVEQNPGP-----------------IQLVYKDRGFYKHYGIRTEEGVWNLNSEDLVKTI
                                                  *.*  **:*****:     :::*::.*:..:

Parecho-B-LV-1        ITGKSDFIKEEDDGKWVHAMTAPLDYFTEKYINSMVGSKHIFSATSNCETIARDLFPGRK
Parecho-C             ITGQATFDKIEDDGCWLVSQVADLDYFTDKYVNSLVGTKHIFSATQNCETIARDVF-GDS
Parecho-E             ISGKATFLKEEDDGNWIHSMTAPLDYFTEKYMTSLVGSHHIFSASQNCETFARDIFPNVP
RtPV                  FNGEAIFRRDEDDGNWQVAFSADLDYFTEKYLNSMVGTKHIFSAQTNCETIARDIFPGKP
Parecho-A-HPev-1      LTGKAKFTKTKLTSDWVIEEECELDYFRIKYLESAVDSEHIFSVDKNCETIAKDIF-GTH
Parecho-D             LAGKAVFTCEPADSSWTRSQPLELDYFTQVYLDASVGSEHIFSARTNCETIVRDMFPNIP
Parecho-F             MRGEAKFILSPNTPDWKMEKEFEADHFTLQALESMKFSRHIFSCDLNCESLARAILSDTS
                      : *:: *        *        *:*    : :   :.****   ***::.: :: .

Parecho-B-LV-1        EITQSKALGIIGVILLSASLLSLLAVPWDYSSLQTVYNQSIEGDASG-LTLLSQRCMTFF
Parecho-C             SMTQGRALGILGVILLSAGLLSLMAVPWDVSSLQQVYNQSLDGDAEG-LTLLSQRCMTFF
Parecho-E             GISQAKALGIVGVILLSAGLLSLLAVPFDYSSLCYVYNQSIDQDVGG-LTLLSQRCMTFF
RtPV                  QITQSKALAIVGTTLLSAGLLSLLAVPFDESCLKVVYNQSLDGDSGG-LTMLVQRCMTFF
Parecho-A-HPev-1      TLSQHQAIGLVGTILLTAGLMSTIKTPVNAVTIKEFFNHAIDGDEQG-LSLLVQKCTTFF
Parecho-D             GISQSQALGLAGLILVSASTLGLCAASFSAQELRDMLNLSFQQNSDGYINSLVQKAMSYF
Parecho-F             RKSQAKALSMASMIILAAMFSYFMVGSVDELVMQKFFNQSNEPEEMT---NLVQRATTFF
                        :* :*:.: .  :::*        . .   :  . * : : :       * *:. ::*

Parecho-B-LV-1        SNTMCETFNNDLVKFIIKILVRLLCYIVLYCHAPNMLTTMCLGTLLVLDITTCEILSANT
Parecho-C             SNAMVETFNNDLVKFIIKILIRLLCYIVLYCHAPNLLTTLCLGTLLVMDITTCEVLSAST
Parecho-E             SNTLMETFNNDLVKFIIKVLVRLLCYIVLYCHAPNMLTTMCLGTLLVMDITACEVLSSSS
RtPV                  SNAMVETLNNDIVRFIIKVLVRLLCYIVLYCHAPNMLTTVCLGTLLVMDITASEVLSSGT
Parecho-A-HPev-1      SSAATEILDNDLVKFIVKILVRILCYMVLYCHKPNILTTACLSTLLIMDVTSSSVLSPSC
Parecho-D             SSILCETLAADIIKTIIKYLVRLFCYIVMYCHAPNLMTTMCMSTLLVLDVTDAKQLSSDT
Parecho-F             STTCVSSIEHTLVKTILMFLARILCYLIMYCHCPNILTTMSFGVLLMMDIKATHILDGET
                      *.   . :   ::: *:  * *::**:::*** **::** .:..**::*:.    *.

Parecho-B-LV-1        KALFQALVDGDVKSLVWKIAENMQFAQSKDEQAEDMAATFNFASDMVNFVP----MEQMR
```

```
Parecho-C             KALFQSLLDGDVKGLVTRIAESMTFARDTKEQAEEMSATFNYATEMVDFA-----RRPME
Parecho-E             RALFQALVDGDIKKLVTQIAENLQFAQSTQEQAEEMVETVRFSSDLLNVSMGGAYDQSMA
RtPV                  KALFQSMVDGDVGRLVSTISENLYYTTDPKEQIEDMKSTVRFANDVVDIQLS-----KLS
Parecho-A-HPev-1      KALMQCLMDGDVKKLAEIVAESMSNTDD-DEVKEQICDTVKYT------------KTILS
Parecho-D             TTLFKGLLEGDIKGFCERIVHCLQFDTT-EEETELKLETLRQAERMLSHEFDT--FVGQT
Parecho-F             AGLLKSLLDGDMKKLCEIIVEKIQFETH-EEKQQLVEDTIKATTSLFSTEES------LT
                        *:: :::**:  :   : . :      .*  :    *.. :

Parecho-B-LV-1        QEG-WREFNDVSMSFRHVEWWLTMFKKVYNVLKSIFAPSIEQKAVDWIDRNQEYIADVLD
Parecho-C             NQG-FAEFNTVSTSFRHIEWWLTTFKSAYNVLKGIFSPNLEQKAVKWMERREEQIADILN
Parecho-E             DQG-FKKFNEVSTSFRHVEWWLTMFKKLFNVLRSIFMPNEQQKAVKWIEMNAEKIASLLD
RtPV                  NQGPVKDFNEISMSFRHIEWWISVFKRIFAALKGIFAPSSEQKAVKWLNERSEAISQILG
Parecho-A-HPev-1      NQGPFKGFNEVSTAFRHIDWWIHTLLKIKDMVLSVFKPSIESKAIQWLERNKEHVCSILD
Parecho-D             NESPIKEFNAFTTASKNVSWWLGIFKQIIMFFKNLFSPNSNARSLQWLADHEGQICDLLA
Parecho-F             QQESFSDFNKFSMSARHMDWWMSKIQSLIKWLKTIFGSDTSREALNWLRDHQGFLSDLLI
                      ::     ** .: : :::.**:  :      .  :* .. . .::.*:  .   :..:*

Parecho-B-LV-1        HASNIIIKMKDPKEQRRASTISEYFEVLKQLKPIVSLCMKVAPSTKFSSQVFRIYSEMMR
Parecho-C             EASEVVVLMKDPKNQRSTEAIQRYFAILERMKILATICLKVAPSTRFSSQCFRMFSELLR
Parecho-E             EASDVLVLLKEVKNQRDQSVIKRYSDVLESMKPLVSLFVRVAPSTRFASTVFRIYSELLK
RtPV                  EASEVLVIMKDPTKARQKDNISRYLSILEAMKKLVAMCVKVAPSTRFSSTIFRMYSELLK
Parecho-A-HPev-1      YASDIIVESKDQSKMKTQDFYQRYSDCLAKFKPIMAICFRSCHNS-ISNTVYRLFQELAR
Parecho-D             TCNNHIIDMKKPENQRDPRFHDKHKWLCRRLTDVATIVYKSATYSPLATQVLRLNAEMAK
Parecho-F             TSSNHLISMRDPEYAKKKDNQVKHRYLLNRLSWCASIFAENQISSPLCNLLMRIHTQMLN
                       ..: ::  :.    :      .:      :.   ::  .    : :..   *:  :: .

Parecho-B-LV-1        VNVRVPANTDLTRLEPIGIWVSSEPGQGKSFFTHMLSTCLLKSCNLEGIYTNPTGSEFMD
Parecho-C             INVRVPTNTDLTRMEPVGIWISSEPGQGKSFLTHMLTTRLLKSCDLQGIYTNPTGSEFMD
Parecho-E             VNVRMPVNKNMTRLEPIGIWISSEPGQGKSFLTHALATKILQKTKLNGIFTNPTGSEYMD
RtPV                  VNVKTATNTQLTRMEPVGIWITGNPGQGKSFLTHALATKLLKKNGFVGIYTNPTGSEFMD
Parecho-A-HPev-1      IPNRISTNNDLIRIEPIGIWIQGEPGQGKSFLTHTLSRQLQKSCKLNGVFTNPTASEFMD
Parecho-D             IRLTQPSSGNMVRQEPVGVWICGDPGQGKSFFAHALIKAVQKKTKLVGIFTNPTGSDFMD
Parecho-F             CQYQQPYSGMFTRMEPIGIWITGDPGVGKSFLSHALISQICKMTKLSSVWPHPSGSEFMD
                           . .  : * **:*:*: .:** ****::* *   : :   : .::.:*:.*::**

Parecho-B-LV-1        GYIGQDIHIIDDAGQNREEKDLALLCQCISSVPFTVPMADLTEKGTFYTSKIVIATTNKF
Parecho-C             GYVGQDVHIIDDAGQNREEKDLALLCQCISSVPFTVPMADLAEKGTFYTSKIVVATTNKM
Parecho-E             GYCGQAVHIIDDAGQNREEKDLALLCQCISSVPFTVPMADLSEKGMFYESQFVIATTNKS
RtPV                  GYCGQDIHIIDDAGQNREEKDLALLCQCISSVPFSVPMASLDEKGMFYTSKVVIATTNKS
Parecho-A-HPev-1      GYDNQDIHLIDDLGQTRKEKDIEMLCNCISSVPFIVPMAHLEEKGKFYTSKLVVATTNKS
Parecho-D             GYAHQDIHIIDDAGQNREEKDLALLCQCISSVPFTVPMADLCEKGIQYTSKLVIATSNRT
Parecho-F             SYAGQDIHYIDDLGQNREENDIALLCQCISSMPFIVPMASLSEKGSEYSSKIVIVTTNKN
                      .*  * :* *** **.*:*:*: :**:****:** **** * ***  * *:.*:.*:*:

Parecho-B-LV-1        DFTSMVLTDPAALERRFPFHLRIRAVASYSRNNKLDVARSMAAMADGSCWEYSTDGGRAW
Parecho-C             DFSTMVLTDSEALARRFPFNFRLRAKVPYCKNNRLNVPDAMAQMADGSCWEISRDQGRTW
Parecho-E             DFVTTVLTDVEALKRRFPYKIKIRAKTVYSKDGRLYVPKAMSQMADGTCWEVSADG-RTW
RtPV                  DFTSTVLSDSGALSRRFPYRFSIRAAKSYCKAGKLFVPNAMGAMADGSCWEFSLNG-RDW
Parecho-A-HPev-1      DFSSTVLQDSGALKRRFPYIMHIRAAKAYSKAGKLNVSQAMATMSTGECWEVSKNG-RDW
Parecho-D             DFTTVVLSDHGALARRFPVYLRIRAKQQFQKNGKIDTAAAMIYMKTGEPWEVSTDG-YKW
Parecho-F             DFSTTVLSDSGALERRFPFKIKVRPHIAYSKAGKLDVDRSMTMMYEGSCWEICEDG-RTW
                      ** : ** *  ** ****  : :*.   : : .:: .  :*  *  *  ** . :    *

Parecho-B-LV-1        KTLSMDELVKQITAVYTQRSDALMVWKRKLNTIRNEMSPGSSTGRIFEPLEETLCALERR
Parecho-C             NSLCMDTLVKEITDFFKARQDALMVWQRKLNQVRNESGLSP-----LDSMDETIASLERR
Parecho-E             VDLDLNGFVDMIIKDYEERVDSLDKWKRKLGLHNQSP---------LDDISDTIASLERR
RtPV                  SPMNLDQLVDDISADIKQRQDALALWRRKLSLTNEGC---------FEDMEDTLAALEAR
Parecho-A-HPev-1      ETLKLKDLVDKITIDYNERVKNYNAWKQQLENQ------------TLDDLDDAVSYIKHN
Parecho-D             SPCNMDEIATTVANDIIRKHDAVSAWTN------------------MMEEDEGPIVHQDV
Parecho-F             KTCSISDLASKVSSLFERRTVAFQHWNTQLTQNQSADDDQ-----AILDVEVEEHPLSRR
                          :. :.  :      :      *                    :   .      .
```

```
Parecho-B-LV-1          FGQLADSLKDNYHKTADELIEAIEDMMAPSQSPFACFAE---SYRPTIKYTASDKVKSWV
Parecho-C               FGQISNYIRKEIGRCTDDLIEEMEDYLCEYDTPFQCFER---KQR-LRYATAPENIKQWV
Parecho-E               FGQMSATLSDYINRTSDELMEAVEDLFSPGETPFLCFEK---QQPLFAPRTTKEKVVDWV
RtPV                    FSNLASAIQSEMGKTADELIDAIEDMLSPLDTPFQCFER---AQPLFSQKTTCQKIHDWV
Parecho-A-HPev-1        FPDAIPYVDEYLNIEMSTLIEQMEAFIEPKPSVFKCFAN---KIG-SKISKASREVVDWF
Parecho-D               YDMCLNDITADCRR-LDDICQKLEADLNGLDSPFEALRK---RSD-MELDKAPTTVISWF
Parecho-F               FVLSSNYLQR-NGLSSQICLENLKDDFLRIDCPLPCLEIERDQREKIQLDRADSKFIAWI
                        :      :        .   : ::  :      : .:              :   .  *.

Parecho-B-LV-1          KNHMNRWKEFVMRNKGWFTLFSVLSSFLSILTLVYLHYKKEKKEEERQERAYNPQTAISK
Parecho-C               KKHMVRLKDFLAENKGWFLFFSILSTFLSVLTLVYLHYRSKPKEPEKQERAYNPQTQPKK
Parecho-E               KKTSPALEDFCERNKGWILFLSLLSSFLGILILVYQHYKNSQPDS-KEQRAYNPQTSQKK
RtPV                    KTQSSKLLQFCEQNKGWIMFFSLLTSFLGILAIVYSFYKDASKPT-ESQRAYNPQTANSK
Parecho-A-HPev-1        SDKIKSMLSFVERNKAWLTVVSAVTSAISILLLVTKIFK---KEESKDERAYNPTLPVAK
Parecho-D               RKKLTALRNWCQRNVGWLSLVSVLTTGASMLATYLILRK--PDPPAADNRAYNPQTTNPK
Parecho-F               QNAFNRIKSFYERNHAWFKLIGVLGTFIGIGLSIWRILK----TDEKDERAYNPQTTSSK
                                .:  .* .*: ... : :  .:        :        .:*****     *

Parecho-B-LV-1          KGGKPKLSLVKTTN---FVNEAPYMQDLEHCFAQTAYISSPETQDIIHCAALSEDTILVY
Parecho-C               GVRQVLRRRDEPVV---FKNEAPYMQDLEHCFAQTCYLSSSVTTDVIHCAAVSGTTLVCY
Parecho-E               G-GKHAKFAIKTTD---FRNEAPYVAELEHCFSQAAYISASSSSHVTHCAAMKENQLLVH
RtPV                    KGGKPK-FETTYAN---FKNEAPFDGELEHIFAQSAFITAETNHNVVHCAAYRENAIILH
Parecho-A-HPev-1        PKGTFP---VSQRE---FKNEAPYDGQLEHIISQMAYITGSTTGHMTHCAGYQHDEIILH
Parecho-D               GKSVFTMLPNQPVVT--QVNQSPYNGEIEHCMQATAYITGKNVNYPLHCIAWKQRYIVTY
Parecho-F               GGTLARSVVKAKAKFDLPTSNESWNEYIHLAQYCCYFATEKNMLNPIHGILLGSNQVLTY
                                           .: .:   :.       : :        *        :: :

Parecho-B-LV-1          GHSQFYFNRY--EDLRLHFKGAIFPIEGGKISQVTVNGQPMDLILVKIDKLPITFKNYTK
Parecho-C               GHSEFFVDNA--PDLVLHFKGASFAIDGGMISRVSLDGSAMDLVLIKVDKLPIVFKNYTK
Parecho-E               GHGAFFLEQE--EDLKLHFKGATFEIDSGQVSQVTLNGQKMDLMIVKLDKFPICFKNYTK
RtPV                    GHSAPFLHCE--DGIKFHFKNAVFDVDSGKVAHVTINDQKMDLILLKVDHLPICFKNYSK
Parecho-A-HPev-1        GHSIKYLEQE--DELTLHYKNKVFPIEQPSVTQVTLGGKPMDLAILKC-KLPFRFKKNSK
Parecho-D               GHIAEVLPHI--DCPQLWYKGNLFEIEEEAEMSYLSSNGGPMDLLLIHLPKFPIQFKDITK
Parecho-F               AHSRQWFGKYSDEQIHIFCKGVSYPCDELEIQELTYQGVDLDLCIIRIKKLPILFKNSKK
                        .*    .         :  *.  :  :   :  ::  .  :** :::  ::*: **. .*

Parecho-B-LV-1          YYTTEVGKETLLIWNSEKGRLAMPVQCVAPAGPVETMEGTITHKTYSYKVASKKGMCGGL
Parecho-C               YYTKTIGRDSLLLWSSPQGRLAMPVTNVHMAGNLVTLEGTQTAQTYSYAVSSKRGMCGGL
Parecho-E               YYTNKIGTDSLLIWNSPQGKLAMPVTNVQMSGPITTQEGTQTYKTYSYKVSSKRGMCGGL
RtPV                    YYTNEIGTDNMLVWTTPKGRMAMPVECVSPGGPITTAEGTQTTHTYNYKVKSKKGMCGGL
Parecho-A-HPev-1        YYTNKIGTESMLIWMTEQGIITKEVQRVHHSGGIKTREGTESTKTISYTVKSCKGMCGGL
Parecho-D               YISNGISKEAYLIFSTPMGRMMYEVKNPYLSGFHQTLEGTKNSETITYCLNTKKGMCGGL
Parecho-F               HICAQIGKEPKLMWLTKYGMLVKDVGQVYYSGRNITQEGTVSANTVRYPVDSKKGMCGGI
                        :    :. :  *:: :  * :   *     .*   * *** . .*  * : : :*****:

Parecho-B-LV-1          LVTRVHGTFKVLGMHIAGNGQVARAAAVHFISNGAAGFMDQGVVVAKEKLQKPIYLPSKT
Parecho-C               LVTRVDGSYKVLGMHIAGNGSIARAASLHCLTQ----YRNEGVIMSKVFVGHQVFQPSKT
Parecho-E               LVTRIEGAYKILGVHIAGNGMIGMAAAVGFIQN-APEYHDQGVVVKRESLPLPVFQPSKT
RtPV                    LVTRVKGAFKILGMHIAGNGQIGVSAAMGFLQN-DNNFKNEGIISQVKKAVGLVHQPTKT
Parecho-A-HPev-1        LISKVEGNFKILGMHIAGNGEMGVAIPFNFLKN---DMSDQGIVTEIT-PIQPMYINTKT
Parecho-D               LITKIDGNFQIAGLHISGNGVVGSSAMLKVLKQ----SSNQGVIIETTTSPVRVFQPGKT
Parecho-F               LITNVGGAYKICGMHIAGNGFEGVSVLLYPTLQ----MKDEGVVVNVE-KAQIMFTPAVS
                        *::.: * ::: *:**:***  . :  .    :      ::*::         :.    :

Parecho-B-LV-1          ALNPSPLNGVVPVKMEPAVLSPHDTRLEVIMPSVVKTAAA-KYRVNIFNPDFEIWERVVD
Parecho-C               SLHPSPLYKAFEIKMEPAVLSPHDPRLEVALPSVVKDAAS-KYRVDVFKPDQKLWMYVLD
Parecho-E               KISPSPLNGIFPIKMEPAVLSPFDSRLIEPMSSVVKTAAL-KYRVNVFNVDQQLFLKVVD
RtPV                    KLNPSPVHDVVPVNMQPAVLSPRDLRLLEPMESVVKSAAQ-KYRVNVFNVDQDLWMSVLD
Parecho-A-HPev-1        QIHKSPVYGAVEVKMGPAVLSKSDTRLEEPVECLIKKSAS-KYRVNKFQVNNELWQGVKA
Parecho-D               QIHPSPLHGLWDVKMEPAVLSAHDPRLEVECTSVVKMCSNDKYVGNVFSVDMDIFKMAMS
Parecho-F               KLQKSPIHGCYEELKQPAALSPRDPRIEVPCNNLVKLSAQ-KYRRNRFDPEESLMNAVES
                         :  **:         **.**  * *:      ::* .:  **  : *. : .:   .
```

```
Parecho-B-LV-1        ELKSKFRTKLGIHKHVSFQKAVQGFSSLSSLDLSTSPGQKYVEKGMKKRDLLSTEPFWMH
Parecho-C             EVKSAFRSKLGIHSKVTLEQAVLGYSDLSSLDLTTSPGTKYVKLGFKKRDLLSLDPFFVH
Parecho-E             YWKQKFRQTFGLTQRVSIQQAIQGAGKLASLEISTSAGYKYASRGIKKKDLISLEPFWIS
RtPV                  QVKEKFRSTLGIQTHTTVEVAVCGGTEMNSLDLSTSPGLKYVQMGFTKRDLIQTDPFWLH
Parecho-A-HPev-1      CVKSKFREIFGMNGIVDMKTAILGTSHVNSMDLSTSAGYSFVKSGYKKKDLICLEPFSVA
Parecho-D             NVLAKLYRQFGTNKTVSMEKAIVGFGKFNRIDLATSPGIKYSLK-YKKRDLIQYDPLVVH
Parecho-F             FVATSFQKYYGKHHPLMLHQAVKGVDGLNPIDMNTSAGVKYQSRGLKKRDLLAIEPFWIS
                           :    *      .. *: *   .  ::: **.* .:     .*:**:  :*: :

Parecho-B-LV-1        PQLEGDVKDILGAVYSGKKPHTFFAAHLKDELRKKEKIAQGKTRCIEACSIDYVIAYRVV
Parecho-C             PVLLEDVKQLLAAVYRLEPPRTIFTAFLKDELRKRDKVKNGKTRCIEACSIDYVVAYRVV
Parecho-E             DELVEDVKAILGDIYAGRVPKVVYTAYLKDELRKIEKVMTGKTRCIEAGSVD-LIAYRVI
RtPV                  PLLATDVKKILDDVYTGKKPWTVFAAHLKDELRKLAKIQQGKTRCIEACAVDYVIAYRVI
Parecho-A-HPev-1      PLLERLVQDKFHNLLKGNQITTTFNTCLKDELRKLDKIASGKTRCIEACEVDYCIVYRMI
Parecho-D             KVLVEDVQKTFEDVKSG-CVQTVFATHLKDELRKLPKIKSGSTRVIEACSLDYVIVHRMI
Parecho-F             DRLAMDIKKYWDALSSGKNPEVLFGCFLKDELRPIQKIKDANTRCIEAAPLDYVLVHRQI
                        *   ::     :       . :   ******   *:  ..** ***  :*  :.:* :

Parecho-B-LV-1        MSSLYEAIYQTPAQELGLAVGMNPWTDWDPMINVLQPYNYGLDYSSYDGSLSEQLMRYGV
Parecho-C             MSSLYEAIYQSKPQELGLAVGMNPWTDWDAMLNSLLPYNYGLDYTSYDGSLSEELMRSAV
Parecho-E             MSELYEKIYQTPPQVLGLGVGMNPWVDWDSMMEALLPYNYGLDYASYDGSLYDELMRSDV
RtPV                  MHNFYEKIYQTMPQQLGIAVGMNPWQDWHFMMESLYQYNYGLDYSAYDGSLSDELMRYGV
Parecho-A-HPev-1      MMEIYDKIYQTPCYYSGLAVGINPYKDWHFMINALNDYNYEMDYSQYDGSLSSMLLWEAV
Parecho-D             MGEIYEKIYATAPQLTGFAVGMNPWTDFDMLVRSLHDNVYCFDFRQWDGSLPPELMDAGV
Parecho-F             LGGIYSQFYSKPCVLTGMCPGINPWTDFDMMMSCLHENCYNLDFSKYDGSLSAEIMEMGV
                      :  :*. :* .     *:  *:**: *:. ::  *    * :*:  :****   ::   *

Parecho-B-LV-1        EILAYCHEQPEAVMILHEPVINSQHLVMDEIWHVNGGMPSGAPCTTVLNSICNLLVCTYL
Parecho-C             EVLAYCHEDPDQVMVLHETVLNSEHLVMDEIWEVKGGMPSGSPCTTVLNSICNILVCRYL
Parecho-E             EVMAYCHVDPEQVMILHETVINSEQAVMDEIWSVHGGMPSGAPCTTVLNSICNLLVCTYL
RtPV                  EVMAYCHTNPEQVMILHEPVINSVHHVLDEIWFVDGGMPSGSPCTTVLNSVCNVLVCAYT
Parecho-A-HPev-1      EVLAYCHDSPDLVMQLHKPVIDSDHVVFNERWLIHGGMPSGSPCTTVLNSLCNLMMCIYT
Parecho-D             WVLSGLHEDPSLVRNLMAPVITSEQICLDAKHLVYGGMPSGAPCTTVLNTVCNLLACEYA
Parecho-F             EIIKGCSEVPHLVDQIFKPVITSQHHVFDEVWTVYGGMPSGAPCTTILNTICNLLMSRYC
                       ::      *  *  :  .*: * :  ::    : ******:****:**::**:: . *

Parecho-B-LV-1        AYEQSL---DIEVLPIVYGDDVIFSVSSPLDAEYLVQSAAQNFGMEVTSSDKSGPPKLLK
Parecho-C             AYSQGP---SVECLPIVYGDDVIFSVSEEIDASCLVEQARREFGMEITSSDKSAVPTLLN
Parecho-E             AWEQDP---TIQVLPIVYGDDVIYSVDQPLDMERFVSQAKNCFGMDVTNTDKTPIPTLVD
RtPV                  AKVLDP---ECDVLPITYGDDVIFSVDRPLDMEKFCEIVKNSFGMEVTGSTKDGPPKLLD
Parecho-A-HPev-1      TNLISP---GIDCLPIVYGDDVILSLDKEIEPEKLQSIMADSFGAEVTGSRKDEPPSLKP
Parecho-D             SLKIG-----AESLCVAYGDDLLFSTPVPINPVHVLQIWKCDLGLTATGPDKTEHVPAVC
Parecho-F             AILASSALEQDEVVIFAYGDDVVVSTEEEMDTTVFVDSMYKHFGMTVTDANKKLGINKLD
                      :   .      : : ..****:: *    ::   . .     :*  *..  *

Parecho-B-LV-1        MDEIEFLKRTTKFFPGSTYKVGALSLDTMEQHIMWMKNLETFPEQLVSFENELVLHGKEI
Parecho-C             LEEVEFLKRTTRFFPGTTYKVGALNLSTMEQHIMWMKNLSTFPSQLQSFENELALHGQHV
Parecho-E             FDEIEFLKRRTKIFPKTTFRVGALNLDTLEQHIMWMKNLDTFPEQIVSFENELSLHGRDV
RtPV                  FEQIDFLKRTTKFFPGTRFMVGALLEETLMQHIMWMKNLDTFKDQLMSFENELCLHGEDK
Parecho-A-HPev-1      RMEVEFLKRKPGYFPESTFIVGKLDTENMIQHLMWMKNFSTFKQQLQSYLMELCLHGKDT
Parecho-D             PLDIEFLKRTPKFFPNSGFIVGALDLDNMLQHIMWSHSSEAFLQQLSSFENELVLHGEHV
Parecho-F             KNEISFLKRKPKFFPGTQFFVGQLDLSEMKQNIMWMHGMKEFEQQLTSFENELVLHGQEV
                        ::.****  .  ** : : ** *  . : *::** :. . * .*: *:  ** ***..

Parecho-B-LV-1        YDDYKNRFNPILNQWRVCMQDYEVALHRMLRYVF-
Parecho-C             YESYQNVFQEVLKPWKITMEDYDVVVRRMLFYVFE
Parecho-E             YDFYKEKFKDDLAKWSIFMNDYDVVIRRMVGYVFE
RtPV                  FNEIKNKLQPYLMKWNCCMNDFDVVKRRMISFVFN
Parecho-A-HPev-1      YQHYIKILEPYLQEWNITVDDYDVVITKLMPMVFD
Parecho-D             YNEVQSKVNKHLSKMGMNMLPFNVVYNKMVQLVFE
Parecho-F             YERTIQEMKDHLDGYPLNFSNWNTALRRMTGYVFG
                      ::   . .:  *      .  ::..  ::   **
```

Potamipivirus Polyprotein Alignment

```
Potamipi-A      MATTKDTSSLSIISDIVKPVTALLADAGTEQAANASDRNGGTVSVNASSSTQATSKLKHG
Potamipi-B      ---------MDLIKSVVSTASGLLNDPSTEQANNASDRVGGMLGANASLASQASVQIKYG
                         :.:*..:*...:.** *..**** ***** ** :..*** ::**: ::*:*

Potamipi-A      FFPPKETHDKFWSQATKPTTAFINPTKLVEIKSESWVDTHIEGHALMKVDLPKDFYPTTA
Potamipi-B      FFPPTSSADRFKSQGTDSKASQINPSKLVELGSGTMTSTVATGEEMVKLELPKAFWKNKS
                ****..: *:* **.*...:: ***:****: * : ..*   *. ::*::*** *: ..:

Potamipi-A      FPARGQTRYFASIRTAFDFELQVNAATGCSGGVLIGYVPKGVDLTKGCFRSAPLLCSEVL
Potamipi-B      FPAHGQTRYYRSVRCSFDFEIQVNAATGCSGSIIALYVPDGVDLTKINQKTWPMFASTII
                ***:*****: *:* :****:**********.::  ***.******   :: *::.* ::

Potamipi-A      NIGTNTTAKLHVPYTNFQNYANTDTDDLGSLYIWVWSKLIVPVSTPNDITVVLYGAMNEL
Potamipi-B      NIGPNTSGKLHVPYVCDKNYVNIDSAELGSFVIACWTPLLVPTGTPNEVSLTVYGAISDL
                ***.**:.******.  :**.* *: :***: *  *: *:**..***::::.:***:.:*

Potamipi-A      DLQCPRP--QGPLRAKYDMAEGVGNMNLATGLYTRRSQPVALCNESAAVDSTTAGARCAV
Potamipi-B      DLQAPKPVAQGPHRNKIEIAEGVGLMNLSNSSVTKKAQYISLCGESAEVDCTTAGMSRAV
                ***.*:*  *** * * ::***** ***:..  *:::* ::**.*** **.****   **

Potamipi-A      RDILQLARVPLPAIALQTDDVTTLWENTKAKGSSIFQANFAFNSLNNLTMFLANGYKYCR
Potamipi-B      TDVMDILKIPYIQGKVSEDTIYTPWAATAAKGDSIYDYDVHFDGINPAAGLMSGAFRYCR
                 *:::: ::*     :. * : * *  * ***.**:: :. *:.:*  : :::..::***

Potamipi-A      GSVLFHVTVFNSTLHKGRLKLAFAPTTNDSTYPGQFTELQANNLFYTVHDIGLNSTATLL
Potamipi-B      GSVIFTVNCYNSTLHKGRLKICFFPLAEGEENYKAYTEEEANNAFFVVHDIGLNSSMDLV
                ***:* *. :**********:.* * ::..     :** :*** *:.********:  *:

Potamipi-A      VPFVCNSWVRSTVQPLGRFQVFVNSRLSVSPAMAANVRFIVSTSLGDDFELLVPYSRGLN
Potamipi-B      VPFSSNTWLRGSKEAVGRLSIFVNSKLSVTPATSNSISFTVTTRLGDDFEYYTPYNSNMV
                *** .*:*:*.: :.:**:.:****:***:** : .: * *:* ******  .**. .:

Potamipi-A      FQMKDETGADDEQDDPLMSWGSTMDLTDGAAVSDTSEAAAAAGLEAPEEIDKSDRQINAR
Potamipi-B      LQAG--------------------EMGDGVVEDNTAAAASSAGMVAPEEMDKEDYLNEHN
                :*                      :: **.. .:*: **::**: ****:**.*   : .

Potamipi-A      P-TSLNAVVKKVTVVRASHTKLQALFARSQYSGRISLTEGN-ASTSIPVTKTGYMAFLKL
Potamipi-B      PGTGYAMRIQKVQIMRAVHTNINALFGRAQYVGRYTVGADTGHVFDLPIPVAGYWNIPKM
                * *.    ::** ::** **:::***.*:** ** ::  ..    .:*:. :**  : *:

Potamipi-A      FRYWTGDLNLHIMNVSTVPVAVAHGYDTETVITPEKISTMGSVIIMPNQAASITVPWYFD
Potamipi-B      FHFFTGDLNIHIYNATEGPVKVAHSYTKMTESSLEFISSLGCVVVPTQQFASLTVPWYFD
                *:::*****:** *.:  ** ***.* . *  : * **::*.*:: .:* **:*******

Potamipi-A      KPMRDTEATDSLGHLITTTFGATTLLVWFSFQTISLFYPKMVPKTATTRTWIEQ------
Potamipi-B      KPVRYFRGERPLGNIILYTLGHGKVFVWISFTNLKLFQPIGVPKLATLRTWLKDGALCNC
                **:*  ..  .**::*  *:*  .::**:** .:.** *  *** ** ***:::

Potamipi-A      ---NKDTTLETS---------------------------------------AFESVKKN-
Potamipi-B      WNLASDLALNASNALDEDEPLMNFVTHNNTRVTNLDKLMEKTEEAGWLRDLTREGVEENP
                    .* :*::*                                       : *.*::*

Potamipi-A      ------------------------------------------------------------
Potamipi-B      GPNYGHQDRNQAFTRINLCYMKNEEGSFHYGLKVQGFVLHRPKGNILNEEHYWRKPVLSQ


Potamipi-A      --------------------ILAGEFNSPLED-------------------------APV
Potamipi-B      MSGWTVCPEDYVSGSRMLDNLLLGSTMEIIHDPKGLFFGPETPFIMSWMAKYIAASYIPI
                                    :* *.  . :.*                          *:
```

```
Potamipi-A      QARQPTALQKLLEDYEIGEILTDKMWIRDLTQEGVEPNPGPHAELVYKDRGLYRHYGVRY
Potamipi-B      QHRRNTWDEEVLFEEEFDDYHQEGGWIRDLTAEGVEPNPGPLVELVYKQRGLYRHYGMRH
                * *: *  :::* : *:.:    :  ****** ********* .*****:********:*:

Potamipi-A      AGDVIHMDSENILETATTGKVSIIKVPDDGKWIVSTSVMLSELKAHALEKSEGLISYFSA
Potamipi-B      NGQVLHMSTDNILMAAVDQRVPIVLVDDDGTWVKEESVDITSARMAAIERSATMPQVFSA
                 *:*:**.::*** :*.   :*.*: * ***.*: . ** ::. :  *:*:*  : . ***

Potamipi-A      TNNCETFAKEALGIESVSQARALATFGFIIATTTGVIAAGQTNVADYVKAGAGKLADMVT
Potamipi-B      DNNCETWAKDALGIKSITQARALLVFGLIMASVTGVMTAGQTGLTQKVIDGVKNAYQGSK
                 *****:**:****:*::*****  .**:*:*:.***::****.::: *   *. :  :  .

Potamipi-A      GMGKAIKANVTTFFKEQLLDSIKCDVVKGLFKLVVRVICYGILFCSCPGVLTGACVATLI
Potamipi-B      NMFSAMGETIKGFFKEQIFDAIKCDITKTLFKLILRTVCYGICFCSSPGLLTGAAVGTLI
                .* .*:   .:. *****::*:****:.* ****::*.:**** ***.**:****.*.***

Potamipi-A      AMDLSTVDGLSKMTKDLLSAMLEGDLFGAAGAMSDIIYLNNPDREYMVKETTRELKSFLG
Potamipi-B      AMDLANVEGLTSTTKDLLTALLNGDMGECAAAMSNIIYENSSDREMMVRTACEEFSRLVG
                ****:.*:**:. *****:*:*:**:   .*.***:*** *..*** **: : .*:. ::*

Potamipi-A      EFREPQ--APPFQDFNSTTLAAKNLEWWLAGMQRLWDWLKKWFCGDEQDVIKDWFETRRN
Potamipi-B      ENPDKVRGQGPFSGFNEASLAAKNFEYWFGKIRSFFFWLKSFFVTDEEGKIATWFEDNME
                *  :       **..**.::*****:*:*:. :: :: ***.:*  **:. *   *** . :

Potamipi-A      EVLKVLALGDSLLSQSTMSSVLRDPDFQYKVTQAKQKLTALKLLAIKGGVSYLMSTISIM
Potamipi-B      LVARYASEADEVLAEYMNVNNRRDANYQRRVKSIVKQMTVLKNLAVRAHCVPALTLVSQL
                 * :  : .*.:*::     .   **.::* :*..  :::*.** **::.      :: :* :

Potamipi-A      LGKLQNIPHPPGMPGGAMRMEPVGIWISGKPGSGKSSLSLSIMTGIAEYVDKHSIRDKDG
Potamipi-B      LPRLQHLNLPPAVGDGIFRFEPIGVWLSGGPGCGKSMLASLVMKGTSEELERR-------
                * :**::  **.: .* :*:**:*:*:** **.*** *:  :*.* :* ::::

Potamipi-A      KRVNPFTIFPHATGSEYFDNYEGQYFHVIDDLAQDREENDVKLICQCISSIPFSVPMANV
Potamipi-B      GHTGTKEVYTEAGGSQFYDNYRNQFFHIIDDIGQCREEEDLKLICQVISSIPFTVPMADL
                 :...  ::..* **:::***..*:**:***:.* ***:*:***** ******:****::

Potamipi-A      NDKGMSYTSRVVIATTNRADFSTQVLSCEQAFKRRFQYQLWVKPNHTY-LTTSGKLDLPK
Potamipi-B      AEKGTQYSSKIVIATTNRTDFASVVLTSDKALQRRFPFCYTIRVREKYRVVDTQLLDVLK
                 :**  .*:*::*******:**:: **:.::*::*** :   :: ...* :. :   **: *

Potamipi-A      ALEAGAVDNGHCWMYSSSGQNDQKDVLDIKQLSKMIGDDIIARVKITEQANKRLGRFKTN
Potamipi-B      AQADGSLERGHCWEYDRFGSSKNFKILDLKELTSLVADRFQSKMVVTHTMNQGLTDFQQF
                *   *:::.**** *.  *...: .:**:*:*:.::.* : ::: :*.   *: *   *:

Potamipi-A      P----------------------------VKIEEP---------------IETDTKEMVK
Potamipi-B      PTSRVCVTPGTLEHGLNTIDAEDVTKRGEVKVQAPGPRKTWTHPGPQDTAYKKDFHSIIK
                *                            **:: *                :.* :.::*

Potamipi-A      ------------------------------------------------------------
Potamipi-B      NCGFELIAKQEYATKIMVFVKASGGDVSADLRRGLQDVCVSDKKVVVFYMVGEEVKDSYD


Potamipi-A      --TVMKQGAEENENKLYARFDK------------------------LGQTVELSVSFDS-
Potamipi-B      KLTYSKKSWDGSDNRVEAVFVLNMTDRGVIQDTTMNSHAIDELRSLLGENDEEVVHFEAP
                  *  *:. : .:*:: * *                         **:. *  * *::

Potamipi-A      ------------------EDELDVVG---SMRFIDGATEETPFASVNEN-------HFMP
Potamipi-B      KKDIMDQVFSVVNYNADYDEEVDLSTDEKCLSYLEVAGEESPFKHVQMQRKDGGFVEVGC
                                  ::*:*:     .: ::: * **:**  *: :        ..
```

```
Potamipi-A      DVKVDEKALSWYEKKMNAFREWWKKSKDKILGISIIAGCVTAIAGAFTAYFFLESATKIG
Potamipi-B      KLPLSAKIAKWYDGRITAWKIWYKRHKYVLQGISIAVGILTAIAGICTAWGVLRRHISEV
                .: :. *  .**: ::.*:: *:*: *  : **** .* :*****  **: .*.   .

Potamipi-A      SLKALVSDSLARLPRVNEPVDIME---PERAYDQTQKIRATAKKVRMPQGGFNPQEYAHL
Potamipi-B      NLEDVKKSQILDFLEEKQEKNFFGGDAQGRAYGGGPGLAVKKKKVVVGQG-FAPAEYAHL
                .*: : ...:  : . ::  :::      ***.    : .. *** : ** * * *****

Potamipi-A      SKCAVVMYGNFVKEFYGMASGPHQVLTYKHYFKSGGTLNGLYWNGLKYSVQDPDDVEFEF
Potamipi-B      YKRCVTMEGPDGKRFYGMVTASKQVLTYKHYFKMGGRLTTLWYDNLAYSPAEDEIELEEV
                 * .*.* *   *.****.:..:********** ** *. *:::.* **  : :    *.

Potamipi-A      FYFEDGDRVYETDMVKINFKKLPFQMKCVQNYLSEPQQGVGGVLIFGGTTTHSVPVHNVR
Potamipi-B      EYYDDKGVNYPSDCVLINFEKLPFNMKTGMKFLAPPKSGDSGVLIYGGKDIYSQGVENVQ
                 *::* .  * :* * ***:****:**   ::*: *:.* .****:**.  :*  *.**:

Potamipi-A      KILNYCMDVEGDTWHFNNCLEYEAQTYPGFCGGMLCQKINGTWRVVGMHHAGDGMMFGNA
Potamipi-B      TMNSYSALIAGKHTTYIDGISYQTRNQMGMCGGLVCQKQNGSWKIIGMHHAGDSMMFGNA
                .: .*.  : *.   : : :.*:::.  *:***::*** **:*:::*******.******

Potamipi-A      VPILR--IAQGVVVTKQNTGKTVFSPSKTALRKSPLYGLVETTMEPAVLHPRDPRLERQV
Potamipi-B      FRIKVGVVGQGVVTEIRKNPIPVFNPTRSKIMKSPLYGVVETRMGPAPLHPHDPRVEEEI
                . *    :.****.  ::.  .**.*::: : ******:*** * ** ***:***:*.::

Potamipi-A      DNLVKDASDKYRIDVFAPNEKNFERAKLYTKKQLFGVIGRHGNWNIQQACSGNGGNPLDL
Potamipi-B      PHLVKHAAAKYRVDKFEPDEENLNRAMMQTASQLFAVTGRCGRWTIEEALSGGGGNPVDL
                 :***.*: ***:* * *:*:*::** : * .***.* ** *.*.*::* **.****:**

Potamipi-A      TTSPGFKYTQKNLKKKDLIKKLDDGSYIFNHDFEKDVEKMLEEIEQGVAKPIFTASLKDE
Potamipi-B      TTSPGRKYTLKKLQKKDLVHRNENGILVPTPSFREDVNEAVRRIERGEAQTTFVANLKDE
                ***** *** *:*:****::: ::*  : . .*.:**:: :..**:* *:. *.*.****

Potamipi-A      LRPIPKVKEGKTRCIEMCEVDYTVAHRMILGSIFESIYNTGATATGIAVGIDPVTDFHVL
Potamipi-B      LRPLAKIKAGGARCIEMCEFDYVVAHRMILGPMFEKIYNAEMTSTGIAVGCNPYTDFNLF
                ***:.*:* * :*******.**.********.:**.***:  *:****** :* ***:::

Potamipi-A      QGIMYPHWYAMDYSRYDGSLSEGLMRAGVEILSACHEDPDLVVKTLEPVVNSVHRVADEL
Potamipi-B      FGTASENWIAIDYSRYDGSLSEGLMRKGVEVLAACHEDPELVEKLLEPVVMSTHLVADEE
                 *    :* *:*************** ***:*:******:** * ***** *.* ****

Potamipi-A      WNVVGGMPSGSPTTSVLNSICNLLVVRTVALECGLEDLDDMVIVTYGDDVLFSTKHEIDF
Potamipi-B      WFVKGGMPSGSPCTSVLNSVCNLIVVKTACLSAGVEKPEDIQIVTYGDDVLMTIDYDFCD
                * * ******** ******:***:**:*..*..*:*. :*: *********:: .:::

Potamipi-A      SEAPAMIKEMFGMDATSADKQSTNFDVSPEEATFLKRNFRLFPDTLFVTGVLTLDTMLQK
Potamipi-B      DALPEIIHSQFGMEATSANKLSKTLKVSPADATFLKRSFRLFPDTQFVTGVLDLENMMQK
                .  * :*:. ***:****:* *..:.*** :******.******* ****** *:.*:**

Potamipi-A      IQWCRGLNEFKQQFESFTQELVLHGPHVYHQVMSMCAHRLARMKMYAPTYQEALRDATTK
Potamipi-B      IQWCHGKEAFIQQWTSFTQELVLHGRETYERVIRLCSPRLDQYKIFTPRYDQRYKEVYQS
                ****:* : * **: ********** ..*.:*: :*: ** : *:::* *::  ::.  .

Potamipi-A      IFY
Potamipi-B      IFM
                **
```

Shanbavirus Polyprotein Alignment

```
Shanbavirus-A1      MESLNNLISTVPQTINTLLQDPAVEESENTSDRVMAVTSALSTDVGQHATRPAAGLHPVT
Shanbavirus-A2      ---------------------------------VLAVTSALNTDFGQHATRPAAGLNPVL
                                                     *:******.**.***********:** 

Shanbavirus-A1      GPTEPTNTGTEQDLFIKMFSRNVYMESADFGTGQNNWDIIARVPIVQRLFQGGRPIEFLA
Shanbavirus-A2      HHTEPTNTGVDYDLFIKMFSRNVFLESVQFGTGQAAWNIPARVPITQKMFSTGKPLQMVG
                      *******.: ***********::**.:*****  *:* *****.*::*. *:*::::.

Shanbavirus-A1      RMWKYFRGDIKLTIQCNAPAGAVGAFVAYWVPHHIIDQQNKWSTGTVFNLPHVIFNVGNM
Shanbavirus-A2      KMWKYFRGNLKITLQCNAPAGAVGSFVAYWVPHHLLDQEGKWSPACIFNFPHVIFNVGNM
                    :*******::*:*:**********:*********::**:.***.. :**:**********

Shanbavirus-A1      NIAHLVVPFTFHKTYMTTSQNRDFGNLFVQVLSKYNVPSGTPTGIKVSIFGALDNPTFTN
Shanbavirus-A2      NIATLLVPYTYHKTYMTVGVNDSFGKLYIQIIAPYNVPTGSKTGIRISVFGALEDITFTN
                    *** *:**:*:******.. * .**:*::*::: ****:*: ***::*:****:: ****

Shanbavirus-A1      PMITQAPPVVPKKAPHSTNFKYTTQRSLVCESIGCANMANTLYTGSNFSTAIAGERIMYA
Shanbavirus-A2      PTVTQAPEVKPSKSTHATNFKYTTTRTIVPESIGTANMANVLFTGSNYSLALAGERIMYA
                    * :**** * *.*:.*:******* *::* **** *****.*:****:* *:********

Shanbavirus-A1      RDSSGQTKPIRDFLEIAKIPGMWLTDTLNANQETIFDWPTTTTGGNNVFDKQIKFKDMGN
Shanbavirus-A2      RDSSGQTKPVRDFMELAKIPTMWMNDRLDLNASTLFEWSTSTNAGSSVFQANINFKDMGN
                    *********:***:*:**** **:.* *: * .*:*:*.*:*..*..**: :*:******

Shanbavirus-A1      LGIMMNFFSGWSGTIVLELTVFGSVMHKGKLAVIVNQSSDGTPTLDYMNRLQYSLLDVGL
Shanbavirus-A2      LGILSRFYVGWSGTIIVDLTVFGSVMHKGKLAVVINQATDDTANLDTMNRLQYTLLDIGL
                    ***: .*: ******:::***************::**::*.*..** ******:***:**

Shanbavirus-A1      NSSIQVPIPFMHDSWMRSTNPNEHLKIVVKVVSELMHNATASNSVRCMIRFKAGDDFKFY
Shanbavirus-A2      NSSVQVPVPYMNDSWMRSTNGDEHLRITVLVINELTYNATAATAIKCLLRFKAGDDFKFY
                    ***:***:*:*:******** :***:*.* *:.** :****:.:::*::***********

Shanbavirus-A1      FPKPSALATQISWGSEMDLRDPFTTDETVQEALSTNHATNDQDQAAAAGLADAGNAGTLD
Shanbavirus-A2      FPKPSTMAVQISWGSEMDLRDPFSTDDSVQEALTSNHSANEQSVAAATGLSDAGNAGTLS
                    *****::*.**************:**::*****::**::*:*. ***:**:********.

Shanbavirus-A1      DVVADPTPMCIGVKTNRMNIAPISYTDIFTYFGRSWLVNEQVYRENSNNIVVNVPAPRSG
Shanbavirus-A2      DIVTDPTPVCVGVKVNRMNISPISYTDVFSYFGRSWKVAERQYEAATSNLVIPVPAPSHG
                    *:*:****:*:***.*****:******:*:****** * *: *.  :.*:*: ****  *

Shanbavirus-A1      HAVLMNFFTFFAGEVNFTVCNDSENTIIVSHAYQDFVNDSSGAGSVAIPARTVSTFTAPF
Shanbavirus-A2      HAVLMNFFTFFAGEVNFTIANDSDNTLIVSHAYQEFNNDSSGAGAVAIPARSVATFTAPF
                    ******************:.***:**:*******:* *******:******:*:******

Shanbavirus-A1      YSVDPLRGLEDSKLFGVLHFNCGYLSGSFKVYASLRCPNFFVPRPFPKTANTRSVEEEPA
Shanbavirus-A2      YSVEPLRGLEDPKLFGSLHFNCGYLQGSFKVYASLRCPNFFLPRPFPKVANSRSLMEDPS
                    ***:*******.**** ********.***************:******.**:**: *:*:

Shanbavirus-A1      NYVISKVTRDAVIWHLTHKDEWQPLEIKEETVNPMLVSFMQELRRTGYRGTLLMQAGDVE
Shanbavirus-A2      RYVISRVTKEACLWHILNKDETEPLQITEKYVDPLLVSFTQELRRAGYRGDLLMQAGDVE
                    .****:**::* :**: :*** :**:*.*: *:*:**** *****:**** *********

Shanbavirus-A1      SNPGPVELVYRHRGLYKHYGVTDGTQVYHLNTDDILYSALTGKANTRCDPLDQSWIHTGR
Shanbavirus-A2      SNPGPVELVYRHRGLYKHYGVTDGS-----------------------------------
                    ************************:

Shanbavirus-A1      FADSFGYEIPVTMDFSIDANCETFAKQFVPDGMTQGQALKMWAAIVFTFSLGTCVTNQDY
Shanbavirus-A2      ------------------------------------------------------------
```

```
Shanbavirus-A1      TSLFSKLSTVIMDTMHAAVTSKLLRFMLRMLLYAIMFCHAPNLMTGGSMAALMFMDYQDL
Shanbavirus-A2      ------------------------------------------------------------

Shanbavirus-A1      VKERSPGWIKGLVKAMIDGDVQSVCVNLVEGMQDDASPEEMAETVKEAAKMTSQAPDFDG
Shanbavirus-A2      ------------------------------------------------------------

Shanbavirus-A1      FNKFSLAAKNFDWWLDMMKKIIEKVVDFFKPSTARKFAALVEQYRHQLAMLFTSVAQAAQ
Shanbavirus-A2      ------------------------------------------------------------

Shanbavirus-A1      EAKRPGATSSIVFAEKVDWLKRQVNHWNQGFVEFCPRHELAQTMAAAVRTMTAIDMAPCK
Shanbavirus-A2      ------------------------------------------------------------

Shanbavirus-A1      PSEVSRVEPVGVLIRGPPGQGKSFFSMVLIKEICRLKNWDTADVFQHPVGSKHMDGYQQQ
Shanbavirus-A2      ------------------------------------------------------------

Shanbavirus-A1      NIHLIDDLGQNADDSDYELLCQMISTIAFPVPMARLEEKATWYTSKLVIATTNRGDFNTK
Shanbavirus-A2      ------------------------------------------------------------

Shanbavirus-A1      TINTTGALERRFGFNFSIKAKQQYTKDGKLDVVKFTPEIQEGMTWTDEADRPINPFHLAR
Shanbavirus-A2      ------------------------------------------------------------

Shanbavirus-A1      EIVEELNRREQITKFWNNYMKPELNKKYPGVFCYPSAEALRESNKLLNNQNASLLDFESW
Shanbavirus-A2      ------------------------------------------------------------

Shanbavirus-A1      ESRLATLMAPIEEWLFSTDEEEALPPAPLNRFSRTVDEKIGKFGRWLAKHGHWISFGSAL
Shanbavirus-A2      ------------------------------------------------------------

Shanbavirus-A1      VGIVGIVTWWVCNGKTGFKGPDKKEEDDQVYGGEVTVKPKKTLFKKNASEPIRDQGPGDE
Shanbavirus-A2      ------------------------------------------------------------

Shanbavirus-A1      LTHITRGCVRLVADGIQVFGLAIGDDRILTFGHSDQVLFKAGTVEVVYGDIRMILESPEF
Shanbavirus-A2      ------------------------------------------------------------

Shanbavirus-A1      TRLTVGNAETDLAIVKTNAPFRMTSMVKHFTRSLGSDPILVWNTKNGIYTQSVSNLQALG
Shanbavirus-A2      ------------------------------------------------------------

Shanbavirus-A1      AATTAEGTWSHDCVSYNAHTGSGTCGGALCVKVGGMYKILGMHVAGNGFIGRAIMLPTNQ
Shanbavirus-A2      ------------------------------------------------------------

Shanbavirus-A1      GTYYPINPLPIPPANLVTKTRLKPSPLHGIVPVTKGPAVLTKNDPRTHGDPLDQIFLKNV
Shanbavirus-A2      ------------------------------------------------------------

Shanbavirus-A1      GNYFEVRDPNRFDEAVMNLRARLVDTIGVHSMATMEEALFDGANAVDMSTSPGHKYTSQN
Shanbavirus-A2      ------------------------------------------------------------
```

```
Shanbavirus-A1      LRKADLINKEERWISPILRADVDQLIEQAKVCTPLVYFTTALKDELRSEDKIKEAKTRVI
Shanbavirus-A2      ------------------------------------------------------------

Shanbavirus-A1      EASNFDYTVAFRMIFGKQVDIICATPSEDTGIAMGINPMVDWTSLVRSLYKNNLDFDYKA
Shanbavirus-A2      ------------------------------------------------------------

Shanbavirus-A1      FDGSLSTELMRAAGYVLAGCVEDENLFLNLLDASVISIHHGVFEDYMLVGSNPSGTPFTT
Shanbavirus-A2      ------------------------------------------------------------

Shanbavirus-A1      VLNCACNLLVVEYYMLGLAQQIPYVAITYGDDLILSTQEPIDARDFQRMLKLEFGMTVTP
Shanbavirus-A2      ------------------------------------------------------------

Shanbavirus-A1      SDKDSTEFVNKSPMEVEFLKRKPKHHTSDTIIGVLSLENMLQHIMWCKGLDEFATQTISF
Shanbavirus-A2      ------------------------------------------------------------

Shanbavirus-A1      QIELAAHGEATYNEVRELFKTRKIILPKFADAKFQLDKIVYQL
Shanbavirus-A2      -------------------------------------------
```

```
LOCUS       NC_032820               9550 bp    RNA     linear   VRL 13-AUG-2018
DEFINITION  Wenzhou picorna-like virus 48 strain beimix75770 hypothetical
            protein gene, complete cds.
ACCESSION   NC_032820
VERSION     NC_032820.1
DBLINK      BioProject: PRJNA485481
KEYWORDS    RefSeq.
SOURCE      Wenzhou picorna-like virus 48
  ORGANISM  Wenzhou picorna-like virus 48
            Viruses; Riboviria; unclassified Riboviria.
REFERENCE   1  (bases 1 to 9550)
  AUTHORS   Shi,M., Lin,X.D., Tian,J.H., Chen,L.J., Chen,X., Li,C.X., Qin,X.C.,
            Li,J., Cao,J.P., Eden,J.S., Buchmann,J., Wang,W., Xu,J.,
            Holmes,E.C. and Zhang,Y.Z.
  TITLE     Redefining the invertebrate RNA virosphere
  JOURNAL   Nature (2016) In press
   PUBMED   27880757
  REMARK    Publication Status: Available-Online prior to print
REFERENCE   2  (bases 1 to 9550)
  CONSRTM   NCBI Genome Project
  TITLE     Direct Submission
  JOURNAL   Submitted (17-JAN-2017) National Center for Biotechnology
            Information, NIH, Bethesda, MD 20894, USA
REFERENCE   3  (bases 1 to 9550)
  AUTHORS   Shi,M., Lin,X.D., Tian,J.H., Chen,L.J., Chen,X., Li,C.X., Qin,X.C.,
            Li,J., Cao,J.P., Eden,J.S., Buchmann,J.P., Wang,W., Xu,J.G.,
            Holmes,E.C. and Zhang,Y.Z.
  TITLE     Direct Submission
  JOURNAL   Submitted (16-SEP-2016) Department of Zoonoses, National Institute
            for Communicable Disease Control and Prevention, Chinese Center for
            Disease Control and Prevention, Changping Liuzi 5, Beijing, China
COMMENT     PROVISIONAL REFSEQ: This record has not yet been subject to final
            NCBI review. The reference sequence is identical to KX883301.

            ##Assembly-Data-START##
            Assembly Method       :: Trinityrnaseq v. 2.0.6
            Sequencing Technology :: Illumina
            ##Assembly-Data-END##
            COMPLETENESS: full length.
FEATURES             Location/Qualifiers
     source          1..9550
                     /organism="Wenzhou picorna-like virus 48"
                     /mol_type="genomic RNA"
                     /strain="beimix75770"
                     /host="Bivalva"
                     /db_xref="taxon:1923635"
                     /country="China"
                     /collection_date="2013"
     gene            58..9525
                     /locus_tag="BWS63_gp1"
                     /db_xref="GeneID:30853955"
     CDS             58..9525
                     /locus_tag="BWS63_gp1"
                     /codon_start=1
                     /product="hypothetical protein"
                     /protein_id="YP_009336671.1"
                     /db_xref="GeneID:30853955"
                     /translation="

MPKHSIKALAEQWILDANTPYQDDERETDNIQWTTVVGKNAARR
RRKSMVASQKQSTFKKEKYRLKVCKHGSACHFAKTGSCYFWHPSREEKVCKTLCSKVN
```

CKDTTCLYTHLPKLTHKKSKSVCWYGSRCLRQACPFMHVGDNVRAAVYNLPGAKAYTK
KCRYGGDCVNDMCFFAHSRKELRDSHRRKEFKEVPSLDLSSDSEMTKKEVPSLDKSSE
SEMTKLREKEVPPHASGKTTNLNIVNGFQCTAGQFISTAHNQTVEPDFVLDPDIAVYQ
KTGTPAPVRVIKHLTKGIIKVGNLVTTGSGYATPVVCSTGQAELKMFEGRTLLMYSFL
PTEMGTCGTPVYMDGQIIGLHVISNASGPIKSNFAIPVGSIDQRIVEREVQGQLQIVG
KLQSDVKREDSAPSQEPDVLSSLATLPSLITEVGGAVSGLSAAASGIAGIFDTPAIIP
PTPQQIYTSDLPREVYTVGFKKDDYVGDQSDCVSSASMMGTSFRERCMIPSRIGVNEW
TTTQVDGTLLASYPVSPMLSENFLTIGSYDYYDTTMLAGAAVNYTYWRGAIRFHIEAF
PTSFHQGQLYVCFNPNPQPDTINFRECWTLGGVSIDLSVSNSTTYEIPYVFPKDYCHV
HDVVHDEDFLNASNQTKNTCCTGYVYIFVQNRLIASSDKVPDRIDVNVSISGGADFDC
KVPTRIYHDQSYYARGVWQHYTIDPENVTPDDELEDDQGNSNNNSNREGSTFMKVKTN
VPAYYQAKTSEDVSVIGSVPTATTENIFQDYYLVDNGVVISKTQPRGTKVASCSLPGC
FFLVENAPSGIANYHALYRSDFQVKLKLSTTQFQCGCARLVFEPIQGILDNLSVPAGT
DLGTWASFNASSQLPHADINFNGQTECEFIIPWASVCRAIAKMDSAQQNPSEIGVISI
YVWNQLRTTGSASIYGSLWIKAIDPYFSVRRIAGVLQGEDPPASSEQRTVNVSALDNR
SSGVEKYYVNDHFSPIELMRRLDYRTSQLIECNEKRQSRVNPGMNPMDLYYAMKIPLD
GGFSNTFIESMYAFNSGSKRLTLITNLTPLHPVLFWVKYEYWESRVNQIFHSDFRYNA
ATAKTNPNVIRARRQRINSHFGQPCVVYQPAHTPIKTIEFPQYLGIPIIQSHGVVKSY
QYMVAEGEDRGCVHLSYQWNYIHKYRGMTFTVHAATSVGDDYMLYYPQPPPVIRQPKH
VANTFDFVEDA
GPSCYDRNNHCNILLSGDIEENPGP
KDVLMQFNEWGFQFHKPPTITY
SQMDGSWYCWIELFLGEERRMFYGVGPSKKIAKRNAVATIPDFSEQASHRSVDEHLVS
PDSTASGTKIQEQVLPNFGITDKIVGALSSGARSLFQLLGSALKETLWINLDEHKEFF
YDVLKDVAILVMFFDNVVLAHLQGKTIIYKLPLYIMALRKNPLVVHLFNLITRTGEQA
LTDGYVQKIFRSCIQALGRVIGCEFSDVTVNRAMYDFTRPSITEMGKKFYAYITNLLQ
RLMANEMPLDQAVATMTKIRTLYNNKREIFTTDLLFKNRLEFMEIYDQVKICQPILEK
YGKPGFNFFMKEVNERYTALAKIAIGSGPRCEPVGIMLSGAAGQGKSVFQTILARAMA
KYLLPEGERTSDAIDKNIYALRPGEIEYMDGYCMQDVVTVDDALADADYKTVIPFIQL
ISTAPTPVAMADLADKGRYFTSKALVISSNFETVQGYNEIKYPEAINRRFELCYHIVC
IKPRDNKLDIESLLSDFSNQGVKVWDVLDEHMAFHKYNFGETAYIKKFMKPSEVYREC
MNRLKTKINTFGNFESLYSRLDVEDFNNSAESRICDKCETIHDFGCPLDDEIESLYDV
TVLDQLHAAYQRGELEKVLADQNYKKVKVPLDISLMSDEESIYDQLTFAQITDVDVVC
TVYWCLLHKYKYGFKEGFKEIQKWICDTVSSFIVFIRKHLTKILTAALAIVSAIGAYY
ISKKVKLGERLTNLSLYFEEWYAQSQNKKRLGEIIASAPRHQYGAEMLKGLLNGEIKP
YKGKTEIPHWAFLEAEAMEECKEAFRKCNMEVDEEESDYWKRVISLLRRAGALDEQSL
KEGFRKPPKKIQRREHLADFECHGLTNNCVPLYSSPLEDTVVGYGFLVDNVNLLMPKH
YLSAYKYLYLSVGSDQTRQEIYLPNSRSIPGKDLMVVNVPPQQSVRKRVKMFLPKNTL
YSEYAGYGHRVVGKLSLTTGEEDSHGVLKLCEIREEGAVVGTEFMSLRVTKETRVGIC
GTPYMCGSFVMGLHVNGAGFDAGMAIVTREELEEVLFPGFVEYELPECEQ YVEENWTG
NNVGFKHLGAYYGEQGDLLSTNINMSTEKVHSDMYNPVDFPDEFDVSPKNYEVLFKRA
NKYGFHQPLGSHTAQETSFAFSLYETLLAGQDRDISLLTDEVILNGDHEIMPIKIDSS
AGHWSCISNKKKAFIDVQETPEGNWFTWSDDYYNKKHPVLGKSLFEVIQERINLGEKG
IRAESFWVTTLKDELLLKEKVEAGKTRVFEAPPLDTTILFKKYFGSFANWYRHNAGPV
LSHTIGVEKEKVWGSLYYHLKRNSDFGIAADYSQFDGTIPPSAFSFFQKVVRLYYEGA
PEEEHKVRDVLIHELQHTTQLIGSGLYQSGKGNKSGNYLTDVFNSITNVWAWMTSFHR
VFTNELGKMPTLDDWFENVVLFTHGDDCILSLKRVITPQKLLDEIRALGFLITSADKS
GEDIKYENVDGLTYLKSGFRRSQGIVWPPMPMATCYREVNWCKRSMRYNSTVRKTQIS
EGRRFAAYHGKEQLAAFDHAYRKNVHKEIGLLLDPVIPETYDSIEIDVRL
KQLMQEMCPDN
VFNASYLDCFISLLSCGDIESNPGP
LSKKFLLLRDILFEHDFCFSAVPFVIQSHY
YETVEDTNFWEWMKNHWWERLCYSWHPKEPLDDGETKEQVMLDAIEAIALGCWEHQVI
VVDERLDGRHTMLDTRFDYFTGVTAVIGPYRAISRASELMSLGEPLERQYPLFEYFVR
CNLRGIPPSVQHDTMFQHRDKWFRKDRSSFPLPPPK
PIQGLTQRFESTLLLGGDIEENPGP
FYFSLVEYPDEFVGTPPYYEKLFARAVDNKLHESLITLFFQFICFYFVFHAIGRR
IHGEEEWNRVINEACDCFYACLIYICSFFRSDHGRGT

ORIGIN

AAGTAGTATATCCATATTTTGTTTATTTATTTATGTAATTACATTTTATGAATCATCATG
CCTAAACATTCAATCAAAGCTTTAGCTGAACAGTGGATTTTAGATGCTAATACTCCATAT
CAAGATGATGAACGTGAGACCGATAATATACAGTGGACAACCGTTGTTGGAAAGAACGCC
GCCAGACGTAGGAGAAAATCTATGGTGGCAAGCCAGAAGCAATCGACCTTTAAGAAGGAG
AAATATCGTCTTAAGGTTTGTAAGCACGGCAGCGCGTGCCATTTCGCGAAAACTGGCAGC
TGTTACTTCTGGCATCCGTCGCGTGAAGAGAAGGTGTGTAAGACTTTATGTTCCAAGGTG
AATTGTAAAGATACAACTTGTTTGTATACTCATCTCCCAAAACTAACACATAAAAAATCT
AAATCAGTGTGTTGGTATGGTAGTAGGTGTCTTCGCCAGGCGTGTCCATTTATGCACGTA
GGTGATAACGTACGAGCAGCAGTTTACAATTTGCCCGGAGCCAAGGCTTATACTAAGAAG
TGTAGATATGGTGGAGACTGTGTCAATGATATGTGCTTCTTTGCTCATTCACGTAAGGAA
CTTCGGGATAGCCACCGTCGTAAGGAATTTAAGGAAGTCCCATCACTAGATTTATCTAGT
GATAGTGAGATGACCAAGAAGGAAGTCCCCTCACTGGATAAGTCTAGTGAGAGCGAGATG
ACCAAACTTCGAGAAAAGGAAGTCCCACCACATGCTAGTGGGAAGACTACTAATCTCAAT
ATTGTTAATGGATTTCAATGTACTGCAGGTCAATTTATATCGACCGCTCATAATCAAACT
GTTGAGCCGGACTTTGTCCTGGATCCAGATATAGCTGTATACCAAAAAACGGGTACACCA
GCACCCGTTAGAGTCATAAAACATTTGACAAAGGGAATTATTAAAGTAGGTAATTTAGTA
ACAACCGGTAGTGGTTATGCTACACCAGTTGTTTGTTCTACCGGGCAAGCTGAACTCAAA
ATGTTTGAAGGTAGGACTTTATTAATGTATTCATTTCTACCAACAGAAATGGGTACTTGT
GGTACTCCTGTATACATGGACGGCCAAATCATAGGCTTACATGTTATCTCTAATGCGTCT
GGTCCAATTAAATCCAATTTTGCTATCCCTGTGGGTAGCATAGACCAACGTATTGTCGAG
AGAGAAGTCCAAGGTCAATTACAAATTGTAGGCAAATTACAAAGCGATGTCAAACGTGAA
GACAGCGCACCATCTCAGGAACCAGATGTTTTGTCATCCCTCGCGACTTTACCTAGTCTT
ATTACTGAAGTAGGGGGAGCTGTGTCAGGTTTGTCTGCCGCGGCTAGTGGTATAGCTGGA
ATTTTTGATACACCCGCGATTATTCCACCCACACCCCAACAAATATATACTTCAGATTTA
CCACGCGAAGTTTACACCGTAGGTTTTAAAAAGGATGATTATGTTGGAGATCAGTCTGAT
TGTGTCTCCTCTGCATCCATGATGGGAACATCATTTCGTGAACGCTGTATGATACCATCC
CGCATTGGTGTCAATGAATGGACAACTACTCAAGTTGATGGGACATTGCTTGCATCTTAT
CCAGTGTCTCCAATGTTGTCCGAAAATTTTCTTACCATCGGTTCCTATGATTATTATGAT
ACCACCATGTTGGCAGGTGCTGCTGTCAACTATACTTATTGGAGAGGAGCTATAAGGTTT
CATATTGAAGCCTTTCCTACCTCTTTTCATCAAGGTCAATTATATGTATGTTTTAATCCC
AATCCTCAACCAGATACCATAAATTTTCGTGAGTGCTGGACACTCGGAGGGGTAAGCATA
GATCTTAGTGTTAGTAATTCAACTACATATGAGATTCCTTATGTTTTCCCTAAGGATTAT
TGTCATGTACATGATGTTGTTCATGATGAAGATTTCCTTAATGCGTCTAATCAAACCAAG
AATACGTGTTGTACTGGTTATGTCTATATATTTGTCCAAAATAGGCTCATAGCTAGCAGT
GACAAGGTACCTGATCGTATCGATGTCAATGTTAGTATTTCAGGTGGGGCAGACTTTGAT
TGTAAAGTCCCAACTAGGATTTATCATGACCAAAGTTATTATGCTAGGGGTGTATGGCAA
CATTACACTATAGACCCAGAAAATGTAACACCAGATGATGAATTAGAAGATGATCAGGGA
AATTCTAATAACAATAGCAATAGGGAGGGTTCAACCTTTATGAAGGTTAAGACAAATGTA
CCCGCGTATTATCAAGCAAAAACCAGTGAAGATGTATCTGTAATAGGTTCCGTTCCAACC
GCGACAACCGAAAACATATTTCAGGATTACTATTTGGTTGATAATGGTGTTGTCATCAGT
AAAACTCAACCAAGAGGAACTAAAGTTGCTTCATGTTCTTTGCCAGGATGTTTTTTCTTG
GTAGAGAATGCTCCTTCAGGTATAGCTAATTATCATGCACTTTATAGGTCAGATTTTCAA
GTTAAATTAAAATTATCTACTACTCAATTTCAATGTGGTTGTGCTCGTTTAGTTTTTGAA
CCCATTCAAGGTATTTTAGACAACCTCTCAGTTCCTGCCGGCACAGATTTGGGTACTTGG
GCTTCATTTAATGCCTCCTCGCAATTGCCACATGCTGATATCAATTTTAATGGACAAACA
GAATGTGAGTTCATTATCCCATGGGCATCAGTATGCAGAGCAATCGCCAAGATGGATTCT
GCACAACAAAATCCATCTGAGATAGGAGTTATTAGTATATATGTATGGAATCAATTACGC
ACTACAGGTAGTGCTTCTATCTATGGTTCCTTGTGGATCAAGGCCATAGATCCTTATTTC
TCTGTGAGAAGAATAGCAGGAGTTTTACAAGGAGAAGATCCACCAGCTTCTTCTGAACAA
CGTACTGTTAATGTTTCAGCTCTTGACAACCGATCATCAGGAGTTGAGAAATATTATGTC
AATGATCATTTCTCTCCCATTGAATTAATGCGAAGGCTGGACTATCGCACTTCACAACTT
ATTGAATGTAATGAAAAAAGACAAAGTAGGGTCAATCCTGGTATGAATCCTATGGATTTA
TACTATGCCATGAAAATTCCACTTGATGGGGGATTTTCCAATACTTTCATTGAATCTATG
TATGCTTTTAACAGTGGTAGTAAGAGACTCACTCTTATTACCAACCTCACCCCGCTTCAT
CCAGTTCTTTTTTGGGTGAAGTACGAATATTGGGAATCACGCGTCAATCAAATCTTTCAT
AGTGATTTTAGATACAATGCCGCAACAGCAAAGACCAATCCAAATGTTATTAGAGCTCGA
CGTCAAAGAATTAATTCTCATTTCGGCCAGCCGTGTGTAGTTTATCAACCAGCACACACG
CCTATTAAAACAATAGAATTTCCTCAGTATCTTGGCATACCAATTATTCAATCACATGGT
GTTGTCAAGAGTTATCAATATATGGTTGCGGAAGGGGAAGACAGGGGTTGTGTTCATCTC

AGTTATCAGTGGAATTACATTCATAAGTATAGGGGCATGACCTTCACCGTTCATGCAGCA
ACTTCAGTAGGGGATGATTATATGCTCTACTATCCTCAGCCGCCACCTGTTATTAGACAA
CCTAAACATGTGGCAAACACTTTTGATTTCGTGGAAGATGCTGGTCCATCTTGCTACGAC
AGGAATAATCATTGCAACATCTTACTTTCAGGAGATATTGAAGAGAATCCAGGTCCTAAA
GATGTCCTCATGCAATTCAATGAGTGGGGATTCCAGTTTCATAAACCACCAACTATCACT
TATTCCCAAATGGATGGGTCTTGGTATTGTTGGATAGAACTTTTTCTCGGGGAGGAACGC
AGAATGTTTTATGGGGTAGGTCCATCAAAGAAAATTGCTAAGCGGAATGCCGTCGCTACT
ATCCCGGATTTTAGTGAACAAGCGTCTCATCGATCCGTCGATGAACACTTGGTCTCACCC
GATTCAACGGCCTCAGGGACTAAAATCCAGGAACAAGTACTACCAAATTTCGGAATTACA
GACAAAATTGTGGGGGCTTTATCTTCAGGTGCTCGTTCACTTTTTCAACTTTTGGGATCA
GCTCTAAAAGAAACTCTTTGGATTAATCTTGATGAACATAAGGAATTTTTTTATGATGTC
CTTAAAGATGTTGCTATTCTTGTTATGTTTTTTGACAATGTTGTTTTGGCTCATTTGCAA
GGGAAAACCATTATTTACAAATTACCTCTTTATATTATGGCACTTAGGAAAAACCCTCTT
GTTGTTCACTTATTCAATCTTATTACTCGTACAGGTGAGCAGGCTCTCACCGATGGATAT
GTTCAAAAAATTTTTAGATCATGTATTCAAGCTTTAGGACGTGTCATAGGTTGTGAATTT
TCAGATGTTACTGTCAATCGTGCTATGTATGATTTCACCAGGCCCTCCATTACTGAAATG
GGGAAAAAAATTTTATGCTTATATTACCAATCTACTTCAACGTCTCATGGCAAATGAAATG
CCATTAGATCAAGCCGTGGCTACAATGACCAAAATTCGGACATTGTACAATAACAAACGC
GAAATTTTTACCACCGATCTTCTTTTCAAAAACCGTTTGGAATTCATGGAAATTTACGAT
CAAGTCAAAATTTGTCAACCCATTTTAGAAAAATATGGAAAACCAGGATTTAATTTCTTT
ATGAAGGAAGTTAATGAAAGGTACACTGCTCTCGCGAAAATTGCAATAGGATCAGGGCCA
AGATGTGAACCCGTCGGCATTATGTTATCAGGTGCCGCCGGACAAGGTAAGTCAGTCTTT
CAAACAATCCTAGCTAGGGCCATGGCAAAATATCTCCTTCCGGAAGGAGAAAGAACTTCA
GATGCAATTGACAAAAATATTTATGCTTTACGTCCAGGTGAAATTGAATACATGGACGGA
TATTGTATGCAAGATGTTGTAACAGTAGATGATGCGTTAGCTGATGCTGATTACAAAACA
GTTATTCCTTTCATTCAACTTATTTCCACAGCACCAACTCCTGTTGCTATGGCAGATTTG
GCAGATAAGGGTAGGTATTTTACATCAAAAGCTCTTGTTATTTCCTCAAATTTTGAAACT
GTTCAGGGTTACAATGAAATTAAATATCCTGAAGCAATCAACAGGCGTTTTGAACTTTGT
TATCATATTGTTTGCATCAAACCTAGGGACAACAAATTAGATATTGAATCTCTACTCAGT
GATTTTTCAAATCAAGGTGTTAAGGTGTGGGATGTTCTTGATGAACACATGGCATTCCAC
AAATACAATTTTGGAGAAACTGCCTATATCAAAAAATTCATGAAACCATCAGAAGTTTAT
CGCGAATGTATGAACCGTCTCAAAACAAAAATCAACACCTTTGGAAATTTTGAATCATTG
TACTCCCGTCTTGATGTCGAAGATTTCAACAACTCAGCAGAAAGTAGAATCTGTGATAAA
TGTGAAACCATTCATGATTTTGGCTGTCCCTTAGATGATGAAATTGAAAGTCTCTATGAT
GTTACCGTTTTAGATCAATTACATGCTGCATATCAGAGGGGGGAATTAGAAAAAGTTTTG
GCAGATCAAAATTACAAAAAAGTTAAGGTTCCTTTAGATATTTCACTTATGTCCGATGAA
GAATCTATTTATGATCAACTTACATTTGCTCAAATTACTGATGTTGATGTTGTTTGTACT
GTTTACTGGTGTCTTCTTCACAAATACAAATATGGTTTTAAGGAAGGTTTCAAAGAAATT
CAAAAGTGGATTTGTGATACAGTCAGTTCTTTTATTGTTTTTATTAGGAAACATCTTACC
AAAATTCTTACAGCAGCCCTTGCCATTGTTTCAGCCATTGGAGCATACTACATATCCAAA
AAAGTCAAATTAGGGGAACGTTTAACCAATCTCTCTCTCTATTTCGAAGAATGGTATGCT
CAAAGTCAAAACAAAAAACGTTTAGGGGAAATTATAGCTTCGGCTCCTCGTCATCAATAT
GGGGCCGAAATGCTAAAGGGGCTACTCAACGGGGAAATTAAGCCATACAAAGGGAAAACC
GAAATACCACATTGGGCCTTTTTAGAAGCTGAAGCCATGGAAGAATGTAAGGAAGCCTTT
CGAAAATGCAATATGGAAGTTGATGAAGAAGAATCAGATTATTGGAAACGCGTTATCTCA
TTACTCCGTCGCGCTGGGGCTCTCGATGAGCAATCACTCAAAGAGGGTTTCAGAAAACCA
CCCAAGAAAATTCAACGTAGGGAACATCTTGCTGATTTTGAATGTCATGGTCTTACTAAT
AATTGTGTTCCTCTTTATTCTAGTCCTCTTGAAGATACTGTTGTAGGATATGGTTTTCTT
GTTGATAATGTCAATCTTCTCATGCCAAAACATTATTTGTCAGCTTATAAATATTTATAT
TTGTCAGTAGGGTCCGATCAAACTAGGCAGGAAATTTACTTACCAAATTCACGTTCTATA
CCAGGGAAAGATCTTATGGTAGTCAATGTTCCACCTCAACAATCTGTTAGGAAAAGAGTC
AAAATGTTCTTGCCAAAAAATACGCTTTACTCGGAATATGCCGGGTACGGTCATCGCGTC
GTAGGGAAACTTAGTCTCACCACCGGAGAAGAAGATTCACACGGAGTCCTTAAACTGTGT
GAAATACGTGAAGAAGGTGCTGTCGTAGGTACCGAATTCATGTCTCTACGAGTTACCAAA
GAAACGCGTGTAGGAATTTGCGGGACACCATACATGTGCGGGTCATTTGTTATGGGTCTT
CATGTAAACGGAGCTGGCTTCGATGCAGGAATGGCCATTGTTACACGCGAAGAACTAGAA
GAAGTACTCTTTCCCGGTTTTGTGGAATATGAACTACCAGAATGTGAACAATATGTTGAA
GAAAATTGGACAGGTAATAATGTAGGATTCAAACATTTGGGGGCTTACTACGGGGAACAG
GGTGATTTACTTTCTACCAATATCAATATGTCTACAGAGAAAGTTCATTCAGATATGTAC
AATCCAGTTGATTTTCCAGATGAATTTGATGTTTCTCCCAAAAATTATGAAGTTCTTTTT
AAACGTGCAAACAAATATGGATTTCATCAACCTTTAGGATCTCATACTGCTCAGGAAACA

TCATTTGCCTTTAGTTTATATGAAACATTACTTGCAGGTCAAGATAGGGATATTAGTTTA
CTTACTGATGAAGTCATTCTTAATGGTGATCATGAAATTATGCCAATTAAAATTGATTCC
AGTGCTGGTCACTGGTCATGTATTTCCAACAAAAAGAAAGCTTTTATTGATGTTCAGGAA
ACTCCCGAAGGTAACTGGTTTACCTGGTCAGATGATTATTACAATAAAAAACATCCAGTT
TTAGGCAAATCATTATTTGAAGTCATTCAAGAAAGAATCAATTTAGGTGAAAAAGGTATT
AGGGCAGAAAGTTTTTGGGTCACTACTCTAAAGGATGAACTCCTTCTTAAAGAAAAAGTT
GAAGCAGGTAAGACTCGTGTTTTTGAAGCTCCTCCATTAGATACAACCATATTATTCAAA
AAATATTTTGGCAGTTTTGCAAATTGGTACCGTCACAATGCGGGACCAGTTCTTTCTCAT
ACAATAGGGGTAGAAAAAGAAAAAGTATGGGGTAGTTTATATTATCATCTTAAACGAAAT
TCAGATTTTGGTATTGCAGCAGATTACAGTCAATTTGACGGCACAATCCCGCCGTCAGCT
TTCAGTTTCTTTCAAAAAGTCGTTAGGTTATATTATGAAGGGGCTCCAGAAGAAGAACAC
AAAGTTAGGGATGTTCTTATTCATGAACTTCAACATACTACTCAACTAATAGGGTCAGGT
CTATATCAATCAGGTAAGGGAAATAAATCAGGAAATTATCTTACAGATGTTTTTAATTCT
ATTACAAATGTTTGGGCTTGGATGACATCTTTTCACCGTGTTTTTACAAATGAATTAGGG
AAAATGCCAACTCTAGATGATTGGTTTGAAAATGTTGTTCTTTTTACTCATGGGGACGAT
TGCATTTTATCTCTCAAACGTGTCATCACACCTCAAAAATTATTAGATGAAATTAGGGCT
TTAGGTTTTTTAATTACATCAGCAGATAAGTCAGGGGAAGACATTAAATACGAAAACGTG
GACGGGCTCACTTATCTCAAAAGTGGGTTTCGTCGATCTCAGGGAATCGTATGGCCTCCT
ATGCCCATGGCAACCTGTTACCGGGAAGTCAATTGGTGTAAGAGGAGCATGCGTTACAAC
TCTACTGTTAGGAAGACACAAATCTCCGAGGGCAGGAGGTTTGCGGCTTATCATGGAAAA
GAACAACTAGCAGCTTTCGACCACGCGTATAGGAAAAATGTACATAAGGAAATAGGTTTA
CTTTTAGATCCAGTCATACCCGAAACTTATGATTCTATTGAAATTGATGTTAGGTTAAAA
CAATTAATGCAAGAAATGTGTCCAGACAATGTTTTTAATGCCAGTTATTTAGATTGTTTC
ATCAGCTTATTATCTTGTGGGGATATCGAGTCAAATCCAGGTCCTTTATCAAAGAAATTT
TTATTACTTAGGGATATTTTATTTGAACATGATTTTTGTTTTTCCGCAGTTCCATTTGTA
ATTCAAAGTCATTATTATGAAACAGTAGAAGATACTAATTTTTGGGAATGGATGAAAAAT
CATTGGTGGGAACGTTTATGTTATTCTTGGCATCCTAAGGAACCTTTAGATGATGGGGAA
ACAAAAGAACAAGTTATGTTAGATGCAATTGAAGCAATAGCTTTAGGTTGTTGGGAACAT
CAAGTCATTGTCGTCGATGAACGTTTAGACGGCAGACATACTATGTTAGATACTCGTTTT
GATTACTTTACTGGGGTAACTGCAGTTATAGGACCATATAGGGCAATCTCTCGCGCTTCT
GAACTTATGAGTTTAGGAGAACCTTTAGAACGTCAATATCCTCTTTTTGAATATTTTGTT
AGGTGTAATTTACGTGGTATTCCTCCTTCAGTTCAACACGATACCATGTTCCAGCATAGG
GATAAATGGTTTAGGAAAGATAGGTCCTCATTTCCTTTACCACCACCTAAACCAATACAA
GGTCTTACACAACGTTTTGAGTCAACTCTTCTTTTGGGGGGCGATATTGAAGAAAATCCA
GGTCCTTTTTATTTTTCTTTAGTTGAATACCCTGATGAATTTGTAGGTACTCCACCATAT
TATGAAAAATTATTTGCTAGAGCTGTTGACAATAAATTACATGAATCTTTAATTACTTTA
TTTTTTCAATTTATATGTTTTTATTTTGTTTTTCATGCTATAGGTCGCCGCATTCATGGA
GAAGAAGAATGGAATCGTGTAATCAACGAAGCCTGTGATTGTTTTTATGCATGTTTAATA
TATATATGTTCATTTTTCAGATCAGATCACGGACGCGGTACATAAAGAGCACGGGTGGGT
GATGGGAGAT

```
LOCUS       MG600066                7781 bp    RNA     linear   VRL 08-NOV-2018
DEFINITION  Wuhan carp picornavirus strain DSYC18088 polyprotein gene, complete
            cds.
ACCESSION   MG600066
VERSION     MG600066.1
KEYWORDS    .
SOURCE      Wuhan carp picornavirus
  ORGANISM  Wuhan carp picornavirus
            Viruses; Riboviria; Orthornavirae; Pisuviricota; Pisoniviricetes;
            Picornavirales; unclassified Picornavirales.
REFERENCE   1  (bases 1 to 7781)
  AUTHORS   Shi,M., Lin,X.D., Chen,X., Tian,J.H., Chen,L.J., Li,K., Wang,W.,
            Eden,J.S., Shen,J.J., Liu,L., Holmes,E.C. and Zhang,Y.Z.
  TITLE     The evolutionary history of vertebrate RNA viruses
  JOURNAL   Nature 556 (7700), 197-202 (2018)
   PUBMED   29618816
  REMARK    Erratum:[Nature. 2018 Jun 26;:. PMID: 29946168]
REFERENCE   2  (bases 1 to 7781)
  AUTHORS   Shi,M., Lin,X.-D., Chen,X., Tian,J.-H., Chen,L.-J., Li,K., Wang,W.,
            Eden,J.-S., Shen,J.-J., Liu,L., Holmes,E.C. and Zhuang,Y.-Z.
  TITLE     Direct Submission
  JOURNAL   Submitted (01-DEC-2017) School of Life and Environmental Sciences,
            The University of Sydney, Sydney, NSW 2006, Australia
COMMENT     ##Assembly-Data-START##
            Assembly Method       :: Trinity v. 2.1.1
            Sequencing Technology :: Sanger dideoxy sequencing; Illumina
            ##Assembly-Data-END##
FEATURES             Location/Qualifiers
     source          1..7781
                     /organism="Wuhan carp picornavirus"
                     /mol_type="genomic RNA"
                     /strain="DSYC18088"
                     /host="Carassius auratus"
                     /db_xref="taxon:2116199"
                     /country="China"
     CDS             248..7603
                     /codon_start=1
                     /product="polyprotein"
                     /protein_id="AVM87438.1"
                     /translation="

MDALLCYPEMILMLFGGALIAFPLLGDSLSAGIRCHSINKPVET
HMDTIKSIVSGASKVTNLLGAAGTEAAENASDRVGAVASTNASLLSQATTRTKYGFYP
SKDCKDHFFSQAMKTGMSETNKQKMIELASADWIDANVEGTILATIKLPEAFYSAKEK
PAHGPTRPFGSIRTNFTIEVQANVSTGSIGSLGVVYVPPGVQMLGPDVTRNLRTWRLH
PSDELNIGVNTSAKLYIPYTHYQNYVDTDSDQLGQVVVFVWARLTSAPGTTASGIAIF
GAMDQTDLQKPRPQAPERVRIREGPGSMNLANCKETCQAESLALAGEAVAVDPTTSGS
SRAIRNLKHILQVYAATGNDQTNLNFTTAWEASAIRGTSLASYAVSGTNLSGISNIFS
NAFRFWRGIVVFRVTVFNSTLHKGRLKVAFYPGDVAAYTAENSQNAIYSILDIGLNPS
IELVVPYMNNTWLKDTGDFFGRLTVFVSSRLSAANNAAPRVRFIVEARYGDDVEFLVP
YDRGLNFQMRDNGDETDYTGWGTNTDVQVTDSVVDAGTTAAASETGLAAPSQEVNKPG
EGDPIKNPISLAAITKKVRIARADHLSTSILFGRASYAGRYTHSSSSIIATAVPEPSS
GVASLLKLFAYFNCDLTLHIHNQSTGVLACAHSYVVNDLQATTQNMATMGTIMIPPNEN
GSFTVPFYNITPARLLAGDHPFGYLYMYSFASSVIDLWISFKTISLFQPIAVPRWTTV
RAFLTRNREAEWEHPQQDVIDNLVRLKMWDATVNSCPRRALRARITGPNTMGYAA
MKEDEAGGWKEDLTEDGDVESNPGP
FKNMMIPPEKPIGDVIQAVVGSEVPANTQTISIKILMDRLGRAHVQINCL
EQAIPETTWRRDLTQSGDVESNPGP
SRSLVIPPTMTIGEIIAAVLGPNDTSAETQTLSIKILIDRLGRSHVQIFGF
PGAIPASVWVHDLTTDGDVESNPGP
T
```

CELVYIQRGLYKHYGVKYGQKVFHMGSENILGAAMMGKVRIECSDHGPQWITERSVDI
TQLRLHSIEKSAGMISYFSASNNCETYAYEALGITGFTQARALCVIGLIILGATAISA
LPEGESEQTVFKQFTTKMKNTGQKCWSKVSTFFSETMFDAIKCDVTKTIFKLVLRTVC
YGILFCSCPNLLTAAAVGSLVAMDIASIEGLSHTVKDLCSALLDGDLVKCVEAMSDLI
HENREDRQTLVRDCVRELRMIMKDVGETDVLHAKPEGPNDPFRLFNSGTSAAKNVEWW
ISLLSRFLTWIKSFFCENKTDMAIKYLKENDEIVMQIMCDADTLLVEGREGKNLRRKD
FQEDVDHTLQKLTKLKFICLTAHAMDVVGQINLLLGKLQRMPKPVANDGAIFRMEPIG
VWVSGDPGCGKSSFTYTLLTELRAWLKERTKIPDSGVYTTAAGSDHMDGYEGQWMHVI
DDMAQNKEEEDVKFLCQMISSVPWTTPQADLPSKGTQYSSQIVIATTNRTNFESCVLY
DSSALRRRFPFTFTLKPAANFRNDDGKLDITKATESGAAKNGAAWDIAKGVPGTDFRV
GNMKQIVHSIGCAYLERLKVSTLLTKQYEPLKGVTGMRQMTVDWAEIMDEIKKKKPEI
TPLKQAKPEGNFDAEEFCEWVEATDIEAKCREVVETGVLSEEMKERLEQSMAYLMSTS
YDTPFESLIETPRTRIGKISDRLKKWWGTRMEKIGIWWTKYKPFFIAAGILGSVISTC
VGIYMLVKTFSHDPIKEFFNPLPAGDPERAYNGPTMNTAKANKTQRIKIHPDGPNPAE
YSHLYKAAAALQIGGKNCYAIVVKPHTVMTYKHYWFRCGGKIEALHWNGLKHCPEPCT
VHVTDVQLENDDGAEIVSDYIFVTFERCPFQMKTIQKFHSPPEWGREGVALMAHPAGN
YSQHVDDIQASGAYVVGFDDGDQVCSDSVIYRTKSFAGMCGAMICQKVQGAWKVVAMH
HAGNRHTFGFGCRIRIPELPD GVVTEKIPAAKPHYTPTKSKLEKSPLFGIVAPEMQPA
PLSAKDKRLEVEVDNLVKFASDKYRVNVYEPDKTLMNAVAMYTAKQIFAATGRCGMWT
LEEALEGGIGNPIDMRTSPGEKYVKLNMRKKDLFKRNNDGTWWVFPGFREDVEEQLRL
AGAGEAHTVFAATLKDELRANEKVRQGKSRCIEACNVDFTVAHRMIYGPLYEKIYSST
PLQTGLAVGCNPYTDFHGIASAMKEHWFAIDYSRFDGSLSKELMQQAAEILVACTENC
ELARNILKPVIDSTHLVADEVWMVSGGMPSGSPCTTVLNSLCNLLVVRYAMATVGFSF
EEVILTTYGDDVIGSATQRVSAESVVRTIKDVFGMEATSADKKSLDLTVHPNEATFLK
RRFRHFPGTRFVTGQLDLDSMLQKIQWCHGLEEFKQQFESFTQELVLHGEETYAKVTQ
ACAPILDKYRILIPTYAQRYVEVYDMLFN

ORIGIN

TGCTCTTCCGATCTCATGAAGATACGGCATGGTAAACTTCCGGCGTAAAACTAATGTCAA
GCCACACTGTTATGTTATTCTACTTTTGACTTATATATAGAGCTTCTGCAGTAACTTCCT
AGCGTCACTCTCTCGCAACTCAGATTCCGCCGAAATGATTCCTTAACCGGTACATAGTAG
GAGGCGTCTGGGGTGAGAGTTCCTGTGACAGGAGTGTGTACCTGGAGAGGTGGTATGGAT
TAAGCGGATGGACGCACTTTTGTGCTATCCTGAGATGATCCTAATGCTATTTGGGGGGGC
CCTTATAGCTTTTCCACTACTGGGTGATAGCCTATCGGCAGGCATCCGGTGCCACTCTAT
AAACAAGCCTGTTGAAACCCACATGGACACAATCAAATCAATCGTAAGTGGAGCATCTAA
GGTTACTAACCTTCTTGGCGCAGCAGGAACAGAAGCAGCTGAAAACGCCTCAGACAGAGT
TGGAGCAGTGGCTTCAACCAATGCGTCTTTGCTATCACAAGCCACAACAAGAACAAAATA
TGGCTTTTATCCATCTAAAGATTGCAAAGACCATTTCTTTTCACAAGCCATGAAAACTGG
CATGAGCGAGACCAACAAACAGAAAATGATTGAGTTGGCCTCTGCTGATTGGATTGATGC
AAACGTGGAAGGCACAATTTTGGCCACAATCAAATTACCAGAAGCCTTCTACAGTGCCAA
AGAGAAACCCGCTCATGGACCCACTCGACCTTTTGGATCAATTAGAACAAATTTCACAAT
TGAGGTCCAGGCCAATGTTTCAACAGGAAGCATAGGTTCTCTTGGAGTTGTTTATGTGCC
ACCTGGAGTACAAATGTTGGGTCCAGACGTCACTCGAAACCTCAGAACGTGGCGATTACA
TCCTAGTGATGAGCTCAACATCGGTGTCAACACATCTGCTAAATTGTATATACCCTACAC
ACATTATCAAAATTATGTTGACACCGATTCTGACCAGCTCGGTCAAGTTGTGGTATTCGT
GTGGGCACGTTTAACGTCAGCCCCTGGCACCACAGCTTCTGGCATTGCCATTTTTGGAGC
AATGGACCAAACAGACCTTCAAAAACCTCGGCCACAAGCACCAGAGCGAGTTCGAATCAG
AGAGGGCCCTGGGTCCATGAACCTGGCGAATTGTAAAGAAACCTGCCAGGCTGAGAGTCT
AGCTCTCGCTGGAGAAGCGGTTGCTGTGGATCCCACAACGAGTGGATCAAGCAGGGCTAT
CAGGAACTTAAAGCACATCCTGCAAGTTTATGCCGCCACTGGAAACGATCAGACCAATCT
GAATTTCACAACAGCATGGGAAGCATCAGCCATCAGAGGCACTAGCCTCGCTTCATATGC
TGTCTCTGGCACAAATTTGTCAGGTATATCAAATATATTCTCAAACGCTTTTCGGTTCTG
GAGAGGTATCGTAGTTTTCAGAGTTACAGTATTTAATTCGACACTTCATAAAGGTCGCCT
CAAGGTGGCTTTTTACCCTGGAGACGTTGCCGCTTACACAGCCGAGAACAGCCAGAATGC
CATCTACAGCATCTTGGACATTGGCCTGAATCCCAGCATTGAACTCGTCGTACCCTACAT
GAACAACACCTGGCTCAAGGACACTGGCGACTTCTTCGGCAGGCTCACAGTTTTTGTGTC
GTCACGCTTATCAGCAGCCAACAATGCTGCTCCGAGAGTTCGCTTCATTGTTGAAGCGCG
TTATGGTGACGATGTGGAGTTCCTTGTCCCCTATGACCGTGGACTTAATTTCCAGATGCG
CGACAATGGTGATGAAACCGATTACACAGGTTGGGGAACCAACACAGACGTTCAGGTCAC
CGATTCGGTTGTGGATGCAGGAACAACAGCTGCTGCCAGCGAGACGGGTTTGGCAGCCCC

AAGCCAAGAGGTTAACAAACCTGGTGAGGGAGACCCAATTAAAAATCCAATCTCTCTCGC
TGCGATCACCAAGAAAGTCAGAATTGCCCGGGCAGACCACCTTAGCACTTCCATCCTGTT
TGGCCGTGCATCCTACGCAGGGCGATACACTCACTCATCAAGTTCAATCATCGCCACGGC
CGTGCCTGAACCATCCTCTGGTGTCGCATCATTGCTGAAGCTGTTCGCTTACTTTAACTG
TGACCTCACGCTCCACATTCACAATCAGAGCACTGGGGTTCTCGCCTGTGCCCATTCGTA
TGTCAACGATCTGCAAGCCACTACACAAAACATGGCCACCATGGGCACCATTATGATCCC
ACCGAATGAGAACGGTTCTTTCACAGTTCCTTTTTACAACATCACTCCCGCCCGGCTCTT
GGCTGGAGACCACCCGTTTGGCTATCTTTACATGTACAGTTTCGCTTCGTCGGTAATAGA
TTTGTGGATCTCATTCAAAACTATTAGCCTCTTCCAACCGATTGCAGTGCCCAGATGGAC
GACAGTGCGAGCTTTTCTCACCAGAAATAGAGAAGCAGAATGGGAACATCCGCAGCAGGA
CGTTATTGACAACCTTGTCCGCCTTAAGATGTGGGATGCTACAGTCAACAGCTGTCCCCG
CAGAGCGCTCAGAGCGCGCATCACTGGCCCTAACACCATGGGCTATGCTGCCATGAAGGA
GGATGAAGCTGGTGGGTGGAAAGAAGACCTCACTGAGGACGGTGATGTGGAGAGTAACCC
TGGACCGTTTAAGAACATGATGATCCCTCCAGAAAAACCAATAGGAGATGTCATACAAGC
AGTGGTTGGTTCTGAAGTTCCCGCAAACACTCAGACAATTAGTATTAAGATCCTAATGGA
CCGTCTGGGTCGTGCTCACGTGCAGATCAATTGCTTGGAGCAAGCAATTCCTGAAACCAC
ATGGCGTCGAGATCTCACACAATCTGGTGATGTGGAATCCAATCCAGGCCCCTCACGAAG
CCTTGTTATACCACCAACTATGACCATTGGAGAAATCATTGCTGCTGTGCTTGGTCCTAA
TGACACATCAGCTGAAACCCAGACACTTAGTATTAAAATTTTGATAGATCGATTGGGTCG
TTCTCACGTCCAGATATTCGGTTTCCCTGGTGCCATACCAGCAAGTGTCTGGGTTCATGA
CCTCACAACAGATGGTGATGTTGAATCCAATCCTGGGCCTACCTGTGAATTAGTTTATAT
ACAGCGTGGCTTGTACAAGCATTATGGTGTTAAGTATGGACAAAAAGTGTTCCATATGGG
CTCAGAAAATATTCTTGGGGCCGCCATGATGGGTAAAGTCAGAATTGAGTGTTCAGACCA
TGGCCCACAGTGGATCACAGAGAGGTCTGTTGACATCACACAGCTCCGCCTCCACAGCAT
TGAAAAATCAGCTGGTATGATCTCGTACTTCTCTGCAAGTAACAACTGTGAGACGTACGC
TTATGAGGCGCTTGGCATAACAGGTTTCACACAAGCCAGGGCTCTCTGCGTGATAGGCTT
AATTATTCTGGGGGCCACTGCTATTAGTGCACTCCCAGAAGGAGAGTCGGAACAAACAGT
CTTCAAACAGTTCACCACCAAAATGAAGAACACCGGACAAAAGTGTTGGAGCAAAGTGTC
CACTTTCTTCTCTGAGACAATGTTTGATGCAATCAAGTGTGATGTCACCAAGACGATTTT
CAAATTGGTCCTGAGAACCGTCTGCTACGGTATCCTGTTTTGCAGCTGTCCTAATCTGTT
GACTGCTGCAGCCGTTGGGTCCCTTGTTGCTATGGATATAGCAAGTATTGAAGGATTGTC
ACACACCGTTAAAGATCTCTGCAGTGCATTACTGGACGGTGACTTAGTTAAGTGTGTCGA
GGCAATGTCTGATTTGATACATGAAAACAGAGAGGACAGACAAACCCTTGTGCGTGACTG
TGTGCGTGAACTTCGCATGATCATGAAGGATGTTGGAGAGACAGATGTCCTGCACGCCAA
ACCCGAAGGCCCAAATGATCCATTTAGACTATTTAATTCTGGAACCTCAGCCGCTAAGAA
CGTTGAGTGGTGGATATCACTCCTCTCTCGTTTTCTGACCTGGATTAAGAGTTTTTTCTG
TGAAAACAAAACAGATATGGCCATCAAATACCTCAAAGAAAACGATGAGATCGTCATGCA
GATCATGTGTGATGCTGACACCCTGCTGGTGGAAGGGCGTGAAGGTAAAAATCTCAGGCG
TAAAGATTTCCAGGAGGATGTTGACCACACACTCCAAAAATTGACCAAACTCAAATTTAT
TTGTTTAACAGCTCACGCTATGGACGTGGTTGGTCAGATCAACCTTCTCCTTGGGAAATT
ACAACGCATGCCTAAGCCAGTGGCTAATGATGGGGCCATATTCCGGATGGAACCAATAGG
AGTGTGGGTTTCAGGAGATCCTGGATGTGGAAAGTCTAGCTTCACTTACACCCTCCTCAC
AGAACTGCGCGCCTGGCTCAAAGAAAGGACTAAGATCCCAGATTCAGGGGTGTACACCAC
TGCTGCCGGTTCAGATCACATGGACGGTTATGAAGGGCAGTGGATGCATGTTATAGATGA
CATGGCCCAAAATAAAGAGGAAGAAGATGTTAAGTTTCTATGCCAAATGATCTCCTCTGT
GCCATGGACAACGCCCCAAGCTGACCTCCCGAGCAAGGGTACCCAGTATTCATCTCAAAT
TGTGATAGCAACAACAAACAGGACCAATTTTGAATCATGTGTCCTGTATGATAGCAGTGC
TCTTAGACGCCGCTTCCCATTTACATTTACTCTCAAGCCCGCCGCAAACTTCAGAAACGA
CGACGGCAAGCTTGACATTACAAAAGCGACTGAGTCAGGAGCTGCAAAGAATGGGGCCGC
CTGGGACATTGCAAAAGGTGTGCCTGGCACTGACTTCAGAGTTGGCAACATGAAGCAGAT
TGTTCACTCGATTGGCTGTGCTTACCTGGAGCGTTTAAAAGTTTCCACTCTCCTAACCAA
ACAGTACGAACCCCTTAAGGGTGTCACCGGTATGAGACAAATGACTGTTGACTGGGCGGA
GATTATGGATGAGATCAAGAAGAAGAAGCCAGAAATCACACCGCTCAAACAGGCTAAGCC
TGAGGGTAACTTTGATGCAGAGGAGTTCTGTGAATGGGTTGAAGCGACTGATATTGAAGC
AAAGTGTCGCGAAGTTGTTGAGACCGGCGTCCTGTCCGAGGAAATGAAAGAACGCCTGGA
ACAAAGTATGGCTTACCTTATGTCAACCTCCTATGACACACCATTTGAGTCACTCATTGA
AACCCCCCGAACCCGGATTGGTAAAATTTCTGACAGATTAAAGAAGTGGTGGGGTACCAG
GATGGAGAAAATTGGCATTTGGTGGACTAAGTATAAACCCTTCTTCATAGCGGCTGGTAT
ACTTGGGTCAGTTATTTCCACCTGCGTTGGCATTTATATGCTGGTTAAGACCTTCTCACA
TGACCCGATAAAGGAGTTCTTTAACCCGTTGCCAGCCGGAGACCCTGAGAGAGCTTATAA
TGGCCCCACTATGAACACTGCCAAGGCCAACAAGACCCAGCGCATTAAGATTCATCCAGA

TGGCCCTAATCCCGCTGAGTACAGCCACTTGTACAAGGCTGCTGCTGCGCTCCAGATCGG
TGGTAAAAATTGTTATGCCATAGTCGTAAAGCCACACACTGTCATGACATACAAACACTA
CTGGTTCAGGTGTGGGGGGAAAATTGAAGCCCTCCACTGGAATGGCCTTAAGCATTGTCC
TGAGCCATGCACTGTGCATGTCACCGACGTCCAATTGGAGAACGACGACGGTGCTGAGAT
AGTTTCTGATTATATATTTGTAACTTTTGAACGTTGCCCCTTCCAAATGAAGACAATTCA
GAAATTCCACAGTCCACCAGAATGGGGCAGAGAAGGTGTTGCCCTTATGGCACACCCAGC
AGGGAACTACTCACAACATGTTGATGACATACAGGCTTCTGGCGCTTATGTTGTGGGTTT
TGATGACGGAGACCAAGTTTGCTCCGATTCTGTGATATACCGTACCAAATCTTTCGCCGG
TATGTGCGGAGCCATGATCTGCCAGAAAGTTCAGGGCGCCTGGAAAGTTGTTGCCATGCA
CCACGCTGGCAACCGACACACTTTTGGCTTCGGGTGTCGTATTAGAATTCCAGAGCTGCC
AGACGGTGTGGTTACAGAAAAGATACCAGCAGCCAAACCGCACTACACCCCCACCAAGTC
CAAACTAGAGAAGAGTCCCCTGTTTGGCATAGTTGCGCCAGAGATGCAACCAGCACCCCT
CAGTGCCAAAGACAAGAGGCTTGAGGTTGAGGTGGACAATTTGGTTAAGTTTGCCTCAGA
CAAGTACCGTGTCAACGTGTATGAACCTGATAAGACCCTGATGAACGCAGTGGCCATGTA
CACAGCAAAACAAATCTTTGCTGCTACAGGTCGTTGTGGGATGTGGACACTTGAGGAAGC
TCTTGAAGGGGGAATTGGTAACCCGATTGACATGAGAACATCCCCCGGTGAGAAGTATGT
CAAACTCAACATGAGAAAGAAAGACCTTTTCAAAAGGAACAATGATGGAACCTGGTGGGT
TTTTCCTGGTTTCCGCGAGGACGTTGAAGAACAACTCCGTCTCGCGGGGGCTGGTGAAGC
TCACACAGTTTTTGCGGCAACACTTAAAGATGAGTTGCGCGCAAATGAAAAAGTCAGACA
GGGCAAATCCCGATGTATTGAAGCCTGCAATGTCGACTTCACCGTGGCCCACCGCATGAT
TTATGGGCCCCTTTATGAGAAGATCTACAGTTCAACACCCCTGCAAACTGGCCTCGCAGT
CGGGTGCAATCCTTACACAGACTTTCACGGCATAGCCTCTGCCATGAAAGAACACTGGTT
CGCCATTGATTATTCAAGGTTTGATGGCTCACTCTCAAAAGAGTTGATGCAACAAGCAGC
AGAAATTTTGGTTGCTTGCACGGAAAACTGTGAACTGGCCAGGAACATTTTGAAGCCAGT
CATTGATTCCACCCATCTCGTTGCAGACGAGGTTTGGATGGTGAGTGGGGGTATGCCTTC
TGGCTCACCCTGCACCACCGTCCTGAACTCCCTCTGCAACTTGCTGGTTGTGAGATATGC
CATGGCGACAGTTGGTTTCAGTTTTGAAGAAGTCATACTGACCACCTATGGAGATGATGT
GATTGGATCTGCCACACAGAGAGTGTCAGCTGAGTCAGTTGTCAGGACAATAAAAGATGT
CTTTGGCATGGAAGCCACATCAGCAGACAAAAAGTCCCTGGACCTTACAGTCCACCCAAA
CGAGGCCACTTTCCTCAAACGCCGTTTTAGGCACTTCCCAGGTACAAGATTTGTCACAGG
ACAATTGGATTTGGACAGCATGCTCCAAAAGATACAGTGGTGTCACGGTCTCGAGGAGTT
CAAACAGCAGTTTGAGAGTTTCACCCAGGAGTTAGTCCTGCACGGTGAAGAAACGTACGC
TAAGGTAACCCAAGCGTGCGCCCCAATCCTGGACAAGTACAGGATTTTGATCCCGACGTA
TGCGCAGCGCTACGTTGAGGTATATGATATGTTGTTTAACTAATCACCCCGGCCCTTAAG
GCCTTAATTTTTGCTTGCTTAATTTGATAGATAATATTGATAGTTTAAGGTAAGTAGTAT
ACTGCACCATTGTGTGTAGCTCGTCAGTCAAAGGACCCCGTCAGGAGTTGTGTCCAGGTA
ACTTTGCAGTTGTGCACACCGTGGTACAAGTTTTTGTGATA
//

Picornavirus 3CD Alignment

(known/predicted Proteinase/Polymerase boundary and GDD polymerase motif highlighted)

```
DERSV-SD609           -------------------GPLDMKSISKLLVTIEDEDGDLATGLAIGDKTVVTFGHE--
WDALV                 -------------------GSIDMKTISKLLVTLEDIDGDLATGLAIGDKSIVTFGHE--
Aalivirus-A1          -------------------GPVNMKPILKSLVNLQDRDGYRATGLAIGNKTVVTYGHD--
PBD-A1                -------------------GPVNMKPILKSLVNVQDKDGYRSTALAIGNKTVVTYGHD--
QKE55028              -------------------GQINLKPILNSLVNVETENGEVSTAICIGNKEIITYGHD--
Grusopi-A1            -------------------GLMDMRHICQRLVNLRGPKG-TATGLALGGKTIITYGHE--
Grusopi-A2            -------------------GLMDMRHICQRLVNLRGPKG-TATGLALGGKTIITYGHE--
Grusopi-C             -------------------GLLDLRHICQRLVNLEGPHG-EATALALGNKTVITYGHE--
Grusopi-B1            -------------------GFADLRPICNRLVNLKSDRG-EATGLALFGKTVITYAHN--
Avihepatovirus-A      ------------------SGRVNFRHICNRLVNVSNEDE-VATGLAIGGKNVLTFGHS--
Avisivirus-A1         --------------------GPSYYSLKDRLVEVGETGS---TGLALGGKVVLSFGHN--
Avisivirus-C1         --------------------GPNFHSLKDRMVEIGTSHS---TGLLLCDKKVLTFGHN--
Avisivirus-B1         --------------------GPNLFHLKDRLVEVGSSGS---TGLILGGKQVLTYGHN--
Orivirus-A1           --------------------NLLNSVVESAILEVVDVTGHRSTALAIGKKHLVSYSHG--
Orivirus-A2           --------------------NLLNSVVENAIIEVVDITGHRSTALVIGKKHIVSYSHG--
Crohivirus-A          ---------------APFLNEFGHIFNRLAYIEDAA-NPIIVHVLPLWENKVLVYSHS--
Crohivirus-B          ---------------APLLPEMVHLTERTAYIKADN-TRSIYHVVPFFQTKILAYGHL--
Parecho-B             ---------------APYMQDLEHCFAQTAYISSPE-TQDIIHCAALSEDTILVYGHS--
Parecho-C             --------------------DLEHCFAQTCYLSSSV-TTDVIHCAAVSGTTLVCYGHS--
Parecho-E             ------------------VAELEHCFSQAAYISASS-SSHVTHCAAMKENQLLVHGHG--
Parecho-A             -------------------GQLEHIISQMAYITGST-TGHMTHCAGYQHDEIILHGHS--
Parecho-D             ---------------------IEHCMQATAYITGKN-VNYPLHCIAWKQRYIVTYGHI--
Parecho-F             -----------------SWNEYIHLAQYCCYFATEKNMLNPIHGILLGSNQVLTYAHS--
Potamipi-A            --------------GGFNPQEYAHLSKCAVVMYGNF--VKEFYGMASGPHQVLTYKHY-F
Potamipi-B            ---------------GFAPAEYAHLYKRCVTMEGPD--GKRFYGMVTASKQVLTYKHY-F
WCP                   ---------------GPNPAEYSHLYKAAAALQIG---GKNCYAIVVKPHTVMTYKHYWF
Limnipi-B1            ------------------AHEFQHLIDAIVEIKFEAGYKVPLQGLALAGYSLLTYAHG--
Limnipi-B2            ------------------AHEFQHILDAMLTVTFEKEQVMPIQGMAYHDYTLLTYAHG--
Limnipi-C             --------------APVECEQYEHFAKPMVFLKFE-DRKHNSRGFAIAKRQIITYAHG--
Limnipi-A             --------------GGNNSTEYKHILDSMVHLEFESGLRS--TGFCVGQRDIFVYAHG--
Limnipi-D             ---------------GQVDLMPVHILKNCVIIVWGNGIQQ--KGIALGDREICTYSHG--
Pasivirus-A1          --------------------------HYSKYCVFLHSGSITLHGLAFGGNSFLFYTHG--
Pasivirus-A3          -------------------GPYNEFQHYSKYCVFLPCGSVTLHGVAFGNNSFLFYTHG--
Pasivirus-A4          ---------------------------FSKYCVYLSSGTVTLHGISFGNNSFLFYTHG--
Pasivirus-A2          -------------------GPYNEFQHFSKYCVFLHSGSVTLHGLAFGGNSFLFYTHG--
Shanbavirus-A1        -------------------GPGDELTHITRGCVRLVADGIQVFGLAIGDDRILTFGHS--
Kunsagi-A             ------------------SPWQPQFNHCFKNCVFIEAD-GFTWYAVMFGRVLVVNKHYLD
Kunsagi-B             ------------------GPWSAPCHHLFKHTGFIQLESGTVYFCAISSRTVIINTHMFR
Kunsagi-C             ------------------SPFSAPFTHLFKNCAYLTCG-DRWAHALVSGRNLIINKHMSK
Aquamavirus-A1        -----------------GPSDVSEFVHLLKFCAYVETPSGPVFGVILGGRKMYFNTHYAS
Passerivirus          -------------GLQECDIALSRNVVTVTSYDETSGKMLQLNGLALFDRWVATVTHIKL
Sicinivirus           -------------CLSLGAMTISKNVVQITGLDVESGAPCKVNGTGIYDRWILTVSHVVP
Gallivirus            -------------GIVGYNPTIVNN--TVGGVSTNAQKTSTFTAIGIGERYFVTADHVVL
Pygoscepivirus        -------------SLHPAVYKISRSVVRIFSTGPIG--YGSSVGFFIRGRWVVATSHAVG
Kobuvirus             -------------GISPAVPGISNN--VVHVESGNGLNKNVMSGFYIFSRFLLVPTHLRE
Sakobuvirus           -------------GVSPAVVKAMRNCAVIATEGVNGSDPHVVGGFFFFSRYFVTVSHILP
Salivirus             -------------GFDPAVMKIMGNVDSFVTLSGTKP-IWTMSCLWIGGRNLIAPSHAFV
Livupivirus           ------------------ISGKVRGAVHP-IFNSH----GGMSAFNIVGRVWVDPRRFTD
Oscivirus             --------------GVPPILRKVQDSVKWTTFFSDSVPIGACSSWNVVDRFHLTVNHVWE
Ludopivirus           --------------AIPPNIGTIMRNVVTCTGHSEDRYITAQITF-LYSRFGVTVRHIFP
Rafivirus             ----------AIDKATFPLMAESYECRTTQSFMGIMDDMYVMNKHTWDAACSFVMRGKSY
Danipivirus           ---------TLHATNEGLLTEPLYTLIKPNQVVVTNSAGFPANGLMLGRTIMLTNTHMFA
Symapivirus           ---------AEKHGPRSGMGDLIKKLTASTTPLVMSD--QSLTATCVWDRYYVFNAHAFE
Dicipivirus           ----------IYPRVCNNTFPIKFIDADPNS-EDPK--FMHMTTLGLKDWMYAVNTHALE
Rosavirus             ----------IYRPVVANCFPINFYDSDPTDRANPHCGMFTLTAVGVFDRTYICNAHGFK
Myrropivirus          ----------VYTKVTRNCTPVTWHFEDGTQ--------GVLTAVGLRDRTYLVNTHGYS
Pemapivirus           ----------IYNKVERNIFPITAHFENAND--------STLSCIGIADRVYAINFHNIK
Hemipivirus           ----------IGPVICKNTFSIKFINEDTQLS-------TDLTGVGLCDRIFLCNWHGMQ
Tropivirus            ----------IYAKVDKNSFPVFFEYDD-ETG-------GRLTALGVYDRWYVVNKHALK
```

```
Megrivirus           ----------IYPKVEKNCTSITFHQDS---------HSFDLTALFICGRTFVCNNHAFS
Poecivirus           ----------IWTAVERNSVPVFFDN-------------MALTGFGLHDNYMVVNHHALS
Ailurivirus          ----APTMDFEKFVLTHVSATFTFYDGN--K-------ALSQTCLTPTDRLIVVNAHTWE
Mischivirus          ---GPGNPDFERHLACHAVVAIHFFPPNTQQ-------PVSQSAILLFGRCFMVNSHTWN
Cardiovirus          --GPNPVMDFEKYVAKHVTAPIGFVYPTGVS---------TQTCLLVRGRTLVVNRHMAE
Senecavirus          --QPNVDMGFEAAVAKKVVVPITFMVPNRPS-------GLTQSALLVTGRTFLINEHTWS
Mupivirus            --NGNPQLDFEIYCATKMVYQIQFRDGTTVE---------SQSAIAIKERVFVVNAHTMK
Cosavirus            ----SPLMDMEKKIAQNVMPFQIFYNGKR----------YTQSCLAIGKRVILVNKHAFE
Marsupivirus         ----SPNIDLERSLFKRNIHEIEFFTKTEDHS----FTSLIATVLSPGGNNFLVNTHIFD
Aphthovirus          --SGAPPTDLQKMVMGNTKPVELILDGKTVA---------ICCATGVFGTAYLVPRHLFA
Hunnivirus           -GP--MNFDLEKSLLARNIVTLHCRRKDGSE--------FETGALAVRGRLVVMNFHLWN
Malagasivirus        -GPHAVNISMEAAIMKKNMVRVKCTRHDDVV--------FYTTGTFVRDRYMLMNWHLFE
Tottorivirus         -GP--YCQDLEMSFKKKSVLVAPCRRPDGRI--------FNTNMIGLKGRVVLWNFHLFN
Teschovirus          -----ANMEMERTLMKKNIVEMTYEKHNGRF--------QTTTVLFVRDRIFLINTHILS
Bopivirus            ---DGGVLEV----LMDNAVPVQLTRELPDGR----ERVSSFTGYLLRGRCLMVPNHSFS
Erbovirus            --GASTGLTVNSLSLLNNVVPVTVSTVIETENGPLSQIVSECCGTYLYNKVMIMPRHILI
Mosavirus            ----GPKSDAEASLLERNTTPVTYLKDGKEVS--------SLTAIKLCSGKALINKHQFD
Torchivirus          ---LAQAPDLELSVLRNCVPLDVDIPNKPKMM--------PFTALGLFELTFATNRHAIE
Felipivirus          ---------GPDSEFALKLMSTNLLDVLTAKG--------HFSGLAVCDTWILLPMHSDP
Parabovirus          ---------GPNMEFETRLFKSSLFDVQTDEG--------HYSGLGVYDEWMVLPRHSRP
Sapelovirus          ---------GPDLEFAKSLMKSSLFPVCTSTG--------SYTALGLFDQWLVLPAHSNP
Boosepivirus         ---------GPLDEFSASIMKRSLFRVTTEKG--------KFTGLGLFDKYILVPKHATP
Anativirus           -----GPGTHPDLQYIQALLNNNIFPIETGSG--------PYTALGIFERWFVLPKHAVV
Rabovirus            ---------GPDYDFATSLHKKSLFPIRCVNG--------PFTALGLKGKWLVLPRHCVV
Diresapivirus        ---------GPN-EFNNKMLKDSLYDLGTENG--------NYSALGLFDNYYVIPTHAKP
Enterovirus          ---------GPSLDFALSLLRRNIRQVQTDQG--------HFTMLGVRDRLAVLPRHSQP
Crahelivirus         -------------SVLELSGVVHRNLVRVGVGNDDEYIQWRVNGIGIKDDWMLVPAHALE
Hepatovirus          -------------STLEIAGLVRKNLVQFGVGEKNGCVRWVMNALGVKDDWLLVPSHAYK
Gruhelivirus         -------------SVLEEANLVHGNLVRFGIGKEDGVVSWHVNALGVCENYLLVPYHAFK
Tremovirus           -------------SVVDISNVVHGNLVRVGVGPNEARIHWLYNGLGVYDTYILMPYHGIK
Caecilivirus         -------------AVLEIVRLCNSNIVQLGRG-CDESPEWCLNALGIQGHTYLCPQHFFL
Rajidapivirus        ------------------GPTEQKGDKILGNVREVDYGECKMTTTFLDSNTCVLNHHG--
Rohelivirus          ------------GPYSGHVIKEQLKANPKPSFVELPVKSESTNLTDIFTKNQIVYTANGV
Fipivirus            --------------RHAETSDVVRKNMVQIGWSKGEKRFLSGHGVFVTPSIMVCVTHCLA
Harkavirus           -----------------QKVSSNVLNISAVGSVDGVVRTIRMNALAVGGDRFVFPAHLVE
Ampivirus            ------------------SDIPPLFPIIRKNTVRLSSCGFSIRAIGWKKDIILLNRHFAQ
WenzhouPL-48         -------------GFRKPPKKIQRREHLADFECHGLTNNCVPLYSSPLEDTVVGYGFLVD
WenzhouPL-47         AKNLKRTFSIWKACLKDELRKVEKVHYGTTRAFIAPPMESFLMGRFLFGRWKAAFKSNQE
```

```
DERSV-SD609           ------------NFKKVVCFRDTEVNWEMVNSTQITINGDSMDLRQYDVKSDIQFKSVNH
WDALV                 ------------TFKKVVSFRDVDVDWELCDAQKITINGEHMDLVQYKANCDIQFKNVNY
Aalivirus-A1          ------------RFNTLVHLKEEQLDHQLGEPTAIRINGEKMDLVQYEVDCPFQFKSSNH
PBD-A1                ------------LFNSVVHIKEEIVDYPLNEPISIRINNEKMDLVQYEVTAPFQFKSCNH
QKE55028              ------------SFVRVSGFRDEKVMWNLNNPRRVTISDQSMDLTQYTVDTNIQFKNINH
Grusopi-A1            ------------EFHSLEFVKDTQVNANLSPPVAVRISDEPTDLALYQADTPFQFKNAYH
Grusopi-A2            ------------EFHSLEFVKDTQVNANLSPPIAVRISDEPTDLALYQADTSFQFKNAFH
Grusopi-C             ------------EFTKLTYVKDTAMECELKEAVHVQVSGEPTDLAMYECKTSFQFKSASH
Grusopi-B1            ------------NFTHCTYHQNKELNAPLISGIKVAYQGSTTDLAMYNVDLKYQFKNSMN
Avihepatovirus-A      ------------KFTQLDEIRDIQFNAPAKG-TPITYDGEPTDLQLLECDIPHQFKDVSK
Avisivirus-A1         ------------DDSKFIIYKDQ--EHPVVKEENISVNNSPQDLALLTVQTPYQFKELRR
Avisivirus-C1         ------------TDCGFITHKDQ--TFKVTSETYISVSGCDQDLKILEVETPYQFKNCSH
Avisivirus-B1         ------------MDRSFIKHKDI--VYSVTKVEWIKVNDSEQDLAILTIDTNLQFKQLVN
Orivirus-A1           ------------PELTRITFYKGPTSIPIEYAFNINYDGEPTDLVVYKVTGPTQLTSP-Y
Orivirus-A2           ------------PELVRISFYKGPCSIPIEYAYNVNYDGEPTDLVVYKVTGPVQLSSP-Y
Crohivirus-A          ---QFILSKM--EKPHLVYKGFKVPIESAEFKRITISEGPMDVAIISIEKLPFIFKSIRS
Crohivirus-B          ---KNTLNKL--ENPKLVFKGKVFEIEDAVIQDVTLNGKEMDLIVIDLIGFPVQFKDLRK
Parecho-B             ---QFYFNRY--EDLRLHFKGAIFPIEGGKISQVTVNGQPMDLILVKIDKLPITFKNYTK
Parecho-C             ---EFFVDNA--PDLVLHFKGASFAIDGGMISRVSLDGSAMDLVLIKVDKLPIVFKNYTK
Parecho-E             ---AFFLEQE--EDLKLHFKGATFEIDSGQVSQVTLNGQKMDLMIVKLDKFPICFKNYTK
Parecho-A             ---IKYLEQE--DELTLHYKNKVFPIEQPSVTQVTLGGKPMDLAILKC-KLPFRFKKNSK
Parecho-D             ---AEVLPHI--DCPQLWYKGNLFEIEEAEMSYLSSNGGPMDLLLIHLPKFPIQFKDITK
Parecho-F             ---RQWFGKYSDEQIHIFCKGVSYPCDELEIQELTYQGVDLDLCIIRIKKLPILFKNSKK
Potamipi-A            K-SGGTLNGLYWNGLKYSVQDPDDVEFEFFYFEDGDRVYETDMVKINFKKLPFQMKCVQN
Potamipi-B            K-MGGRLTTLWYDNLAYSPAEDEIELEEVEYYDDKGVNYPSDCVLINFEKLPFNMKTGMK
WCP                   R-CGGKIEALHWNGLKHCPEPCTVHVTDVQLENDDGAEIVSDYIFVTFERCPFQMKTIQK
Limnipi-B1            --IGSGRMTVQYGNMSLELEEDEYELAVFSTDDG----DTDLCWIRLHKGIGIQFKNLTN
Limnipi-B2            --IGSGRLTIQHGNMNVELEEDQYEIMIMSTEDG----DTDLCWIVMDKRIGFQFKNMTK
Limnipi-C             --LGNGNLIVHYGGIEMNLTPDDYTISVFVVEDG----ETDMACIEFVSALGVEFPDFTH
Limnipi-A             --VGEGSMMFEHRSVAYEIPEDMYEVEQFVTGQG----KMDMARIVIDSSIGLEFKGMWK
Limnipi-D             --VGAGPVTLKWRGGSLVLAPQELEFENVQFEIDGKVKKTDFVTFQIPKEYGVQFSNIRR
Pasivirus-A1          -----LATLERYDNWVLDYNGSMFDLDIETVDELYLNGESMDLCLITCKPLPVTFASIVG
Pasivirus-A3          -----LSTIERYEGWVLDYNGSTFDLDIESVDELYLNGESMDLCIVTCKPLPITFASIVN
Pasivirus-A4          -----LATIERYDNWVLDYGGSSFDLDIESVDELYLNGESMDLCLITCKPLPITFASIAG
Pasivirus-A2          -----LATLERYGDWVLDYNGSMFDLDIDTVDELYLNGESMDLCLVTCKPLPITFASIVG
Shanbavirus-A1        -----DQVLFKAGTVEVVYGDIRMILESPEFTRLTVGNAETDLAIVKTN-APFRMTSMVK
Kunsagi-A             -----------CWS-GPVVVSTAVSSFSADLTVPPQFVEGDLAYFHLPSAPPLKAAPKHY
Kunsagi-B             -----------DLP-ENFTLTTLLGEFKISKSKLQLRSDGDITYAKCMQIPPHRTIQP--
Kunsagi-C             -----------DFS-GIVKIATAYGEYSG--RLLHIRDEGDVSVFLLPPGTPIYKELKFR
Aquamavirus-A1        -----------VILDKDVTVCTPNKKYFTRLTKVSSNYDTMIVQLDIPELLPSISKYVSS
Passerivirus          ----DRP-MTVDI--------NGCSYPVTRHVV-------HGEIAAVYAPKMPQVRDIRR
Sicinivirus           ----NVSGVVITH--------EGKDYTPSKVIY-------DGEICALYVPGVPQFKDLRR
Gallivirus            ----DNM-AQLTI--------GDTSYPAYKVFS-------FRQLCVLHAPEAPQMKILER
Pygoscepivirus        ----DDAIVEDAV--------N--SYHVDKIIR-------IGELALLHVPKATEHPDLTR
Kobuvirus             P---HHTTLTVGA--------DTYDWATLQTQE-------FGEITIVHTPTSRQYKDMRR
Sakobuvirus           -----ASTVSIGG--------IEHRLEDLEPFV-------WNELLVLKVP-GREHPDLRR
Salivirus             SDEYEITHIRVGS--------RTLDVSRVTRVD-------DGELSLLSVPDGPEHKSLIR
Livupivirus           H---YTESFTLAG--------ATYQWNQCKIEV-------CEELLYFRVPTGPMGKDMTK
Oscivirus             K---ATN-FKIGN--------VMYSKEKISFTR-------IGEAVLFYLPNVPQGKNLLK
Ludopivirus           E---DWTHLEIAG--------KKYEKKDVVVTF-------HYELAYVSLLTGPEYKDIRR
Rafivirus             QKDEIPSWSAPNS--------DLLYFCLKDQTK-------VRNMTKFFLPPPPATKFKMN
Danipivirus           N----RPQTMTLS--------GHGEVRVVNSYV-------DGDHLFVDLDLPTQYRDLRK
Symapivirus           N------SPRLSI--------LGQPFTAAQCSF-------KGDLAMVHLPMLRPGIDLRR
Dicipivirus           DTLWIELRNVFI---------PLAECKRYDIKH--DGKLTDLTILEVPRKYFSAVKDITK
Rosavirus             EATHIGIRGRIY---------AVGDINKRHVRR--NQRRTDLMVFQLPDG--DCVRNILR
Myrropivirus          DGGLTAVRCTING--------ATFQMNELKLQV--SPRSADLSFLTLPTG--NCVRDITK
Pemapivirus           GAREITLRGIRF---------SALDAKASRVIH--DCCKSDLCFITAPKET-PSVPDIRK
Hemipivirus           NATMVKARGLEC---------KISDMAVCRIFRGDIKFPTDLCLAQFPKG--DQFRDIRQ
Tropivirus            DSTRIWIRGVIY---------NVKDMLQRVLQY--KGHETDLVAVWCSES--PQVRDIRH
Megrivirus            RSHTIEIGGQKY---------KPEELSPELLVR--PSGVTDVVICTLPRGDER--KNLVP
Poecivirus            LSDTMTVCGKKF---------KLDDLQCRRLVR--GGKPTDLVVVQLPLSSNIRFKNMSR
Ailurivirus           R-VEDTFEVKG----VKYHR-ESCKYVQLTKDDKD----TDAVFVLLPNG--PLFKNSIN
```

```
Mischivirus       K-DWTKFEIRG----VEYTR-EECDWLDLYKEGIS----TDATVVQLPKG--QMFKDNLS
Cardiovirus       S-DWTSIVVRG----VTHAR-STVKILAIAKAGKE----TDVSFIRLSSG--PLFRDNTS
Senecavirus       NPSWTSFTIRG----EVHTRDEPFQTVHFTHHGIP----TDLMMVRLGPG--NSFPNNLD
Mupivirus         A-DWDSFTLVRPEGSLSFKRDEGYDVVEIHKKGAS----TDLAFVRLHAG--PLFKDNVS
Cosavirus         S-VEHKFVVDQ-----KEYTLDQVTAISLDCGSGV----TDVCAVCLPPG--PDFKSIKK
Marsupivirus      K-LWHHFVYKG----VTYER-DDYMVKSFNSKGQK----LDLYLVMLPRG--NPSHDIIH
Aphthovirus       E-KYDKIMVDG-----RAMTDSDYRVFEFEIKVKGQDMLSDAALMVLHRG--NRVRDITK
Hunnivirus        DATHLQLDGEWM-------PRDTIPAVRPAANGIP----TELVFMNWAKTPGRQFRDITT
Malagasivirus     KCKLIQPDFEF--------PVEEVLALRPTFHGMP----SDLVLLQFPNK-GRAYRDITD
Tottorivirus      MAEEVEIDGEWY-------KLEDLETIRVTSNGEP----TDMVATRLPKG--RPFQDISK
Teschovirus       SIKNFHYENTEI-------PAASVQKVQAIFDGHP----SDVTAVQFTVG--RQYRDITS
Bopivirus         K-DWVRMHVCGF--------MFHRDEITSVAFTIG-GMESDAMMVHLP-KRFPAAKDRTN
Erbovirus         K-DWTHITAGRN--------SATRDQLEAVDVIDQFDMPSDAVAVKFPDKRGSSYKNIMH
Mosavirus         KDIWDTMVIDG--------IKQTRDQCQIVGFTTKRGIAYDLYVVDVPK---MQCRNIVS
Torchivirus       N--CTSFEIQG--------HTYKIEDVDVKMVSTQEGKT-DLAIVTLKKG--TRFRNIMK
Felipivirus       GDVVSVEGKEMD----------VLERVDLNNEQGA------LELTLIRINRPTKFRDIRK
Parabovirus       GESIRVEGDEYQ----------VLDLVELVSKVGD------LELVAVKINRPIKFRDIRK
Sapelovirus       KDSIVFKGESVK----------IVDSMLLESSRGN------LELVVVKLDRTEKFRDIRK
Boosepivirus      TSKVSLDGKEFI----------VLEHIDLHNVSGQ------LELTVLKIDRPVNFRDVRK
Anativirus        EP-MMVGGKQID----------CDDIVELRSNGKM------LELVALHCPTLNEFRDIRK
Rabovirus         GEEITLCDQEFK----------VLDNVELECKEGK------LELRCVKIDRPINFPDITK
Diresapivirus     GQIVKLRGADVK----------VLRAIDLHVHNGP------TELTLLQLDTKERTRDIRK
Enterovirus       GKTIWVEHKLIN----------ILDAVELVDEQGVN-----LELTLVTLDTNEKFRDITK
Crahelivirus      ------FDVD-DFSKLFFHRNGTFYSCDKSKVEILSLETGFQDVLAIRVPGMPKFRNIED
Hepatovirus       ------FEKDYEMMEFYFNRGGTYYSISAGNVVIQSLDVGFQDVVLMKVPTIPKFRDITQ
Gruhelivirus      ------FDSD--YNCFYFFRKNVCYCAKVCDVEMFELGVGYNDLVLMCVPGMPKFKDIRH
Tremovirus        D-----ADVD---DDLYIERAGTIYSTNMKMVQVLFLESREGDLVLINVPRLPKFRDIRN
Caecilivirus      E-----MGCN---DRIHVRSKDVVYSFSRENLVLRTFEGPYQDAVLVDFVGIPPCKKITD
Rajidapivirus     --------------VQISKGMIDIDGTVYRIEDLQMHKTTGDLVICRLPHHKQNVVDLTK
Rohelivirus       SCNGFMVGGSTLLTNKHSWPKDGKITIGDKVYETKVYQSNSGDVLLAEINGFREYKSMSK
Fipivirus         G-------------DTLILSCEEDIVELKKEEYETKSLGGDLTAVMINTTRVRKRRNIVG
Harkavirus        S----FTGTISMQLEHPLFKYSYDYNTETREGRVELGGSPCDMIIMHIPGLSYHFPDITK
Ampivirus         TIPDGATVTMERWTPNTATSRLETFQLKYNVANTAFFIYTDDRPIDLVMWRTGWKTGSFK
WenzhouPL-48      N-VNLLMPKHYLSAYKYLYLSVGSDQTRQEIYLPNSRSIPGKDLMVVNVPPQQSVRKRVK
WenzhouPL-47      R--LFHGLGLDMKSLDVTDFISKFKQYKYFMDVDYKNFDQKLLAQFIKAVAVIIIETIRH
```

```
DERSV-SD609         KIYGEDYHGDGYLVWKEMKHYLYIPV------TNIRPTSTIITQQG-------TTTQHTY
WDALV               KIYEDDYRGDGYLVWKEKKHYLYLPV------SNIHPTSTVITQQG-------TTTAHTY
Aalivirus-A1        RIYDGDYNGDGYLVWKEGNTYSYLPV------SNIHATNEITTTDG-------TTTANTY
PBD-A1              RIHDEDYKGDGYLVWKEGNTYSYLPV------SNIHPTNEITTTDG-------TTTANTY
QKE55028            KIYEEDYNGSGYLLWKEGSSYTVLPV------ENIHATNTLITLEG-------TGTEHTY
Grusopi-A1          LIHDEDYRGKGYLIWKNQTEYMILAV------DNIRPAHPITTNQG-------TISSRVY
Grusopi-A2          LIHDDDYRGKGYLIWKNQTEYMILAV------DNIRPAHPITTNQG-------TISSRVY
Grusopi-C           LIYDQDYRGKGYLIWKHHGEYMLLAV------DNIRPAHPITTTQG-------TISSRVY
Grusopi-B1          LIATEDYHGRGYLVWKNHDTYTMLAV------EDIRPGPQITTIQG-------VVSSRTY
Avihepatovirus-A    LIATEDYRGNGWLVWKDQDQYMVQDV------TKIRPFGSTTTASG-------TTSCQTY
Avisivirus-A1       KIYADVYRGDGFLLFLKKGTLIAHQV------KRITPCDNIMTQQG-------HQTQFAY
Avisivirus-C1       KIYSGNYKGDGNLIFLRNNQLIIKDV------FRIREKQGIGTIDG-------TYTHSAY
Avisivirus-B1       KVYSGEYHGDGYLLYFRDNSLLATQV------TGIRPFDPIATQEG-------HITCRTY
Orivirus-A1         THFSDQLGQHPIMVSRRGGSLTVRPV------DKIQPGGQITTLQG-------TTSSRTA
Orivirus-A2         THFSEHIGLHPIMVSRRSGTLSIRPV------DKIQWGGQITTLQG-------TTSARTA
Crohivirus-A        LVSS-DLGSDTMILWNSPRGFLAYPV------SNAHHSGSIETLEG-------DMTVRTI
Crohivirus-B        HFTS-RIGRENYLVWSTDKGTLVLPV------NNAHLTGNSVTFEG-------TQCYQTI
Parecho-B           YYTT-EVGKETLLIWNSEKGRLAMPV------QCVAPAGPVETMEG-------TITHKTY
Parecho-C           YYTK-TIGRDSLLLWSSPQGRLAMPV------TNVHMAGNLVTLEG-------TQTAQTY
Parecho-E           YYTN-KIGTDSLLIWNSPQGKLAMPV------TNVQMSGPITTQEG-------TQTYKTY
Parecho-A           YYTN-KIGTESMLIWMTEQGIITKEV------QRVHHSGGIKTREG-------TESTKTI
Parecho-D           YISN-GISKEAYLIFSTPMGRMMYEV------KNPYLSGFHQTLEG-------TKNSETI
Parecho-F           HICA-QIGKEPKLMWLTKYGMLVKDV------GQVYYSGRNITQEG-------TVSANTV
Potamipi-A          YLSEPQQGVGGVLIFGGTT-THSVPV------HNVRKILNYCMDVEGD----TWHFNNCL
Potamipi-B          FLAPPKSGDSGVLIYGGKD-IYSQGV------ENVQTMNSYSALIAGK----HTTYIDGI
WCP                 FHSPPEWGREGVALMAHPAGNYSQHV------DDIQASGAYVVGFDDG----DQVCSDSV
Limnipi-B1          SISTPEYGMDCVLVKKIGSNFEIRP-------MTNVVDSDYFMMVS-DVSRLSGVRYGNF
Limnipi-B2          IVASPVYGYDCLLLKKIGHNYEIRQ-------MTNVSEQDHHVLQ--DRDRVIGMRFGNF
Limnipi-C           LISAPEYGRDARLLTAWGGSFHIRT-------GENLTKGDYSMLRDPNSGKMWGMRFGHF
Limnipi-A           HIAKPIFSRDALLITKMDNLFRERE-------AGTVKDAGMIVMKD-FDGNIQGMRMNEI
Limnipi-D           HVAPIEETRDGVMFVPIDEFTIERKD------VANLIVEDHQWLQS------FGDMYGHV
Pasivirus-A1        HLSDGDLGD-GVILWRGREGLTCMPI------YDLHHYGTVTTNQG-------DTCTSAI
Pasivirus-A3        HLSDGDFGD-GVILWRGRDGLTCMPM------YDLHHYGTVTTNQG-------DTCTSAI
Pasivirus-A4        HLSDGDLGD-GVILWRGHDGLTCMPI------HDLHPYGSITTNQG-------DICTSTL
Pasivirus-A2        HLSDGDLGD-GVILWRGQEGLTCMPV------YDLHHYGTVTTNQG-------DTCTSAI
Shanbavirus-A1      HFTRSLGSD-PILVWNTKNGIYTQSV------SNLQALGAATTAEG-------TWSHDCV
Kunsagi-A           TVPEPASGTQAMLLYAGRDGTYAVTT------HTNSYYTFTNGSFFCG------IVPSVH
Kunsagi-B           -VPEIVQGSQTMLLFSTPSGNYIQTV------EK--CASFQNVKFWHG------TQTIAY
Kunsagi-C           -IPSLPFNPPAFLLYMTSDSTFAQQV------KDLKHIPISN--YWHG------AQKDSF
Aquamavirus-A1      -----SVPADALLLYFTGKGFYSQSV------SQVSYLPCLE--VREG------MHGASY
Passerivirus        FIRAPRMHHVG----TVIANST------S---GPVFMLANNVKHG-DTPYPDLTGLTQVY
Sicinivirus         FTRNIRQHTTG----VLPSHTP------S---GPAFILTANVRLR-NSPWPSLTGKREVY
Gallivirus          FIKDCNS-KIG----YLVASFP------R---GNGYIQVSNAQWV-VSDCPEITS-KECY
Pygoscepivirus      FVLPPQG-SSG----LLISSFD------N---GPGFILASDVKYI-TSDCPDITA-AQTY
Kobuvirus           FIG-AHPHPTG----LLVSQFK------A---APLYVRISDNRIL-DLDFPGVVVCKQAY
Sakobuvirus         YVDRPTSATTG----HLIGCLA------G---GPVLMRVDRLRPT-PFKSPGFEARDLVY
Salivirus           YIRSASP-KSG----ILASKFS------D---TPVFVSFWNGKSH-STPLPGVVDEKDSF
Livupivirus         YMRAKPTLYGS---GVMISNLE------G---YTSIVKATQMRDHGMLRTGDGITHSETV
Oscivirus           FVKAR-TIRGVR-AGFLAGNMD------G---VPNVVRVWEMTTFRGIETQDGIFNEHCL
Ludopivirus         FLSEKISEKGY-----LIGTTN------T---TQLLVNFWDASYRKVNR--DGIVIPDAV
Rafivirus           LLSKMYHASHDI-FNLDLIDAK------I---VPATNFRMMPAKRVQIASGEIRVIYNTF
Danipivirus         HVLVAESDVPANPTRSLSGYLV------S---ATPTMVLPTMSVYRTSVDTDLAGLVDAY
Symapivirus         YFVDSPTLCNGK-RGILLVNSN------K---YTTTAIATNITYVPSMETSDGQSDTDIL
Dicipivirus         YLRKTPEIPSYNDPVVLCVRGN----------FNLDVLGDNVRNFS-VINTDTTAYHDVI
Rosavirus           YFRKTPEEAPSRSPAVMAVRGK----------FNIDVLATCVESFP-FVQMSGDINYGAL
Myrropivirus        AFMSQSDSVHVK-AGILAVRSQ----------RSVDVLATGVRPCAPVVCEDGMTLGEIV
Pemapivirus         YLLSQDTDISASKGLLMVRTDK----------KLIDMLATNIKPIG-METIDGIVEPNCF
Hemipivirus         HMM-RLGANVPHDSAIMYVRSE------K---FSCDVMVSEPSDMATVEVNGGGWLSGVM
Tropivirus          LFLSELDETQSWTDGLLLHRADTTGNGKE---HPLDALISNINKMKTLRVAG-DEHKGII
Megrivirus          YLLSQKDRPTNDDVLMVSRSKTIAAN--F---ECTNLRGRKSVCVKEFENADEQNFHRCY
Poecivirus          YLRSVNDGYNATHGMLLNKSHDVVQL--I---EVNNIALGRRVVS------EGDAFEGVI
Ailurivirus         KFIA--SNQTFPIRGTAVNGLN-----AN---GPLMYSGNIITGPSLHETESG-EKSLMF
```

```
Mischivirus          KFMT--KDLPFPQKNTPVTCVNC----SN---GTLFYSGHIIRAPQTCEIIRG-LSSSMF
Cardiovirus          KFVK--AGDVLPTGAAPVTGIMN----TD---IPMMYTGTFLKAGVSVPVETGQTFNHCI
Senecavirus          KFG----LDQMPARNSRVVGVSS----SY---GNFFFSGNFLGFVDSITSEQG-TYARLF
Mupivirus            KFS-----EGLPKKNLEVTGVAHF---CE---VPLLFEGTVVTDVSSMNTTTG-VYNNCF
Cosavirus            HFLP--FNTTMFP-GTRLTILSN----DH---YPMSREGSFLRFEDEVPTNVG-NMPFVM
Marsupivirus         HFVKSWKEESLPTVGSPVNGCVLR---QG---ERFIFSGSFLGTSQYNHTNEG-VFAAVG
Aphthovirus          HFRD---TARMKKGTPVVGVINNA---DV---GRLIFSGEALTYKDIVVCMDGDTMPGLF
Hunnivirus           YFPRSGEGHFKLSPAAKVTGICGH---MQ---PSFMFQAESLGTAESAK-TWESVVPMVL
Malagasivirus        LFINKGE--CNVTPGMTGKGLMMD---EA---P-FMFDIQPVLFAEKIS-VQGLDIPQVL
Tottorivirus         YTTDDSP-----RYGAPIIGVCKS---LD---QNFAGVLRCHKDKVQLT-GFQSTN-DVY
Teschovirus          NFIISLP-----NPGTPIVGLMKT---EG---SSYIWSGECLPFKNTMN-TYEGCVPHVL
Bopivirus            LLSD----FTPPRGAELLVMVNNE---VR---KRNLISGTMIGKRESVPITNNRLFPSVI
Erbovirus            HLAS----TLPIRGKPVFILVNNN---VA---GRAVVHGTYIGCTQKITTLDGFTFPNVS
Mosavirus            MFTD----EQPKQE-TLVGCCNST---NY---QKMMWHGEVLRCVENLHTNDG-ILPKTI
Torchivirus          HLLD----EIVEPTGHVVGIVNSS---LF---PRTLFKGKALRTARKITASGK-QMYNVF
Felipivirus          FFPPAFSAERDCTLVVNNKNFP------------RVMLPVGAVTAFGFLSLSFLPRYNTC
Parabovirus          YFPDSFVSEKNANLLVNNENFR------------NMFCPVGVVTRYGRLNLSGMPVQNTC
Sapelovirus          YLVENFHTEKECWLALNSDQFR------------DVYVPVGSVSLFGFLNLSMTPTYNTL
Boosepivirus         FFPSHFSSVK-AKLLINSDKFP------------EMVLDVGRVTMHGYLNLSFKPVYNTC
Anativirus           HLPESIHGEDGCYLVMNSSVYP------------RMNTSVGRVSVFGLLNLDMQMTHNTL
Rabovirus            RIPKKFVETPDCILCINSEKYP------------TMTCPVGKVKNWGKLVLSGNVTCRTL
Diresapivirus        FIPKTIQTHKQSWLLVDNLNYP------------RMMFPTGTITPYGMINLSGNFRHNIM
Enterovirus          FIPENISAASDATLVINTEHMP------------SMFVPVGDVVQYGFLNLSGKPTHRTM
Crahelivirus         HFVSERDLDRCDGHIATLCTMNGGIYQMISEGTLKLVDRSDYLHRMDDGSTKQLHIGKSW
Hepatovirus          HFIKKGDVPRALNRLATLVTTVNGTPMLISEGPLKMEEKATYVHKKNDGTTVDLTVDQAW
Gruhelivirus         HFIEREQLKHCDGKNGTLATLNCGVFQLIAEGPLDYLEHASYKHPGKDGKPIEITIGACW
Tremovirus           HFSTEENIRRAEGMPGTLCTLDHERFTLVTESDLKMVEAATYVCEDDKGVRTDISVGRSW
Caecilivirus         HFVTQDALPFIAGSSGTLTTQYFGVHTMVTELDLKVLEKFEYNHR-EAGKTRVISNGCTV
Rajidapivirus        YLPTTDYVINQNTTFITLTPKP----------RTMQLGMPNQTNVRIYG-----VTYGGV
Rohelivirus          HFITVSELDGLQMESCLLLTRKNGKE------VRVLESEVKVQKHGVFQHNDEVIKVPIY
Fipivirus            SFMDEAELTRAYTGYAVGYTAAPTSR----IMPFTNLRPTGSVDYPDPQDKTKRIELPNA
Harkavirus           LVASGDTFPRMGMGNVGCLIVRNDG-------LTHALQARNFWHYGRMVLQGKVLSEYII
Ampivirus            DISNHWIRESDLDRVTGKNGYRVSDVITN---FSNFAYTDMEIPFDRASMSPIVIPLAIV
WenzhouPL-48         MFLPKNTLYSEYAGYGHRVVGKLSLT------TGEEDSHGVLKLCEIREEGAVVGTEFMS
WenzhouPL-47         YEKNDEYANARYVYFEELIHTVICAS------KTLFMTNRGNKSGNVLTTELNCLVNFMY
```

```
DERSV-SD609           SY-VGKTWRGLCG-----ALLVGVVNGNPKILGIHVAGNKS-------------------
WDALV                 SY-TGKTWKGLCG-----AVLVGIVNGNPKILGIHVAGNKS-------------------
Aalivirus-A1          TY-IAKTWKGSCG-----SVLVGIVDGNPKILGIHIAGNKT-------------------
PBD-A1                TY-IAKTWKGSCG-----SVLVGIVNGNPKILGIHIAGNKT-------------------
QKE55028              SY-CARTWKGACG-----AVLVGIVNGNPKILGIHIAGNKC-------------------
Grusopi-A1            MY-NAKTGAGTCG-----GILVGLVNGNPKILGIHTSGNGV-------------------
Grusopi-A2            MY-NAKTGAGTCG-----GILVGLVNGNPKILGIHTSGNGV-------------------
Grusopi-C             MY-NAKTGSGTCG-----GVLVGIVNGNPKILGIHTSGNGV-------------------
Grusopi-B1            IY-KANTQRGTCG-----GVLIGFENGNPKILGIHTSGNGI-------------------
Avihepatovirus-A      IY-NCKTGPGSCG-----GVLVAQIGGNLKILGIHTSGNGT-------------------
Avisivirus-A1         RY-QVNSASGWCG-----GVLVGIVGGNPMILGMHVAGNGS-------------------
Avisivirus-C1         AY-SARTGSGSCG-----GILVGYVSGNPIILGMHVAGNGD-------------------
Avisivirus-B1         CY-HAKTARGSCG-----GVLVGMVGGNPMVLGLHVAGNGH-------------------
Orivirus-A1           RY-LGYNKEGDCG-----NVILTMHNGNYRILGIHTAGNGC-------------------
Orivirus-A2           RY-LGYNKEGDCG-----NIILTMHNGNYRFLGLHTAGNGC-------------------
Crohivirus-A          TY-VGQTVRGMCG-----GVLVSKVGGAYKINGLHIAGTGI-------------------
Crohivirus-B          TY-EANTKKGMCG-----GLFVTKIDGAFKIAGMHIAGNGV-------------------
Parecho-B             SY-KVASKKGMCG-----GLLVTRVHGTFKVLGMHIAGNGQ-------------------
Parecho-C             SY-AVSSKRGMCG-----GLLVTRVDGSYKVLGMHIAGNGS-------------------
Parecho-E             SY-KVSSKRGMCG-----GLLVTRIEGAYKILGVHIAGNGM-------------------
Parecho-A             SY-TVKSCKGMCG-----GLLISKVEGNFKILGMHIAGNGE-------------------
Parecho-D             TY-CLNTKKGMCG-----GLLITKIDGNFQIAGLHISGNGV-------------------
Parecho-F             RY-PVDSKKGMCG-----GILITNVGGAYKICGMHIAGNGF-------------------
Potamipi-A            EY-EAQTYPGFCG-----GMLCQKINGTWRVVGMHHAGDGM-------------------
Potamipi-B            SY-QTRNQMGMCG-----GLVCQKQNGSWKIIGMHHAGDSM-------------------
WCP                   IY-RTKSFAGMCG-----AMICQKVQGAWKVVAMHHAGNRH-------------------
Limnipi-B1            KY-FGKHSRGDCG-----SLILQKIKGTWKMIGLHNSGAAA-------------------
Limnipi-B2            KY-FGKHAAGDCG-----SLIIQKIKGCWKVIGMHNSGAAS-------------------
Limnipi-C             RY-LCKTQPGDCG-----SIIIQKQNGTWKLIGMHN--AAG-------------------
Limnipi-A             SY-YALTVRGDCG-----SLLLQKQYGTWKIIAMHN--GSR-------------------
Limnipi-D             AY-RAMTRSGQCG-----SPVFQLQRGTWKLVAMHN-ASNG-------------------
Pasivirus-A1          RY-RAKTTRGMCG-----GLVLSKISGTYKAVGLHVAGNGV-------------------
Pasivirus-A3          RY-RARTTRGMCG-----GLVLSKISGSYKVVGLHVAGNGV-------------------
Pasivirus-A4          RY-RAKTVRGMCG-----GLVLSKINGTYKPVGMHVAGNGV-------------------
Pasivirus-A2          RY-RARTTRGMCG-----GLVLSKIGGTYKAVGLHVAGNGV-------------------
Shanbavirus-A1        SY-NAHTGSGTCG-----GALCVKVGGMYKILGMHVAGNGF-------------------
Kunsagi-A             SY-EVPTQAGMCG-----GLLVLQVGGNWIPYAVHFAGLPH-------------------
Kunsagi-B             AY-NTATRPGMCG-----GLLVCLHEGNWVPVGIHMAGTPS-------------------
Kunsagi-C             SY-STHTRSGMCG-----GLLITQIDGNWVPLGIHMAGLPT-------------------
Aquamavirus-A1        AY-TTRTQKGMCG-----GLLVGKIDGAFKALGFHAAGSLE-------------------
Passerivirus          TY-DTPSYDGLCG-----APLILHHPASPTVVGIHEAGVAG------------------V
Sicinivirus           YY-TGATFPGLCG-----APLILQNPGGPSLVALHQSGVAG------------------T
Gallivirus            HY-SCVSFSGLCG-----APLVLSTPAGPRLVGVHVAGVAG------------------V
Pygoscepivirus        HY-KTPSYHGLCG-----APLLSITGSGPRLVGVHVAGVLG------------------T
Kobuvirus             GY-RAATFEGLCG-----SPLVTDDPSGVKILGLHVAGVAG------------------T
Sakobuvirus           MY-GGPTFPGLCG-----APLFTDDSSGPALLSVHFAGVTG------------------Y
Salivirus             TY-RCSSFQGLCG-----SPMIATDPGGLGILGIHVAGVAG------------------Y
Livupivirus           GY-IAATYEGLCG-----APLISQNPADNHIVGLHYAGYVG------------------H
Oscivirus             GY-RCASYSGLCG-----APLILEDPADYRIAGIHFAGYAG------------------Y
Ludopivirus           GY-RTASFEGMCG-----SPCVCVNNGIATIVGFHTAGIAG------------------Y
Rafivirus             SY-DTNSYPGMCG-----SPVVLMNPAGPKIIGTHISGLAG------------------R
Danipivirus           TY-TSVHFDGLCG-----SPLLLAFANGLRLGGIHTAGAGH------------------A
Symapivirus           QY-TAGVAKGFCG-----APLLVVFPDCLRVAGIHYAGYTG------------------I
Dicipivirus           SY-RAMTTRGFCG-----SPVVSTNNAESVIFGIHMASNG------------------YG
Rosavirus             RY-HAMTMPGYCG-----APLISHDKASEKILGIHMASNG------------------CG
Myrropivirus          TY-KAATTRGYCG-----APLIGYN---DKVIGIHVASDGTDE-------------INGH
Pemapivirus           SY-SCLATFGHCG-----GPILACQSNREFIAGFHIASDS------------------NG
Hemipivirus           QY-VAATRPGFCG-----APVVSTHAAKELLLGVHSAGRG-----------------NG-
Tropivirus            SY-SGLFGQGFCG-----APIVHS-SRKEIVLAIHFAGQL-----------------SSN
Megrivirus            TY-DLKSTPGMCG-----AALISRNPARETLLGIHFAGGP-------------------G
Poecivirus            SY-SASTKRGTCG-----APLIASCKGLDRVLGIHFAGGS-------------------N
Ailurivirus           LY-RATTKYGFCG-----SPIVGPVGGNTRILGMHSAGTCG-------------------
```

```
Mischivirus           IY-QAQTYPGYCG-----SAVVATVKGRKLILGMHSAGNSG-------------------
Cardiovirus           HY-KANTRKGWCG-----SALLADLGGSKKILGIHSAGSMG-------------------
Senecavirus           RY-RVTTYKGWCG-----SALVCEAGGVRRIIGLHSAGAAG-------------------
Mupivirus             RY-VAKTRKGYCG-----AAVVGLEGNAKRVFGMHAAGSEG-------------------
Cosavirus             LY-KSTSYFGMCG-----SVVCSRFVDGGGIIGMHCAGGGG-------------------
Marsupivirus          RY-RANTYSGYCG-----APMLGNVGGSIKIFGVHSSGTGV-------------------
Aphthovirus           AY-RAATKAGYCG-----GAVLAKDGADTFIVGTHSAGGNG-------------------
Hunnivirus            KY-KAQTAPGFCG-----SVVVVDNGIWKKVFGLHCAGAHG-------------------
Malagasivirus         RY-KAQTAPGFCG-----SLIVLDAGIHKRVVGIHCAGAHG-------------------
Tottorivirus          TY-QAATGPGYCG-----SPIFCQVGNGRYVVGMHCAGGTE-------------------
Teschovirus           PY-KAVTAHGYCG-----SVMVADAGVWKGICGIHSLGDGA-------------------
Bopivirus             SY-SCHTENGFCC-----APVVAKERGRYVIVAFHCAGNGS-------------------
Erbovirus             SY-KATTHLGMCG-----APVIANENGNGKIIGFHCAGTGL-------------------
Mosavirus             AY-KTPTRAGFCG-----APLTARRGGQLKIIAVHSAGNGV-------------------
Torchivirus           SY-DCPTYGGYCG-----APIIGQVGNEKKILGIHCAGDGT-------------------
Felipivirus           TY-RYPTKMGQCG-----GVVLKA----GKIVAMHIGGDGL-------------------
Parabovirus           MY-RFPTKNGQCG-----GIVVAN----GKIIAMHIGGDGL-------------------
Sapelovirus           KY-NYPTKVGQCG-----GIVVKA----GKILGMHIGGDGV-------------------
Boosepivirus          TY-LYPTRVGQCG-----GVCVSE----GKIVAMHIGGDGV-------------------
Anativirus            TY-AYPTKTGQCG-----GVICKA----GQVLGIHIGGDGS-------------------
Rabovirus             KY-NYPTKSGQCG-----GVVTKC----GQILGIHIGGDGA-------------------
Diresapivirus         QY-PYPTRSGQCG-----GVVVSD----NKIIAMHVGGDGL-------------------
Enterovirus           MY-NFPTKAGQCG-----GVVTSV----GKVIGIHIGGNGR-------------------
Crahelivirus          RG-RGETLPGSCGG----ILVSSNNKLQNPLIGMHVAAGGG-------------------
Hepatovirus           RG-KGEGLPGMCGG----ALVSSNQSIQNAILGIHVAGGNS-------------------
Gruhelivirus          RG-HGDSLPGTCGG----AVITSSTKCGNKIVGIHVAGGRN-------------------
Tremovirus            KA-KACTVAGMCGG----ALVTSNNKMQNAIVGIHVAGG-A-------------------
Caecilivirus          RG-TAYTTSGMCGG----ALISNVNKVQTPILGIHVAGGAN-------------------
Rajidapivirus         VY-RFPSAPGNCG-----GIYYTEYSNKPMIVGFHFGGVSG------------------K
Rohelivirus           AVGEAYTEAGYCG------GILITTESQPRIIGIHTAGTGF-------------------
Fipivirus             VKALGVGESGLCGM----PWISSVISTGCRIIGLHVAGSDS-------------------
Harkavirus            GY-STVTVVGHCG-----APVLVRLPEGYRVIGIHVAGDGACR-----------------
Ampivirus             AR--GVGSPGICGSP-WVVTSPRYFGTMGKICGIHAFGNEEIV-------------GAIP
WenzhouPL-48          LRVTKETRVGICG---------TPYMCGSFVMGLHVNGAGFD------------------
WenzhouPL-47          GWYVFIKTTGDTSLQSYLRYVRDKNFGDDKAIGLTQEAVDMGFNFHAYKRVMAEIGQTVT
                               *                      .
```

← **3C Proteinase / 3D Polymerase** →

```
DERSV-SD609          LGMAAR--LFPMF---------------------------NQGKAKVVG-PNPT---PYY
WDALV                LGMSAR--LFPMF---------------------------NQGKARVVG-PNLN---NYY
Aalivirus-A1         LGCAAR--LFPMF---------------------------NQGKVVHVE-KTGI---QYH
PBD-A1               LGCAAR--LYPMF---------------------------NQGKVIHVE-KTGI---QYH
QKE55028             MGVAAR--LFPMF---------------------------NQGKATKIA-DIR----PYF
Grusopi-A1           TGAANR--LYSFF---------------------------DQGQVTKKISEDKP---KYF
Grusopi-A2           TGAANR--LYSFF---------------------------DQGQVTKRISEDKP---KYF
Grusopi-C            TGAANR--LYSFF---------------------------NQGKVVKKEIEPRP---KYF
Grusopi-B1           TGAANR--LYNNL---------------------------DQGLVVEKKYVGN----SYH
Avihepatovirus-A     MGAANR--VFPVF---------------------------NQGKVVSKQYAGKI---LYH
Avisivirus-A1        HGIAAR--IFPNF---------------------------SQGIVTQRMPN----TELYF
Avisivirus-C1        TGIAAR--LYPCF---------------------------AQGVTMHKWKQEQMFDTNYH
Avisivirus-B1        QGIAARVERYLWQ---------------------------SQGTVVKIEPG-----TVYH
Orivirus-A1          VGYCNI--VYGVV---------------------------SQGQVLQRRQSDKV----VY
Orivirus-A2          VGYCNI--VYGVV---------------------------CQGLVIQKRETDKR----VY
Crohivirus-A         MGMAASISFINAMPS-------------------------SQGVITHIEDTPLK----VH
Crohivirus-B         IGKSAQVGFFQIAD---------------------------QGIVESKEVSPIV----VH
Parecho-B            VARAAAVHFISNGAAG----------------------FMDQGVVVAKEKLQKP----IY
Parecho-C            IARAASLHCLTQ--------------------------YRNEGVIMSKVFVGHQ----VF
Parecho-E            IGMAAAVGFIQN-APE----------------------YHDQGVVVKRESLPLP----VF
Parecho-A            MGVAIPFNFLKN---D----------------------MSDQGIVT-EITPIQP----MY
Parecho-D            VGSSAMLKVLKQ--------------------------SSNQGVIIETTTSPVR----VF
Parecho-F            EGVSVLLYPTLQ--------------------------MKDEGVVVNVEKAQI-----MF
Potamipi-A           MFGNAVPILR-----------------------------IAQGVVVTKQNTGKT----VF
Potamipi-B           MFGNAFRIKVGV---------------------------VGQGVVTEIRKNPIP----VF
WCP                  TFGFGCRIRIPE---------------------------LPDGVVTEKIPAAKP----HY
Limnipi-B1           GQRCAGVRLDIVPIQM-----------------------VLEGIVVHREAAGLT----SF
Limnipi-B2           GPRCAATRLDYVPVEA-----------------------ISEGIVVSREQSPYH----TF
Limnipi-C            QGSAVAFRFDLYPLD------------------------IAQGVIVSKEKSTMR----SF
Limnipi-A            QGLAYGVRLDVCIAK-------------------------YEGLVTSKTPSDDV----FF
Limnipi-D            RGEACGLNLSKFVLK------------------------DPEGLATLVG-THQR----IH
Pasivirus-A1         YGVAASLSVCKQLE--------------------------SQGLVTDTKPWPGVR---VH
Pasivirus-A3         YGVAASLNVCKQLE--------------------------SQGLVTDVKPWPGVR---VH
Pasivirus-A4         YGVAASLKVCRQLE--------------------------SQGLVTDVKPWPGVR---VH
Pasivirus-A2         YGVAASLSACRQLE--------------------------SQGLVTDVKPWPGVR---VH
Shanbavirus-A1       IGRAIMLPTN-------------------------------QGTYYPINPLPIPP---AN
Kunsagi-A            RGYAQGLNLDWIAAVNAPAHPLPPLPD-----------DALPAPQGIITKIEPTSNFRLG
Kunsagi-B            QGFSAGFVDPYLFES--------------------------ARAQGKIVTVKEVPRFTLG
Kunsagi-C            TGFAASP----IHALP-------------------------PLPQGIITQVREG-QLRIH
Aquamavirus-A1       RGFANAFNSVPPLSTVPDFPESINLSG-----------LFSNDVEGVVVGQFEG--EMLH
Passerivirus         SGVAIPLLPL-LEKIPLP-----------------------TQQSV-ITPLPMPGPG-VH
Sicinivirus          SGYAIPIADL-LAMLDVP-----------------------QSQSE-ILECEPGGPP-PH
Gallivirus           TGYADPLVDL-LHAFKDA-----------------------MPQSL-IVDIPRSGPP-AH
Pygoscepivirus       TGYADPIGPV-VETLNTLS----------------------QPQSV-VIPAEPIEP--VF
Kobuvirus            SGFSAPIHPI-LGQITQFAT---------------------TQQSL-IVPTAEVRPG-VN
Sakobuvirus          SGFGFPLAGL-AEAILGHYA---------------------TSQSI-ITPTPLPADGPVH
Salivirus            NGFSARLTPERVQAFLSHLA---------------------TPQSV-LYFHPPMGPP-AH
Livupivirus          SGFAAIFKLDSVRAVLAIMA---------------------VGQGL-RTYLETLDKT-VH
Oscivirus            SGFATHFNKQELVEAMAKIS---------------------VPQSQ-IVEAGTLEKP-VH
Ludopivirus          SGFSQKFDYK---------L---------------------TTQSL-MKAIEHPGRS-PH
Rafivirus            MGSAEALDATWRTAFVALHP---------------------EYGQG-DMQILGEAEPIAY
Danipivirus          TGYSTRLTREMCNKAFDTLA---------------------RATCEGVRSLVGPITPGSF
Symapivirus          QGFALTFSRVTLSEMAARLP---------------------IPVAKHGIITSREAGPNVF
Dicipivirus          TGFAVPIYLSDIP-----------------------------VTLEGVRKKVG-EVPTPF
Rosavirus            IAYGTSVYQSDFEN----------------------------LEYEGLRTCQG-AGLPVH
Myrropivirus         QAYALTVFLEDFAT---------------------------LPSLEGTRTLIG-PGPRVH
Pemapivirus          YSIGTRITREDVEKYINAKPTIEDSLV--------------IPSYNGLRLETATPVKPVF
Hemipivirus          RGVGSILYREDVDSYMVQHP---------------------VERLEAERKWLEKANQRIH
Tropivirus           MGFGQIITKEMFAS----------------------------PEFEGIRTKIGEAEKPVH
Megrivirus           VGIGVPLYKEDFAH-----------------------------LFQGNLKPIEHPGQPNH
```

```
Poecivirus      KGYAVPLYKEDCEV-----------------------------VFQALYVPTDEVAKSVH
Ailurivirus     VAGATLITQEMIKLALNHLKKK------------------ETLKDEGAITEI-DDGPRTH
Mischivirus     TAGAIFVTQEDLRQVRDYFAKNSAP------------PPPEPLSDEGLLTEL-PDGPLIH
Cardiovirus     IAAASIVS-QEMIRAVVNAFEP-----------------------QGALERL-PDGPRIH
Senecavirus     IGAGTYISKLGLIKALKHLGEPL-------------------ATMQGLMTEL-EPGVTVH
Mupivirus       IAGACCLQRSMIQKVLKQLGVDT------------------KLESQGLMIEK-EPGPFVH
Cosavirus       VSVGTRLTARMIESVFDYFYPP---------------------VAQGIIENT-ETGPRVH
Marsupivirus    IGSSTLVTQDMLRTHIS----------------------------QGLKIDE-PPGEKVY
Aphthovirus     VGYCSCVSRSMLLKMKAHIDPEP--------------------HHEGLIVDTRDVEERVH
Hunnivirus      VGMAAIISREMVDAISQL------------------------AEFQGRIHSA-KNHQYVY
Malagasivirus   VGAASVITKAGLIALLD-------------------------SKFEGKISDV-VEHPYVY
Tottorivirus    IGVACRITRTLVEKVLESFQ---------------------PPSFQGLITDE-QPHPRVY
Teschovirus     IGAATVLSRQHLLNLLEG-----------------------FLEFQGKIYDV-QKTDFVY
Bopivirus       TGFASVIPKRFLLEACDALEKQTTPTHDTVERVDLEQPDTSSAMEAEHITSMRTADKASY
Erbovirus       VGYASNLTKMSANNICKVWGDP----------------------VAQGWTYFDTTHTPVH
Mosavirus       NGFGTLISKKMIEEAVT----------------------------QGIIYDE-RPGPFVA
Torchivirus     TGWATVITKNIVKKIEE----------------------------QGLKVPIGEANPVCH
Felipivirus     NGYGAILTRKHFAF--------------------------MEGAIVSTS----QAPRPIN
Parabovirus     NGYGAILKSSYFNFDRVDVK--------------------SQGEIVSKK----KAEKSVN
Sapelovirus     SGYAAMLKKSYFSV--------------------------CQGEIVHKESTKERGVKSIN
Boosepivirus    NGYGAVLTANMFTK--------------------------IQGHIVEEK----PARAKVN
Anativirus      NGYAASLKRSYFAS--------------------------LQGQIVAER----PARKSVN
Rabovirus       NGYATAIYQHYFDKLDEAE---------------------EQGHIINIQ----QTKTPVH
Diresapivirus   NGYGAALKQSYFDFL---IY--------------------EQGAIVKVERNLET--INVN
Enterovirus     QGFCAGLKRSYFAS--------------------------EQGEIQWVKPNKETGRLNIN
Crahelivirus    NLISKVITKEMLSVMGK------------------------HLTQSGRIKMVEVSDIVVP
Hepatovirus     ILVAKLVTQEMFQNIDK------------------------KIE-SQRIMKVEFTQCSMN
Gruhelivirus    TLISKTVTREDIESLLQ------------------------SEIHAHRTRRVEFITECVP
Tremovirus      HAISRVITKEMIEEMLK------------------------TRAQCSRIWKTEFVEEKIS
Caecilivirus    YAYCAVVTKEMLHQMLL------------------------ASS-QQRIWKVEEAPVPIH
Rajidapivirus   MGFCAPLSFMDYS---------------------------EACADFQGKTLTPCEITRVH
Rohelivirus     KAYTSVVPLEQLYMLAK----------------------------PNRMMKVELQQDTIS
Fipivirus       HSICQPIMREQLEELS-------------------------ALYPQCKIMCVQPAPMKVV
Harkavirus      -GFCVPLISEMLQGG-------------------------IHQQGLPYVVNVGRLDQKVY
Ampivirus       MTIEGLESTELVLPGTSFQPSQ-------VTFQMAVDEDADTQLVFHDFHGRVGPRDAFV
WenzhouPL-48    AGMAIVTREELEEVLFP-------------------------GFVEYELPECEQYVEENW
WenzhouPL-47    PGNKSDVELPYFEDICELQFLKRNFYQLYPTIWIAPLDKTSIESVFNYSCLTEEEIEEWQ
```

```
DERSV-SD609           QPRKTKYEPSP-------------------------VQQDE-PTFGPAVLSNKDKRLEVQ
WDALV                 QPRKTKFEKSP-------------------------VCSDD-PKFGPAVLSNKDKRLEVE
Aalivirus-A1          QPRQTAYEPSP-------------------------VNTGH-STVGPAVLSKNDKRLEVE
PBD-A1                QPRATAYMPSP-------------------------VNTGH-STVGPAVLSRNDPRLEVE
QKE55028              QPRKSKLEPSP-------------------------VYES--SNVAPAVLSKNDPRLDIM
Grusopi-A1            QPRKSAYVPSP-------------------------VHVY--TDVGPPVLSKNDPRLEVE
Grusopi-A2            QPRKSAYVPSP-------------------------VHVH--TDVGPPVLSKNDPRLEVE
Grusopi-C             QPRKSAYVKSP-------------------------VYQD--SDVGPPVLSKNDKRLEVE
Grusopi-B1            QPRKTKFRPSP-------------------------FYVN--PRVSPAVLSNRDPRLIDP
Avihepatovirus-A      QPRKTAYMKSP-------------------------VYED--SPYEPAVLSINDQRLGVK
Avisivirus-A1         QPRRSEIYPSP-------------------------ANDGT-SNVEPPVLSNRDRRLETP
Avisivirus-C1         QPRRSKFSPSC-------------------------FFD-T-GAQEPAILSNRDPRNPG-
Avisivirus-B1         QPRRSRIVPSP-------------------------VYC-D-SALAPAVLSRADPRLEVP
Orivirus-A1           VPHQTNLIKSP-------------------------CWVPS-CELEPAALSGRDPRLEE-
Orivirus-A2           VPHQTNLIKSP-------------------------CWTPA-CDLEPAALSSRDPRLEE-
Crohivirus-A          QPTRSVLKRSP-------------------------LYNTWAVTMAPAVLSPFDTRLDPA
Crohivirus-B          QVSKTKLKQSP-------------------------LNGLWPVEQQPAVLTPNDKRIEEP
Parecho-B             LPSKTALNPSP-------------------------LNGVVPVKMEPAVLSPHDTRLEVI
Parecho-C             QPSKTSLHPSP-------------------------LYKAFEIKMEPAVLSPHDPRLEVA
Parecho-E             QPSKTKISPSP-------------------------LNGIFPIKMEPAVLSPFDSRLIEP
Parecho-A             INTKTQIHKSP-------------------------VYGAVEVKMGPAVLSKSDTRLEEP
Parecho-D             QPGKTQIHPSP-------------------------LHGLWDVKMEPAVLSAHDPRLEVE
Parecho-F             TPAVSKLQKSP-------------------------IHGCYEELKQPAALSPRDPRIEVP
Potamipi-A            SPSKTALRKSP-------------------------LYGLVETTMEPAVLHPRDPRLERQ
Potamipi-B            NPTRSKIMKSP-------------------------LYGVVETRMGPAPLHPHDPRVEEE
WCP                   TPTKSKLEKSP-------------------------LFGIVAPEMQPAPLSAKDKRLEVE
Limnipi-B1            QPGKSSLRPSP-------------------------FHGAFDVTKEPAVLSKKDRRLTVN
Limnipi-B2            QPGRTNLKKSP-------------------------FHGAFDVLKEPAVLSKKDRRLTVD
Limnipi-C             MPSKSKLRESP-------------------------FHGAFPVEKEPAVLSSRDTRLIVN
Limnipi-A             QPPKSAIHKSP-------------------------FYGIQEATMQPAPLRATDQRITVP
Limnipi-D             QPSQSTLKKTP-------------------------IHGVFEETKQPAVLTPRDRRLEVQ
Pasivirus-A1          QPSKSVLKPSP-------------------------LYGFVEQELHPAVLSPFDNRLKCE
Pasivirus-A3          QPSKSVLKPSP-------------------------LYGFVEQELHPAVLSPFDGRLKCK
Pasivirus-A4          QPSKSVLKPSP-------------------------LYGFVEQELHPAVLSPFDKRLKCE
Pasivirus-A2          QPSKSALKPSP-------------------------LYGFVEQELHPAVLSPFDNRLKCE
Shanbavirus-A1        LVTKTRLKPSP-------------------------LHGIVPVTKGPAVLTKNDPRTHGD
Kunsagi-A             FCPYTKYSPSP-------------------------VSLVIRSELEPAVLSAHDNRLEVK
Kunsagi-B             FCPKTKYMPSP-------------------------VSLVVESDLAPAPLSAFDSRLEVK
Kunsagi-C             RPSHTKLRPSP-------------------------VAAIVESELAPAVLSAHDRRLDIQ
Aquamavirus-A1        RSIRTKFRPTD-------------------------LQNKIVPELEPAVLSASDPRLKVQ
Passerivirus          IPRRSRLRPSP-------------------------AMGAFPVEKIPAALSSHDKRLPEG
Sicinivirus           VPRRSRLIKSP-------------------------AYGAFPVTKEPAVLSKYDSRTDK-
Gallivirus            VPRKTKLTHSP-------------------------AWGAFEPTKEPAALLNHDRRLPDG
Pygoscepivirus        VPRKSKLVPSP-------------------------AHGAFPVTKIPAVLSNKDRRIRDS
Kobuvirus             VNRMSRLHPSP-------------------------AYGAFPVKKQPAPLKRNDKRLQEG
Sakobuvirus           VPRRSTLVPSP-------------------------AFGAFPVLKEPAPLTNKDPRLNPD
Salivirus             VSRRSRLHPIPP------------------------AFGAFPITKEPAALSRKDPRLPEG
Livupivirus           IPRTTRLRPSP-------------------------AAGAFEATHGPAVLTTRDPRLMQD
Oscivirus             IPRNSVLKPSP-------------------------AAGAYPTVLEPAVLRRSDGRLLDG
Ludopivirus           INRKTRLEESP-------------------------FFGVFPSTKEPAALTKFDPRLEVD
Rafivirus             VPRVSALKPSP-------------------------YYGMVTPTKSPAVLRQSDPRLGEG
Danipivirus           IPRKSVLKPSP-------------------------LHGVYPVTKIPAALSDYSRTLDPG
Symapivirus           INRNTRLTPSP-------------------------AAGAFPLLKEPAVLRQSDPRLNPD
Dicipivirus           APTHTKLKRSP-------------------------VYGVYPVTKEPAPLKPTDRRIDEG
Rosavirus             VPVRSKLRPSP-------------------------AHGAFPVKKQPAVLRQSDERLCA-
Myrropivirus          MPTTTKLKPSP-------------------------AHGAFPVLKQPAVLHARDGRYQG-
Pemapivirus           AFTKTALAPSP-------------------------AAGAFPATKQPAVLHASDKRLNTC
Hemipivirus           MPAKTHIKQSP-------------------------AYGAYPVEKEPAVLHPKDKRLDPT
Tropivirus            VPTKTSLEKSP-------------------------AYGAYVVEKAPAVLKDSDYRTD--
Megrivirus            IPRRSALKKSP-------------------------AYGAFPVKSEPAILSQKDKRC---
Poecivirus            VPRKTALFPSP-------------------------AYGCFQVKKGPAVLSRNDHRLGE-
Ailurivirus           ISRKSKLKKTM-------------------------AHTVFKPDYAPAALSKKDKRLNEG
```

```
Mischivirus           VPRKTKLRKSP-------------------------AFPIFQPSAGPAVLSKNDVRLNPE
Cardiovirus           VPRKTALRPTV-------------------------ARRVFQPAYAPAVLSKFDPRT--E
Senecavirus           VPRKSKLRKTT-------------------------AHAVYKPEFEPAVLSKFDPRLNKD
Mupivirus             VSRRTQLRPTC-------------------------AKETFNPQFGPAALSKNDKRLLPG
Cosavirus             VPRTSKLKRTN-------------------------ATYPATEKYGPAALSRYDPRLNEG
Marsupivirus          VPRMTSLRQTV-------------------------AHKFYGSSLAPAVLSCRDSRLNDG
Aphthovirus           VMRKTKLAPTV-------------------------AHGVFNPEFGPAALSNKDPRLNEG
Hunnivirus            TPHKTQLYPTV-------------------------ACDDN-TTVEPAALSPNDKRLVKP
Malagasivirus         TPAKTAFYPTI-------------------------AHDEN-TTVAPAVLSPRDPRLTNP
Tottorivirus          MNTKSNFYPTP-------------------------AHDQY-TSVSPAALSPRDPRLDED
Teschovirus           TPTRTSLKPTF-------------------------VCVD--PKLEPAALSHLDPRLKEP
Bopivirus             IPRRSNLRPSP-------------------------VYDGHPS-HEPAVLSSYDDRLEDP
Erbovirus             VPRKTKLKPTV-------------------------AINTFDCDVEPAVLSKFDSGLEEP
Mosavirus             VNRKTQIKRSP-------------------------LFPLFQPEAGPAVLSQYDRRLADG
Torchivirus           VMRKSKICPSG-------------------------FS--YPTDVEPAILTQKDPRLDDG
Felipivirus           LNTKTTLRPSV-------------------------FYDTFKGEKEPAALHVKDRRLE--
Parabovirus           VSTRTSLHPSV-------------------------FHDVFEGSKEPAALSKRDPRLS--
Sapelovirus           VKTKTGLYPSV-------------------------FHDVFEGTKEPAALRPGDSRLK--
Boosepivirus          LPTKTKLHPSI-------------------------YYDVFEGNKQPSVLHEKDPRLSRY
Anativirus            VRSTTSLQPSV-------------------------WHDIVPGEKEPAVLSKFDKRCE--
Rabovirus             VSTKTKLQPSV-------------------------WHDIIPGKKEPAALNQKDKRLE--
Diresapivirus         --TKTKLRPSV-------------------------FYGVFDGEKQPAVLSKFDNRCE--
Enterovirus           GPTRTKLEPSV-------------------------FHDVFEGNKEPAVLHSRDPRLE--
Crahelivirus          VGAKTRFLKSP-------------------------IHDSVKHLTNKVPAALPYQQSHEV
Hepatovirus           VVSKTLFRKSP-------------------------IYHHIDKTMINFPAAMPFSK-AEI
Gruhelivirus          LNARSSFEKTV-------------------------FHKYFDETEINHPSVMPFSRKFDQ
Tremovirus            VGSKTKYHKSP-------------------------LYDFCPQEVIKCPTKLFYQG--EI
Caecilivirus          TTSRTQFEKSP-------------------------IYGVYKVEKEPSVISVRDAR-TEL
Rajidapivirus         TPTKSKLRKSP-------------------------FYGLYEETKQPSPLSEMDPRIKRQ
Rohelivirus           IQRKTKYKKGI----------------------------EFEVDREPAILSDNDVRSVGL
Fipivirus             TSGKSKIEPTV-------------------------FHGWLPETKHPAALGPWTKGVIND
Harkavirus            IPTRTRYGPTV-------------------------FQHYLTTNAAPSVKHPNDPRCEVD
Ampivirus             QPRRTELQPSPI------------------------FNEVCDVTHEPAVLSNRDPRLTNP
WenzhouPL-48          TGNNVGFKHLG-------------------------AYYGEQGDLLSTNINMSTEKVHSD
WenzhouPL-47          ATIREQLIEAMLHGKKYYSAFVKKLREWVSTYKFKHYHPELREAIMPILLNRYVDILRSY
```

```
DERSV-SD609          IEDIT------KHAAQKYIGNHFD---------PPRGAFQMAKSHVTQLLSQVLE-----
WDALV                VEDVT------KHVASKYIGNTFN---------PPRQAFQLAKAHVVSKLSQILR-----
Aalivirus-A1         VEDVT------KNAAAKYIGNVFD---------PPVPIFSLAKAVVIDKIRRVVK-----
PBD-A1               VEDVT------KKAAAKYVGNTFE---------PPVPIFSLAKAVVIDRIRRVVK-----
QKE55028             VDDVT------KNAADKYTGNTFN---------PPKEYMSMAKAAVISKFSKVVK-----
Grusopi-A1           VDDIT------VRAAEKYIGNTFD---------PPPSIFEHAKARLAENLSKVLEY----
Grusopi-A2           VDDIT------VRAAEKYIGNTFD---------PPPSIFEHAKARLAENLSKVLEY----
Grusopi-C            VDDVT------VRAAEKYVGNTFD---------PPPTIFEHAKIRLAENLSKVLEY----
Grusopi-B1           IEDIT------KKAAMKYRGNTFD---------PPGFAFELAKIALFSRLYKVLP-----
Avihepatovirus-A     IEDMA------KKASDKYIGNVFQ---------PPPEAFQLAKTHVAEKLSRVLG-----
Avisivirus-A1        IDDIT------KHNADRHKMNRFN---------PPMDAFQVAKSNVISELASIVE-----
Avisivirus-C1        IEDIT------KHNADKLTGNVFD---------PPEDAFALAKSRLIGSMSAHIE-----
Avisivirus-B1        VEDIT------KRAAAKYVGNIFK---------PPEDCFIAAKAHVTRLLSTVVP-----
Orivirus-A1          PRDLL------VSNCDKYTGNIFE---------ISTELCIDTVASVTSRLMEYGP-----
Orivirus-A2          PRDLL------VANCAKYTGNVFD---------ITTELITDTVATVTGKLMEYGP-----
Crohivirus-A         LERPYP--VLKKAAE-KYRVNVFI---------QPPN-FAKFKSQITDCFVRVLG-----
Crohivirus-B         VES-----VIKQAAL-KYRVNHFA---------PDKDSFFSVKNELKKAFTQNYG-----
Parecho-B            MPS-----VVKTAAA-KYRVNIFN---------PDFEIWERVVDELKSKFRTKLG-----
Parecho-C            LPS-----VVKDAAS-KYRVDVFK---------PDQKLWMYVLDEVKSAFRSKLG-----
Parecho-E            MSS-----VVKTAAL-KYRVNVFN---------VDQQLFLKVVDYWKQKFRQTFG-----
Parecho-A            VEC-----LIKKSAS-KYRVNKFQ---------VNNELWQGVKACVKSKFREIFG-----
Parecho-D            CTS-----VVKMCSNDKYVGNVFS---------VDMDIFKMAMSNVLAKLYRQFG-----
Parecho-F            CNN-----LVKLSAQ-KYRRNRFD---------PEESLMNAVESFVATSFQKYYG-----
Potamipi-A           VDN-----LVKDASD-KYRIDVFA---------PNEKNFERAKLYTKKQLFGVIG-----
Potamipi-B           IPH-----LVKHAAA-KYRVDKFE---------PDEENLNRAMMQTASQLFAVTG-----
WCP                  VDN-----LVKFASD-KYRVNVYE---------PDKTLMNAVAMYTAKQIFAATG-----
Limnipi-B1           VDN-----LVKDNAG-KYRVDRYT---------ANETIMSFAMQKIKDRLVSHVS-----
Limnipi-B2           IDN-----LVKDNAG-KYRVDRYD---------ANETIMAFAVQRVKDRLHPYIS-----
Limnipi-C            IDS-----LVKTNGE-KYRVDRFD---------PNTTVFAVAAHKVKERFQNHIP-----
Limnipi-A            IEN-----LTKEAAE-KYRVDQFD---------VDLNTFAVAKARVLERIRPHVK-----
Limnipi-D            IED-----LVKNSSQ-KYRVDIFD---------PDTPTFLIAKSNVKERLKRVVS-----
Pasivirus-A1         VDSVI------GRQALKYNSNVF----------NPGPYYDEVLEAFAVDFNGRFG-----
Pasivirus-A3         VDSVI------GKQALKYNSNVF----------NPGPHYDEVLEAFAIDFNGRFG-----
Pasivirus-A4         VDSVV------GKQALKYNSNIF----------NPGPYYDEVLGAFAIDFNNRFG-----
Pasivirus-A2         VDSVI------EKQALKYNSNVF----------SPGPEYDKVLEAFAIDFNGRFG-----
Shanbavirus-A1       PLDQI------FLKNVGNYFEVR----------DPN-RFDEAVMNLRARLVDTIG-----
Kunsagi-A            RESNA---MFLLEKTQKYDTNVTV---------PRPLLLQTLATEYGTHLRNLMST----
Kunsagi-B            RESNA---LFLLEKFKKYDRDVSC---------KSPELLTAVTDEYFTKLQVLFTR----
Kunsagi-C            RVSNE---EFLMEKCKKYCSDQVC---------NHPDLLQAVVDELEMAITRHTEL----
Aquamavirus-A1       IDG-----DLPDHLCKKWKVNTKV---------SRPDVLELVVNEYISSLD---CE----
Passerivirus         -------VDVDTVAFSKQNRGDL---------DKPWPTLPVAVDLYFSQCDFPKLR----
Sicinivirus          --------DVDEVAFSKQGGGDL---------DEPWPSLIPATKLYFSHCNFSKLR----
Gallivirus           -------VTVDEVAFSKQNRGDV---------VEPWPGLTEAADLYFSQCNFPRLK----
Pygoscepivirus       G------IDIDEVAFSKQGTGDI---------TTPWSGLEEAADVYFSQ--FPTFR----
Kobuvirus            -------VDLDTQLFLKHGKGDV---------TGPWPGLEAAADLYFST-FPTSLP----
Sakobuvirus          -------VDLDVSVMAKHNKGDL---------TKPWPNLEAAVALYFSY-LPDHFR----
Salivirus            -------TDLDAITLAKHDKGDI---------ATPWPCMEEAADWYFSQ-LPDNLP----
Livupivirus          K-------ALDQTIFAKYVK-DQ---------KEMWPAFEPSLSHYLTAFQNVEIR----
Oscivirus            V-------VLDEAIFSKHDKGDM---------TEPWKNLPQAFSVYFNQFQNKEIR----
Ludopivirus          ---------LDSTIFSKHGRGDI---------TIPWKNLEAAFALYFSAFNGKKFR----
Rafivirus            -------VILDQRIFAKYTG-DT---------CEEWPSFFPACQLYFSK-FPRSFE----
Danipivirus          -------VSLDDVIFQKFTS-DV---------STPWDTLAVGYDIWKAKAMLPEPT----
Symapivirus          -------LSLDAVMFSKFTG-DI---------TKPWRNLRAAVDLYLTSALWDYKHS---
Dicipivirus          -------VDFNEAVFGKYGADM----------KEPFRNLDVGRDVVIARLKKVLPT---K
Rosavirus            -------ADLDEVLFSKYTQDM----------VEPFPGLDIGLQVVRNRLKILIP----E
Myrropivirus         -------DDFDGGLFAKYVSDT----------DVAWPNLSVANDVVLSKLKLLFP----S
Pemapivirus          LSGEERKQQFEDGVFGKLSRNM----------TTPWKNMTASADLYIEKLKQLIP----E
Hemipivirus          -------VDFREVLAARYKGDS----------VKPWKNLELAVDVYKNKLFQLVG-----
Tropivirus           -------VKMSDVLFAKYKADV----------KTPWTNLETSRDVLLARIKSLDP----P
Megrivirus           ------EVDLDEVMFSKHVP-----------DHEGWPTLEPAMAYVVEELMQKCGFSKDD
Poecivirus           ------GVDFEDVIMSKHTKSMSDREVKL--NEEGWDTLKAGVDYVVNKIMRTIGAGRKS
Ailurivirus          V-------DLDKQVFTKHTGNTEKYPE-----EFVWAAREYANELF--THLGKDFG----
```

```
Mischivirus           V-------DFDKQVFSKHSANQKVYPE-----AFRRMARWYANEVF--THIGKDNG----
Cardiovirus           A-------DVDEVAFSKHTSNQESLPP-----VFRMVAKEYANRVF--TLLGRDNG----
Senecavirus           V-------DLDEVIWSKHTANVPYQPP-----LFYTYMSEYAHRVF--SFLGKDND----
Mupivirus             V-------DLDQKIFSKHVQNVCKMPA-----VMSDMAREYANQIF--TQLGRDNG----
Cosavirus             V-------NLDEVIFSKHTQNTLVEKG-----STFRSALDMAAEIYGEKFRGNDFS----
Marsupivirus          I-------DFDEALFKKHTGNCTKFPK-----ALRLATLMYADKLFG--VIGRKNE----
Aphthovirus           V-------VLDEVIFSKHKGDTKMSEEDK--ALFRRCAADYASRLH--SVLGTANA----
Hunnivirus            E-------EFKKTILAKHVGDRTDGPL-----AMIRGARFYARLVR-AKC-GQVNE----
Malagasivirus         Y-------QFKASIMEKHVGDMPHGPD-----VWVRAARVYARLLR-AHIPADVTK----
Tottorivirus          V-------DLDKAVFRKHTSNENRTPN-----WMIEGAREYASIVK-SVCGPGIEK----
Teschovirus           E-------NFKAVILSKHVGDSLQLPW-----GIRWASYKYAERIR-AMLPPDFLE----
Bopivirus             A-------NFEKNLLAKNDAMPQPCDESLR-PWLERAAKDYAAKLF--SVVGKDNE----
Erbovirus             D-------SFELTLLHKNDRR-YPEDASMD-KHLEAAVEAYASSLF--AQIGTDNG----
Mosavirus             I-------VLDEALFEKHVSDMDVLPK-----EFEIACDMYAEELF--ARIGRENG----
Torchivirus           V-------VLDDKIFEKHQNNMETLPP-----VFEVAAKMYAKQVF--SIVGKDNG----
Felipivirus           -------VDLEKAMFSKYKGN-LDVEF--------PPELSLAVDQYVEQIRPLIPPD---
Parabovirus           -------VDLDEAMFSKYKGN-VKVEE--------CPELDVAVDHYVSQLRTILPEN---
Sapelovirus           -------VDLNEALFSKYKGN-KHISI--------PPETFIAIDHYVEQIRPLLPEN---
Boosepivirus          -------VDFDKTLFSKYKNSEKKLQP--------TQHMLTAVKHYADQVKPIIPPN---
Anativirus            -------VDFEKALFSKYEQN-VAVPE--------NENVRTATLHYLEQIRPLMPEN---
Rabovirus             -------VDLDTAVLSKYDNDDYEIGV--------SKHMKEAVAEYTERLKSIMPED---
Diresapivirus         -------VDFEQHLMDKYKGN-KELNI--------TENMKLAIDQYAAQLRPILPDD---
Enterovirus           -------VDFEQALFSKYVGN-TLHEP--------DEYIKEAALHYANQLKQLDIN----
Crahelivirus          DPV--------SVMLSKFDAVDCD-----------EPPDYGQVSEFVLD---VFKQEIGQ
Hepatovirus           DPM--------AVMLSKYSLPIVE-----------EPEGYKEASIFYQN---KIVGKTQL
Gruhelivirus          DVM--------SLMLQKFTRVNQM-----------EPSDFDDVVEHQVA---ILS-PIAQ
Tremovirus            DVM--------QVMLAKYSSPIVS-----------EPLGYATVVEAYTN---RMVSFFSE
Caecilivirus          DFA--------ANMLAKYATPLVE-----------EVPEYKVASTFIKTKLTALMRSKAQ
Rajidapivirus         T-------TFIARILEKSTTN--------------KPVPQFAEEAMDHWIERNIKN----
Rohelivirus           DP--------WVVGLSKYSTPVID----------VRKNYENISDLLLGYFRNVDRR----
Fipivirus             P---------EVQLVVKHRAPIRPVP-------KNWHVAEAVAVVMGDELKTQVCQS---
Harkavirus            ---------FIHAIFKKYDRPIAP--------MPMIKHHQSCVLEYMWSLLNRVCG----
Ampivirus             AD-------FDVDLCRKTDRKSSWFN--------KESEVTMAVNAIIDDLTVLPIN---L
WenzhouPL-48          MYNPVDFPDEFDVSPKNYEVLFKRANKYG--FHQPLGSHTAQETSFAFSLYETLLAGQDR
WenzhouPL-47          LLRIGVLSPSDLQKEKIYCESIFENGRTRLRYYTKTETDSFENEYITESLDKSLMSVMDN
```

```
DERSV-SD609          VEDNMSFEQAVTSD-------VLPIDWQTSSGLKYIGFSKKQ-----------------
WDALV                VSSCMPYEKAITSD-------VLPIDWQTSSGLKYSGKKKEE-----------------
Aalivirus-A1         PSKCMTYDEAISVT-------ELPIDWQTSPGLKYKGRTKAD-----------------
PBD-A1               TSKCLSYEQAITVE-------ELPIDWQTSPGLKYKGKTKAG-----------------
QKE55028             HKDVATYEEAIDST-------ILPIDWTTSSGHKYAPRKKMD-----------------
Grusopi-A1           KPPPLTYDEATSTE-------ILDIDWTTSPGEKYKGKTKKE-----------------
Grusopi-A2           KPPPLTYDEATSSE-------ILDIDWTTSPGEKYKGKTKRE-----------------
Grusopi-C            KPKMLTYEEATSTD-------ILDIDWTTSPGEKYKGKTKKE-----------------
Grusopi-B1           RAKQISFEKATDSS-------YLAIDWQTSPGHKYQGCTKKQ-----------------
Avihepatovirus-A     CHDTISYESAVSSD-------VIPMNWDTSPGIKYKGETKRQ-----------------
Avisivirus-A1        PCYHMTYDQAVDST-------LLPIVWTTSPGLEFKGKTKRQ-----------------
Avisivirus-C1        PEGQATFEEAVSSE-------LLPIDWGTSPGDKYRGKTKAE-----------------
Avisivirus-B1        PVGSLEYREAIDNS-------ILPIDWSKSPGIKYKGMSKRQ-----------------
Orivirus-A1          ---YAPVDYETSFT-------IVDMDWNTSPGHKYHNTCKQM-----------------
Orivirus-A2          ---FSPVDYETSFT-------ILDMDWGTSPGHKYHNTTKDA-----------------
Crohivirus-A         THTGISIEEACFEEG-----DEHALDLKTSPGFKY----VQMGLKKTDLVNRP------N
Crohivirus-B         KCKMMTIEEALLEP------NEHALDLTTSPGNKY----TSQGLRKQNLVDRN------K
Parecho-B            IHKHVSFQKAVQGFS-----SLSSLDLSTSPGQKY----VEKGMKKRDLLSTE------P
Parecho-C            IHSKVTLEQAVLGYS-----DLSSLDLTTSPGTKY----VKLGFKKRDLLSLD------P
Parecho-E            LTQRVSIQQAIQGAG-----KLASLEISTSAGYKY----ASRGIKKKDLISLE------P
Parecho-A            MNGIVDMKTAILGTS-----HVNSMDLSTSAGYSF----VKSGYKKKDLICLE------P
Parecho-D            TNKTVSMEKAIVGFG-----KFNRIDLATSPGIKY----SLK-YKKRDLIQYD------P
Parecho-F            KHHPLMLHQAVKGVD-----GLNPIDMNTSAGVKY----QSRGLKKRDLLAIE------P
Potamipi-A           RHGNWNIQQACSGN------GGNPLDLTTSPGFKY----TQKNLKKKDLIKKL------D
Potamipi-B           RCGRWTIEEALSGG------GGNPVDLTTSPGRKY----TLKKLQKKDLVHRN------E
WCP                  RCGMWTLEEALEGG------IGNPIDMRTSPGEKY----VKLNMRKKDLFKRN------N
Limnipi-B1           RGRMVSIEKAITGC------GCNPIDPTTSPGFKY----TKLGMKKTDLYRIN------V
Limnipi-B2           PGRRITIEQAITGL------GCNPIDKNTSPGLKY----TNLGLKKEDLYKVD------E
Limnipi-C            LGYMISMEDAIRGG------DINPIDKDTSPGYKY----VSRGFRKCDLYQIL------P
Limnipi-A            VGRSIPMEEAITGA------GTNPIDKNTSPGLKY----TRDHLKKSDLFTID------E
Limnipi-D            VGHNVKINDAIRGV------DSNPIDPTTSPGLKY----RELNLSKKDLFTIS------P
Pasivirus-A1         -INEMMSAEEVFALS-----GDEAIELSTSPGLKYTSKGLRKRDLVPGGK----------
Pasivirus-A3         -VNEMMSSEEVFALA-----GEEALDLSTSPGLKYTSKGLKKRDLVPGGK----------
Pasivirus-A4         -VNGMMSADEVFGLT-----GDEALDLSTSPGLKYTSKGLRKRDLVPGGK----------
Pasivirus-A2         -VNPMLSADEVFALS-----GEEALDLSTSPGLKYTSRGLRKRDLVPGGK----------
Shanbavirus-A1       -VHSMATMEEAL-FD-----GANAVDMSTSPGHKYTSQNLRKADLINKEER---------
Kunsagi-A            LASPASIEEAVFDT-------VCPMDHRASAGPHYPGVKRSELIDFQRR-----------
Kunsagi-B            PARPVSIETAVFDT-------VTPMDHKASAGPKYPGIKRSELIDFQHR-----------
Kunsagi-C            VCEPVTLEEAAFDT-------VTPLDHTSSPGYKYAGTKRRDLIDFENK-----------
Aquamavirus-A1       QFEPVTLNEAINSS-------ESPLNFNGTAGAKYPGMNRRQLLLPLN------------
Passerivirus         ---MLSMDEAINGTP-----LLDGLDMKQSAGYPWSLTTNRRSLFTQDPET---------
Sicinivirus          ---TLTMLEAINGTP-----LLDGIDMNQSPGYPWCLTRNRRSLFDIG-ED---------
Gallivirus           ---MLTMDEAINGTE-----GLDGIDMNQSPGYPWNQRTSRRELFILN-ED---------
Pygoscepivirus       ---TLTMSEAINGTP-----SLEGLDMNQSAGIPWSPR-SRRSLFTLE--D---------
Kobuvirus            ---VLTQEQAIHGTP-----NMEGLDMGQAAGYPWNTLGRSRRSLFDEVEP---------
Sakobuvirus          ---TLSMHEAINGTP-----GLDGMDLTQAAGYPWNTRGVSRRSLFIDT-P---------
Salivirus            ---VLSQEDAIRGLD-----HMDAIDLSQSPGYPWTTQGRSRRSLFDE------------
Livupivirus          ---TLTLLEAINGTP-----RLDGIDMSNSAGFPYNTMGISRKSLFDWDDTL--------
Oscivirus            ---TLTLHEAINGTP-----LLDGIDMNQSPGYPYITQGVSRRSLFTWNPD---------
Ludopivirus          ---VLSLMEAINGTP-----ELDGIDMNQSAGYPYALT-TPRRNLFIETAE---------
Rafivirus            ---PLSMFEAINGID-----GLDGIDMNQSPGYPYVSVGRSRRSFFTVGVD---------
Danipivirus          ---LFTVEMALNGAA-----CLDPIDMSLSVGYPYTGLGLSRDDFVTRLPD---------
Symapivirus          ---ELSIEQAINGIP-----YLEPLDMNQSSGFPWCASGVSRRSLFTRDGE---------
Dicipivirus          KFAPCTVSEALNGKD-----GLPKLDLKQASGYPYNLSAIKRKHLIESDKD---------
Rosavirus            KLPQITVAQAINGID-----NMDGLDMNQSPGVPYVSEGVARRSLFDC-VD---------
Myrropivirus         KVGELSIMEAVNGIE-----GLDPLDMSQSPGLPYTKEGITRASLFDR-KD---------
Pemapivirus          RLAPISQHEAINGIE-----GLDGLDMNQSPGYPYTTEGISRRSLFTLTPE---------
Hemipivirus          KCRTLTVWEAINGFE-----NLDPMDMSQSPGYPYNSQGKSRRSLFQRFGD---------
Tropivirus           RFRELTMLEAINGIE-----GLDGLDMTQSPGYPYTTMGISRRSLFEKTID---------
Megrivirus           PVPMWTLEQAINGDG-----VMEGIDMGQSAGYPFSAQGRSRRSFFEWNGE---------
Poecivirus           -FKMLTVEQALNGYG-----VMEGMDMSQSPGYPHNTCGVKRRDLFFQKDD---------
Ailurivirus          ---IMSSEDAIKGIP-----NLDGMDPRTSPGLPYTLHGERRTDHIDFETGS--------
```

```
Mischivirus           ---PLSLKDAIKGID-----FLDAMDPTTSPGLPYSAAGIQRTDLVDFDTGEI-------
Cardiovirus           ---RLTVKQALEGLE-----GMDPMDKNTSPGLPYTALGMRRTDVVDWESAT--------
Senecavirus           ---ILTVKEAILGIP-----GLDPMDPHTAPGLPYAISGLRRTDLVDFVNGT--------
Mupivirus             ---PITVQEAILGMD-----GLDAMEKKTSPGLPYTLQNKRREDLIDFETGSI-------
Cosavirus             ---PLSVEDAILGIP-----GLDRLDPNTASGLPYTKT---RRQMIDFNTGQI-------
Marsupivirus          ---PLTMVQAVNGVD-----GLDAMEKDTSPGLPFTLLHKRRQDIFDFEKVAW-------
Aphthovirus           ---PLSIYEAIKGVD-----GLDAMEPDTAPGLPWALQGKRRGALIDFENGT--------
Hunnivirus            ---RLSLHEAVFGTD-----NLDPMDQTRSPGWPYIG-TKRRPDLL-WQTDEG-------
Malagasivirus         ---RLTIGEAILGIP-----GLDPMDMDKSPGWPYVARNVRRPDLIKFKEHYQ-------
Tottorivirus          ---ALTLGEAVRGID-----GLDPIDFDKSPGYPYVLSGQRRPELL-KDCGDH-------
Teschovirus           ---PLSVREAVEGID-----GLDPMDMDKSPGLPYVKKGLRRTDLWNPKTGSS-------
Bopivirus             ---LLDLRTAIEGLD-----HLEALDMHTSPGLPYTDYGARRVDLFGED-GEP-------
Erbovirus             ---PISEYEAVTGIE-----NLDSVETTTSPGLPYTTAGIPREALFDPDKTQL-------
Mosavirus             ---LVSMYRAMNGDG-----ISDAMDMTRAVGYPYCLDSKKRLDMVEIVETEN------G
Torchivirus           ---EITTTEAINGYK-----TAEKMDLSTSPGYPYVNMGLRRENMLDCADG---------
Felipivirus           VTDPLSLEDVVYGID-----NLEGLDLNTSAGYPYVTMGVRKKDLIPERGQ---------
Parabovirus           VTEPLSLEEVVYGID-----HLEGLDLNTSAGFPYVTMGVKKRDLIPERGE---------
Sapelovirus           LTEQLELEDVVYGIE-----NLEGLDLNTSAGYPYNTMGIRKRDLIPHRGE---------
Boosepivirus          VTEPLALDEVVYGIE-----NLEGLDLDTSAGFPYVTMGTTKKDLIPPRGQ---------
Anativirus            VTEPLPLEDVVYGME-----GLEALDLNTSAGYPYCTMGISKKSLIPPKGE---------
Rabovirus             VTEGITLEEAAYGIP-----DLEGLDLNTSAGYPYTLNGIKKRDILDPETK---------
Diresapivirus         LCVYMEIDEVVDGVE-----GLEPLDLDTSAGYPYNTMGIKKRDIIPERFA---------
Enterovirus           -TSQMSMEEACYGTE-----NLEAIDLHTSAGYPYSALGIKKRDILDPTTR---------
Crahelivirus          VDGFLDSRSAIKGIE-----GLDAIPMNTSAGFPYVLKNLRKEDLVDVEGN---------
Hepatovirus           VDDFLDLDMAITGAP-----GIDAINMDSSPGFPYVQEKLTKRDLIWLDENGL------L
Gruhelivirus          TQGFLSIRESILGIP-----GMDGIDPVTSAGLPYTLRGLSKKDLVDFEEG---------
Tremovirus            PR-QLTYDECINGIE-----GLDAIDLKTSAGFPYNTLGLRKSDLIINGKM---------
Caecilivirus          EQGPLRMEEAVFGID-----GMDRIDPNTSPGLPYILHGIRKKDLFSENEIT--------
Rajidapivirus         --MYFPILSVEEALN-----LDGGIDLTQSVGYPYVNRGMRRKDFVDVDANFR-------
Rohelivirus           ---PLTMHEAVEGID-----GLESVDFKTSSGLPWSLKNMKKKEIWNQGDPIP-------
Fipivirus             YMPVLTMSQVVMGIP-----GLEGVDMKTSAGLPWSQLPQKERHKAKLLAN---------
Harkavirus            PFHRVSFDSAVNGWSP----YVKPLNLQSSAGWPYLKDYTNKQKCLEAGAAEE------G
Ampivirus             VPRLLTLDEAINGVDGPPFMAETGLEMRNSPGYPWNKLSNGKGKFPYFEERPQ------E
WenzhouPL-48          DISLLTDEVILNGDH-----EIMPIKIDSSAGHWSCISNKKKAFIDVQETPEGNWFTWSD
WenzhouPL-47          VKRYIQRKGEALYNLGLNYGNYSPQETNPETDIQFEGVQSDIGPPVKVMCADGPVYAYDL
                                                     .
```

```
DERSV-SD609          ---LVQMESFKADVLKILE-----------GGETFFTCYLK------------------D
WDALV                ---LVQLESFRQDVQEILD-----------GSPTFFTCYLK------------------D
Aalivirus-A1         ---LVQDPKFKEDVKEILA-----------GKPTFFTTYLK------------------D
PBD-A1               ---LINDPSFKEDVKMILS-----------GGPTFFTTYLK------------------D
QKE55028             ---LITDPQFRKDVMEVLE-----------TGSTYFTTYLK------------------D
Grusopi-A1           ---LVVSESFKTDVLTQLA-----------NPNTYFVTYLK------------------D
Grusopi-A2           ---LVASDSFKTDVLAQLA-----------NPNTYFVTYLK------------------D
Grusopi-C            ---LIAMPDFKQDVIDQLE-----------NPNTYFVTYLK------------------D
Grusopi-B1           ---LIDDEKFVADVAQQLN-----------DPDTYFTTYLK------------------D
Avihepatovirus-A     ---LVLKSSFRQDVLKQLQ-----------SPSTVFTCYLK------------------D
Avisivirus-A1        ---LVDDPGFKERVMKLYRS--FAGG-NSAPPQVKYTTYLK------------------D
Avisivirus-C1        ---LVDDKKFRADVYNLVKR--FNGDPNREPVDVYFTCYLK------------------D
Avisivirus-B1        ---CVQDQSFKRDVLHLLL---------AQNPEVEFVTYLK------------------D
Orivirus-A1          ---LYESETFRSDVAKMLE-----------VPTTYFVALLK------------------D
Orivirus-A2          ---LHGSASFKQDVQDMLA-----------VPSTYFVALLK------------------D
Crohivirus-A         ---KFIHPILRNDVRLIFD-----EMAKGQMPVVTFTAHLK------------------D
Crohivirus-B         ---GFISDILRRDVANLEK-----EISN---ADVYFYAHLK------------------D
Parecho-B            ---FWMHPQLEGDVKDILG-----AVYSGKKPHTFFAAHLK------------------D
Parecho-C            ---FFVHPVLLEDVKQLLA-----AVYRLEPPRTIFTAFLK------------------D
Parecho-E            ---FWISDELVEDVKAILG-----DIYAGRVPKVVYTAYLK------------------D
Parecho-A            ---FSVAPLLERLVQDKFH-----NLLKGNQITTTFNTCLK------------------D
Parecho-D            ---LVVHKVLVEDVQKTFE-----DVKSG-CVQTVFATHLK------------------D
Parecho-F            ---FWISDRLAMDIKKYWD-----ALSSGKNPEVLFGCFLK------------------D
Potamipi-A           DGSYIFNHDFEKDVEKMLE-----EIEQG-VAKPIFTASLK------------------D
Potamipi-B           NGILVPTPSFREDVNEAVR-----RIERG-EAQTTFVANLK------------------D
WCP                  DGTWWVFPGFREDVEEQLR-----LAGAG-EAHTVFAATLK------------------D
Limnipi-B1           DGSVWVSDMLRNDVQAWID-----SIDAGETKQTLFNTVCK------------------D
Limnipi-B2           HGDVWVSDRLRADVEKWIK-----NIDSGVCLETVFNTVCK------------------D
Limnipi-C            DGTVQISDMLRKDVEVWLT-----AIKTGKEIDTLFTAHLK------------------D
Limnipi-A            KGNAVVSDRLRADVEEQEE-----LLQSGGYPTTTFTACLK------------------D
Limnipi-D            SGDLWISERLQADVAKKWM-----DLTLTRSIDTTFTAHLK------------------D
Pasivirus-A1         -----ACDLLLSDVERLMN------DPKGTP--VYFYCHLK------------------D
Pasivirus-A3         -----ASDLLLDDVECLIN------DPKGTP--VYFYCHLK------------------D
Pasivirus-A4         -----ACDLLLEDVGRLIE------DPKGTP--VYFYCHLK------------------D
Pasivirus-A2         -----ACDLLMEDVEHLLE------NPKEAP--VYFYCHLK------------------D
Shanbavirus-A1       ----WISPILRADVDQLIE------QAKVCTPLVYFTTALK------------------D
Kunsagi-A            ----TISDRLREDVVALRA---AFARGDNVY--LPFSSFLK------------------D
Kunsagi-B            ----IIHDTLRADVHTLTQ---DLEQGKFDG--VVFSSFFK------------------D
Kunsagi-C            ----IISPRLRNDVANLEL---QFRGTSTGAGEVKFASFLK------------------D
Aquamavirus-A1       -------PQVRDDVVKLAG---DVGNG---TATVVFETFMK------------------D
Passerivirus         -GKYKPVPELEEAVLACLE----------NP-DYFYTTMLK------------------D
Sicinivirus          -GLYHPCPELYQEIEACLH----------NP-DYFYTTFLK------------------D
Gallivirus           -GRYEPVEELKLAVLKLLQ----------DP-DYWYSTFLK------------------D
Pygoscepivirus       -GVYSPVPELEDAVKRVLA----------DP-KYIYNTFLK------------------D
Kobuvirus            -GVFVPKPELQAEINQTLE----------DP-DYVYSTFLK------------------D
Sakobuvirus          -GGYKPTPELEKSVDDCLD----------NP-DYWYTSFLK------------------D
Salivirus            --DGNPLPELQEAIDSVWD----------GG-SYIYQSFLK------------------D
Livupivirus          -SCWVPTPLLEADVNVALE----------TPGAFLYTTFLK------------------D
Oscivirus            -GHWDPVPELVAEVERALE----------NPQEFIYTTFLK------------------D
Ludopivirus          --GWVPTDQIKEDVEKCLK----------DP-VYYYTTHLK------------------D
Rafivirus            --GYYPTIELKNAVMDVLT----------GN-FGKYMTFLK------------------D
Danipivirus          -GTLTPTPLLLREMEKFRS----------NPEDFYFATFLK------------------D
Symapivirus          --NWVPIGELTDAIEATRR----------DPNVLPFCTFLK------------------D
Dicipivirus          -GFLTATPKLLADIEESKK----------HPEKFPYTSFLK------------------D
Rosavirus            -GQWVPRERLASDIAQVSG----------DPSLGHFATFLK------------------D
Myrropivirus         -GKWVPNDRLMNDIKTMYE----------APQSAVFTTFTK------------------D
Pemapivirus          -G-YEPKERLQNDIDAALN----------DPSSFYFTSFLK------------------D
Hemipivirus          -G-YIPCDELNIEIERALR----------SPDDYIFTEFLK------------------D
Tropivirus           -G-WKPKESLERDVERALE----------HPELFCYVTFLK------------------D
Megrivirus           -K-WQATEELKKLVDHALQ----------HPDDYYYATFLK------------------D
Poecivirus           -K-WYPKPEVEKQIMRELE----------DFGQSKFTTFLK------------------D
Ailurivirus          --VSPELGAKIEHMLATG------------EIDIEYQTFLK------------------D
```

```
Mischivirus           --ISAALAVEYNNYVEGN------------YEEHTFQTFLK------------------D
Cardiovirus           --LIPYAADRLKKMNEGD------------FSDIVYQTFLK------------------D
Senecavirus           --VDAALAMQIQKFLDGD------------YSDHVFQTFLK------------------D
Mupivirus             --KDRGLINEIASYQLGN------------YESHVFQTFLK------------------D
Cosavirus             --LDDTLKCRLGQWLAGRP-----------PQEVHYQTFLK------------------D
Marsupivirus          --KDEKAKQMYLQYLNGD------------FEGHIFQTFLK------------------D
Aphthovirus           --VGPEVEAALKLMEKRE-------------YKFVCQTFLK------------------D
Hunnivirus            --LDMDPVLRAELMLMMEG----------NFSHHKFVTFLK------------------D
Malagasivirus         --VELDQILYAEMVNYLSG----------DFRNHKFVTFLK------------------D
Tottorivirus          --FEMDQIVYAELINYLSG----------DFGNHKFVTFLK------------------D
Teschovirus           --LEL----MAEINRYLDY----------NYDKHVFLTFLK------------------D
Bopivirus             --HPEVLGRIKRFLDGDYS-------------EHVFQSFLK------------------D
Erbovirus             --IGPAAERLQEFLRGDFS-------------KHVFQTFLK------------------D
Mosavirus             -KLYLPTEQLVEETEKYFT----------GEEKPKFVTFLK------------------D
Torchivirus           --VYTPKDWFNKNIIAVEK----------DPKDATFATFLK------------------D
Felipivirus           --------PLGALVEALDL----------HGYGHPYVTYLK------------------D
Parabovirus           --------PLTKLQSALDL----------HGTNLPFVTYLK------------------D
Sapelovirus           --------PLTQLQKALDL----------HGYDLPFSTYLK------------------D
Boosepivirus          --------PLTKLQQALDL----------HGTGLPFVTYLK------------------D
Anativirus            --------PLTRLQEALDL----------HGTRLPFVTFLK------------------D
Rabovirus             --------DTKKLQECLDK----------YGVDLPFISYLK------------------D
Diresapivirus         --------DKTKLREVIDK----------YGIMLPYTTYLK------------------D
Enterovirus           --------DVSKMKFYMDK----------YGLDLPYSTYVK------------------D
Crahelivirus          ----ITHELCASRLAYILD-----KFDAGECVDIDYIMAAK------------------D
Hepatovirus           ---LGVHPRLAQRILFNTV-----MMENCSDLDVVFTTCPK------------------D
Gruhelivirus          ----TVCDEVFDRVCSHHS-----YAANGVPLDVDFQICAK------------------D
Tremovirus            ------AQRLQQDVEKMEE-----DLHMNRSIQVVFTTCAK------------------D
Caecilivirus          -------DKFKNDVEDLID-----KVVNGKDFECVFATFPK------------------D
Rajidapivirus         ---LHPTQLLLDELETYDD------------RDQIWTTFPK------------------D
Rohelivirus           ----EFVDAVKYQLSAMTN---------GDPCEVVFATYLK------------------D
Fipivirus             ---PNFLDRVEHIVEYMTN-----PFVPMPVPEIYYSVHAK------------------D
Harkavirus            ------VVELERRYRSGLD------------ADIIFVSYLK------------------D
Ampivirus             DGERLKYDMKDPTLIARVNERMTLAMQGRIPDNSIWLDCMK------------------D
WenzhouPL-48          DYYNKKHPVLGKSLFEVIQERIN-LGEKGIRAESFWVTTLK------------------D
WenzhouPL-47          GQSHGILPKQIPKIMDVAMSLPDNIKHFQPMDPIYLNGILPRVVLSPTLQQIAPKADVLM
```

```
DERSV-SD609          ELRPNDKVAIGKTRAIEAGNFDYVIAWRMVMGRLTARLFNDF---DRITGFAPG------
WDALV                ELRPLDKIALGKTRAIEAGNFDYVIAWRMVMGNMTIQLFNDY---DRVSGFAPG------
Aalivirus-A1         ELRPIEKIASGNTRAIEAANFDHVVAWRQVMGNIVKQLFSDH---DRVTGFAPG------
PBD-A1               ELRSVEKIANGNTRAIEAANFDYVIAWRQVMGNIVKQLFNDQ---DRVTGFAPG------
QKE55028             ELRPLSKVRVGKTRTIDAANFNYVIAYRMVMMKVVKQLFEDQ---DRITGFAPG------
Grusopi-A1           ELRSNEKIRNGNTRAIEACNFDYTVAFRMVMGHHYKNIMNDV---EQLSEICVG------
Grusopi-A2           ELRSNEKIKNGNTRAIEACNFDYTVAFRMVMGHHYKNIMNDV---EQLSEICVG------
Grusopi-C            ELRSSDKIKSGNTRAIEACNFDYTIAFRMVMGNHYRNIMDDV---EQLSEICVG------
Grusopi-B1           ELRPNEKVAMGKTRAIEASNFDYVIAYRMVMGEIYKAIIEDV---QCVSGIAVG------
Avihepatovirus-A     ELRKKEKIKEGKTRGIEACNFDHTVAYRMVMGDIFSNIYDDS---FIMSGCAVG------
Avisivirus-A1        EVRIKEKVKKGATRTITASSFDYTIACRMIFGNIFRQLFGN----GLPAGFAPG------
Avisivirus-C1        ELRPKEKARACKTRVISAANWDYTIATRMVAGPILRQLYAW----GREFGFGPG------
Avisivirus-B1        ELRKLEKIKQGKTRSIEAASFDYTIACRMLFGQIMMHLFVK----GREVGFGPG------
Orivirus-A1          ELRTREKVRKGKTRVIEAANFDYVVAYRMVMGEFLARVIEDP---EKRAGICLG------
Orivirus-A2          ELRTKEKVRKGKTRVIEAANFDYVVAYRMVMGEFLARVIEDP---EKLAGICLG------
Crohivirus-A         ELRKLEKIRSGKTRCIEACDFDYTVAHKMMFGTLYKAIYDTP---GIITGLAVG------
Crohivirus-B         ELRPNTKIKTANTRCIEASDMDYVVLHRMVFGRLYEKIYNSN---VMLTGLAVG------
Parecho-B            ELRKKEKIAQGKTRCIEACSIDYVIAYRVVMSSLYEAIYQTP---AQELGLAVG------
Parecho-C            ELRKRDKVKNGKTRCIEACSIDYVVAYRVVMSSLYEAIYQSK---PQELGLAVG------
Parecho-E            ELRKIEKVMTGKTRCIEAGSVD-LIAYRVIMSELYEKIYQTP---PQVLGLGVG------
Parecho-A            ELRKLDKIASGKTRCIEACEVDYCIVYRMIMMEIYDKIYQTP---CYYSGLAVG------
Parecho-D            ELRKLPKIKSGSTRVIEACSLDYVIVHRMIMGEIYEKIYATA---PQLTGFAVG------
Parecho-F            ELRPIQKIKDANTRCIEAAPLDYVLVHRQILGGIYSQFYSKP---CVLTGMCPG------
Potamipi-A           ELRPIPKVKEGKTRCIEMCEVDYTVAHRMILGSIFESIYNTG---ATATGIAVG------
Potamipi-B           ELRPLAKIKAGGARCIEMCEFDYVVAHRMILGPMFEKIYNAE---MTSTGIAVG------
WCP                  ELRANEKVRQGKSRCIEACNVDFTVAHRMIYGPLYEKIYSST---PLQTGLAVG------
Limnipi-B1           ELRSLEKVALGKTRVIEAAELDYVVAYRMYMSTIYSDLYESS---AEDTGIAVG------
Limnipi-B2           ELRSLEKIALGKTRVIEAAELDYVIAYRMIMTTIYSDIYEAA---AEDIGLAVG------
Limnipi-C            ELRSCEKVELGKTRVIEAAELDFVVAYRMYMSSIYSGFYNTA---AHLTGIAAG------
Limnipi-A            ELRADEKVALGKTRVIEAGELDYVILYRMHMNSIYRDLYNAY---SGDVGVAAG------
Limnipi-D            ELRPVSKVAVGKTRVIEGCELDYVIVYRMVMSTIYRDLYNCP---TVSCGVAVG------
Pasivirus-A1         ELRPQEKIEQGLTRCIESSDFDYVFSAKRVFCKLFRQLYDSD---PIETGFAVG------
Pasivirus-A3         ELRPQEKIEQGLTRCIESSDFDYVISAKRVFCKLFRQLYDSD---PIETGFAVG------
Pasivirus-A4         ELRSQEKIEEGLTRCIESSDFDYVFSAKQVFCRLFRQLYDSD---PIETGFAVG------
Pasivirus-A2         ELRSKEKIEQGLTRCIESSDFDYTFCAKQVFCKLFQKIYDSD---SIETGFAVG------
Shanbavirus-A1       ELRSEDKIKEAKTRVIEASNFDYTVAFRMIFGKQVDIICATP---SEDTGIAMG------
Kunsagi-A            ELRPKPKVRNGDTRVVECSSLHYTVAFRMQFLSVLRMMYGSD---PNQTGLAPG------
Kunsagi-B            ELRSWDKIRQGETRVVECSSLDYTVAFRMQFLEVLTVLYGSD---MVETGLGPG------
Kunsagi-C            ELRPLSKIASGDTRVVEACSLDYTLLMRMYLLRFFQMCYQSD---PTLFGMAPG------
Aquamavirus-A1       ELRPKEKIESGKTRIVESCPLDYLLLYRMVMLKSMIWWYNSD---CIKTGVAPG------
Passerivirus         ELRPTEKALAGKTRLIEAAPIHAIIAGRMLLGGLFEHMHARP---GQ-YGSAVG------
Sicinivirus          ELRGVDKVAAAKTRLIEAAPIHAIIAGRMLFGGLFEVMHSQP---GS-YGSAVG------
Gallivirus           ELRKTEKARAGKTRLVEAAPIDAIIAGRMIFGQLFALFHSNP---GM-FGSAVG------
Pygoscepivirus       ELRPTEKALKGKTRLVEAAPIHAIIAGRMLFGGLFEYMYSNP---GK-HGSAVG------
Kobuvirus            ELRPTAKVEQGLTRIVEAAPIHAIVAGRMLLGGLIDYMQGRP---GE-HGSAVG------
Sakobuvirus          ELRPTEKVALGATRVVEAAPVQAIIAGRMLLGGVLEHMQSNP---CQ-YGSAVG------
Salivirus            ELRPTAKARAGKTRIVEAAPIQAIVVGRRLLGSLINHLQGNP---LQ-HGSAVG------
Livupivirus          ELRSLEKISEGKTRVVESASLPLILAGRMLLGGLFEEMQSNP---GC-YGSAVG------
Oscivirus            ELRKVEKVENGLTRVIEAAPLPVILAGRMLLGGLFEEMQSQP---GK-YGSAVG------
Ludopivirus          ELRPIEKVKAGKTRVIEAAPMHAILAGRMIFGGLFEHMQTNP---GR-YGSAVG------
Rafivirus            ELRNDEKVEQGKTRIVDAANLPSIIAGRMIFGRLFAYMHKNV---GIKHGSAVG------
Danipivirus          ELRNVPKAKAGKTRVVDTAPFPVLVCMRVAFGSFYSFMHANP---GVRTGSAVG------
Symapivirus          ELRPVTKIASGKTRLVDCSPLPHAILMRSLFGTLYAHYHRHN---GTRVGSAVG------
Dicipivirus          ELRSVKKVKAGKTRVVEAGSLPVIVEGRMIFGNLFAYFNTHP---GFETMAAVG------
Rosavirus            ELRSTEKVAAGKTRVVEAGSLPHIIVGRKIFGNLFALFNGNP---GFRTMCAVG------
Myrropivirus         ELRDNGKVSAGKTRVVEAGNIAHTIVGRMIFGNLLARFTQNP---GFEAMCAVG------
Pemapivirus          ELRTNEKVANGATRVVEGDSLPRIIAMRMVFGRFFALFNMNP---GFKTGSAVG------
Hemipivirus          ELRSLDKIKTGNTRVIHGDSLPRIIAARMLFGDFFSKMLGLT---GPKLMCAVG------
Tropivirus           ELRSLAKIKEANTRVIEGSSLPVIIAMRMVFGHFFAWCHQNN---GPILGSAVG------
Megrivirus           ELRPSEKVKAGKTRLVDSDSLPRILAMRMVFGPLFEAMLRKN---GSEIHSAVG------
Poecivirus           ELRSQEKIVAGKTRVIDADRVERVVAMRMVFGQFFEAMLRNH---GTGVFCAVG------
Ailurivirus          EVRPIEKVKKGGTRTVDVPPVEHVILGRMLLGKFCAAFHANN---GTTIGSAVG------
```

```
Mischivirus           EIRSEEKIKAGKTRIVDVPSLAHVIMGRVLLGRFCSKFQASP---GTTLGSAIG------
Cardiovirus           ELRPVEKVQAAKTRIVDVPPFEHCILGRQLLGRFASKFQTQP---GLELGSAIG------
Senecavirus           EIRPSEKVRAGKTRIVDVPSLAHCIVGRMLLGRFAAKFQSHP---GFLLGSAIG------
Mupivirus             EIRPLEKVREGKTRIVDVASVGHCIVGRTLLGRFASKFQTHP---GFKIGSAIG------
Cosavirus             EIRPIEKVKAGKTRIIDVPPLDHVIAFRMLFGRFIAHYHLNF---GFKTGSAIG------
Marsupivirus          ELRPLEKVKEGKTRIIDVAAFPHCLVGRMLLGRFCAHMHKNN---GFYTGSAVG------
Aphthovirus           EIRPLEKVRAGKTRIVDVLPVEHILYTRMMIGRFCAQMHSNN---GPQIGSAVG------
Hunnivirus            ELRDKEKVKQGKTRVIDIASYGHAIMGRVLFGRLAAAMHAHN---GVDLGSAVG------
Malagasivirus         ETRPIEKVAAGKTRVIDVASLGHAIVGRMLFGRLAAWMHSKN---GVDIGSAVG------
Tottorivirus          ELRPDAKVKVGGTRTVDIASFGHMLVGRMLFGRLASQMHLHP---GVELGSALG------
Teschovirus           ELRPKEKVQAGKTRVIDVAGFGHAIVGRMLFGRLFAFFHKNP---GWNTGSAVG------
Bopivirus             ELRPIAKCNAGATRVVEVAAVDHVIVGRMLLGKFTNKLLINH---GDQDRSAIG------
Erbovirus             ELRSKAKVRAGNTRVVEVAAVDHVVAGRMLLGKFTAKMHANN---GLGIGSAVG------
Mosavirus             EVRSNEKIKQGKTRIVDASPFPYAIAGRMVMQNFMSNMMRCN---GTEVGSAVG------
Torchivirus           ELRPIAKARSGKTRIVDASPFCHAIVGRKLLLRFTEKFMVNN---GTSVGSAIG------
Felipivirus           ELRPIEKVKLGKTRLIECSSLNDTIRFKTVFGRFMQVYHRNP---GTITGCAVG------
Parabovirus           ELRPIEKVRAGKTRLIECSSVNDTIWMKTRLGRLFQAFHANP---GTVTGSAVG------
Sapelovirus           ELRPKAKVEAGKTRLIECSSLNDTIRMKRIFGRLFQTFHSNP---GTCTGSAVG------
Boosepivirus          ELRPEEKVKFGKTRMIECSSLNDTIRMKRVMGRLFQAYHANP---GTVTGSAVG------
Anativirus            ELRPKDKIKVGKTRLIEASSLNDTIHMKTHLGRLFAVFNQNP---GTTTGSAVG------
Rabovirus             ELRPLEKIKKGKTRIIECSSMNDTIRMKMMFGKLFQTMHRNP---GTITGSAVG------
Diresapivirus         ELRKPDKIKTGKTRLIEASSMNDTIHARVVFGNLFRTFHLNP---GVVTGSAVG------
Enterovirus           ELRSIDKIKKGKSRLIEASSLNDSVYLRMAFGHLYETFHANP---GTITGSAVG------
Crahelivirus          ELRPKAKVLLSKTRAIECCPFDFNIFVRMVLGRVAAKFYSKP---GLRSHIAIG------
Hepatovirus           ELRPLEKVLESKTRAIDACPLDYTILCRMYWGPAISYFHLNP---GFHTGVAIG------
Gruhelivirus          ELRPQHKVMSGDTRLIECSPIDLTIFLRMCWGKVVSKLQSNP---GWKTGIAVG------
Tremovirus            ELRPLSKVMLGKTRAIEACPVSFTILFRRYLGYALAQIQSHP---GFHTGIAVG------
Caecilivirus          ELRPFEKIKAANTRAIDGSPVSYSTMFRMFCGRAIAWLQTHP---GFLTGIGVG------
Rajidapivirus         ELRSIEKIEMGKPRFIDMCPFPMVVKHRQLIGSFLSYCTQNF---GTRMGSAVG------
Rohelivirus           ELRKKSKILKNATRLIEAAPFHHVVAFRMIAGRFMAHMVKNN---GTKVFSAVG------
Fipivirus             ETLSIDKAKMGRTRWIAAAPFHLVCAARRVFGRAVAAIHLTSPTTMKAYGIAVG------
Harkavirus            ELRPLEKIKQGKTRLIEASPIHYVILFREVVCEFMWSFHARH---GVELHSAVG------
Ampivirus             ELRPKAKCASGATRIINAPPLDLMIAMNVLFG-AFRIFFMDPDHVGLPLESALG------
WenzhouPL-48          ELLLKEKVEAGKTRVFEAPPLDTTILFKKYFGSFANWYRHNA---GPVLSHTIG------
WenzhouPL-47          DIFQYHRAKMCLLRIDSRPPLGYSQLIKIAITSTSADDESAFNRRGVTYNLAKCPTMYFL
                      :     :      *             .
```

```
DERSV-SD609           -----------------------LNPYVYWDSMMENVKESV----IGLDFKNYDGSLSPQ
WDALV                 -----------------------FNPYTCWDSMMDQIKPNI----LALDFKNYDGSLSPQ
Aalivirus-A1          -----------------------MNPYTHFDSLMDQVKWNV----LALDFKKFDGSLSPQ
PBD-A1                -----------------------MNPYTHFDSLMDQVKWNI----LAIDFKKFDGSLSPQ
QKE55028              -----------------------VCPYTHWDSLYDTIHSNV----LALDFSGYDGSLSDT
Grusopi-A1            -----------------------INPYVYFDTIVDSLYDYN----LCLDYKKFDGSLSPQ
Grusopi-A2            -----------------------INPYVYFDTIVDSLYDYN----LCLDYKKFDGSLSPQ
Grusopi-C             -----------------------INPYVYFDTIVESLNEYN----LCLDYKKFDGSLSPQ
Grusopi-B1            -----------------------MNPYEDFDELYYGLYDNN----LCLDFSGFDGSLPPQ
Avihepatovirus-A      -----------------------INPFCEWDNLLANLQPYN----LCLDFSGFDGSLSAQ
Avisivirus-A1         -----------------------MNPYTQFDELYDSCWLNV----ICLDYSKFDASLSKD
Avisivirus-C1         -----------------------LNPYSHFDDLYDKILPFV----ICLDFKGFDGSLSSD
Avisivirus-B1         -----------------------INPYTEFDELFDRLHPHC----LEIDYSGFDGSLSRE
Orivirus-A1           -----------------------LNPYTDFSAIVNSLYQYN----LCLDFKGFDGSLSEG
Orivirus-A2           -----------------------LNPYTDFSCMVNSLYEYN----LCLDFKGFDGSLSEG
Crohivirus-A          -----------------------MNPWKDWELIQQSLFKYN----YDFDYKTFDGSLSRE
Crohivirus-B          -----------------------INPWTDWDSMIQCLNQYN----YDFDFSKFDGSLSDE
Parecho-B             -----------------------MNPWTDWDPMINVLQPYN----YGLDYSSYDGSLSEQ
Parecho-C             -----------------------MNPWTDWDAMLNSLLPYN----YGLDYTSYDGSLSEE
Parecho-E             -----------------------MNPWVDWDSMMEALLPYN----YGLDYASYDGSLYDE
Parecho-A             -----------------------INPYKDWHFMINALNDYN----YEMDYSQYDGSLSSM
Parecho-D             -----------------------MNPWTDFDMLVRSLHDNV----YCFDFRQWDGSLPPE
Parecho-F             -----------------------INPWTDFDMMMSCLHENC----YNLDFSKYDGSLSAE
Potamipi-A            -----------------------IDPVTDFHVLQGIMYPHW----YAMDYSRYDGSLSEG
Potamipi-B            -----------------------CNPYTDFNLFFGTASENW----IAIDYSRYDGSLSEG
WCP                   -----------------------CNPYTDFHGIASAMKEHW----FAIDYSRFDGSLSKE
Limnipi-B1            -----------------------INPPADGHGLFLELNKYHT--FMALDYSRFDGSLPAM
Limnipi-B2            -----------------------INPPQDGHSLYLELNKYST--FLALDYSRFDGSLPKR
Limnipi-C             -----------------------INPPRDGHELYAELCSYSK--FLALDYSRFDGSLPEM
Limnipi-A             -----------------------INPLAEAARLREDLSQYDS--FLALDYSRFDGSLSEK
Limnipi-D             -----------------------CDPLVEAHDWHSCLQSFDN--IMALDYSGFDGSLSEN
Pasivirus-A1          -----------------------INCIADWKNLLDGMYDYI----YDFDLKGYDGSIPRQ
Pasivirus-A3          -----------------------INCIADWKNLLDGMYDYI----YDFDMKGYDGSIPRQ
Pasivirus-A4          -----------------------INCIADWKNLLDGMYDNI----YDFDLKSYDGSIPRQ
Pasivirus-A2          -----------------------INCIANWKSLLDGMFDNI----YDFDLKGYDGSIPRQ
Shanbavirus-A1        -----------------------INPMVDWTSLVRSLYKNN----LDFDYKAFDGSLSTE
Kunsagi-A             -----------------------MNVYLEFSTMVSNLYPNN----LCLDFKKYDSRLPSD
Kunsagi-B             -----------------------INVYTQLYPAFAQLYERN----LCLDFRKYDSRLSTE
Kunsagi-C             -----------------------MNVYTDMLPLCTSLFDYN----YCFDYSKFDSRLPLQ
Aquamavirus-A1        -----------------------MNVYTDFVPMVKQFKKIK----YCLDFSAYDSTLSDE
Passerivirus          -----------------------CDPDYHWTPFFHSFDRF--AEVWALDYSCFDSTLPSC
Sicinivirus           -----------------------CDPDYHWTPFYHNFLDY--REVWALDYSNFDSTIPSV
Gallivirus            -----------------------CDPDFHWTPFAHSFKPF--RNVWSLDYSCFDSTLPSV
Pygoscepivirus        -----------------------CDPDIHWTTFFHSFSDF--ENVWAIDYSCYDSTLPTA
Kobuvirus             -----------------------CNPDVHWTSFFYAFSEF--SQVYDLDYKCFDATLPSA
Sakobuvirus           -----------------------CDPDRDWTRIYWDFNEF--DEVWDMDYKCFDATLPTV
Salivirus             -----------------------CNPDIHWTQIFHSLTSF--SNVWSIDYSCFDATIPSV
Livupivirus           -----------------------ADPDVHWTQFYHSFVDY--SRVYDLDYKNFDGSIPSG
Oscivirus             -----------------------CDPDIDWTKFFWKFERF--EHVYDMDYKAFDSTVPTA
Ludopivirus           -----------------------CDPEVNWSTFFYDFSSF--EQVFDLDYSAYDSTVPSI
Rafivirus             -----------------------CNPDFHWTQFYGEFSKF--EEVWDLDYSAYDSSIPTF
Danipivirus           -----------------------CNPDIHWTAFANGFAGY--PDVFDIDYSCYDGTVPSA
Symapivirus           -----------------------CNPDVDWTRYFQSFQSF--ENVYDLDYSGFDGSIPSC
Dicipivirus           -----------------------CDPEVCWTDWYYKMREK--AHTWDYDYTGFDGSIPSC
Rosavirus             -----------------------CDPDVTWTDLYHPLSSK--AYVFDYDYSGFDGSVPSC
Myrropivirus          -----------------------CDPEVHWTTLYWEMYDK--QHVYDFDYSGFDGSVPTV
Pemapivirus           -----------------------CDPDFHWTQWYHELAKK--SNVYDLDYKNFDGSLPTC
Hemipivirus           -----------------------CNPDVHYTEWFGRLHNS--LICGDMDYKNFDYTVPTV
Tropivirus            -----------------------CNPDSDWTRYFHALNEK--NFCFDVDYKNFDGTVPSI
Megrivirus            -----------------------CNPDVDWTRFYYEMGPDSFPYCFDLDYSCFDSSEPKI
Poecivirus            -----------------------CDPDVFWTRLYYEVGPTAYAYLYDFDYRNFDSHQPKA
Ailurivirus           -----------------------CDPDVDWTRFATEFCECE--NIYDIDYSAFDSSHGTG
```

```
Mischivirus           -----------------------CNPDTDWTKFAHELMERH--WCYDIDYSNFDSTHGTG
Cardiovirus           -----------------------CDPDVHWTAFGVAMQGFE--RVYDVDYSNFDSTHSVA
Senecavirus           -----------------------SDPDVFWTVIGAQLEGRK--NTYDVDYSAFDSSHGTG
Mupivirus             -----------------------CNPDVDWTRFYHDAMRRK--YVYDVDYSNFDASHGTA
Cosavirus             -----------------------CDPDVAWASFGFELSGFP--YLYDFDYSNFDASHSTS
Marsupivirus          -----------------------CDPDVDWTRFVLEAGHFS--YVFDIDYSGFDSCHSTL
Aphthovirus           -----------------------CNPDVDWQRFGTHFAQYR--NVWDVDYSAFDANHCSD
Hunnivirus            -----------------------TNPDIDWTRYAAEFK-FK--NFVDVDYSGFDATHSTF
Malagasivirus         -----------------------CNPDLDWTRYAMEFK-YK--HFADVDYSGFDASHGTF
Tottorivirus          -----------------------CDPDTDWTRFANELD-NS--YYLDLDYSGFDSTHGIG
Teschovirus           -----------------------VNPDLAWTQIFYTAP-SR--NVLAMDYSGFDASHTSG
Bopivirus             -----------------------VNPDLDWTRYYYDFHKFR--YVYDFDYKAFDSSHSKL
Erbovirus             -----------------------CNPDVDFTRFAYQILDWD--YVYDIDYKNFDASHSPK
Mosavirus             -----------------------CDPDTEWTRYFFELC-DK--YVFDLDYKAFDSTHPTA
Torchivirus           -----------------------TDPDCDWTRFYHELS-NE--YVFDLDYSQFDSTHPTA
Felipivirus           -----------------------CNPDEHWSQFYHEFKQEP---IMAFDYSNYDASLHPI
Parabovirus           -----------------------CNPDVHWSKFYAEIGDNP---LLAFDYSNYDASLGPF
Sapelovirus           -----------------------CNPDYHWSQFASEIGMDN---ICAFDYTNWDASLSPF
Boosepivirus          -----------------------CDPDEHWSKFYAEMAPNP---LVAFDYSNYDGSLHPV
Anativirus            -----------------------CNPDTDWSKFVNMIGHDN---ICCFDYKNFDASLGSV
Rabovirus             -----------------------CNPDTDWTKFRAEMH-DS---IIAFDYSNYDASLNKV
Diresapivirus         -----------------------CNPDTDWTSFFNMLGVEN---ICEFDYTNYDASLNPV
Enterovirus           -----------------------CNPDTFWSKLP-ILLPGS---LFAFDYSGYDASLSPV
Crahelivirus          -----------------------IFPEDDWHPLFTSLVRFA-EFGVDLDFQNFDGTVSKF
Hepatovirus           -----------------------IDPDKQWDELFKTMIRFG-DVGLDLDFSAFDASLSPF
Gruhelivirus          -----------------------IDPETQWHALWMSALRFG-DQCVDLDYKNFDCSLHAY
Tremovirus            -----------------------VDPDQDWHCMWYSIVTQC-DLVVGLDFSNYDASLSPF
Caecilivirus          -----------------------LDPDRDWSHMFREMESKG-GTAIDLDFKNFDASVQPW
Rajidapivirus         -----------------------CDPDIYWTQLYHELEPLY---CYDGDYSGYDGTTSAA
Rohelivirus           -----------------------CDPDVDWHRFLTEFKMKC-YKIVDLDFSDWDGTMQPW
Fipivirus             -----------------------MDPEKEWTQLALAKPGWP---VIALDYSNFDGSLQSH
Harkavirus            -----------------------CDPEVFWTVLFHSLKQHK--HAFDIDYSKFDASVSVE
Ampivirus             -----------------------VDPRTVWPDYGILYRQAMS--LFGVDFSKFDSSQLAE
WenzhouPL-48          -----------------------VEKEKVWGSLYYHLKRNS-DFGIAADYSQFDGTIPPS
WenzhouPL-47          IPFCDRDFVKTRYGKWFKVLIEQVTPPVILSDVPEPFRFRPSFEVLELDYYVHKAAPNTA
                                                                      *    .
```

```
DERSV-SD609           VMEAAVEVLACFHKQ----------------PELVKLIHYPTIYSTNLVSDEKWFVEGGM
WDALV                 VMEEAVDILASFHTD----------------PELVKRIHKPTIYSTNLVSNEVWEVEGGM
Aalivirus-A1          VMEEAVDILASFHDM----------------PQMVKDIHKHTIYSTNVVSDETWFVEGGM
PBD-A1                LMEAAVEVLACFHEE----------------PQMVVDIHKPTIHSWNVVSDETWLVEGGM
QKE55028              LMLEAVEVLSCFHEQ----------------PDLARFIHLPTIYSENLVADEKWYIEGGM
Grusopi-A1            VMEAAVEVFSWFASN----------------PDLVKAIHRPTIYSTNWVSNQVWTVEGGM
Grusopi-A2            VMEAAVEVFSWFANN----------------PDLVKAIHRPTIYSTNWVSNQVWTVEGGM
Grusopi-C             VMEAAVEVFSWFARD----------------PDMVRKIHHPTIYSTNWVSNQVWTVDGGM
Grusopi-B1            LMEAAVEVLSHFHVE----------------PDLVVKIHQPVIKSTNLVGDELWKVDGGM
Avihepatovirus-A      ILEEAVDVLSYFHND----------------PALVKRIHAPTIYSTHYVTDEIWQVEGGM
Avisivirus-A1         LMEHAIEVVSCFSED----------------PMSVIRAFQPTLISQERVSDELWEVRGSM
Avisivirus-C1         LMFEAAQVIACFSTK----------------PEAIMASAELTIGSTERVSDEVWYNYGGM
Avisivirus-B1         LMIHCLDVLVSFHES----------------PETCRKLAMLTIDSVERVSDEVWHVSGGM
Orivirus-A1           LMRAAVQCLANCSSN----------------PELVVKIHEPTIVTTEIVRDEEWLVSGGM
Orivirus-A2           LMRAAVQCLANCSTD----------------PELVVKIHEPTIVTTEIVRDEEWLVVGGM
Crohivirus-A          LMLHAVDILSACVEN----------------DEMAKLMLSVVVESVHLVLDQKWNVSGGM
Crohivirus-B          LMLHAADILASCTEK----------------PDLAKKILLKTIYSKHIVKDELWNVKGGM
Parecho-B             LMRYGVEILAYCHEQ----------------PEAVMILHEPVINSQHLVMDEIWHVNGGM
Parecho-C             LMRSAVEVLAYCHED----------------PDQVMVLHETVLNSEHLVMDEIWEVKGGM
Parecho-E             LMRSDVEVMAYCHVD----------------PEQVMILHETVINSEQAVMDEIWSVHGGM
Parecho-A             LLWEAVEVLAYCHDS----------------PDLVMQLHKPVIDSDHVVFNERWLIHGGM
Parecho-D             LMDAGVWVLSGLHED----------------PSLVRNLMAPVITSEQICLDAKHLVYGGM
Parecho-F             IMEMGVEIIKGCSEV----------------PHLVDQIFKPVITSQHHVFDEVWTVYGGM
Potamipi-A            LMRAGVEILSACHED----------------PDLVVKTLEPVVNSVHRVADELWNVVGGM
Potamipi-B            LMRKGVEVLAACHED----------------PELVEKLLEPVVMSTHLVADEEWFVKGGM
WCP                   LMQQAAEILVACTEN----------------CELARNILKPVIDSTHLVADEVWMVSGGM
Limnipi-B1            LMRNAVEILASLHHD----------------EDKVKLLHETVITSKHLVADEFWTVKGGM
Limnipi-B2            LMEKAVDVLASFHVE----------------EEVAKLIHQTVITSKHQVVDEFWCVQGGM
Limnipi-C             LMRKAVEILAELHES----------------PEEVARLHETVIVSKHLVLDELWTVKGGM
Limnipi-A             LMRAAVDILADLHED----------------PDLVRRLHEPVVISKHLVVDEDWIVTGGM
Limnipi-D             LMRHAVDVLSSLHED----------------PALVRALHEPVINSTHVVSDEVWFVRGGM
Pasivirus-A1          LMIDACRVLSESCSD----------------EELAYNLLHKTVDSQHVVGNQLLTVSGGM
Pasivirus-A3          LMIDACRVLSESCSD----------------EELAYNLLYKTVDSQHVVGSQLLTVSGGM
Pasivirus-A4          LMIDAARVLSESCEN----------------EELAYDLLHKTIDSQHVVGNQLLTVSGGM
Pasivirus-A2          LMIDAARVLSESCED----------------EELAYDLLHRTVDSQHVVGKQLLTVSGGM
Shanbavirus-A1        LMRAAGYVLAGCVED----------------ENLFLNLLDASVISIHHGVFEDYMLVGSN
Kunsagi-A             VMSVAADLFASLTED----------------PVVSRRYFDPIIDSIHEVGPYRVEVHGGM
Kunsagi-B             VMIQGARLLSNLTSD----------------PEVSMNFFWPIINSNHQVACYDVVVGGGM
Kunsagi-C             VMHRVAQMISNLTPE----------------PQFVMRLFQPILISTHIVGSNEIVVEGGM
Aquamavirus-A1        ILAAGVEVLACTSAV----------------PSYVRKLHAPIICSHHWHNNVVDLVLGGM
Passerivirus          AFDLIAAKLSQIITPLPG-----------IAEDAIVKYIRSISVSKHVFGDRAYLMTGGN
Sicinivirus           IFKLIGEELAKIIELPPS-----------IPPGSIQKYVQSIYLSKHVFGDHWYTMKGGN
Gallivirus            CFNHIGSRLQDIIEVTEE-----------CHPDIVPKYINSIRASKHVFGARAYVMIGGM
Pygoscepivirus        CFDVMAKHLARLIKPSPD-----------LPENCVQKYIASISYSEHVFGTRHYFLQGGN
Kobuvirus             VFTLVADHLTRITG-----------------DPRVGRYIHSIRHSHHIYGNRMYDMIGGN
Sakobuvirus           AFELIANELAKRIG-----------------DPRVIPYLRSVSNSQHVFGRQAYIMTGGN
Salivirus             LLSAIASRIAARSD----------------QPGRVLDYLSYTTTSYHVYDSLWYTMIGGN
Livupivirus           AFFVLGRHLANLIG-----------------DDEVRIYVEAIGVSKHVYGSAVVLMEGGM
Oscivirus             AFDLLSYHLERLIG-----------------DPRISKYISSIASSRHVYGNKIYLMLGGM
Ludopivirus           CFDLLAEHLSDLIQ-----------------HPAVIPYIKSISRSMHIFGDQAYELQGGM
Rafivirus             AFKRLAQCLRQIVN-----------------HPLAADYIEHLANTVHVYGSMEYMVRGGM
Danipivirus           AFEVLIDNVSDMSV-----------------DPDAGKMLRWACYSKHVYGAELYQIVGGM
Symapivirus           MYHLVADWMAAHGF-----------------SSDAVTALRTLSTTRRLYAGSIYTVDGTM
Dicipivirus           SFDALADLLCEFVE----------------NEDDVRRYISNIKNSYHAHDGNLYLIEGAM
Rosavirus             CFDALADLLAEFVE----------------GEEDVRRYISSLKTSFHHYKGKLWRLDGAM
Myrropivirus          SFRFLTQLMEQLVD----------------SPQ-VSAFINSICSSTHAWRGNLYNMQGAV
Pemapivirus           AFDVLSYVLSKFIDL---------------EDTEIKKFVDSIANSYHVWGGKLYNHVGGM
Hemipivirus           LFDSLAQVLEPCIE-----------------DPRAYPMIRAYAHSRIIQFDDIYQEDGGM
Tropivirus            AFDFLADMLQDLCD-----------------SNRVKSMINSVKDSIHIWKNEKFLITGAM
Megrivirus            AFLLMAKYLEPYFQ------------------HDVTPFLSAVATSKHVYGDKAYEMEGGM
Poecivirus            VFKMLASALQPWFE------------------ADVIKPLMSLAVSRHVYETEVYELHGGM
Ailurivirus           MFELVANEIFTPKNG--------------FHPRVR-DYLMSLAVSTHAYEEKRYLIEGGL
```

```
Mischivirus           MFELLIECFFTKENG--------------FSPAVA-PYLRSLATSKHAWMDKRYLIEGGL
Cardiovirus           MFRLLAEEFFTPENG--------------FDPLVK-EYLESLAISTHAFEEKRYLITGGL
Senecavirus           SFEALISHFFTVDNG--------------FSPALG-PYLRSLAVSVHAYGERRIKITGGL
Mupivirus             VFDVIKQEIFNSANG--------------FDPRVW-NFLDSLAESNHAYESRRFKLVGGL
Cosavirus             IFEILEQKFFSPELG--------------FDPRCS-LLLKSLAVSTHCYENKRLQIAGGL
Marsupivirus          LFEILAEDFFNEKNG--------------FDPRVK-QYLKSLAVSRHAYGEERYTLEGGL
Aphthovirus           AMNIMFEEVFRTEFG--------------FHPNAE-WILKTLVNTEHAYENKRITVGGGM
Hunnivirus            SFQCLK--IFLQELG--------------FDDVAL-KYVDSLCDSTHIWDDEIFKISGGL
Malagasivirus         SFHCLK--VFLKELG--------------FDEVAM-AFVDSLAVSRHIWDDEEYTLTGGL
Tottorivirus          SFEALK--IFLKELG--------------FDDVAM-KYVDSLGTSTHVYKNKQFVMVGGL
Teschovirus           MFCILK--HFLTTLG--------------YGTLQL-SYIDSLCYSKHHWDDETYRLDGGL
Bopivirus             VFQVVRDHVFNPENG--------------FDARAQ-AYIDSLCFSTHRFGDVEYTTDGAL
Erbovirus             VFQHLK-KLLSPANG--------------FDVRVQ-NYIDSLCYSKHQFGETYYECEGAL
Mosavirus             MFNLLAERFFTERNG--------------FDQQAVRIFLNGLSDSDHVYEGKHFRIRGGL
Torchivirus           MFDLVQKHFFNTENG--------------FDEKTG-KYLDSLSVSKHVYGREKFLTIGGL
Felipivirus           WFDALKMIFRKLGYS----------------ENDL-GLIDHVCFSKHIYKSTLYEVEGGM
Parabovirus           WFEALKKVLRQIGFS----------------ED---RLIDYICYSKHVYKDVEYEVVGGM
Sapelovirus           WFDALKVFLVKLGYG----------------EGAI-DAIDHICYSSHIFKDQYYVVHGGM
Boosepivirus          WFECLKIFLNELGFG----------------KEAE-ECIDYICNSVHIYKDKEYNVEGGM
Anativirus            WFEEVKFILRSLGFD----------------PQITDSMIDHICNSTHIYRNKEYDVEGGM
Rabovirus             WFKCLKMVLTNLGFK----------------DLGP---IDHIIYSKHIYKNIEYDVEGGM
Diresapivirus         WFECLKLLLIKLGYK----------------QEAL-NIIDHMCNSTHLFRNVKYVVEGGM
Enterovirus           WFRALELVLREVGYS----------------EEAV-SLIEGINHTHHVYRNKTYCVLGGM
Crahelivirus          MISRAVAVLCGLTPLD---------------ELRTSVLQRYYTQTRLRMRDHIFHIEGGM
Hepatovirus           MIREAGRIMSELSGTP---------------SHFGTALINTIIYSKHLLYNCCYHVCGSM
Gruhelivirus          IIMAAVRVLCRLAGLS---------------DDYCRAIAGTLAFSRNVVRNVRIWTNGGL
Tremovirus            MIYHAGRVLGQICGLD---------------PRLVDRIMEPIVNSVHQLGSMRYYVHGSM
Caecilivirus          MLEAAAQVLGSMSGLS---------------VNLTNSLFKPLIFSKHQFGSLIYHIIGSM
Rajidapivirus         WYQLMAEKLASFVPG-------------------FEQFKNEIVIRNHIYQNITYESFGST
Rohelivirus           MLREALRVISPEHQT---------------------SLAKTYEVTTRQYANIQYTISGSL
Fipivirus             VLTSACVLLGNLCGFP---------------DGVGYRLAEFVAYSKQIVGDRMYTINGAL
Harkavirus            FLQMVLWLLKKFTDPE--------------NASLLEWLWQPILRTKHVYMDEIYLVEGGV
Ampivirus             FYRQITKIINAWYRMFQVDKT-----HIEQECLARETLAFEIGHTLHLFGATLYTDDHGL
WenzhouPL-48          AFSFFQKVVRLYYEGAPE-----------EEHKVRDVLIHELQHTTQLIGSGLYQSGKGN
WenzhouPL-47          PPRQKETKEVPNSATRDQESTGRFEVGEASDAELIKQFECVKPIDYISILKERCDLYGEE
```

```
DERSV-SD609          CSGSPC--TTVLNTIVNLIVNYTVMFDYG-----------YSPSELYIIGYGDDTVISAD
WDALV                CSGSPC--TTVLNTIVNLLVNYTILFDYG-----------LSPSQTYVIGYGDDTVLSTD
Aalivirus-A1         CSGSPC--TTVLNTICNLLVNTTILLSEG-----------IQPDNFYIAAYGDDTIISVD
PBD-A1               CSGSPA--TTVLNTICNLLVNTTILLSEG-----------LQPDQFYIAAYGDDTVISID
QKE55028             CSGSPC--TTIINTICNLLVNYCVAFSYG-----------LSMDEVHIAAYGDDTLFSTT
Grusopi-A1           CSGSPC--TSILNSAVNILVISTMMMSYG-----------YDPKELRLLTYGDDCVISVP
Grusopi-A2           CSGSPC--TSILNSAVNILVISTMMMSYG-----------YDPKELRLLTYGDD-----L
Grusopi-C            CSGSPC--TSVLNSAVNVLVLSTMMMSYG-----------YDPKEIRLLTYGDDCVISVP
Grusopi-B1           CSGAPC--TSVLNSICNYLAIATVLISYG-----------YKDDDFKIYTYGDDCVVSVK
Avihepatovirus-A     CSGSPC--TTVVNSIVNQLACYTILAVLG-----------YDINQCYVVSYGDDCVLSVP
Avisivirus-A1        PSGSPW--TTMINTICNLLMCKTYLLDMG-----------HDLTKTYVVCYGDDCVISVD
Avisivirus-C1        PSGSPW--TTTLNTICNLLMCYTYLLDMG-----------HCWSETFVVAYGDDVVISAN
Avisivirus-B1        PSGSPL--TTLMNTVCNMLMCYTWAFYQG-----------YSCEEVFVAAYGDDVIISAK
Orivirus-A1          CSGSPS--TTLLNCVCNIFVHTAFALVYN-----------LEFK---VYCYGDDVIFSTK
Orivirus-A2          CSGSPS--TTLLNCVCNIFIHTAFALVYN-----------LDFK---VYCYGDDVIFSTK
Crohivirus-A         PSGSPC--TTVLNSVCNLIVSSTIADMCT-------------EGDFKILVYGDDLIIS-S
Crohivirus-B         PSGSPC--TTVMNSICNILVSASVALQTT-------------NGNFQCVVYGDDLILS-S
Parecho-B            PSGAPC--TTVLNSICNLLVCTYLAYEQSL------------DIEVLPIVYGDDVIFS-V
Parecho-C            PSGSPC--TTVLNSICNILVCRYLAYSQGP------------SVECLPIVYGDDVIFS-V
Parecho-E            PSGAPC--TTVLNSICNLLVCTYLAWEQDP------------TIQVLPIVYGDDVIYS-V
Parecho-A            PSGSPC--TTVLNSLCNLMMCIYTTNLISP------------GIDCLPIVYGDDVILS-L
Parecho-D            PSGAPC--TTVLNTVCNLLACEYASLKIG--------------AESLCVAYGDDLLFS-T
Parecho-F            PSGAPC--TTILNTICNLLMSRYCAILASSALE---------QDEVVIFAYGDDVVVS-T
Potamipi-A           PSGSPT--TSVLNSICNLLVVRTVALECGLED----------LDDMVIVTYGDDVLFS-T
Potamipi-B           PSGSPC--TSVLNSVCNLIVVKTACLSAGVEK----------PEDIQIVTYGDDVLMT-I
WCP                  PSGSPC--TTVLNSLCNLLVVRYAMATVGFS-----------FEEVILTTYGDDVIGS-A
Limnipi-B1           PSGSPC--TTVLNCLCNLLVLEYAFLEIFGLGERDDQFRKHVD-DHLIVVYGDDCIVAHN
Limnipi-B2           PSGSPC--TTVLNCLCNLIVLEYAFLEVFGLEELSMKDHERVNSDYLTVVYGDDCVIAYN
Limnipi-C            PSGSPC--TTILNCICNLLVLEYSFLDTFGIDYQFHFDGDYTSRDFLTVVYGDDCIVAYN
Limnipi-A            PSGSPC--TTVLNCICNLLVLDYAMLVHHDVYEDG---VGLPQCDYLSVVYGDDCVVAYN
Limnipi-D            PSGSPC--TSVLNTVCNLLMLEYALER-------------EVQGAWMTFAYGDDCVVAHD
Pasivirus-A1         ASGSPF--TTIVNTACNILANMTVLE--------------EEGVEYTLVCYGDDILVSTS
Pasivirus-A3         ASGSPF--TTIVNTACNILANMTVLE--------------ETGVEYTLVCYGDDILISTS
Pasivirus-A4         ASGSPF--TTIVNTACNILANMTVLE--------------EEGVEYTLVCYGDDILVSTS
Pasivirus-A2         ASGSPF--TTIVNTTCNILANMTVLE--------------EEDVEYTLVCYGDDILVSTS
Shanbavirus-A1       PSGTPF--TTVLNCACNLLVVEYYMLGL------------AQQIPYVAITYGDDLILSTQ
Kunsagi-A            PSGCAV--TTLLNSVCNVLMCSYAVLLQDPDM------------DFQVVAYGDDNIVSTA
Kunsagi-B            PSGCPI--TAVYNSVCNVIMMSYALLKCNPDV------------HFITFAYGDDNVVSVD
Kunsagi-C            PSGCPI--TTIMNTLCNVVMTHYCMLLLDPNS------------DFWPVAYGDDLILSTR
Aquamavirus-A1       PSGAPC--TSVLNSIVNVLMARYICALMDID-------------YPVMVAYGDDNVVSFD
Passerivirus         PSGCVG--TSILNSMINNSVLISAFLTHPQFN----------PAEMRILTYGDDVLYATT
Sicinivirus          PSGCVG--TSIFNSMVNNISLLSAMLTHPDFD----------SSAYRILCYGDDVIYATV
Gallivirus           PSGAVG--TSIFNSMINNICVLSALISRPDFV--------PTPNNYRILAYGDDVLYACT
Pygoscepivirus       PSGCVG--TSIFNSIINNMAILSVMLHHHVPI--------HDPTSYQILSYGDDVIYSSY
Kobuvirus            PSGCVA--TSILNTIINNICVLSALIQHPDFS----------PSRFHILAYGDDVIYATE
Sakobuvirus          PSGCVG--TSCWNSIINNCVLLSALMSHPDFD----------PDRYRILAYGDDVIYAHE
Salivirus            PSGCVG--TSILNTIANNIAVISAMMYCNKFD--------P-RDPPVLYCYGDDLIWGSN
Livupivirus          PSGCVG--TSILNTILNNAFIISALSMHPDFN----------AEAYRILAYGDDVVYAHN
Oscivirus            PSGCVG--TSILNTICNNCFVLSALLEHKDFD----------INQYYIIAYGDDVVYATN
Ludopivirus          PSGCVG--TSIFNTMINNCIVLSALMEHPDFD----------PQSYRILAYGDDCLYATN
Rafivirus            PSGCVG--TSIFNTMLNNCFILSALITHPDFN----------PEQFGMMAYGDDVIYATN
Danipivirus          PSGMSG--TSVFNTVLNNTLILSALATSPLFN----------PDDYAILAYGDDVLYANR
Symapivirus          ASGVSG--TSIFNSIFNNCFILSALMDHPDFD----------PDTYRVLAYGDDVIYAHD
Dicipivirus          PSGCAG--TSVFNCLINAMLCFSCFMDLEPE---------MDPFEPLLIAYGDDILVSSD
Rosavirus            PSGCCG--TSVFNSLINAMLLFSAFSQVCPD---------LRADEPLLVAYGDDVLVGTD
Myrropivirus         PSGCVG--TSVFDSVINVTLMMSAMISLG-----------LSPTEPQFLAYGDDLLVATN
Pemapivirus          PSGCVG--TSIFNSILNNIFVQSAFISINPD---------YVPDEVLILTYGDDVVYGTN
Hemipivirus          PSGCVG--TSIFNSIMNSLVVLSAFYSLDPKT--------DHIVEAIVLTYGDDVIFGTN
Tropivirus           PSGCVG--TSIFNSFINIMVFQSAL--LENPE--------YSPEDTYFLTYGDDLLVATD
Megrivirus           PSGCVG--TSMFNCINNSAFIVSALIALKIS-----------PDSCKWICYGDDVIISTD
Poecivirus           PSGCVG--TSIFNSIHNAAFIASALIAVGVD-----------VEQCSWICYGDDLLLASD
Ailurivirus          PSGCSC--TTVLNTVMNNIIIRAALKMTYKN---------FDSKDVTILAYGDDLLVGTE
```

```
Mischivirus           PSGCSA--TSVLNTVMNNIIIRALLSLTYSN---------FHPEDVAVLAYGDDLLVASD
Cardiovirus           PSGCAA--TSMLNTIMNNIIIRAGLYLTYKN---------FEFDDVKVLSYGDDLLVATN
Senecavirus           PSGCAA--TSLLNTVLNNVIIRTALALTYKE---------FEYDMVDIIAYGDDLLVGTD
Mupivirus             PSGCSS--TSVINTVINNVVIRTGLAMVYQN---------FDFYDVEILAYGDDLLIATD
Cosavirus             PSGTAG--TSVLNTVINNIIFHGALYHTYTN---------FERDDISMLAYGDDIVVASK
Marsupivirus          PSGCSA--TSILNTVINNIIIRTVLLMSYVN---------IDWEHVEFLAYGDDLLVATN
Aphthovirus           PSGCSA--TSIINTILNNIYVLYALRRHYEG---------VELDTYTMISYGDDIVVASD
Hunnivirus            PSGCSC--TSIFNTILNNIVVRSLVPEVYD------------GEFQILAYGDDLVLCSQ
Malagasivirus         PSGCAC--TSIFNTILNNIIMRGAISTLTT------------EPFQLLAYGDDLVICAY
Tottorivirus          PSGCAC--TSIFNTVLNNIILRG-LAKFKG------------YEIKMLAYGDDVIVTSG
Teschovirus           PSGCSG--TTIFNTIMNNIVARAAASYAAD------------GPVGILCYGDDILVSSP
Bopivirus             PSGCSG--TTVVNNMINNIIVRAALRMVYTD---------WDDSDIGVVAYGDDLLLGCN
Erbovirus             PSGCSA--TSILNCLMNNIVLRAAAYDVFTN---------YEEGDLAFLTYGDDVLLCAN
Mosavirus             PSGCPC--TSILNTVINNIIVRAAIIGAYQIDT-------VDFQKFRMLAYGDDVVYATP
Torchivirus           PSGCSC--TSMLNTVFNNIIIRAAILSCYEG---------VDWSDFKMLAYGDDVVYSSR
Felipivirus           PSGCSG--TSILNSIINNLIIKTLVLRTYKG---------VDLDQMKILAYGDDVIVTYP
Parabovirus           PSGCSG--TSIFNSIINNLIIRTLVLRCYKG---------IDLDSLKIIAYGDDVVCSYP
Sapelovirus           PSGCSG--TSIFNSIINNLVVRTLVLKCYKG---------INLDLLRILAYGDDLLVSYP
Boosepivirus          PSGCSG--TSIFNSIINNIIIRTLILDVYKN---------IDLDYLRVIAYGDDIIASYP
Anativirus            PSGCSG--TSIFNSIINNIIVRTLVLDTYKG---------IDLDQLRILAYGDDLLVSYP
Rabovirus             PSGCSG--TSIFNSMINNIIIRTLLLDAYKG---------IDLEQLKMIAYGDDVVATYP
Diresapivirus         PSGCSG--TSIFNSIINNIIIKTLILDAYKG---------INLDFVRILAYGDDVLASYP
Enterovirus           PSGCSG--TSIFNSMINNIIIRTLLIKTFKG---------IDLDELNMVAYGDDVLASYP
Crahelivirus          QSGAPC--TSLINSVINVTNLFWALQRMT-GWSLPAI-----RENYRIIVYGDDVMMAPK
Hepatovirus           PSGSPC--TALLNSIINNVNLYYVFSKIF-GKSPVFF-----CQALKILCYGDDVLIVFS
Gruhelivirus          PSGTPC--TSLLNSISNLLILRYVLKKTYPALSFVDL-----DQCFRLLTYGDDVVIVVN
Tremovirus            PSGTPA--TSVLNSIINVVNICHVLCALE-KISVFEV-----FKLFKILTYGDDVLLCIK
Caecilivirus          VSGSPC--TSILNSIVNLTNTRWAIWQAT---GVMPD-----QQEQEIIVYGDDVLLLEV
Rajidapivirus         PSGMVG--TSIINSIINFLNFTSASWRTG-------------HATPTFISYGDDFLCCFK
Rohelivirus           PSGMPA--TSLVNSLINTLNTIYVFREMGFS-------FQQFLDHVCLLTYGDDIVMGVS
Fipivirus             PSGAPF--TSILGSMCNFLMLLYTLSRATGQR------MSAFQEWCHLCTYGDDVLVFVH
Harkavirus            PSGMPC--TTVVNTLVNMMIVVYVWSVTGHDVCD-------LDSQMTFCCYGDDLVLSTS
Ampivirus             PSGVPGGFTTIFNIMVNMMLARITYIRTGLHVS-------TYRTHTRNIFLGDDGLHAVM
WenzhouPL-48          KSGNYL--TDVFNSITNVWAWMTSFHRVFTNELGKMPTLDDWFENVVLFTHGDDCILSLK
WenzhouPL-47          VTYSFKRLVSPDNAPLYECVCMIGARRFSAVEIGKKK-AKRTASYIMLLAISDSVYQADI
                       :      .   .                                      .*.
```

```
DERSV-SD609          -----------RKVAISDIASKYKKYFGMNVTSAAKTD--QIGWQPKEKLEFLKRSTALF
WDALV                -----------EVFDCSDIVEKYRDYFGMNVTSADKTS--EIKWEKKENVEFLKRTTGLF
Aalivirus-A1         GLS-------SSLPDPKVMQQKYKEWFGMTVTSADKGS--EITWDTRNHVQFLKRRPGFF
PBD-A1               SWP-------SRLPDTSIMQQKYKEWFGMTVTSADKGD--VIKWDTRNNTQFLKRRPGFF
QKE55028             -----------RCFDVEGIEQRYMDWFGMKVTSSDKVS--KITWQGKSQSNFLKRSPTVL
Grusopi-A1           -----------YKVDISDFKKRLKNYFGMTVTNFDKTE--EFKWLSRGEISFLKRTPAIL
Grusopi-A2           -----------YLIHI--------------------------------------------
Grusopi-C            -----------YKVDVSDFKWRLKNYFGMTVTNFDKTE--EFKWLGRGEISFLKRTPTIL
Grusopi-B1           -----------EKTSMEDLEWRFAAYFGMTVTNFDKSS--EISFLPPNQITFLKRSPLEL
Avihepatovirus-A     -----------EVRDISKLSHYFKLFFGMTATASDKES--DITWLAPMEIEFLKRTPAFL
Avisivirus-A1        -----------QCHKLEGIEQWFMDKFGATVTPEDKSG--KINWRFKNKLKFLKRTPMQL
Avisivirus-C1        -----------IKHNLEGIENWFKTKFGATVTSSDKQS--KITWTTKNNMEFLKRRPKEL
Avisivirus-B1        -----------KKSNITDIVQCFKSWFGASITPAIKEG--DISWAPKHQVVFLKRRPKQL
Orivirus-A1          -----------QQFDPRDYVEFMKEKFGMTVTSAQKTA--EIAFVPPAEIEFLKRKPTIF
Orivirus-A2          -----------QWFDPSVYVSFMAEKFGMTVTSAQKTS--DITFVPPYEIEFLKRKPVSF
Crohivirus-A         T----------APLDCDRFKTLVELHYGMEVTPGDKGD--EFKVKDREQVSFLKRVTRKF
Crohivirus-B         T----------EPLDCEGFKQCVKEQFGMEVTPGNKAE--IFQCSEPGAVRFLKREPKNF
Parecho-B            S----------SPLDAEYLVQSAAQNFGMEVTSSDKSG--PPKLLKMDEIEFLKRTTKFF
Parecho-C            S----------EEIDASCLVEQARREFGMEITSSDKSA--VPTLLNLEEVEFLKRTTRFF
Parecho-E            D----------QPLDMERFVSQAKNCFGMDVTNTDKTP--IPTLVDFDEIEFLKRRTKIF
Parecho-A            D----------KEIEPEKLQSIMADSFGAEVTGSRKDE--PPSLKPRMEVEFLKRKPGYF
Parecho-D            P----------VPINPVHVLQIWKCDLGLTATGPDKTE--HVPAVCPLDIEFLKRTPKFF
Parecho-F            E----------EEMDTTVFVDSMYKHFGMTVTDANKKL--GINKLDKNEISFLKRKPKFF
Potamipi-A           K----------HEIDFSEAPAMIKEMFGMDATSADKQS--TNFDVSPEEATFLKRNFRLF
Potamipi-B           D----------YDFCDDALPEIIHSQFGMEATSANKLS--KTLKVSPADATFLKRSFRLF
WCP                  T----------QRVSAESVVRTIKDVFGMEATSADKKS--LDLTVHPNEATFLKRRFRHF
Limnipi-B1           A----------EEELGPAFKETIFTSFGMEVTPASKVG--DVFEVPLDEVEFLKRKFFKI
Limnipi-B2           G----------PD-IGLGLKQCIGDSFGMEVTPATKTG--DEFCVDLDEVEFLKRTFFKL
Limnipi-C            G----------PE-VGAALAEVVKNAFGMEITPASKVG--EEYNVEIQDIEFLKRTFFRL
Limnipi-A            G----------MR-MGLDFAQTIEDTFGMTVTPASKLG--DHFNVELHEVEFLKRKFMAF
Limnipi-D            G----------AQVDADTFVNSMKTAFGVEVTPADKGE--GDIYVPIERVQFLKRTFVFS
Pasivirus-A1         D-----------PISVNDYVDKMGQYFGFTVKLDGDYP------KTKNNFTFLKRRTTYW
Pasivirus-A3         D-----------PISVTDYVDRMRQYFGFTVKLDGDYP------KNKLNFTFLKRRTTYW
Pasivirus-A4         D-----------PISVDDYVNKMRQYFGFTVKLDGDYP------KTKTNFTFLKRRTTYW
Pasivirus-A2         D-----------PISVDDYVDKMRQYFGFTVKLDGDYP------KTKTNFTFLKRKTTYW
Shanbavirus-A1       E-----------PIDARDFQRMLKLEFGMTVTPSDKDST-EFVNKSPMEVEFLKRKPKHH
Kunsagi-A            E-----------PLDVPAFRSVLASDFGMVTTSADKSEE-CYQVPPEQ-VDFLKRRLRWT
Kunsagi-B            Q-----------EISLEKFTSILREEFGMEPTAPDKTLN-YSFVPPSE-VTFLKRTLRAT
Kunsagi-C            K-----------PIDTELYCKIMNEEFGMILTGADKTTT-VQAVPPMS-VDFLKRRMHYT
Aquamavirus-A1       E-----------EIDIERMVSLYKTEFGVTATNHDKTP---VPRPMAN-PVFLKRRLRFN
Passerivirus         -----------PTIHPSFVKSFFDQNTTLIVTPATK-AGDFPDHSTIWDVTFLKRWFVPD
Sicinivirus          -----------PSIHPSFIADFYHKHTLFKVTPADK-GNTFPETSSIHDVTFLKRHFVPD
Gallivirus           -----------PDFHPRDLKAFYDKWTPLQVTPATK-EGDFPNSSSLSDVTFLKRWFVPD
Pygoscepivirus       -----------PNLHPTLLKEYYDKHTTFKVTPADK-TDVFPETSTIYDVTFLKRSFVPD
Kobuvirus            -----------PPIHPSFLREFYQKHTPLVVTPANK-GQDFPPTSTIYEVTFLKRWFVPD
Sakobuvirus          -----------PTIHPSFVADFYRKHTSLVVTPASK-AGSFPEHSSILTVTFLKRHFVPD
Salivirus            -----------QDFHPRELQAFYQKFTNFVVTPADK-ASDFPDSSSIFDITFLKRYFVPD
Livupivirus          -----------PPIPPGFIKEFFDRNTPLEVTPASK-AGDFNLDSTILDVTFLKRHFVPD
Oscivirus            -----------PPISPTFIKSFYDRYTPLVVTPADK-SDKFNESSTIFDVTFLKRSFVPD
Ludopivirus          -----------PPIHPRFVKKFFDEHTNFLITPPSK-SGDFPDFSDLSTVTFLKRWFEPD
Rafivirus            -----------PTIHPSFIKQFYDEHTPLTVTPASK-DGMFPDTSTIHDVVFLKRWFYPD
Danipivirus          -----------NTINPSFVADFLGEKAGMVLTPASK-AGTFPEHSSIRDVTFLKRSFVPC
Symapivirus          -----------PPIDPTHIKTFFDEHTCLKVTPASK-MGEFNTRSTIFDVTFLKRRFVPD
Dicipivirus          -----------HDLFPSRVSEWMKKNTTFKITPADK-GEIFNDDSDVSDVRFLKRLFVED
Rosavirus            -----------QPLFPSKVAEWVNTHTTFRITPADK-GDVFNDESDIHCVQFLKRHFTPD
Myrropivirus         -----------QEFCLTDLTDWITTQTPFKITPATK-DGCYKLDSDIHDVTFLKRRFVPD
Pemapivirus          -----------QKLLPQEISHFYKYNTTLTVTPGNK-LSDWKEKSSIHDVTFLKRWFVPD
Hemipivirus          ----------SVELHPRKVADWIMENTPFQVTCPEGVWKDTP---CVFDLKFLKRKFVLD
Tropivirus           Y---------SIDIAS--VAAFLHKHTPFQVTPADKNAKEFPVDTTVYDVTFLKRYFVND
Megrivirus           -----------EKALSRRIADFYSRNTNLKVTPACK-SGDFPEESTFYEVSFLKRFFVHD
Poecivirus           -----------VECLAQKIADFMNTTTCLEITPADK-GEKFRDDSSIFDVTFLKRKFQPD
Ailurivirus          -----------YDLDFNKVKEKL-ATVNYTITPATKEGT-FPLHSSILDVQFLKRKFEPY
```

```
Mischivirus          -----------FVLDFNRVRATANEHTLYKLTTANKAPD-FPETSTLLECQFLKRKFVLH
Cardiovirus          -----------YQLNFDKVRASL-AKTGYKITPANKTST-FPLDSTLEDVVFLKRKFKKE
Senecavirus          -----------YDLDFNEVARRA-AKLGYKMTPANKGSV-FPPTSSLSDAVFLKRKFVQN
Mupivirus            -----------FELDLNLLKSRL-ETIGYKITPANKGQA-FKSVMQIGEAQFLKRKFVID
Cosavirus            -----------FELDLVMVKAFM-NRIGYKITPADKSDE-FRP-KCMDDICFLKRRFVKV
Marsupivirus         -----------YAFDFGHVASHA-KTIGYKMTPAAKDGD-FQAETTWDDVMFLKRKFEMD
Aphthovirus          -----------YDLDFEALKPHF-KSLGQTITPADKSDKGFVLGHSITDVTFLKRHFHMD
Hunnivirus           -----------EIFPVEKYKEIVEEMTNYRITPASKSGT-FEWT-DLSGVVFLKRYFYKD
Malagasivirus        -----------EKFDLAALRDFFSENSLYRITPATKGGE-LSWG-ELSDMRFLKRGFVLD
Tottorivirus         -----------ECFDFIDFKTTLEQGTNYKVTTAAKDEL-FAWNTDIGGVMFLKRYFKRD
Teschovirus          -----------EKFPVSDWLEFYSK-TPYKVTAADKSEQ-IDWR-DITQCTFLKRGFVLD
Bopivirus            -----------TKLDLTKLADAF-KQLGYTVTPADKNGV-FNPDSTIDSVTFLKRRFVRD
Erbovirus            -----------QPLPLERFRKSL-AKLGYTITPADKSSF-FPEVSTLADVVFLKRQFKPD
Mosavirus            -----------QPIKPQDLADWLHANTNYKVTPASKAGT-FPEESTIWDVTFLKRSFKPD
Torchivirus          -----------DPILPGKIANWLHQNTTYKLTPANKTNV-FPTESKIEDVTFLKRKFVTD
Felipivirus          -----------FQLDASIVAEEG-KLFGLIMTPPDK-TSTFN-ETTWDTVTFLKRKFVPD
Parabovirus          -----------WPIDAQQLADEG-AKFGLTMTPADK-SASFN-EVTWENVTFLKRRFVPD
Sapelovirus          -----------FPLDPAVLADAG-KELGLTMTPADK-SDSFSGCSKLTEVTFLKRSFVFD
Boosepivirus         -----------FPLEASCLAEAG-SDYGLVMTPPDK-SSEFK-QIEWDEVTFLKRKFMPD
Anativirus           -----------FPLDPEALARAG-KVYGLKMTPADK-SEHFDGPKKIWEVTFLKRGFKPD
Rabovirus            -----------FPIDAASLADCG-KRYGLKMTPPDK-GSEFN-NVTWENVTFLKRRFKPA
Diresapivirus        -----------FPINPGELAKCG-VRYGLTMTPADK-AGEFS-VKNIYEVTFLKRRFVPD
Enterovirus          -----------FPIDCLELARTG-KEYGLTMTPADK-SPCFN-EVNWGNATFLKRGFLPD
Crahelivirus         RG--------ISVPRIDLLQG----LFDGLGMKCTAGDKGCVRLVKVLDLTFLKRVVKID
Hepatovirus          RD--------VQIDNLDLIGQKIVDEFKKLGMTATSADKNVPQLKPVSELTFLKRSFNLV
Gruhelivirus         KN--------QAVD--DRLPKALSNWLCLMGFQVTGADKGPVQLRPITEVQFLKRGLRFE
Tremovirus           K---------EYLDQKSFPLSSFVQGLEELGLSPTGADKMEVKVTPVHKMSFLKRTFYVD
Caecilivirus         E--------------SEIPLEDLEQAFKQMGMQVTSANKSALRRVSLSEVTFLKRGFRVD
Rajidapivirus        K-----------EPNFGKIIDNINTETDFKVTSATKGDY-VVGSGHISDVTFLKRWFCPD
Rohelivirus          ----------EELYKEINLDHLLLGYKSLGLKPTAGDKSDKLSWKDVDDFYFLKRRSRVD
Fipivirus            P---------EVVVNADALAHEMYEVHGVTCTDATDKR-LPPQLRELQNVTFLKRGFRRC
Harkavirus           D-----------DDFTFEQFSRVAQDLGFEATNACKDG---VVVDKVEDLTFLKRGFRCD
Ampivirus            RSEDPRINE-ALLKYNRIELARVASEIGMTVTMPDKIS-ALTPFDTFEEISFLKSSFLDN
WenzhouPL-48         R------------VITPQKLLDEIRALGFLITSADKSGE-DIKYENVDGLTYLKSGFRRS
WenzhouPL-47         DPAAAEPSAPRNPMPPVAVASPGSLAAGQTVGTIGARVEVTEQNFVPINTVTVQPDAQSN
```

```
DERSV-SD609           PHT---TKIVGKLDLKNMVGHLDWTSG-------TFQEQLNSFYLELVLHGQEIYDKVRN
WDALV                 PGT---AKMVGKLNLENMLGHLDWTTG-------SFQSQLDSFYLELCLHGQQVYDGIFE
Aalivirus-A1          PGT---QKVVGVLDLESMMEHIAWTKG-------SFQDQLNSFYQELVLHGEQVYMTVRQ
PBD-A1                PGT---QKVVGVLDLESMMEHIAWTKG-------SFQEQLNSFYQELVLHGENVYMTVRQ
QKE55028              YGT---SKIVGALDIDSMMDHIQWTRG-------EFQSQLESFYLELCLHGPTVYGQIRS
Grusopi-A1            EGT---TKLVGALDLDSMREKIQWTRS-----LNDFSSQLESFQLELALHGRGVYEKEIQ
Grusopi-A2            ------------------------------------------------------------
Grusopi-C             DGT---TKLVGALDLSSMKEKIQWTRS-----LNDFSSQLESFQLELALHGKEIYEREIQ
Grusopi-B1            YDT---GKIVGALDLSDSHEKIQWMKS-----PETFEQQLDSYLLEVAIHGEQIYNQTVE
Avihepatovirus-A      PGT---RKIIGVLDKEVLEGKIQWCKG-----PEAFKQQLDSFLLEAALHGEEYYHEVTS
Avisivirus-A1         DWI---PKIVGALDIDSMMDRIQWTKG-------HFQEQLNCFYYELALHGEDTYNEARK
Avisivirus-C1         EFL---PKIVGALDLDNMLQHLEWTKG-------HIQDQLNSFYLELALHGREKYEEIRA
Avisivirus-B1         DFA---PKIVGALDLQNMLDRIQWTTG-------DFQSQLNSFYIELALHGRETYNSVRA
Orivirus-A1           QG-----VTVGALSLDSLEHKIQWCRG-----LEAYKQQLKSFATELALHGRDQYTLLTS
Orivirus-A2           SG-----VTVGALSLESLENKIQWCRG-----LDAYKQQLRSFATELAFHGRSQYELTTD
Crohivirus-A          P---GTNYRVGALDLDTVKQHLMWCKS-----YSSFKQQLDSALMEVAMHGEETYNGFLT
Crohivirus-B          P---GTSFLVGCLDYENIKQHIMWCKS-----LDDFKKQLDTACMELVLHGRECYEEFVS
Parecho-B             P---GSTYKVGALSLDTMEQHIMWMKN-----LETFPEQLVSFENELVLHGKEIYDDYKN
Parecho-C             P---GTTYKVGALNLSTMEQHIMWMKN-----LSTFPSQLQSFENELALHGQHVYESYQN
Parecho-E             P---KTTFRVGALNLDTLEQHIMWMKN-----LDTFPEQIVSFENELSLHGRDVYDFYKE
Parecho-A             P---ESTFIVGKLDTENMIQHLMWMKN-----FSTFKQQLQSYLMELCLHGKDTYQHYIK
Parecho-D             P---NSGFIVGALDLDNMLQHIMWSHS-----SEAFLQQLSSFENELVLHGEHVYNEVQS
Parecho-F             P---GTQFFVGQLDLSEMKQNIMWMHG-----MKEFEQQLTSFENELVLHGQEVYERTIQ
Potamipi-A            P---DTLFVTGVLTLDTMLQKIQWCRG-----LNEFKQQFESFTQELVLHGPHVYHQVMS
Potamipi-B            P---DTQFVTGVLDLENMMQKIQWCHG-----KEAFIQQWTSFTQELVLHGRETYERVIR
WCP                   P---GTRFVTGQLDLDSMLQKIQWCHG-----LEEFKQQFESFTQELVLHGEETYAKVTQ
Limnipi-B1            ATVHSDRIAMR-LSVDTIRQSLMWMRS-----SKTFDDQVYSLAIELSAWGEETYDREFA
Limnipi-B2            STAKYDRYAMR-LSLTTIEQSLMWMRS-----ERTFDDQIFSLAVELSAWGKSEYARIFT
Limnipi-C             RGQRDDRIALR-LSLTTIFNSLMWMRN-----RKTFADQVFSLMVELSAWGREQYDLVVR
Limnipi-A             ETEEGYKVAILALNENVIVQHLMWMRN-----LTTLPQQIQSLMMEYAAYGKEKYDKLRD
Limnipi-D             P--EFNRFVGK-LDLDVIKQALMWTRN-----QHTFDAQMQSLSVELAAHGEEIYNEVRE
Pasivirus-A1          N-----GIPVGAMEVGLLLQHLGYCRS-----VSVFQDQLNSASIELALHGPETYNVLRS
Pasivirus-A3          N-----GIPVGAMEVDLLLQHLGYCRS-----VGVFQDQLNSASIELALHGPETYNELRS
Pasivirus-A4          N-----GIPVGAMDIDLLLQHLGYCRS-----VGVFQDQLNSASIELALHGPETYNELRS
Pasivirus-A2          N-----GMPVGAMDVDLLLQHLGYCRS-----LGVFQDQLNSASIELALHGPETYNELRS
Shanbavirus-A1        TS----DTIIGVLSLENMLQHIMWCKG-----LDEFATQTISFQIELAAHGEATYNEVR-
Kunsagi-A             S---DFPVPVPVLPLDSMLSRLCWCKG-----PHEFRDQLISFSYELGFYGQEVYHRVFS
Kunsagi-B             P---EYPLPVPVLPLDSMLSRICWCKG-----KREFIDQLRSFVTELALFGRETYTTVQA
Kunsagi-C             P---EFPLPVPVLPLDSLLSRICWCRG-----ETEFKQQLESFSYEVALYGQSVYERIRV
Aquamavirus-A1        P---DLNIQFPVLPLGEMIDRMCWTRG-----PEHLSDQTFSFAIELAGYGKQVYTHIRD
Passerivirus          EER--PWYIHPVIEPATYEQSVMWTR------GGDFQDVVTSLSFLAHHAGPTNYDAWAT
Sicinivirus           ERF--PTYIHPVISPETYQQSVMWTR------GGPFQDVITSLCYLAHHAGPNNYQDWCD
Gallivirus            ETI--PYYYHPVIEPDTYEQSVMWSR------GGEFQDTVTSLCFLAHHAGPRNYTNWIA
Pygoscepivirus        ETI--PYYIHPVIEAATYEQSIMWSR------GGDFQDTVTSLSFLAWHAGEDKYNQWVD
Kobuvirus             DVR--PIYIHPVMDPDTYEQSVMWLR------DGDFQDVVTSLCHLAFHSGPKTYAAWCM
Sakobuvirus           ETY--PMYVHPVIDPETYRNSVMWTR------GGPFQCQLDSLSYLAHHSGPNNYQAWVD
Salivirus             DIH--PHLIHPVMDEQTLTNSIMWLR------GGEFEEVLRSLETLAFHSGPKNYSAWCE
Livupivirus           PKM--PVYIHPTIDPAVYQQSVMWVR------DGDWQDTVTSLAQLAFHAGPNNYKNWVT
Oscivirus             PTK--PWLIHPLIDPVVYEQSCMWVR------DGDWQDTLDSLCQLAFHSGPKTYARWVE
Ludopivirus           DKR--PHLIHPVIDPAVYENSVMWTR------GGDLQDLVDACCNLAYHAGPKNYADYTQ
Rafivirus             LMH--PGLVRPIINPEVYYQSVMWMR------DGDIQDTITSLSYLAFHAGPNNYVNWIK
Danipivirus           PTA--PVCWLAPIDPETIRQSCMWLR------KGALGDVLTSLSWLLFHHGQTAYDEFCR
Symapivirus           EST--TVYIHPVIDPDTYEQSVMWLR------EGEFSDVVRSLSYLAWHAGETAYRAWSD
Dicipivirus           P-I--CELIHPVIETETLEPSLNWCH------EGEFETKVDAISMLAFHHGPEYYRDWCK
Rosavirus             PDF--PALIHPTIDPDTYEQSVMWQR------AGDFQETVNSLAQLVFHRGPRSYSRWCE
Myrropivirus          PHF--KCLIHPMIDPSVYQQSVMWVR------SGEFQDTVRSLSDLAYHAGPKSYAAWAN
Pemapivirus           PEH--RYLIHPIIDPSVYEQSAMWVR------GGDFQDTLTSLCQLAFHSGPRNYDAWVK
Hemipivirus           PRF--PPLVHPQIDAQVYQQSVMWTR------SEDFQQVLDCLALLAYHGGPITYQIWKE
Tropivirus            PDC--PVLIHPLIKPEVYQQSIMWTR------GGPFQSMIQSLAELAWHSGPKTYYAWCG
Megrivirus            SHY--PQLIHPYMPLEHLEQSSMWQT------DGEYQQKLDSLAQLAFHAGGPDYRKFCD
Poecivirus            TLY--PHLIHPRMDEDVLEQSVMWQT------DGDFEQKFFSLCFLAFHSGRKWYEQFIE
Ailurivirus           IIH--GFIFRPVMSEKNLEAILSYYK------PGTLQEKLQSVAQLAVHCGIDTYDRLFK
```

```
Mischivirus         SVR--NFIWRPVMDTTNLQTMLSFYK------PNTLSEKLLSVAQLAFHSGYHTYEQLFE
Cardiovirus         GP-----LYRPVMNREALEAMLSYYR------PGTLSEKLTSITMLAVHSGKPEYDRLFA
Senecavirus         NDG----LYKPVMDLKNLEAMLSYFK------PGTLLEKLQSVSMLAQHSGKEEYDRLMH
Mupivirus           EGR--PFLIRPVMEKENLAAMLSFYR------PGTMTERLESIVQLAVHSGKEVYDWLFK
Cosavirus           AG-----VWAPVMETENLEAMLSWYK------PGTLNEKLQSVSRLAHFSGRDVYDHLFK
Marsupivirus        DDT---VLVRPVMSTRLLKDLMAWSR------PGTLQDKINSVALLAFHLDEGDYNDIFE
Aphthovirus         YGT--G-FYKPVMASKTLEAILSFAR------RGTIQEKLISVAGLAVHSGPDEYRRLFE
Hunnivirus          GL-----LVRPVMTYKNLHNILSWAR------AGTVQEKLLSVARLAQHRGEQDYKALME
Malagasivirus       GV-----IYRPQMTKENLHNILSWAR------AGTLQEKVLSVSLLAVHSGKQVYEELFE
Tottorivirus        GI-----LYAPVMSCVHLHNILSWAR------AGTIQEKVTSVAGLAVHCTKDQYYDLFR
Teschovirus         GS-----LVRPVMEEQHLAELLKWAR------PGTLQAKLLSIAQLAFHLPRSAYNRLML
Bopivirus           SLY--PYLIHPVMDAELLANLLKWQR------AGEFQQKVISIAQLLMHSGEADYESVME
Erbovirus           EEF--PFLFKPVMDVRNLQDHLCYAK------PGTLREKLLSTTDLAVHLGPLEYARLFR
Mosavirus           EDH--GHLIRPQMAVGNLRQMLSFMR------PGTFPDKVRSVAGLAVHCGEEVYNQLAD
Torchivirus         -----GVLVRPVISRTNIENMLAWKR------NGEFGDKIRSVAGLAFHLGEDEYNDIFL
Felipivirus         HQF--PFLIHPVFPMSEIYESIRWTRN-----PQQTNEHVDSLCRLAWHCGEEEYNDFIA
Parabovirus         EVF--PFLIHPVFPMSEVHESIRWCRS-----AAHTQEHVSSLCYLAWHAGEKEYNEFLD
Sapelovirus         EQF--PFLCHPVFPMSEIHESIRWTRS-----AATTQEHVTSLCLLAWHNGKEVYEEFCE
Boosepivirus        ERY--PFLIHPVFPMSEIYESIRWTKS-----AANLQNHVTSLCHLAWHNGKVIYDDFIG
Anativirus          SRY--PFLIHPVYPMDQVYESLRWTRK-----PSETQQHVRSLCELAWHNGEEEYNNFLS
Rabovirus           KHY--PFLIHPVFEQQELLESLRWTRN-----PAATQEHVRSLCELAWHSGRKSYEEFCN
Diresapivirus       EVF--PFLIHPVFPIQEIEESVRWTRN-----ATATQEHIYSLLSLLWHSGEQQYNQFVD
Enterovirus         EQF--PFLIHPTMPMKEIHESIRWTKD-----ARNTQDHVRSLCLLAWHNGKQEYEKFVS
Crahelivirus        EDG----RIKACLDPESIWGMLAWRRE-----GADFGVNVDTASWYAFQHGKIFYETFSA
Hepatovirus         ED-----RIRPAISEKTIWSLIAWQRS-----NAEFEQNLENAQWFAFMHGYEFYQKFYY
Gruhelivirus        QG-----VIHAVMSPKTIRSMLAYKRN-----NARLVDNIRVAAGFMYHHGQDVYRQWRF
Tremovirus          EWS----ICHPRISEETVYSMLAWKSD-----NASMKDLIETSIWFMFHHGPRKYVRFCT
Caecilivirus        ETG----SVHPTLDEKSIYGLLNWKRK-----NARFEDNIKDAAWFYYHHGRKKFDVFRR
Rajidapivirus       PKFP--LFVHPTIDPETYEQSVMWYKN-----TECMQEKLTSLTQLAYHAGPKNFKAWRD
Rohelivirus         ECG----VHRPLIDKDVCYQMLRYKTK-----NATLKDNVRTASWFMHHYGKEDYFQFMT
Fipivirus           QSC--PFLTHPVMDKDTVWGMLTWKKK-----GSTYAELMRCAVSFMWHHGKEEYERFVG
Harkavirus          EHFP--FAIHPVIALQTVDNMLCWKSD-----TAVFQDNVDAAFGFLYHHGQDVFDRYQD
Ampivirus           VIP---GYYLPGMDKKTIGNLLNWYRPRK--NPDQFRTNVQEALKFAAPHGKAYYNELLS
WenzhouPL-48        QG-----IVWPPMPMATCYREVNWCKRSMRYNSTVRKTQISEGRRFAAYHGKEQLAAFDH
WenzhouPL-47        DMLFLMRIHPGNFTSGGFESQAQIAYRNHVFSGPGMVNGKISYNTFKITSAANAFQNARI
```

```
DERSV-SD609           YNQKKAPSY----NHLSFGAAYEMMKTICLVY----------------------------
WDALV                 KLSKKAPSY----KHLTFGAARNMMKAVCLIY----------------------------
Aalivirus-A1          TLKSRAPQY----NHPTFLAAYNIMKPIVMVY----------------------------
PBD-A1                TMKSRAPNY----NHPTFLASY--------------------------------------
QKE55028              DLCKRAPSF----NHPTFDWAYNQMKFICQVY----------------------------
Grusopi-A1            ELRKISPGS----AWMPFDVALHRMKGICDIL----------------------------
Grusopi-A2            ------------------------------------------------------------
Grusopi-C             ELRKLSPGS----AWMPFKVALQRMKGVCDIL----------------------------
Grusopi-B1            AMREIAPSL----DYPPFTYMKMRILVITGLM----------------------------
Avihepatovirus-A      KLKARCPVL----DIQPWGVAKLRAYTACMMI----------------------------
Avisivirus-A1         SIAFRCPEL----VHPTYQCALQTIKPMVSLM----------------------------
Avisivirus-C1         KLAPRAPQL----VHPTYACAKATITPMVAIL----------------------------
Avisivirus-B1         FLANKAPHC----VHPTYDTAVLTVQPIVGFL----------------------------
Orivirus-A1           KLGIDIPWG----AAKAWAKALLSSVIEGLDPGPPDRIVSP-------------------
Orivirus-A2           ILGVDIPWG----AAHAWARALLSSVTEGLDPGPPDRVVSP-------------------
Crohivirus-A          EIKTKLDKF--KIYPPKFKHIISRLCLSIFD-----------------------------
Crohivirus-B          EVQPVLDGF--NINVPTFEDKLFDMTQIVFE-----------------------------
Parecho-B             RFNPILNQW--RVCMQDYEVALHRMLRYVF------------------------------
Parecho-C             VFQEVLKPW--KITMEDYDVVVRRMLFYVFE-----------------------------
Parecho-E             KFKDDLAKW--SIFMNDYDVVIRRMVGYVFE-----------------------------
Parecho-A             ILEPYLQEW--NITVDDYDVVITKLMPMVFD-----------------------------
Parecho-D             KVNKHLSKM--GMNMLPFNVVYNKMVQLVFE-----------------------------
Parecho-F             EMKDHLDGY--PLNFSNWNTALRRMTGYVFG-----------------------------
Potamipi-A            MCAHRLARM--KMYAPTYQEALRDATTKIFY-----------------------------
Potamipi-B            LCSPRLDQY--KIFTPRYDQRYKEVYQSIFM-----------------------------
WCP                   ACAPILDKY--RILIPTYAQRYVEVYDMLFN-----------------------------
Limnipi-B1            ACKRMLEGGSLQVNVPFWRAAWETYLGIVDWSVAGAMYPRDLWDPVLEFDDDDDNDVVFF
Limnipi-B2            ACKAVMDEG-QKVNIPFYDAAWETYLGIVDWPVHGTIVPRDLFDPVLEISDTDSEVEFLY
Limnipi-C             KCRERLKENREVVTIPSYDLAFETYLGIVDWDVVGEVTAEELFPVKLEISDEDD------
Limnipi-A             TMKRRLAKQNLQITVPGYDISWTMLNSVVMGDE---------------------------
Limnipi-D             LVNRAMRKSGSQVAMAPYHMS-VMCLAMTLQEVLDPGTLWTMQHFVLARTDNG-------
Pasivirus-A1          RVNSFLNGTG--FALMSYRRAAAVVGSMLS------------------------------
Pasivirus-A3          RVNPFLSGTG--FALMSYRRAAAVVDSMLS------------------------------
Pasivirus-A4          RVNGFLSGTG--FALMSYRRAAAVVDSMLS------------------------------
Pasivirus-A2          RVNSYLNGTG--FALMSYRRAAAVVGSMLS------------------------------
Shanbavirus-A1        ---ELFKTRK--IILPKFADAKFQLDKIVYQL----------------------------
Kunsagi-A             ALLPHATLPPWSQTLQSCRYLLGIEDALHPAGKT--------------------------
Kunsagi-B             ALLPAANLPPWDFAYRSAASVLGLEDMTHSPTFSFYCPLPPSVSTDDALIALTEMTRSLH
Kunsagi-C             QLMPTVTMMSWPVAHRTVLTMLGCY-----------------------------------
Aquamavirus-A1        AFFPYMILPPYSLMENTVRSVCGLNPVQSFLSKYNFGPDDRKKKGIGCWPW---------
Passerivirus          KVREQAARKGLLP--NILPYSYLHHRWLQLVVS---------------------------
Sicinivirus           TVRAQCLKNGFEP--TFIPYEVLQYRWLAMVMT---------------------------
Gallivirus            AVRDACKRNGYDPP-IFLEYSYLQMRWMQLVSG---------------------------
Pygoscepivirus        TVKAKCATVASYP--SFPPYSYLYYRWLQAIAS---------------------------
Kobuvirus             KVREQCLKSGFAP--NFLPYSYLQLRWLNLLAA---------------------------
Sakobuvirus           AVKAQCAANGVEV--RLLPYAYLQAAWLQTVMA---------------------------
Salivirus             KIKAKIRENGCDA--TFTPYSVLQRGWVSTCMTGPYPLTG--------------------
Livupivirus           QVLATAKLKGVLP--NFPSFEFLQYRWILSLD----------------------------
Oscivirus             TVRAKAHSRGVLP--RFYPFDYLQKRWELKLES---------------------------
Ludopivirus           KIVERAAELNLDI--KVLPYAFLQNRWVQKVSK---------------------------
Rafivirus             ACKTKCEDH------YFLPWSYLDLRWYMLCQTGEDPSGNFGGFVW--------------
Danipivirus           KCNAHLIRNGFPPF-SFTAFSYLFYVWSANNGITPLPQPFRTLPPHPTFFD---------
Symapivirus           RICATVREAHLEPP-VILPYAFLRAMWLNSLDYDLD------------------------
Dicipivirus           KL-TDICEERNISPPGLKPYSVHRNRWLR----VNGKGVASVPLK---------------
Rosavirus             SV-TRSCVDAGYPPPFFPPFALLRSQWLKKFEVVTFDRLQNVAQ----------------
Myrropivirus          AV-SQAASNRGE-TVYIEPFSLKNAKWVS---------KFDIGY----------------
Pemapivirus           AVRAQIQKNTHSCRFSFLPFSFLQSRWLA---------KFNTQ-----------------
Hemipivirus           RVMQKLTDNGFGSKYYLPAFKFLDAKWLS---------EFGL------------------
Tropivirus            TVSAKIKENQGTCKYYFPPYHFLRERWYR---------NFEA------------------
Megrivirus            TIQRRCRS--RGTEVYFRPFEYYMAMWYAHFM----------------------------
Poecivirus            RVVNVCEE--KGVDLYPPSFDFLMTIWYMKFR----------------------------
Ailurivirus           PFR---DAGMAVPTWWSMEEKWESNFMGWTT-----------------------------
```

```
Mischivirus       PFR---ELQMKVPSWFVLEHEWEHNFD---------------------------------
Cardiovirus       PFR---EVGVVVPSFESVEYRWRSLFW---------------------------------
Senecavirus       PFA---DYG-AVPSHEYLQARWRALFD---------------------------------
Mupivirus         PYR---DSGFLIPEWSTANRRWYANFE---------------------------------
Cosavirus         PFI---RDGFDVTPWKQLHLEWLNKLSA--------------------------------
Marsupivirus      PFK---QAGWTIPDYDLLNALWYRKNGLT-------------------------------
Aphthovirus       PF----QGLFEIPSYRSLYLRWVNAVCGDA------------------------------
Hunnivirus        PFE---SCGYFVPSFDDLELEFFSLFFG--------------------------------
Malagasivirus     SFN---GTGVVVPSFATVDEDFYYTHASGQ------------------------------
Tottorivirus      PFI---ETGFVVPSYDMLKDLWLWKNGVLPPMQ---------------------------
Teschovirus       PFE---EAGYEIPSHERLNEEWREMFI---------------------------------
Bopivirus         PVR---YYAYVP-TYDSLYDDWLNEFGFV-------------------------------
Erbovirus         PFV---EVGYCVRPFGDARRAWLRNFDL--------------------------------
Mosavirus         AVALYAP-GVSMPAYKYMKACWYAKMV---------------------------------
Torchivirus       EIENDSQYSKYIPHYHLLDFVWKQKNGIYI------------------------------
Felipivirus       KVR----TVPVGRALSLPTYRVLRAFWLDLF-----------------------------
Parabovirus       KIR----TVKIGRALFFPVFGVAQA-WLDMF-----------------------------
Sapelovirus       KIR----STPVGRALSLPSFEVLRYNWLDLF-----------------------------
Boosepivirus      KLR----SRPIGRLLTIPSYEVLEQKWLDKF-----------------------------
Anativirus        IVR----STPVGRALTLPAYQVMCQKWYDSF-----------------------------
Rabovirus         LIK----STNVGKACILPTYISLKRMWLDQF-----------------------------
Diresapivirus     KCR----TVPIGRALHYFDYNVLRHQWLEKF-----------------------------
Enterovirus       TIR----SVPVGKALAIPNYENLRRNWLELF-----------------------------
Crahelivirus      VLSDALRLNSRNERVPSYLFWKLFSVDYTGVRDVGWD-----------------------
Hepatovirus       FVQSCLEKEMIEYRLKSYDWWRMRFYDQCFICDLS-------------------------
Gruhelivirus      WMLSL-------------------------------------------------------
Tremovirus        WLRGVLCRVGIGLYIPTYKELEVRYDRLVKYRFIDDSF----------------------
Caecilivirus      TVEQALSEAGLGFAVPTYCAMHVRFKKAIQEQTLWFVG----------------------
Rajidapivirus     KIALTLVAN----NLTIPTYSELEATWLGKLLN---------------------------
Rohelivirus       EVVKRSKKLAEKMPVFEDMNWTFYHKLGMC------------------------------
Fipivirus         VMENHIGLIPVDQRSAAYNELCSYEELHDHWMNHKA------------------------
Harkavirus        LIRRVATDNGISIFLRPFSYYSRRWTGVMGFDLEGVHLEGAWNDVDLDVVIEDE------
Ampivirus         DLRNNGKMQVLYPGNELQALLQPFETVFYSTYLPFGLDAPETTPLFDFPFDN--------
WenzhouPL-48      AYRKNVHKEIGLLLDPVIPETYDSIEIDVRLKQLMQEMCPDN------------------
WenzhouPL-47      IIAQIPSEYSRLQIQAMKATELKQFPNREHFLHGTETIFNPQWVNKLPVITNHETDPSNT
```

```
DERSV-SD609          -----------------------------------------------
WDALV                -----------------------------------------------
Aalivirus-A1         -----------------------------------------------
PBD-A1               -----------------------------------------------
QKE55028             -----------------------------------------------
Grusopi-A1           -----------------------------------------------
Grusopi-A2           -----------------------------------------------
Grusopi-C            -----------------------------------------------
Grusopi-B1           -----------------------------------------------
Avihepatovirus-A     -----------------------------------------------
Avisivirus-A1        -----------------------------------------------
Avisivirus-C1        -----------------------------------------------
Avisivirus-B1        -----------------------------------------------
Orivirus-A1          -----------------------------------------------
Orivirus-A2          -----------------------------------------------
Crohivirus-A         -----------------------------------------------
Crohivirus-B         -----------------------------------------------
Parecho-B            -----------------------------------------------
Parecho-C            -----------------------------------------------
Parecho-E            -----------------------------------------------
Parecho-A            -----------------------------------------------
Parecho-D            -----------------------------------------------
Parecho-F            -----------------------------------------------
Potamipi-A           -----------------------------------------------
Potamipi-B           -----------------------------------------------
WCP                  -----------------------------------------------
Limnipi-B1           DDRLA------------------------------------------
Limnipi-B2           RETI-------------------------------------------
Limnipi-C            -----------------------------------------------
Limnipi-A            -----------------------------------------------
Limnipi-D            -----------------------------------------------
Pasivirus-A1         -----------------------------------------------
Pasivirus-A3         -----------------------------------------------
Pasivirus-A4         -----------------------------------------------
Pasivirus-A2         -----------------------------------------------
Shanbavirus-A1       -----------------------------------------------
Kunsagi-A            -----------------------------------------------
Kunsagi-B            FDDGIPQ----------------------------------------
Kunsagi-C            -----------------------------------------------
Aquamavirus-A1       -----------------------------------------------
Passerivirus         -----------------------------------------------
Sicinivirus          -----------------------------------------------
Gallivirus           -----------------------------------------------
Pygoscepivirus       -----------------------------------------------
Kobuvirus            -----------------------------------------------
Sakobuvirus          -----------------------------------------------
Salivirus            -----------------------------------------------
Livupivirus          -----------------------------------------------
Oscivirus            -----------------------------------------------
Ludopivirus          -----------------------------------------------
Rafivirus            -----------------------------------------------
Danipivirus          -----------------------------------------------
Symapivirus          -----------------------------------------------
Dicipivirus          -----------------------------------------------
Rosavirus            -----------------------------------------------
Myrropivirus         -----------------------------------------------
Pemapivirus          -----------------------------------------------
Hemipivirus          -----------------------------------------------
Tropivirus           -----------------------------------------------
Megrivirus           -----------------------------------------------
Poecivirus           -----------------------------------------------
Ailurivirus          -----------------------------------------------
```

```
Mischivirus         -----------------------------------------------
Cardiovirus         -----------------------------------------------
Senecavirus         -----------------------------------------------
Mupivirus           -----------------------------------------------
Cosavirus           -----------------------------------------------
Marsupivirus        -----------------------------------------------
Aphthovirus         -----------------------------------------------
Hunnivirus          -----------------------------------------------
Malagasivirus       -----------------------------------------------
Tottorivirus        -----------------------------------------------
Teschovirus         -----------------------------------------------
Bopivirus           -----------------------------------------------
Erbovirus           -----------------------------------------------
Mosavirus           -----------------------------------------------
Torchivirus         -----------------------------------------------
Felipivirus         -----------------------------------------------
Parabovirus         -----------------------------------------------
Sapelovirus         -----------------------------------------------
Boosepivirus        -----------------------------------------------
Anativirus          -----------------------------------------------
Rabovirus           -----------------------------------------------
Diresapivirus       -----------------------------------------------
Enterovirus         -----------------------------------------------
Crahelivirus        -----------------------------------------------
Hepatovirus         -----------------------------------------------
Gruhelivirus        -----------------------------------------------
Tremovirus          -----------------------------------------------
Caecilivirus        -----------------------------------------------
Rajidapivirus       -----------------------------------------------
Rohelivirus         -----------------------------------------------
Fipivirus           -----------------------------------------------
Harkavirus          -----------------------------------------------
Ampivirus           -----------------------------------------------
WenzhouPL-48        -----------------------------------------------
WenzhouPL-47        NGWLVAKILENSLVSTSTAPRLTYWVCANAVVYSMPRTPKALPAVTT
```

Mosavirus polyprotein alignment

```
Mosa-A1         ------------------------------------------------------------
Mosa-A2         MVTTIGIVIDDTVELSDFTTIPESSNILTTLATLEKDLTMDSAELIKILESRCICVEEVC
Mosa-MgMV1      MVTTLGTTFTEFKERVHRIKQQILKQVLKQLILYTPHPISQLQHTMSGINFSTYCFRKFA
Mosa-B1         -MASFNFNTTFFYPKPFPTDPCDILHARLNAQRFR-REIAEVERLCEMYTVPEIRYRGFP


Mosa-A1         ------------------------------------------------------------
Mosa-A2         ECSTPVLNNRKYGVIVIKGRENETDEVVVNSLAAKYLTLYEDDDAEEYCSNVMWTCPYEK
Mosa-MgMV1      ASTQNVTKN-------------------------YRPFFNLHNVDHGPANEMYG-----
Mosa-B1         NPGSTCWFNCFRQAMILSAHPGLGELFANLEYTEEESRELANHFVSSWQSDIWSYGGAVA


Mosa-A1         ------------------------------------------------------------
Mosa-A2         QAFNEHMERFKHPEYRYAG----------FENFNGNCWFNSARQLFRLVGEPDLVMLFDQ
Mosa-MgMV1      -------LLMQNPLNRYEG----------LPNLYGNCWFNTVRQFCRLVGFNEFTRWVEC
Mosa-B1         RTTSFRGGLPQAPFHVYLRDNEWEMQEYCDPHLNPHFLYPWIQILYKLDGTPQEWPIVDY


Mosa-A1         ------------------------------------------------------------
Mosa-A2         LPHNPDVIESFYKATRDILGWRVGTDIGGHPESLVLLLSRSWANDSEGAIYKHRYVRADA
Mosa-MgMV1      S------------------DWR--------------------------------------
Mosa-B1         PSILILRKQDDTTCHVVLAALEN-------------------------------------


Mosa-A1         ------------------------------------------MKVFKTKNWRDHGEPMVG
Mosa-A2         DLGYWWDAYELPVMVLYDDTTYEVPHVRVVAKENDYWIVFDDDRTYCTKNWRHFGKPVVG
Mosa-MgMV1      ---------------------------EIYNDWNQHFLTFPHERFGTNPNGGFFQGQEME
Mosa-B1         --------------IMDHPYYVVYDDDDVYGMEPEELLWDHFIKNLSDPLSRHVEMYYTG
                                                           :

Mosa-A1         SAGINMLQLVPERRNWKGKYGSLIPYTECASTEWTPTMVNEIEEQPECFMQRAIEEYLS-
Mosa-A2         SAGINMLQMVP--AQWR--TPKSVKQHQIVRPRWTKTKVAEIMEQPMCYMQALLIEHFEK
Mosa-MgMV1      AVLISLGLDIDNYLDYEGNRPNTVKRYQPYMGPKTLLPITFIQIKWANGDAHIVLAVQDG
Mosa-B1         SNILQITQPVFHSTRTFNPHYHGLKTMWDFQESYVELATTHKCPLQMSILHQLIKINRSF
                :  :.:   :             :          .                  :    .

Mosa-A1         ------------------------TQNMSYMKAVELRDAIAQLPVYANYADFLKITWLSP
Mosa-A2         DPEFNRCVYQLAQYSIPACIWRTDRRNFTMPRATDLLHKVVRQQLDQKCFAETSVYWYTV
Mosa-MgMV1      ----------------------IMPNRWVVYDDGDVYCVRDPLTLPGAYPILGWSGSNML
Mosa-B1         KR----------------------GHYSNMYSADKAYRQWWMQPCIAESRGTGNCDATTI
                                         .        .

Mosa-A1         K------DEDLWEEQMEIDHESR-------DVEPMEVVDCEFAVTQGGGESKPQQGNVNG
Mosa-A2         PGFKKASFAGNYDSRLPLQHLYRRHHEWFMRHHKLPVVETESALTQGGGQSKPQQGNVNG
Mosa-MgMV1      LRYPVRDLKKKKRKQSAQVGDLCKGNKIATGVAMAEEAEQYHALVKGAGYSKPNNGGSNN
Mosa-B1         S------QCGDVETNPGPVFTLYIMDGVELADEVLEDCFELPIEAQGAGMSTPKQGDVST
                              .                             .:*.* *.*::*. .

Mosa-A1         SQNQGINIYNYYNQQYQNSVDMSAPMLNAGGSNPMGTSSSHNTTSNSLSGLLTAGFNMAS
Mosa-A2         SQNQGINVYNYYNQQYQNSVDMSNAPLSVGGSG-GGNQAASNHTNNSHMNFVTTGFNMAS
Mosa-MgMV1      SGNNGTQIFNYYNQHYQNSIDLS------------ANAAGNQKPEGQLNQLIEAGSQLAG
Mosa-B1         SNNQGTQNFFYYNNHYQNSVDMSNSPVNTND----SSTGETSSSGSGVSSLLNGFTSLAS
                * *:* : : ***::****:*:*            .. .  . . .    ::    .:*.

Mosa-A1         NLVPLMLMDPDTEESTELPDRITEDTAGNTNITTQSSVGTLVAYHQLRSKHPVTSCADKP
Mosa-A2         NLVPLMLMDPDTEDSTELPDRITEDTEGNIKVTTQSSVGTLVAYHQLRSKHQITSCAEAP
Mosa-MgMV1      -----MVSRP---IQKMLPALGFRSVDGNTATMDPCEMGVLRAYGGDR-AAPPTSCADKA
Mosa-B1         TVGPMMLLDPNTEEDNMQPDRIQQESRGNTQTTTQSSVGTLVAYGRTAVKTPVSSCADAP
                     *:  *    ..  *    ..  **      ..:*.* **         :***: .

Mosa-A1         TIGGPAMERNFVQYIGTWETAQVEYQCRYLPLPHGLEEMGVFETTAIRHYTMKCGWKIQV
Mosa-A2         TTGEPAHERNLVQYIGTWEQTQVEYVYLALPFPSGLEETGVFGVTANRHYTLKCGWKIQA
Mosa-MgMV1      TTPGEATDRNFVQVMGTWETTTAQKMCLVLPLPTGLQDTGVFGEMTRRHYCMKCGWKVQV
Mosa-B1         TMGTPSVQRNYTMVVAQWTSTQTAYQYVAGCLPYCLPEAGLFRQNMDRHYTTRCGWKIQG
                *    : :** .  :. *  : .        :*  * : *:*     ***  :****:*
```

```
Mosa-A1         QLNTSHFHAGCLGVFAVPEAQWVGKFTMSTEWE--------------GLPTDMRPESFFL
Mosa-A2         KLNTSHFHAGCIGVWAGPEPSFLDSFQRTTTWQ--------------TFSTDVRPESLFL
Mosa-MgMV1      QVNTSQFHAGSLAVFCIPEFQAYNSLAAQKNWS--------------IAPE--NPNQWTL
Mosa-B1         QVNTSHFHAGCLGVFAIPEFKFPFNESGFSGWTNKMYNNGVQQIGDVETTLSGAPESWTI
                ::***:****.:.*:. ** .   .    . *                 .    *:.  :

Mosa-A1         YPHQLINCRTNTSVDLILPYLNFVPTSAYGYHCPWTLVFMVLTPLQIPTGASPVIDISCT
Mosa-A2         FPHQLINCRTNTSVDMILPYMNFVPTGAYGYHCQWALLFVVLTPLQIPTGASPTVDISAT
Mosa-MgMV1      YPHQFINCRENTSVNFELPYLNLTPATSPMMHAPWCVVVMVCTPLQIPTGAATTVDILAT
Mosa-B1         FPHQFLNCRTNTTVDLLLPYLNFVPTSANGYHAPWTIFIMVITPLQVPTGAATVVDISMT
                :***::*** **:*:: ***:*:.*: :   *. * :..:* ****:****:..:**  *

Mosa-A1         VVPQWVQYNGLRQPQTITQGFAGHIRENQAMYASTIPDETTPVYGLGFNPNTDFVPGEVH
Mosa-A2         ITPKDVVYNGLRQPQ-VAQGMPVHIRENQSMFATTIPDETTPVYGLGFNPTSDFVPGEVH
Mosa-MgMV1      VAPQRAQFHGLHDAV-ATQSVPVHVREDAGSFLNTHPDVSTPAYGKGFNPSVEYLPGEVT
Mosa-B1         VAPTAVQYNGLRHSSSLAQSAPVQIRENNGQFTTTIPDRATPAYPLASVPTVIIMVGRFH
                :.*  . ::**:..   :*. . ::**: . : .* ** :**.*  .  *.   : *..

Mosa-A1         NFLEWTRTPCLMSNVVDDTIRGYFTASNTRSDEPLLQMDVTLASDHMRYTALGQMSFRYA
Mosa-A2         NYLEWARTPCLISMVEDGSIRGYFTANNTRSDTPLLQMDVTIASDHVRYTPLGQLGYRYT
Mosa-MgMV1      NFLSWATVPCLASLGSDTKFS--FEASNSLQDSPLMQVDVVLTSNAMRRTAVAQLGFRYA
Mosa-B1         NYLEIAKIPCFITNTVGSVAAPYFTATNARPDEALLVMDVTLSSSHFLCTALGCMRLIYA
                *:*. :  **: :   .      * *.*:  * .*: :**.::*. .  *.:. :   *:

Mosa-A1         QYRGSINVMLTFTGAAMVKGKFLLAYTPPGAERPSTIEEAMQATYAIWDLGLQSSYDFTI
Mosa-A2         QYRGSINVMLCFTGAAMVKGKFLLAYTPPGADRPNTIAEAMQATYAIWDLGLQSSYDFTI
Mosa-MgMV1      QYRGSLNVSFMFTGASAVKGKFLIAYTPPGGNAPNTKAEAMQSTYVLWDVGATSSYTFNI
Mosa-B1         QYRGSLNIDFMFTGAAMVRGKFLIAYTPPGATQPATMEEAMQATFSIWDLGLQSSFQFVV
                *****:*: : ****: *:****:******.  * *  ****:*: :**:*  **: * :

Mosa-A1         PFISVSDFRLTASGIPSTISVDGWFTVFQYTALTYPANTPQRSDVLVFISSGQDMCFRNI
Mosa-A2         PFISVSDFRLCFSGTTSTLSVDGWFTVFQYTALTYPANTPQRSDVLVFVSAGQDFCYRNI
Mosa-MgMV1      PFISISDFRLTASSTTDVLSVDGWLSVFQYTSLTYPANTPERSEVVVLFSAGKDFCYRNL
Mosa-B1         PFISVSDFRYTNSAGTSTLSVDGWLTVWQYTALTYPANTPQRSDIVVMVSAGADFCFRNP
                ****:****   *. ...:*****::*:***:********:**:::*:.*:* *:*:**

Mosa-A1         IDTREKNNDMQGLDNAEEGASENPTTIEDFEGKELGSLTTHTGLGFVFDRSFGISHIKLD
Mosa-A2         TEVREKT---QGLDNAEEGASENPTAEEDFAAKPLSGEIQHTGLGFVFDRSWGLTQLTMP
Mosa-MgMV1      ISARYQS----GQVDIETGQKPE-VDATQLEDRALAAPSAHTEVSFVMDRSWSLGRRTIN
Mosa-B1         CDLAAQG-----LDVAEKGEQEDLAAPDQIAGKELVSVHQHTDIGFILDRSYFMETVALP
                 .   :          * * . : .   ::  : * .   ** :.*::***: :    :

Mosa-A1         NTASYTMDLSMEVLMT---------KELAWWLSSVTYFKADLEITVLPIMMKATP-----
Mosa-A2         GKATATVPLTITELLNGGVN-----RELHWWLSALTYFKCDLEVTVMPFGNAPSNTTG--
Mosa-MgMV1      ADTPVSVQLALKDLEWN--------KDFKQWVQMFTYIKCDIELTLLKPEG---------
Mosa-B1         NLTPKYHPLTLNGADHSAFSSTQGLYSMGPWLSCITYLKADLEVTLIPRADPTHTGQTGT
                  :.    *::               .:  *:. .**:*.*:*:*::

Mosa-A1         ----FKLAAKFYPVGSTVNIVAN-PTSTEGFLTITGPCPMILGMDRQPLTFAVPYTSPLS
Mosa-A2         RPVCPRVLVKFYPVGATVPTTDDNPIVSGGMIQNTGPVPLMVSGSYEPVSFVVPYTSPLS
Mosa-MgMV1      -----FCWMRWDPVGASVQVDTS-----DKFHQKLGAVPVTCGSGN--LKLSVPYTSPLS
Mosa-B1         ENRNVRLWVKYYPAGAPVPTTLS----MDSFIRRTAGSPLMVSDYGKPISFEVPYNSPTS
                         :: *.*:.*    .       :    .  *:   .     :.: ***.** *

Mosa-A1         VMPTKYLGFADYARTELANWAPGASFGALRLGLLNENQ-EDKVAVLVFVRFKNFRAYCPR
Mosa-A2         VIPTTYFGYADFERTALSNVAPGASFGTLRIATYSP----DPMYIQVYIRLKNFKGYVPR
Mosa-MgMV1      ALPVFYLGKTKFGDNTLG-EMPATSFGTVTIMQETG----KAMRTYLQIRLKNFRGFCPR
Mosa-B1         ALALAYNGYSDFQKTQLTSHAPGNTFGAIYFGISHSLPNEAIVDVDIYYRFHNVEAYAPR
                .:.  * * :.:  . *    *. :**:: :           :   :  *::*...: **

Mosa-A1         P----FKNAGQTPTNSRAKVVTTDDFVVFR----SAHQDVTLGGDVETNPGPTILTVPYE
Mosa-A2         P----LKNAGATPTNSRAKLMVDEDYVIQR----SAHRSVLLDGDVESNPGPVVLSVPFE
Mosa-MgMV1      P----VRNSGPSPGEERSRWDVDTNAVLVRGRARSMQKELGLTGGVESNPGPVASKAALK
Mosa-B1         PFYQFLTYTPSSTTSLRAHTLMNDDDVYVRRSANRTAADISQDGDVETNPGPVSPKHSCP
                *    .  :  :. . *::     : *  *        .:   *.**:****.  . .
```

```
Mosa-A1        T-ARTQGLEKLYNFVSGGIF-------QHVLDTTTEAKDTVNGISRATNAFGDIIENFKK
Mosa-A2        T-ARAQGLEGLYNWLSGGVFGSFADAAKHASNTVGQAEEAAAGVARTTNAFADIVENFRK
Mosa-MgMV1     AEAELQGLGKFYNIVSGGLFG-------EMMDTTKEVGGAAKKMKETLGTIEEVFAGLKN
Mosa-B1        Q---AQGFARVIDHMTAGLIS-------RSHNTLDGVNEAVNSVNSIKQLVDQALS----
                    **:  . : ::.*::        .  :*    .  :.  :      . : .

Mosa-A1        MSDMAVEAMQSVKQWISIVRKMFRMFCYLIIAYRTKDPVVIGLLGVDMAFGDPFDIIEIV
Mosa-A2        LSDYAVDAVKAVKAWMTTVRKVLRMFCYLVIAYRTQDPVVIGLLGVDIAFGDPFDIIEII
Mosa-MgMV1     X--------SSAFVWAKIAKKLFKMVSHAVIAYRCQDPVVLGLLGCDIVFDSAFDIYDIF
Mosa-B1        ----------GFKLWSKVLRKMVKYFIQGLIVYRTRDPMVAALLGISDMMEDPFDLYQIF
                         .   * .  :*:.: .   :*.** :**:* .*** .  : ..**: :*.

Mosa-A1        KNKLQKIFSTPAPPIATTQG-VSDLNALFSLLKNGEWAVKLILTIKDWLNRWIAQEMDTP
Mosa-A2        KAKLKKFFTTPAPPLAQTQG-VSDINAVFNMLKNGEWALKMILTVKDWLQRWINQEMEAP
Mosa-MgMV1     RKYLSPLFQTPPPPVVEEQGTLQEVNQIFTLLKNTDWAIKLILHLKDWLQRWINNELETP
Mosa-B1        KDTLSHVFVTQPPPLA--QG-VNDVNAIFNLMKNVDWLVQLVKRVFAWLNQWVEQDKVTP
               :  *. .* * .**:.  ** :.::* :*.::** :* ::::  :  **::*: ::  :*

Mosa-A1        EKQLQKLMPELLEALEIMEKKDAESQKKKPTLLPKIQHASKLARMCNRMSIANYCDSVLK
Mosa-A2        EKKLTKMMPELLDAIETLEKKDANAQKAAPKMIPIIQEAARLARMCNRTSIANYCDSVLK
Mosa-MgMV1     QSKLAKILPEFNQAQKVLSKKDKTAQVEKGKWLPVVVETAELARQVGKIHIANAAEELLK
Mosa-B1        QRQLQTALIELPDIVASLEKKQAQDNPDLPTNISKAREIKRLATQLCERRIASYMDELLK
               : :* . : *: :    :.**:   :    . :.   .  .**    .  **.  :.:**

Mosa-A1        PFAMSGKQRTEPVVIVLKGKPGQGKTVAATLLAQMISKSLVGTQSVYSLPPDSKHMDGYN
Mosa-A2        PYAMSGKQRTEPVVIVLKGKPGQGKTVAATLLAQMISKSLVGTQSVYSLPPDSKHMDGYN
Mosa-MgMV1     AYHMASGHRTEPIVVVLRGDPGQGKTVAATLLAQMLSKKLVGCQSVYSLPPDAKYFDGYN
Mosa-B1        AYCHDSGARTEPIVILLKGAPGQGKTVAAEILAQMISKYMVGYQSVYSMPPDCDHMDGYN
               .:   .  ****:*::*:* ********* :****:** :** *****:***..::****

Mosa-A1        GQTVVIMDDLGQNPDGLDFATFCQMVSTTQFVTPQASLADKGTCFTSPVIIATTNLGDFR
Mosa-A2        GQTVVIMDDLGQNPEGMDFATFCQMVSTTQFVTPQASLTDKGTCFTSPVIIATTNLGDFR
Mosa-MgMV1     GQTVVIMDDLGQNPDGQDFSTFCQMVSTTQFVTPQASISDKGTLFTSPIIIATTNSRNIR
Mosa-B1        GQYVVVMDDLGQNPDGKDFATFCQMVSTTQFVTKQAAIADKGTTFSSRVIIATTNLGQIR
               ** **:********:* **:************ **:::**** *:* :******  ::*

Mosa-A1        PVTIADPGAVKRRIFLELDVSAGVKTPSGCLDLAAAMQETSDQGLINTKPDCLTTC-PKL
Mosa-A2        PVTIADPGAVKRRIFLELDVSAGLKTPAGCLDLGMAMENCQDLGRP--KPDCLQST-PKI
Mosa-MgMV1     PVTIASPEAVERRFFLHLDTEAVAKDPMGRLDMTVALERQDCCAKEMEAFDGVLSFKPNI
Mosa-B1        PVTVADPGAVDRRITFCYTVEAAAKDANGRLDMECAVK-TDPQFVTQEIYNASPPCLQKV
               ***:*.* **.**: :   ..*  * . * **:  *::  .         :   .   ::

Mosa-A1        MHSAVLKLTETR-INVTFSLIDVFNRVMRQLKQKEEMTDLLANVVMQG----------VV
Mosa-A2        LHSGVLKLHETR-INVTFSLLDVYEKVMRQLKQKEQMTDLLAGVVCQG----------LV
Mosa-MgMV1     LSANFLRFRSARDQKVVFSLETVYHMVLDRLASKEGMSDKLASIVLQG----------IL
Mosa-B1        TNLFSSRCLRFRARDYSDSLLGVFWNAVYKLQEKSALTVELSRIVAQGNTCYRPMTIPEA
                     :    *  .   **  *:  .: :* .*. ::  *: :* **

Mosa-A1        TTEVETVKGSIIEKVTHDGEKVTIYDVQKEKTLLDNTLQNFLVAVALVPTFITILSILVN
Mosa-A2        ITEESITG--TVTQAEDQGRLITIYDIHKKKGELDNSFQEFVTMIALVPVFFSILTMAVN
Mosa-MgMV1     PEHTVRMTERREFTEVIDGVETKIYREMEEVRDLENHMTTILTVAACVGQICTFASILFT
Mosa-B1        REVLQDFRARMESQSVRARLEAAPVNPYRRWFDLMDKLYKFLQVSTIILSVVSVVMAIVK
                                           .. * : :  ::  : :  . :.    ..

Mosa-A1        VISYFFGTQEE---EKQEEEAEA---EGPYCGTCRQKKP-VLKKAVTEGPYTGIVKKA--
Mosa-A2        VITYLFGGKNEPCEMETQTEPEE---EGPYCGACKRKAP-VLKKTVAEGPYSGMPRAT--
Mosa-MgMV1     FVSWCLKSPKTEETPKEEEAPVE---EGPYTGTPPKKQEKKLEKQVLEGPYSGTGKKADI
Mosa-B1        FVQFMWTLINPKQKDDPPTPVEDNVCEGPYSGTPTPTPR-KLKKQQAEGPYSGAPAPA--
               .: :     :     .          **** *:   .    *:*   ****:*    :

Mosa-A1        PKKLKKVVTQTTEEVKELKARFVKVTP-EEIAETQGP-KSDAEASLLERNTTPVTYLKDG
Mosa-A2        PKKLKKVVVQAREE-QELKPRFVTITP-EEIAETQGP-RSDAEASLLERNTCPVIYMRNG
Mosa-MgMV1     KKNLQKVVEQADEEYKKLEARFVKINKNEEPAVVQSQEVMDWEMSLIQRNLVPVVYYRGE
Mosa-B1        PRKLKKAAVQGRPQ-----IRLVRITEDEIAEAQGIAARPDFEYSVMVRNTKPIHFLIDN
                ::*:*.. *   :      *:* :.  *          * * *:: **  *: :  .
```

```
Mosa-A1       KEVSSLTAIKLCSGKALINKHQFDKDIWDTMVIDGIKQTRDQCQIVGFTTKRG-IAYDLY
Mosa-A2       KEVSSLTALKICNGKVLINKHQFDKDIWDILEIEGIQQTRDQCSISGFVTKKG-VPYDLY
Mosa-MgMV1    TQVASLTALKLCDGKVLINKHQFDYDTWDHISVGSWVGTRDEAKVVGFLNQATSLDYDVY
Mosa-B1       NEVS-LTALRVHGDWVVTNRHQLFAKGVTKVRIGSVSHPVSDLEIVEFETKES-KPLDAI
              .:*: ***::: .. .: *:**:  .     : : .   . .: .:  * .:      *

Mosa-A1       VVDVPKM-QCRNIVSMFTDEQPKQETLVGCCNSTNYQKMMWHGEVLRCVENLHTNDGILP
Mosa-A2       VIDVPKM-QCRDIRNMFVDEEPRCETLVGICNSTAFRKMMWHGEVLRAVEHLTTDCGILP
Mosa-MgMV1    VLEVPGM-QCRDIRKHFTNAPPECEEITGICHSKRYPGLIWAGRVTRVLERLNTTTDVLP
Mosa-B1       LLKFPGLPQCRSLLKHFTDKCPTG-PAIGLCNSENYPNMMWQTRIVRTYEGIPTNMGFFP
              ::..* : ***.: . *.:  *      * *:*  :  ::*  .: *  * : *  ..:*

Mosa-A1       KTIAYKTPTRAGFCGAPLTARRGGQLKIIAVHSAGNGVNGFGTLISKKMIE--EAVTQGI
Mosa-A2       KTIAYKTPTRAGFCGAPLTARRNGQLKIIAIHSAGNGVNGYGTLISRKMIE--EATTQGI
Mosa-MgMV1    CTLSYSVPTKAGFCGAPVIATRKGQKVILGIHSAGNGTSGFGTIVTHRMIM--AAT-QGI
Mosa-B1       KIMCYSTPTRVGFCGSPIVAEVDGAKKILGIHCAGNGVNGFGTLLTRKMLESVCAEAQGI
                :.*..**:.****:*: *   *   *:.:*.****..*:**:::::*:    *  ***

Mosa-A1       IYDERPGPFVAVNRKTQIKRSPLFPLFQPEAGPAVLSQYDRRLADGIVLDEALFEKHVSD
Mosa-A2       IYDERPGPMVAVNRKTQIKKSPLFDVFKPEAGTAVLSQYDRRLADGVVLDEALFEKHVSD
Mosa-MgMV1    KIDQYLGPRVHVNRKSQIKKSPLWEVFKPKAGPAVLSQYDRRLFPEVVLEKTLFEKHTGN
Mosa-B1       LYGHSKGEAVPMNQKTQIKKSPLFPIWTPDYGTAPLRASDSRILPGYNLDETIFSKHNSD
                ..  *  * :*:*:***:***: :: *. *.* *   * *:     *::::*.** .:

Mosa-A1       MDVLPKEFEIACDMYAEELFARIGRENGLVSMYRAMNGDGISDAMDMTRAVGYPYCLDSK
Mosa-A2       MEKLPKEFEIACDMYANELFSKIGTNNGVVSMYRAMNGDGISEAMDMSKAVGYPYCLESI
Mosa-MgMV1    MKSLPKEFEIATEMYAAELFGKIGTDNGVVSVSKALNGDGVTDAMDMDTSVGYPYVLANA
Mosa-B1       LNTLPPEFEWATKMYAEELFDVIGRDNGPVSIFRAINGDGVTDAMDMNKASGYPYCLQGI
              :. ** *** * .*** ***  ** :** **: :*:****:::****  : **** * .

Mosa-A1       KRLDMVEIVETENG-KLYLPTEQLVEETEKYFTGEEKPKFVTFLKDEVRSNEKIKQGKTR
Mosa-A2       HRVDLIETKETPEG-KVYFPTKRLEEETRKFFYGEEKPKFVTFLKDEVRSNEKIKQGKTR
Mosa-MgMV1    KRRDFVDVVETPQG-NKLVPKEELVEEVKKYFTGQTKPIFVTFLKDEVRSEAKIQQGKTR
Mosa-B1       KRKDLFEVKETKQGGQVYLPTERLLQDIDEFCDTG-DTTFVTFLKDEIRPNKKIQVGGTR
              :* *:.:  ** :* :  .*.:.* ::  ::     .. ********:*.: **: * **

Mosa-A1       IVDASPFPYAIAGRMVMQNFMSNMMRCNGTEVGSAVGCDPDTEWTRYFFELCDKYVFDLD
Mosa-A2       IVDASPFPYAIAGRMVMQNFMSNMMAHNGIETGSAVGCDPDVAWTRYFFELTDRYVFDLD
Mosa-MgMV1    IVDASPFPYAIAGRMVLMKFMSNMMRNNGTRTGSAVGCDPDVDWTRYAHELCDEYVFDID
Mosa-B1       TVDGSSVPFAVFGRATLMKLMSKMMENNGTGIGSAVGCNPDDDWTRFAFELQDRYVFDLD
               **.*..*:*: ** .: ::**:**  **    ******:**  ***: .** *.****:*

Mosa-A1       YKAFDSTHPTAMFNLLAERFFTERNGFDQQAVRIFLNGLSDSDHVYEGKHFRIRGGLPSG
Mosa-A2       YKAFDSTHPTAIFNLLADRFFLPKYGFDQEAVRIFLNGLSDSDHVYESKHFRIRGGLPSG
Mosa-MgMV1    YKAFDSTHTTAIFDLLASHFFLDKYGFDNELVRVFLRGLSTSWHVYEDQYFILEGGLPSG
Mosa-B1       YSGYDATHTSAMFDLLDKYFFTERNGFDTACTHLLLQTLKDSQHVYGDTYFRMRGGLPSG
              *..:*:**.:*:*:** . **  : ***   .:::*. *. * ***  . :* :.******

Mosa-A1       CPCTSILNTVINNIIVRAAIIGAYQIDTVDFQKFRMLAYGDDVVYATPQPIKPQDLADWL
Mosa-A2       CPCTSILNTVINNIIVRAAMLGAYQVDTIDFQNFKMLAYGDDVVYSTPQPIKPKDLADWL
Mosa-MgMV1    CPATSILNSVMNNIIVRAAILGVYKADSVQWDQFKMLAYGDDLVYSSPQPILPGELADWL
Mosa-B1       CPATSILNTIINNITLRAALLGVYDANTVDWDSFRMLAYGDDVVYASPQQILPGDLAEWL
              **.*****:::*** :***::*.*. ::::::.*:*******:**::** * * :**:**

Mosa-A1       HANTNYKVTPASKAGTFPEESTIWDVTFLKRSFKPDEDHGHLIRPQMAVGNLRQMLSFMR
Mosa-A2       HSNTNYKVTPASKAGEFPDESTIWDVTFLKRAFIPDENHGQLIKPVMSMSNLKQMLSFMR
Mosa-MgMV1    HKNTLYKLTPASKAGSFPVESKIWDVTFLKRAFVPDEKFPLLIKPVMTLENLETLLSFTR
Mosa-B1       NKNTCYKLTPANKTSSFPTESRLSDVTFLKRSFTPDDDYPYLFHPVMPLENFKTMLSFCR
              : ** **:***.*:. ** ** : *******:* **:..  *::* *.: *:. :*** *

Mosa-A1       PGTFPDKVRSVAGLAVHCGEEVYNQLADAVALYAPGVSMPAYKYMKACWYAKMV--
Mosa-A2       PGTFPDKVRSVAGLAVHCSEEDYNELADAIESTVPGVAMPAYSYMKACWYAKMV--
Mosa-MgMV1    PGTFPDKVRSIAGLAFHLPSAQYEKLAEALLSVDGCAYFPAQSYLKHVWYEKNS--
Mosa-B1       PGTFQEKVQSVALLLQHRGEEDYDAIMDQVKAVVPTVRVPSYTRMRAEWLSLFGCC
              **** :**:*:* *  *  .  *: : : :      . .*: . ::  *
```

Aalivirus Polyprotein Alignment

```
AalV-B1         --METLVQPIKKIAGDVEQAVT--TAEEASTMISDSISAVTHQQQVGAAIETG---QPTE
QKE55028        --METLSKTATQISQEVSTVAD--IATELPKLVEGTLNAAVEDPKLAASTTTQSAVTAET
AalV-A1         MQMESIVSSATTLVKEVVPDVQKELSDIVSQTIQTGQEGIVQSDASQSSAILNQEPNNEQ
                  **:: .. . :  :*   .    :   .  :.   .. ...    ::

AalV-B1         VTDLHTEGTTDDMLSCAMSVDYYKDNFSKLVHISTVSWPSTAVDGTKLDYRWLPNAFFQT
QKE55028        SVTPHTTGSADDMLSCSMQVDFFKENISKMVFVHSGSWSSSDTRFKVLSTILVPESLNND
AalV-A1         TMLLSTDHSTDDFLSCSMSVDTSEQNPEKMIMLKTIDWGTTVANNKILARLNVPEAFLVD
                     *   ::**:***:*.**   ::* .*:: : :  .* ::  .   .  *     :*:::

AalV-B1         G-MPSAAASKVFCYTRCGYHFRLTVNASPGVLGSLIMVYIPGGFRTELEGNNLNRNFNSV
QKE55028        DTLPSSQTYKFFRYLRSGWHFKLFINVPPGCSGALAISFVPGGYRNLVSGTTFQRDASSI
AalV-A1         NTCPAWGQSKFFRFIRCGYQFRVTLTAPMGTAGLLVLTYMPPGYGTYSNDTDFTIDANSL
                .  *:    *.* : *.*::*:: :... *  * * : ::* *: .   ... :  : .*:

AalV-B1         MALPHTILDVRCSNQADLVVPYAHYKNYYNYVEST-----DIGAALGVFVFAQMKVAASF
QKE55028        LALPYAFADIRCSNEVDLVIPYTSFKNYVNYTTST-----DDSGMFIVWVMSPVMIG-TM
AalV-A1         LALPHAMLDIRCANEAVLTVPYMNYRNYIDYTTTTGTEYTRDGGQVIVWVLSQLRVG-SS
                :***::: *:**:*:. *.:**  ::** :*. :*       .. .  *:*:: : :. :

AalV-B1         TGEIDFSLYGQMIDPDYQCPRPIAQGR--PKRRKVKPPPAPPVKHHTMVDGAPGCVNMSN
QKE55028        SGAITYSVYGEMVEPDYQCPRPFNQGP--AKRKTVFPPKNPPVKTHVQIDGATGCVNLSN
AalV-A1         AGIVGVSLYGQMLDMDLQCPRLWDNGKKHARRKAVVPPPKPPVNHHVVVNAGPGCANLAN
                :* :   *:**:*:: * ****    :*    .:*: * **   ***: *. ::...**.*::*

AalV-B1         VQSTGVSESLALVGESTAVDYSSAGCASNIHDLLEIMRKWTIIWSGPWEATVTRGQVITQ
QKE55028        VCSTGAAESIALAGESTVFDYATAGCASTINDLLEITRRWTVWFDTEWAVGKAAGERIEA
AalV-A1         GLSMSHGESAALVGESTSVDHTSAGSNACYTDLTQVLRRWTVIGRLSWQASSGARVVINT
                  *  . .** **.****  .*:::**. :    ** :: *:**:      *  .        *

AalV-B1         FDFKPYLYGNFGLVMSSFQFFRGSIELQVLTYASPLATARYQITWFPDS--YDTVTLEQQ
QKE55028        ATFQPSRMGLIGAIFNSFMYFKGSFEIKVLVFGSSFSSGRYQISWVPESGNSANFSLQQC
AalV-A1         LNVILN-QGVMNIVCKNYDWFRGSIEVKAIVVGSEMTAGRLQMSYFPKMT-NGQPTLEQC
                  .     * :. : ..: :*:**:*::.:. .* :::.* *:::.*.        :*:*

AalV-B1         RNGVYLTADVGCESGTLVLPYTYSTWRHETNTRYGRITMSCVNRLAYNTTAPTTIGVKIL
QKE55028        RNSIYATNDIGCEATTLVLPFVHNSWRRETNLDYGRILLHCVNKLTHNQTSPSSVRMMVL
AalV-A1         RNSLYVVSDVGCGPLKLTIPFVSNNWRMQTSTTIGRLVFHVVNKLTHNSTAPSQVDIVVM
                **.:*  .  *:**  . .*.:*:. ..** :*.    **: :   **:*::* *:*: : : ::

AalV-B1         MRAGPDLQLYCPRYPVVTIQ--GNSEEVDEPTLFINYEIEQVPIQSRSHSEVNSFFGRVQ
QKE55028        VRTGPDMQLFCPKMPKLNVSGVGQQLETDTQTCFINYEIEKIKIQNESHSLVNNYFGRLT
AalV-A1         ARIGTDFQLTSPTRSTLLLS-QGAGDDDEQPVYFVNYEIEKIPIQSESHTLVCNVFSRSY
                 * *.*:** .*    . : :.   *    : :   . *:*****:: **..**: *  . *.*

AalV-B1         HHGVYTLTAGQILEQTIDVSAIRAWRVLETVAYWTGELIASVVNHTDSPLYFTHKYTTFI
QKE55028        HVHSQAISASTVTSYNLTPDNIAYMFPLSCFAYWTGELVLCITNITTTPLMVSHQYANTT
AalV-A1         YYKALTVGANTVEDELLEVPMKGVLSMLRCFAYWSGELVLTVQNEGDDFMAVAHSYGDYT
                :    :: *. : .  :            *  .***:***:  : *      : .:*.*

AalV-B1         LNTSQAHMMANGCMVIPPRGVKNINLPFYSPTPLRRTI--------DTLGRFVFLSASSG
QKE55028        VDN-KAALSALGAMMVPPNGSKTMNIPFYSPTPFRKTVG------TDAFGTMYMFSESAG
AalV-A1         PAD-YEGLVAMGAIIIPPKTIKTVNVPFYSGTPLRVLRPGAMGAAQPAFGRLFMWSLNGT
                       : * *.:::**.  *.:*:**** **:*            ::* : : * ..

AalV-B1         KVELNFAFRNVSLFFPIPCKSG-ATTLQVSEYLKD---LTIDGDVESNPGPVFEQ----R
QKE55028        NVIISLALRNPQLAFPMPNKNYSVTNYSLGGWIQD---LTECGDVESNPGPNFEN----R
AalV-A1         SGHLYISLRKPQLFGLVPVPTTRNSLLLTSEGATNSSLLKLAGDVEENPGPLQIEGKPYR
                .  : :::*: .*    :*   .    :     .    :    *.   ****.****    :    *

AalV-B1         KIDLPGLGYVWMDHRSWKGFLIKNEVLYWS------------YHEGGFPSPAVISCHEA
QKE55028        LLEFKGF-YFAVSSNTTHGFLSKTNVRHLT------------CYTDGD--QFIFSRHTV
AalV-A1         VIQKGPNRREIWSTFTHNNLKVKSRVIETKGTLRIRRTTVQTCFNTRDLSCKPVSVMFRI
                 ::          .  : :.:  *..*   .                  .     :    .

AalV-B1         KRKFMWKSKLIVNLEFYHGQKTYWQKTIKN--RDWLGRLKVKKLEGDYVRDLTQEG-VEP
QKE55028        EVN----NLLTKYREDIFSTQAGSFSLIRK--KDWLGRRFSKIIERSHIKDLTTEG-IEP
AalV-A1         ENETPFGFDCQTMYDFITEQHRYVHYTIKKPFREPVTTSFEMPYDDPEWDRLLQAGDIEQ
                : :            :     :      *::  :: :         :      *   * :*
```

```
AalV-B1         NPGPSNFTYSEIN--NQTIRSRSYGESDSHYWENNYYTNGSYSAHLTKIHLKANGVPSPP
QKE55028        NPGPATYTVERNT--LTTRQIRSYDEDEEFYWTQDTYSGHKYAAKVLRVHLKSNGIPSKP
AalV-A1         NPGPDRVEEMRPYGKKITFSQRQGRFLAKHYIRQYEFNVVKREDILLTYYDVEGYWPETP
                ****      .      *   *.     ..*  :  :.  .    :   :   .  *. *

AalV-B1         IFTVKITVNVSWWSIKIRCVIMMRIFG-QWKIFRTKFRVKSRFFNQWSPIVFEENLPGEQ
QKE55028        VFTIKNTVIRCLRGNKVKTVITFDTAN-GTKVIKSVVPPG--------------------
AalV-A1         EATVHVYRTFLQHVIIENFLYELPVRPRGTKRFRIIVERD--------------------
                  *::            . :  :       *  :: .

AalV-B1         HHHDKFSSDVVGSQWVVIKSRGLGETAVSLSKTWEKLEVKIYPVQGVNPISVRVTDAGWV
QKE55028        ------------------------------------------------------HETPLMWH
AalV-A1         ---------------------GVEQTYMTGPYDALLQVYNALPTALKEIIPIPARPDPQW

AalV-B1         RDLTVDGDVESNPGPSNPMHCTKMDSKEWEHNGIRYYYTKYTKSFRWFGHIYEGDTSLIH
QKE55028        EDILRCGDVEQNPGPRKWLTARHS----YNLGEIRVRMVIHILISEEHGVVQLVGPVIYD
AalV-A1         NNLQQAGDVEMNPGPEFRKADTQMT----VDKKVTCFSTTVNKVTIQQEGFEVSKTVLLK
                .::   **** ****       :          :    .           .    . : .

AalV-B1         VMEIHPEGRRKDTFQLRDENRIEKWVFKCHDKCWVDDLTNCGDVEPNPGPYVIHNHWRVD
QKE55028        RNERAPGGKKR-------------------------------------------------
AalV-A1         KNTWFSRSHVETKVVYFKG--------------------------------------HLS
                     . .: .

AalV-B1         NINITEHIYNGGEVVHRVYVNWSNGAKQEVFYFRGEMFIFKCHEGC-----WVEDLTVDG
QKE55028        --RIVEYYYDPNEEPYRK--------------------IYPCHEEC-----WVRDLTMDG
AalV-A1         SMNVVDSSWTPFETKETRVCWVPQAGEPYVTTVVSRSGWFWRSEHFNQTGGWVPDLTQCG
                  .:.:  :   *                          :   *       ** ***  *

AalV-B1         DVESNPGPWTPDTRSIVILGAPGAGKSTVAN--EVIGAKCFRTRDQHNESTYCMSERTHG
QKE55028        DVESNPGP-----RKIVVVGPNQCGKTTLIR--DAFGVPVEPSKNKK----YSHDTYNTA
AalV-A1         DVESNPGPWDPERKHFCVVGPGGVGKSHVANLLTQTGMKLFKEACSPGPVTKEQETREFA
                ********     : : ::*.   **: : .     *        .        .    .

AalV-B1         QLTVYDTP--------ELPKVKDFVSGFIYVHRIGKFNTAEEVYIKMLDREIPNWQAHCV
QKE55028        FGEIIECR--------QVPKLVDLVDCFILVVRPG--LEGYRELIVDMERSYPNWLSHSV
AalV-A1         KRIITDTPCFSDIGQETKPKALDMYDCFVYVHQPGRFQKIEEKYEQELLKVFPNFWAHTV
                   : :            **  *: . *: * : *      .     : :  **: :* *

AalV-B1         LLLKQPEEEDLKVNPDIYLKECQPIGNLAFQMMGRTTDYVEVAKKMIVDCIHSPYFNHYY
QKE55028        IFFSQSP---TRQHPEKYLEKHG-FSSILFKCMGRVTDEPRVIEGLLKAMGNGPYFSHLF
AalV-A1         VVINFRGQEDEKAEQMQRYMEHTIYQKLFHKSQGRVL-MIDNIDKIDELINFPPYHQHFA
                :.:.       : .      :     .: .:  **.       . :       **..*

AalV-B1         RLVYKDRGIYRHYGIKHKGAVYHMNTGNILKSSLSGTAQVQLDYSTSGWIEAEPSEYRSA
QKE55028        KLVYKNRGAYRHYGIKNGSTVYHLNTGDILQSALEGTATVHLDDDPQSWIESDETGYRNA
AalV-A1         QLVYQNRGMYKHYGVKVGETVYHLNTSDIIESSLKGVARVQESEYSARWKPVDEVAYING
                :***::** *:***:*   :***:**.:*::*:*.*.* *: .  .  *   :   * ..

AalV-B1         YHLINSGAVEMEFNFDNNCESWARSVLGNTDEHQGARLKWCLTLAAAASFMFSGLVLEDQ
QKE55028        MHVVNAKLVNYKFSMDSNCETFARMFVDSDSPMQGDRIKWGLTLAAASAFLFCSSSFSDQ
AalV-A1         YNLFSSGAVEVEFNIDDNCDSWSRKMIGSNSPTQGARLKWCLSVAAAMAFISSMDMVSNE
                 ::..:  *: :*.:*.**::::* .:.. .  ** *:** *::*** :*: .   ..::

AalV-B1         SPGMLSKLLSSISGHFYKSLENLVIKTAIRTVCRIICYLILYCHSPNLLTTGVIVALIAM
QKE55028        SPGLFHKLISAISNHFYSNLENIVMKTVIKTVCRLVCYLILYCHSPNLLTTGTLVALISM
AalV-A1         SPGMFQRIITSISSHFYGNLENIVIRTVIRTVCRIVCYLILYCHSPNLLTTGVLVALIAM
                ***:: :::::**.*** .***:*::*.*:****::****************.:****:*

AalV-B1         DLTSIEIDENVKGACEALANGDFTKFCSDVIDMTGDPEAQSMKKYIPRFSNHG-TIMKLR
QKE55028        DLSSIEVDPKIRALCQSLADGEFAQFCSDVVELTGDPDYVELKNHIPKFANQSITLQKLQ
AalV-A1         DMTSINVDPKIKAACESLANGEFAEFCSSIVDLTDEPEYVELRNSIPRFNMHARSLNQLQ
                *::**::* :::. *::**:*:*::***.::::*.:*:  .::: **:*  :. :: :*:

AalV-B1         DEALS----------AVEEIEHSPDCKCYKCTHP-------------------------
QKE55028        EKAIYDKMKWEEEQGPIGVLDHPKDCRCFVCFESKA-----------------------
AalV-A1         RDEIDRQYEAGMGSIPCKHPLHQDGCPCIKCLPPRFHVETDKCECRFCVEKFGYLRERND
                 . :           .     *  .* *  *  .
```

```
AalV-B1          ------GDQSP-KSFNDWTTAAKNVKWWVESLIACFEWIRDKIFPSDAKNKILELEAKKD
QKE55028         ------SNEGP-KSFNEWSTAAKNVKWWVESLMTALEWIRDKLFPSNATKLVEELESKAN
AalV-A1          DGTRKVKNQGPAKGFNEWTTAAKNIQWWVEGVMKCLKWIKEKLFPPNMDKILRELEIKSS
                        ::.* *.**:*:*****::****.:: .::**::*:**.:  : : *** * .

AalV-B1          EIAIVMGLADEHICKCRTDKNYILHKDTPKKHALLVDKLLSLNIDELPTQMSYLQSKLNT
QKE55028         KIALIMSLADEHITKCRTDKQYVLSKDTVDKHRHLVSKLVELNVDSFPTQLAHFQGKINT
AalV-A1          EIATVMACADEHICKCRTDKVYVLDPKTRQRQQILVDKLSNLLSEELPSQLNHYTSKLNA
                 :** :*. ***** ****** *:*  .* .::  **.** .*  :.:*:*: :  .*:*:

AalV-B1          LLNRLQSITIEPPLEYNHRTEPLGIWIQGSPGCGKSFLSHYIVNQLQKRYGWEAYSHPIG
QKE55028         LLTRIQSLNLEPPLDYAHRPEPLGVWIQGSPGCGKSFLSNFLVKSLSKHYNWDAYTHPIA
AalV-A1          LMNRIQGLNLEPPLNYTHRPEPLGIWIQGEPGSGKSFLANFIVKSACERYGWSAYSQPIG
                 *:.*:*.:.:****:* **.****:****.**.*****::::*:.  ::*.*.**::**.

AalV-B1          SDHMDGYTDQEIHIFDDLGQNREEQDVALMCNLISSVPFIVPKANLESKGCRYNGKVVIA
QKE55028         SEHMDGYVSQEIHVFDDLGQNRQENDVALMCNLISSTPFIVPKANLESKGCRYQGRVVIA
AalV-A1          SDHMDGYTDQEVHVFDDLGQNRDEEDMALMCNLISSVPFIVPKADLTSKGTTYNGRIVIV
                 *:*****..**:*:********:*:*:*********.*******:* ***  *:*::**.

AalV-B1          TTNKRDFTTNKLLESAALQRRFPMVLEIRPKERYRREDVHNRIRFNAVTATGDQSLQNGE
QKE55028         TTNKRDFTSNILLDNSALQRRFPMVFEIRPKEKYRREDSFGRTRFNAVNATKDGSLERGD
AalV-A1          TTNKTDFTSLKLADAGALQRRFPIVLNVKPRQEYERVDSHQRVRFNAVNATMDGSLTRGE
                 **** ***:  * : .*******:*::::*::.*.* * . * *****.** * ** .*:

AalV-B1          CWEVNVDAINGVRS-VDCWQPLNPKQLIDDICADLDARMKVCTFMNQGKCKISLDSDGPD
QKE55028         CWEMNVDAKNQNRS-QDCWIPLDPKVLFEEICEEIDSRSAVANFMNQG-VDIEMESDEMT
AalV-A1          CWDRNVGAPKGLTTYADCWVPLKPDIMLKEIFEELESRDAVNKFMNQG-PTLVLDSDEVD
                 **: **.* :   :  *** **.*. ::.:*  ::::*  * .*****   : ::**

AalV-B1          MFSDMFPDPP-KKIEKFTNFVASSITSFKDFVERNKTWFIAAGALGTIISLASVTIPLIR
QKE55028         EFDKLFPQTP-KNFSRFRKYVGDSIASFKAFVERNRSWFLAAGALGTIISLSSFLIPIVR
AalV-A1          EFDKFFPDPPIQKVSKIKKFIHEMVAGVKGFVERHRTWFVAAGALGTVLSLISFLIPIIR
                  *..:**:.* ::..:: ::: . ::..* ****:::**:*******::** *. **::*

AalV-B1          KWMKGEKPEEENFYAGKVGPLKLKDYKLPLHNQGSIDMKTISKLLVTLEDIDGDLATGLA
QKE55028         KWLNKDSSDEN--YSGKPGPIKIQNYKLPLLNQGQINLKPILNSLVNVETENGEVSTAIC
AalV-A1          KIRGKTENDDESFYGGKMCPTKIKNYQVQMTNQGPVNMKPILKSLVNLQDRDGYRATGLA
                 *     . :::  *.**  * *:::*:: : *** :::*.* : **.::  :*  :*.:.

AalV-B1          IGDKSIVTFGHETFKKVVSFRDVDVDWELCDAQKITINGEHMDLVQYKANCDIQFKNVNY
QKE55028         IGNKEIITYGHDSFVRVSGFRDEKVMWNLNNPRRVTISDQSMDLTQYTVDTNIQFKNINH
AalV-A1          IGNKTVVTYGHDRFNTLVHLKEEQLDHQLGEPTAIRINGEKMDLVQYEVDCPFQFKSSNH
                 **:* ::*:**: *  :  ::: .:  :* :.  : *..: ***.** .:  :***. *:

AalV-B1          KIYEDDYRGDGYLVWKEKKHYLYLPVSNIHPTSTVITQQGTTTAHTYSYTGKTWKGLCGA
QKE55028         KIYEEDYNGSGYLLWKEGSSYTVLPVENIHATNTLITLEGTGTEHTYSYCARTWKGACGA
AalV-A1          RIYDGDYNGDGYLVWKEGNTYSYLPVSNIHATNEITTTDGTTTANTYTYIAKTWKGSCGS
                 :**: **.*.***:*** . *  ***.***.*. : * :** * :**:* .:**** **:

AalV-B1          VLVGIVNGNPKILGIHVAGNKSLGMSARLFPMFNQGKARVVGPNLNNYYQPRKTKFEKSP
QKE55028         VLVGIVNGNPKILGIHIAGNKCMGVAARLFPMFNQGKATKIA-DIRPYFQPRKSKLEPSP
AalV-A1          VLVGIVDGNPKILGIHIAGNKTLGCAARLFPMFNQGKVVHVEKTGIQYHQPRQTAYEPSP
                 ******:*********:**** :* :***********.  :      *.***::  * **

AalV-B1          VCSDDPKFGPAVLSNKDKRLEVEVEDVTKHVASKYIGNTFNPPRQAFQLAKAHVVSKLSQ
QKE55028         VYESS-NVAPAVLSKNDPRLDIMVDDVTKNAADKYTGNTFNPPKEYMSMAKAAVISKFSK
AalV-A1          VNTGHSTVGPAVLSKNDKRLEVEVEDVTKNAAAKYIGNVFDPPVPIFSLAKAVVIDKIRR
                 *  .  ...*****::* **:: *:****:.* ** **.*:**   :.:*** *:.*: :

AalV-B1          ILRVSSCMPYEKAITSDVLPIDWQTSSGLKYSGKKKEELVQLESFRQDVQEILDGSPTFF
QKE55028         VVKHKDVATYEEAIDSTILPIDWTTSSGHKYAPRKKMDLITDPQFRKDVMEVLETGSTYF
AalV-A1          VVKPSKCMTYDEAISVTELPIDWQTSPGLKYKGRTKADLVQDPKFKEDVKEILAGKPTFF
                 ::: ..  .*::**    ***** **.* **  :.* :*:   .*::** *:*   .*:*

AalV-B1          TCYLKDELRPLDKIALGKTRAIEAGNFDYVIAWRMVMGNMTIQLFNDYDRVSGFAPGFNP
QKE55028         TTYLKDELRPLSKVRVGKTRTIDAANFNYVIAYRMVMMKVVKQLFEDQDRITGFAPGVCP
AalV-A1          TTYLKDELRPIEKIASGNTRAIEAANFDHVVAWRQVMGNIVKQLFSDHDRVTGFAPGMNP
                 * ********:.*:  *:**:*:*.**::*:*:* ** ::. ***.* **::*****. *
```

```
AalV-B1         YTCWDSMMDQIKPNILALDFKNYDGSLSPQVMEEAVDILASFHTDPELVKRIHKPTIYST
QKE55028        YTHWDSLYDTIHSNVLALDFSGYDGSLSDTLMLEAVEVLSCFHEQPDLARFIHLPTIYSE
AalV-A1         YTHFDSLMDQVKWNVLALDFKKFDGSLSPQVMEEAVDILASFHDMPQMVKDIHKHTIYST
                ** :**: * :: *:*****. :*****  :* ***::*:.**  *::.: **  ****

AalV-B1         NLVSNEVWEVEGGMCSGSPCTTVLNTIVNLLVNYTILFDYGLSPSQTYVIGYGDDTVLST
QKE55028        NLVADEKWYIEGGMCSGSPCTTIINTICNLLVNYCVAFSYGLSMDEVHIAAYGDDTLFST
AalV-A1         NVVSDETWFVEGGMCSGSPCTTVLNTICNLLVNTTILLSEGIQPDNFYIAAYGDDTIISV
                *:*::* * :************::*** *****  : :. *:. .: :: .*****::*.

AalV-B1         D----EVFDCSDIVEKYRDYFGMNVTSADKTSEIKWEKKENVEFLKRTTGLFPGTAKMVG
QKE55028        T----RCFDVEGIEQRYMDWFGMKVTSSDKVSKITWQGKSQSNFLKRSPTVLYGTSKIVG
AalV-A1         DGLSSSLPDPKVMQQKYKEWFGMTVTSADKGSEITWDTRNHVQFLKRRPGFFPGTQKVVG
                        * . : ::* ::***.***:** *:*.*: :.: :**** . .: ** *:**

AalV-B1         KLNLENMLGHLDWTTGSFQSQLDSFYLELCLHGQQVYDGIFEKLSKKAPSYKHLTFGAAR
QKE55028        ALDIDSMMDHIQWTRGEFQSQLESFYLELCLHGPTVYGQIRSDLCKRAPSFNHPTFDWAY
AalV-A1         VLDLESMMEHIAWTKGSFQDQLNSFYQELVLHGEQVYMTVRQTLKSRAPQYNHPTFLAAY
                 *:::.*: *: ** *.**.**:*** ** ***  **  : . * .:**.::* **  *

AalV-B1         NMMKAVCLIY
QKE55028        NQMKFICQVY
AalV-A1         NIMKPIVMVY
                * ** :  :*
```

Alignment of Ori- and Avisivirus Polyproteins.

```
AsV-C1-45C          MEQVGAAIAAVSDAA--------ASLAENPVQEIIEGVGNLATTVSTNALVQTSNP--TV
AsV-C1-HUN          MEQVGAAIAAVSEAA--------ASLAENPVQEIIEGVGNLATTVSTNALVQTSNP--TV
AsV-C1              MEQVGAVVTAVSQAA--------SNLAENPVQEIIDGVGNLSTTISTNALVQTANP--TV
AsV-A1-HUN          MDPISEIAGAVTEVA--------KDLAPAPVNQIIEGVSNLTTTPSANSTIQTSAP--TV
AsV-A1-USA-IN1      MDPISEIAGAVTEVA--------KDLAPAPINQIIEGVSNLTTTPSANSTIQTSAP--TV
AsV-B1-44C          MEVLAAVNGAVADVNN------FATAATEIVTDAVTGVGSIQAAQSDNASVQSSHP--VQ
Orivirus-A1         METVKEIAAKTLAPLTNDVLSAAEQIAGLVVTEPTDDAHQITVNPSANSAIVNSTPPVSD
Orivirus-A2         METVKEIAAKTLAPLTNDVLSAAEQIAGMVVTEPTDDAHQITVNPSANSAIVNSTPPVSD
                    *: :      .              *   : :   .. .: .  * *: : .: *

AsV-C1-45C          ETGIPDSTNVISDDYLSCACTVDTDTMNVEKTILFGSDNWSSNQAFGTCISRYDVPDVFL
AsV-C1-HUN          ETGLPDSTNIISDDYLSCACTVDSDTMNVEKTILFGTDDWSSNHTFGTCISRYDVPNAFL
AsV-C1              ETGLPDSTDTITDDYLSCACTVDTDTMNVEKTILFGTDEWSANHRFGTCISRYDVPDVFF
AsV-A1-HUN          DTSIPHGTSQILDSFFSCGTVQETSVLNYEKMILLDQAEWGTTPDVSYCLLKISVPGAFF
AsV-A1-USA-IN1      DTSIPHGTSQILDSFFSCGTVQETSVLNYEKMILLDQAEWGTNPDVTHCLLKTSVPGAFF
AsV-B1-44C          QVGVADSTSGSTDDFLSCSLKVDTAKVNPAKAVLIGTATWTSNDVMYEVIENWDLPNVFF
Orivirus-A1         AELVSVQQTSSTDDMYSCAYEPSVGEENVTRYTEIMHGSYSTSNAPFDVIAHRLTPGCFF
Orivirus-A2         AELVEIQQVSSTDDMYSCAYEPSVGQENVTRYTELLQGSYSTSNSPFDVIAHRLTPGCFF
                       :        *.  **.   .    *  :   :    : :.      : .   *. *:

AsV-C1-45C          NSDSCPAYGQSSYFRFLRCGFRFQITTNPPPGAGGSLILAYMPPGFQFRVQQKGATITG-
AsV-C1-HUN          DSNSCPAYGQSSYFRYLRCGFRFQITTNPPPGAGGSLILAYVPPGFQFRVQPKGQTITG-
AsV-C1              NSDSCPAYGQSSYFRFLRCGFRFQVTTNPPPGAGGSLILAYVPPGFQFRVQQKGAQITG-
AsV-A1-HUN          SDATKPAHGISKYFRLLRCGYRFTVVLSVPPGACGALAMVFVPPGFTDKITQ-NQPVTA-
AsV-A1-USA-IN1      SDASRPAHGISKYFRLLRCGYRFTVVLSVPPGACGAVAMVFVPPGFNNKMTV-GQTVTK-
AsV-B1-44C          HDSNFPAYGQSRFFRFLRCGYRFHLTFNPPPGSQGCLVLSFVPPGYAHCIPAKGTATNWK
Orivirus-A1         DDDEDPAKGQCQRFCFLRCGFEFSLNVTTPMGGQGALVLLYLPPGFAGLVNSSSKLDNC-
Orivirus-A2         DDDEDPAKGQCQRFCFLRCGFEFSLNVTTPMGGQGALVLLYIPPGFANMVNASSKLENC-
                     .   ** * .  *  ****:.* :  . * *. *.: : ::***:   :   .   .

AsV-C1-45C          FDPEAVLTLPHVIVDIRSSTHSALTIPYVNHKNYFNYSYSGDHR------GTVIVFVLGQ
AsV-C1-HUN          FDSEAVLTLPHVIVDIRSSTHSALTIPYVNHKNYFNYAYQQDFR------GTVVVFVLGQ
AsV-C1              FDPEAVLTLPHVIIDIRSSTHSSLTIPYVNHKNYFNYSYGNDHR------GTVVVFVLGQ
AsV-A1-HUN          WDPEAILTLPHVVVDSRTSNTGTLTVPYVNYQSYCNLDQNGNQ-------AFVAVLVLGK
AsV-A1-USA-IN1      WDPEAVLTLPHVVVDSRTSNAGTLTVPYVNYQSYCNLEQNGNQ-------AFVAVLVLGK
AsV-B1-44C          FDTDALLTLPHVRCDARSTTMSSLVVPYINFNSYVDYTGSGTAT------AHIIVWVLGR
Orivirus-A1         -ARGSLFNFPHVIIDISLANSSTLTIPYMSYKNYFNITGSEQLTSPKMGSGRICVVALTK
Orivirus-A2         -ARGSLFNFPHVVIDISLSNSATLTVPYMSYKNYFNITGTETQTTPKMGSGRICVVALTK
                        :::.:***  *   :. .:*.:**:..:.* :              . : * .* :

AsV-C1-45C          YTVGSGTSSNVGVSVFGEMLEADFQCPRPYRVQGQNRRKIRRRRAPPPPANPPVGRHVNI
AsV-C1-HUN          YTVGSGTSSTVGVSVFGEMLEADFQCPRPYQSQGQNRRRIRRRKAPPPPSNPPVGRHVNI
AsV-C1              YTVGTGTSSNVGVSVFGEMLDADFQCPRPYRVQGQNRRKIRRRKAPPPPDNPPIGRHVNI
AsV-A1-HUN          YNSANGTSSSCDIALYGELLDTDFQCPRPVS-QG------KRRKAAKPEHNPTT-AMVSI
AsV-A1-USA-IN1      YNSGTGTSSTCDIALYGELLDTDFQCPRPVS-QG------RRRKASRPEQNPTT-AMVSI
AsV-B1-44C          YRCGTGTSTNIDYSVYGEMLDMDLQCPRPWD-----------------QGPTR---MVV
Orivirus-A1         YNCGASTTNSIQFTLFGQMLDADLQCPRPLNLNAQG--LQRVSPTKHTTVSFSHTPPMAI
Orivirus-A2         YNCGASTTNSIQFTLFGQMLDADLQCPRPLNLTAQG--LQRVSPEKHTTVSFSHNPPMAI
                    *  . .*:..   :::*::*: *:*****                    . .     : :

AsV-C1-45C          GPAPGAVIAANSVLNITTADSLAIGNEGTAVDCTTAGAASAIPDVRELASDWQILHQQSM
AsV-C1-HUN          GPAPGAVVAANSVLNVTTADSLAIGNEGTAVDCTTAGASSAIPDVKELASDWQILHQESR
AsV-C1              GPAPGAVVAGNSVLNVTTADSLAIGNEGTAVDCTTAGASSAVPDVKELASDWQILHQESR
AsV-A1-HUN          ASGPGSANLANSTLAPKLADSLAIANEGTAVDYSTAGCDQSVTDLIELARSWQIAAYGKL
AsV-A1-USA-IN1      ASGPGSANLANSTIAPRLADSLAIANEGTAVDYSTAGCDQSVNDIIELARSWQLAAYGKL
AsV-B1-44C          DPAPGAVMAGNSKIMN-DCQTVALAGEGTLVDNTTAGAKVAKTSLLSPARHWQIMASFDW
Orivirus-A1         NSTPGCVNLS-SFVTGNAAESTSLACEETMVDLKTAGARSAISDLKTVLRRWQVYAVSNV
Orivirus-A2         NSTPGCVNLS-SFVTGNAAESTSLACEETTVDLKTAGARSAVSDLRKVLRRWQVYAVKNL
                     . **..  . * :    .:: ::. * * ** .***.  :  .:      **:    .
```

```
AsV-C1-45C          SWAALNAG-----DRVWSGNFAPYEVGNIGALMDKFMYWRGSFEVQLVVYGSSLTSGRIQ
AsV-C1-HUN          SWAALTAG-----ARVWSGNFAPYEVGNIGALMDKFMYWRGSFEVQLVVYGSSLTSGRIQ
AsV-C1              SWAALTAG-----QRVWSGNFAPYEIGNIGALMDKFMYWRGSFEVQLVVYGSSLTSGRVQ
AsV-A1-HUN          DSAQKNS-------VVLNINFDPYSYGNLGLLFDKFQYWRGSLEVQFVMYSNSLASGRYQ
AsV-A1-USA-IN1      NSADKDT-------VILNMNFVPYSYGNLGLLFDKFQYWRGSLEVQFVMYSNSLASGRYQ
AsV-B1-44C          TTTGALG------AQIFKANFEPFSYGNIGFLFDKFLFWRGSLEIAVLAFGSSLTSGRFQ
Orivirus-A1         TLNTQGAVTVYPVNIVTAGTGTDVQNNSLFLICSNFQFFRGSLELRALVITSKGMSVKYK
Orivirus-A2         TLSTQGAVTTYPVNIVTAAGATDVQNNSLFLICSNFQFFRGSIELRALVITSKGMSVKYK
                                   :        . ..:  : .:* ::***:*:  :   ..  * : :

AsV-C1-45C          LSFYPGMSNN--NGRTLQQMRNAFYSTGDISAAPTRLTIPFTNDSWRRRCDQQYGSFYIH
AsV-C1-HUN          LSFYPGMPNN--NSRTLQDMRNAFYSTGDISATPTRLTIPFTNDSWRRRCDQQYGSFYIH
AsV-C1              LSFYPGLPND--NSRTLQQMRNAFYSTGDISATPTRLTIPFTNDSWRRRCDQQYGSFYIH
AsV-A1-HUN          LCWLPADWS---ADYSLAQLRNSIYSTGDVSSAPCTLVLPFTSQNWRRRCDGRYGSIIVR
AsV-A1-USA-IN1      LCWFPADWSDSSRAYTLAQLRNSIYATGDVSSAPCTLVLPFTNQNWRRRCDSNYGSITVR
AsV-B1-44C          VSWYPDLSAR---DLTVAQVRNSIFATGDISSVATRLTIPFTNPNWRRRCDSAYGSIYVH
Orivirus-A1         LGWFRSNTTS---TVTYTQTRNTLFVVGDSDGPPPVLTIPYSNDKFRCAVGGQYGTAYFA
Orivirus-A2         LGWFRSNTTG---TVSYSQTRNTLFVVGDSDGPPPILTIPYSNDKFRCAVGNQYGTAFFA
                    : :            :  : **::: .** .. .  *.:*::. .:*   .  **:  .

AsV-C1-45C          IVNRVCVNASASPTMSFVLFVRLGPDFQFFCPRYGDYHIQGPTVEKNPGEPEEGETYSGQ
AsV-C1-HUN          IVNRVCVNASASPTMSFVLFVRLGPDFQLFCPRYGDYHIEGPIVEKTPEESEEGETYSGQ
AsV-C1              IVNRVCVNASASPTMAFVLFVRLGPDFQFFCPRYGDYHIEGPVVPANSEVTDEGETYVGQ
AsV-A1-HUN          MVNRLAVNGSSTTHFSYALFVRAGQDLQFFAPRYGDYSIL--------QGPIEGETYNQT
AsV-A1-USA-IN1      MVNRLAVNGSSTTHFSYALFVRAGQDLQFFAPRYGDYSIQ--------QGPIEGETYNQA
AsV-B1-44C          SINRQTVNSTANPAIQMIILVRLGPDVDFFCPRYGDYHIQG-----------DDTELIDE
Orivirus-A1         VVNKTATNVICPTTCSVVLQIRAGPDFELSVPQYSDLKLQG-----------IGDGVDES
Orivirus-A2         VVNKTATNVICPTTCSVVLEIRAGPDFELSVPQYSDLKLQG-----------IGDGADES
                     :*:  .*  . .     : :* * *.::  *:*.*  :              .

AsV-C1-45C          PNVFLNFDCVEVPIQGASHTLVRNLFGRLWLQ-EHTVSPSSGPHVVKLEVPNESHA----
AsV-C1-HUN          PHVFLNFDCVEVPIHGASHTLVRNLFGRLWLQ-EHTVSPSSGTHVVNLEVPNESHA----
AsV-C1              PHVFLNFDCVEVPIHGASHTLARNLFGRLWLQ-EHTVSPATGTHVVNLDVPNESHA----
AsV-A1-HUN          STLSTNFEISDVVIHGSKHTQIDNFFGRSWVHGFHTSSSADTAMKLPLRTPRHSHG----
AsV-A1-USA-IN1      STLSTNFEISDVVIHGSKHTQIDNYFGRAWVEGFHTSTAASTAMKLPLQTPRHSHG----
AsV-B1-44C          VQSFLNFTIKEVPIQTASHTLIPNFFGRAFYYGKYTSPAETSASVIPLKVPQYGHG----
Orivirus-A1         ATCFVNFRLVDVPITTTPHTNVDAIFGRSVHMFDVTNQQGRYVITPMHNPRSDAHNKRST
Orivirus-A2         ATCFVNFRLVDVPITTTPHTNVDAIFGRSVHMFDVTNQTGRYVITPLHNPRADASNKRST
                         **   :* *  : **     ***       *                 .

AsV-C1-45C          -AILQCFAYFSGEVIISIRNGGDTTVIAAHTYIPEEQHNPVDEFSIMSLGAVIIPPLEIK
AsV-C1-HUN          -AILQCFAYFSGEIILSIRNGGDTTVIAAHTYVPEEQHDPTNEFSIMSLGAVVIPPLEIK
AsV-C1              -SILQCFAYYSGELILSVRNGGDTTVIAAHTYVPEEQHDPTNEFSIMSLGAVVIPPLEIK
AsV-A1-HUN          -SAMLAFAYWCGEVVITVHNRSENMLICAHSYDLEEQHSQVNEQSIFSLGAILVPPREVK
AsV-A1-USA-IN1      -SAMLGFAYWCGEVVITVHNRSQNMLICAHSYDLEEQHSQVNEQSIFSLGAILVPPREVK
AsV-B1-44C          -SLMTMFAYFTGEVVLTVHNRGTGLLVLAHTYIIEEQHNPSDESTIFSLGAVLVPPGEVK
Orivirus-A1         FNILSCFAYFAGELTITAVNFSTQNEAYIGHTYTTENAGDLN--ELINYGTIIVPPGGVK
Orivirus-A2         FNILSCFAYFAGEITITVVNFSKLNEAYVGHTYTRENAGELN--ELINYGTIIVPPSGIK
                       :  ***: **: ::  * .             *: .  :   ::. *::::**  :*

AsV-C1-45C          IIRVPFYSPSPLRMIRRHRDFEPTFGYLYLCSPSTSNVTVYMGLANPNLFFKLPCPQYTV
AsV-C1-HUN          TIRVPFYSPSPLRMIRRHNTFEPTFGYLYLCSPSTTNVTVYMGLANPNLFFKLPCPRYTT
AsV-C1              TIRVPFYSPSPLRIVRRGNGFEPTFGYLYLCSPSTTNVTIYMGLANPNLFFKLPCPRYTI
AsV-A1-HUN          TFRAPWYSQTPLRRP-LDNDDEPSMGFLYVTSEGSSNFTVYLALHKPKFFFPLPCPRFTS
AsV-A1-USA-IN1      TFRAPWYSQTPLRRP-LDDPNEPSMGFLYVSSEGTSDFTVYLALHKPKFFFPLPCPMFTS
AsV-B1-44C          TFACPYYAHTPLRPLRLEDT--PAFGYLYASGEGAIPFTVYISLRDPKFFLDMPQPAFTS
Orivirus-A1         TFNAPFYSETPFRVL---NGEGALGYFLSYCADSTATIRVYASLRNCQFVGMAASANYT-
Orivirus-A2         TFNAPFYSETPFRAL---NGEDALGYFLSYCADATATIRVYASLRNCQFVGLAPSANYT-
                     :  *:*: :*:*         .   :*   . .:  . :* .* . ::.   . . :*
```

```
AsV-C1-45C          NSR------------------AAITSAGPRFFR------EDDDHYNILLGGDIEENPGP-
AsV-C1-HUN          NTR------------------SRSARSAPRFFR------EDDDHYNILLGGDIEENPGP-
AsV-C1              NTR------------------AISTRSAPRFFR------EDDDHYNILLGGDIEENPGP-
AsV-A1-HUN          NSAKVAPKGTGMSIAERKIQLTSVARRTLEWARREVGAYDEVDHRDILMGGDIEENPGPQ
AsV-A1-USA-IN1      NQSREAPRQP-KSIAERKIELSSVARRTLEWARREVGAIDETDHKDILLGGDIEENPGP-
AsV-B1-44C          NTRAAGARVG-----RQGYVRSAIRLPLPQFEK------ERSAHEDVLLGGDVESNPGPV
Orivirus-A1         ------------------------------------------------------------
Orivirus-A2         ------------------------------------------------------------


AsV-C1-45C          ------------------------------------------------------------
AsV-C1-HUN          ------------------------------------------------------------
AsV-C1              ------------------------------------------------------------
AsV-A1-HUN          RYQPLHKPEPSSNLLRAVSRRAIMSMVEENIQLT--PITRKAKVKVLDWVRREMGVFDET
AsV-A1-USA-IN1      ------------------------------------------------------------
AsV-B1-44C          ELQNGQQACVGFAPGPISGKEYKMQDYQHSAVLAGEAALVTHQGRDALYWFRSESVQYLE
Orivirus-A1         ------------------------------------------------------------
Orivirus-A2         ------------------------------------------------------------


AsV-C1-45C          -------------------VVVCGPSKGGKTRLLCAMAGHPIVPS--FPGPHVANMLGEE
AsV-C1-HUN          -------------------VVVCGPSKGGKTRLLCAMAGHPIVPN--FPGPHVANMLGEE
AsV-C1              -------------------VVVCGPSKGGKTRLLCAMAGHPIVPS--FPGPHVANMLGEE
AsV-A1-HUN          DHRDILLGGDIEENPGPQSIYFLGLSGCGKSRLVNAIAGHPLCDSRLSPTPIHTETHSHQ
AsV-A1-USA-IN1      -----------------QSVYLLGLSGCGKSRLINAIAGHPLCDSRLSPNPIHTETHSHQ
AsV-B1-44C          PQIDICVCGDVERNPGPK-IVVVGRSGSGKSRLCNMILGHDYFPSRLSSTPVTTKMRTAT
Orivirus-A1         ------------------------------------------------------------
Orivirus-A2         ------------------------------------------------------------


AsV-C1-45C          FT------EIPEDMKLPLNKKILVVLGEDN---DANANYIQWLSEEYPSWNHRAVVFC-W
AsV-C1-HUN          FT------EIPEDMKLPLNKKILVVLGEDN---DANANYIQWLSEEYPSWNHRAVVFC-W
AsV-C1              FT------EIPEDMKLPLNKKILVVLGEDN---DANANYIQWLSEEYPSWNHRAVVFC-W
AsV-A1-HUN          LMG----YEVFEQVGIPPAGGKYVYLQEATRFDKEHVDFIREMDQQHPGWRRHAVLYVNR
AsV-A1-USA-IN1      LMG----YEIFEQVGMPPAGGKYVYLQEATRFDKEHVDYIREMDKLHPGWRRHAVLYVNR
AsV-B1-44C          LPCGTQIVDTPERFSIQEEIKGFIWVLEEGRWTQENKDFLAYMDTSYPGWRRHCVIYHTR
Orivirus-A1         ------------------------------------------------------------
Orivirus-A2         ------------------------------------------------------------


AsV-C1-45C          PGCQLQGNNFR----------ICRNPIAVSALLAQSLPYNTRGTQLVYQDRGLYRHYGVL
AsV-C1-HUN          PGCQLTGNNFR----------ICRNPIAVSALLAQSLPYNTRGTQLVYQDRGLYRHYGVL
AsV-C1              PGCQLQGNNFR----------ICRNPIAVSALLAQSLPYNTRGTQLVYQDRGLYRHYGVL
AsV-A1-HUN          LGDTKLSQYLRGVPELAGFKEVTDNPLSVIPLLTTINSYEGTGVQLVCKNRGVYKHFGVR
AsV-A1-USA-IN1      LGDTKLSQYLRGVPELAGFKEATDNPLNVIPLLVSIDTYVGTGVQLVCKNRGVYKHFGVR
AsV-B1-44C          HEDPGSNFPQFLKDAGLSSFQWSKNPLDLTSRFLMIPPYRNCLVQLVFKDRGLYKHYGAR
Orivirus-A1         -----------------------VAPIAASAALSDQGPKAHKLLYKVTPSR----QYCVE
Orivirus-A2         -----------------------VAPISP-PTVSNQGPRTPRLVYKVTPNR----QYCVA
                                             *:   . .    .        *  .*    :: .

AsV-C1-45C          FDNKVFHLDSQDILKSGLKGSAVVSVDDP-LEWVPCSATDLAGSLDLANSGTIE-IDFNI
AsV-C1-HUN          LDNKVFHLDSQDILKSGLKGSAVVSVDDP-LEWVPCSATDLAGSLDLANSGTIE-IDFNI
AsV-C1              FDNKVFHLDSQDILKSGLKGSAVVSVDDP-LEWVPCSATDLAGSLDLANSGTIE-IDFNI
AsV-A1-HUN          EGDKVYHINTENLVKTCLDGQVAVMVEDYSAGWIPCSHEEKVGAVSFVQTGTLDGVTFSC
AsV-A1-USA-IN1      EGDKVYHINTENLVKTALDGEVAVMVEDYSDGWIPCSPEEKIGAVSFVQTGTLDGVTFSC
AsV-B1-44C          IGARIFEVNSDNLLSALTKKEVPIVSTPDDGSWQVAEDLFTPEAQRLAKNLELEKVKYGF
Orivirus-A1         VSGRTYAMSVTGAIEEVDGPSVVAILRPVDPEFLEAVMKHLS-----FDHRALATLHHNH
Orivirus-A2         VGDKLYALSASGTVEQVVSDSVAAVLRPVDPEFMEAIIKYLS-----FDYTALSVLHHNP
                     . : : :.  . :.     ..          :  .            .   :  : ..
```

```
AsV-C1-45C          NSNCETWAKGVIGDLSPTQSDRLKKVLVVAAAAGFLYCLPHDQ-SGFMDGVTKCLINLFS
AsV-C1-HUN          NSNCETWAKGVIGDLSPTQSDRLKKVLVVAAAAGFLYCLPHDQ-SGFMDGVTKCLINLFS
AsV-C1              NSNCETWAKGVIGDLSPTQSDRLKKVLVVAAAAGFLYCLPHDQ-SGFMDGVTKCLINLFS
AsV-A1-HUN          DFNCETWAKIFVPSEGETQGQRLKKVMAIAAGAAFVYGLPRGEGFDFMQCVTKVMMTLFS
AsV-A1-USA-IN1      DFNCETWAKIFVPSEGETQGQRLKKVMAIAAGAAFVYGLPRGEGFDFMQCVTKVMMTLFS
AsV-B1-44C          DANCETWAKDVLGVATPCQSHVVRKACCIAVLASLGVLTLSSQDVNVMGSITSVIVSLFS
Orivirus-A1         AKLYDMLVTAFIYTCSDQAPD--------------------------MTAMITRAMAVFQ
Orivirus-A2         QKLYDMLVTAFIYTCSDQAPD--------------------------MTAMITRAMAVFQ
                        :  .. .:        .                          *  : .  : :*.

AsV-C1-45C          KQVKSAMIRMAIKFVCRLVCYLILYCHSPNILNTGVLTTLLLMDVFELEVDEGLDKLAHA
AsV-C1-HUN          KQVKSAMIRMAIKFVCRLVCYLILYCHSPNILNTGVLTTLLLMDVFELEVDEGLDKLAHA
AsV-C1              KQVKSAMIRMAIKFVCRLVCYLILYCHSPNILNTGVLTTLLLMDVFELEVDEGLDKLAHA
AsV-A1-HUN          KQVKTIVVKMVIKFFCRLCCYLVLYCHSPNLVNTAMLTILLTLDVFDTEIDEISGKVAKA
AsV-A1-USA-IN1      KQVKTIVVKMVIKFFCRLCCYLVLYCHSPNLVNTAMLTILLTLDVFDTEIDEISGKVAKA
AsV-B1-44C          KQIKTAVVRLAIKFVLRLVCYLVLYCHSPNLINTAMLSALLVMDVFDNELDEAISKFAQL
Orivirus-A1         DLVANDVVRLVLKTLVKALCYGIICVTATSVAPLAACAALALVDLSEIRLGAVAQGISEA
Orivirus-A2         DLVANDIIRLVLKTLVKALCYGIICVTATSVAPLAACAALALVDLSEIRLGAVAQGISEA
                    . : . ::::.:* . :  ** ::   :..:   .  : *  :*: : .:.     .:.

AsV-C1-45C          LIEGDFKGLGKFLKKRTGRDCDDFEPGDDHRPIFRAEGPDDLPKTFNSWSLMAKNVEWWI
AsV-C1-HUN          LIEGDFKGLGKFLKKRTGRDCDDFEPGDDHRPIFRAEGPDDLPKTFNSWSLMAKNVEWWI
AsV-C1              LIEGDFKGLGKFLKKRTGRDCDDFEPGDDHRPIFRAEGPDDLPKTFNSWSLMAKNVEWWI
AsV-A1-HUN          LVSGDFKAAGRAMMEAADRKCEDFK--CEAKELFKSEGPDATGKSFNTWTLVAKNLEWWV
AsV-A1-USA-IN1      LVSGDFKAAGRAMMEAADRKCEDFK--CEGKRTLQSEGPDATGKSFNTWTLVAKNLEWWV
AsV-B1-44C          CIKGDFKGLGRWVKGKVGSDCQDFD-CPENRPIFNSEGP----KDFNDWSLVAKNVKWWL
Orivirus-A1         LVEGDIMGLVTVVLESIQGVDKDEIKATVMAASEAMGDQAPSLKGFTDFTNACKTVGWWL
Orivirus-A2         LVEGDIMGLVTLVLESVQGVDKDEVKATVDAASDAIGDQGPSLKGFTDFTNACKTVGWWL
                     :.**: .    :        .*              .     * *. ::  .*.: **:

AsV-C1-45C          TKFADFCKWLKEKVFPENHEDQIEQVEKLKDTLALTICQADKHLVAMRTDRDYATSYEAN
AsV-C1-HUN          TKFADFCKWLKEKVFPENHEDQIEQVEKLKDTLALTICQADKHLVAMRTDRDYATSYEAN
AsV-C1              TKFADFCKWLKEKVFPENHEDQIEQVEKLKDTLALTICQADKHLVAMRTDRDYATSYEAN
AsV-A1-HUN          DKLKQFINWIRTKLFPSDAKDKIEAMESVRDRMVMSLAAADKHLVTLKADKAYATSKAAR
AsV-A1-USA-IN1      DKLKQFINWIRTKLFPSDARDKIEAMESVRDRMVLSLAAADKHLVTLKADKAYATSKAAR
AsV-B1-44C          EQFKSFFSWMRDKVFGGD-SDQVDEVESRFEQITTTLAQCDAHLVAMATDKAYATGKSAR
Orivirus-A1         ETAIKIIKTVKDRLFSTRVKEASEWLGANRTRLYAFIASVDVHLTKCATNPEYASDPSTQ
Orivirus-A2         ETAIKIIKIVKDRMFSQRVKQASEWLATNRTRLYGFIAAVDVHLTKCSTNPEYASDPATQ
                        .: . :: ::*     :  : :      :   :.  * **.   ::  **:.  :.

AsV-C1-45C          IYHQNLMVKLVDLNAKDWGPDFRELSIKLGQVLQRMQAVNFESSNMNGLRAEPVGIWISG
AsV-C1-HUN          IYHQNLMVKLVDLNAKDWGPDFRELSIKLGQVLQRMQAVNFESSNMNGLRAEPVGIWISG
AsV-C1              IYHQNLMVKLVDLNAKDWGPDFRELSIKLGQVLQRMQAVNFESSNMNGLRAEPVGIWISG
AsV-A1-HUN          DYHLKITNEIIDLNAMDLGPDFRDLGTKIGQILNRLQSVTFDSVDAGSMRQEPLGIWISG
AsV-A1-USA-IN1      DYHLKITNEIIDLNAMDLGPDFRDLGTKIGQILNRLQSVTFDSVDAGSMRQEPLGIWISG
AsV-B1-44C          QYHEVLSARLRDLSLLPLGGPLSQVSQKINYMQGRMGKVTFDCVEAGSARMEPLGIWISG
Orivirus-A1         AIHLKLQSDLIKCREKFLETPFTDIKNLVSGAITRMERVKLTPVTMRPYRPEPYGVWIQG
Orivirus-A2         AIHLKLQSDLIKCREKFIETPFSDIKNLVNGAIARMERIKLTPVTMRPYRPEPYGVWIQG
                      *  :   : .         : ::   :.    *:  :.:        * ** *:**.*

AsV-C1-45C          GPGCGKSFLAQILIKHLRVAHGFSTYNHPTGSEHMDGYTGQEVHYIDDMGQIREEEDMKL
AsV-C1-HUN          GPGCGKSFLAQILIKHLRLAHGFSTYNHPTGSEHMDGYTGQEVHYIDDMGQIREEEDMKL
AsV-C1              GPGCGKSFLAQILIKHLRVAHGFSTYNHPTGSEHMDGYTGQEVHYIDDMGQIREEEDMKL
AsV-A1-HUN          EPGCGKSFLSHLIIKHLKEKKGFSVFCNPSGSDHMDGYNGQEIHYFDDLGQIREEADIKL
AsV-A1-USA-IN1      EPGCGKSFLSHLIIKHLKEKKGFSVFCNPSGSDHMDGYNGQEIHYFDDLGQIREEADIKL
AsV-B1-44C          GPGAGKSFLAQRIMKELKTSLGYDVYYHPTGSKHMDGYNGQEIHYIDDLGQLREEGDVAL
Orivirus-A1         SAGAGKSFLSGLIATAVRKQFGFSTYYHPVASAHMDGYGSQEIHVFDDFGQCRDEADYTL
Orivirus-A2         APGAGKSFLSGIISTAIRKHFGFTTYHHPVASAHMDGYGSQEVHIFDDFGQCRDEADYTL
                     .*.*****:  : . ::   *: .: :* .* ***** .**:* :**:** *:* *  *
```

```
AsV-C1-45C          LCQLISSQPFIVPKAELLSKGTQYRAKIVIATTNRTSFDTMVLSDTGALQRRFPIRLKIR
AsV-C1-HUN          LCQLISSQPFIVPKAELLSKGTQYRAKIVIATTNRTSFDTMVLSDTGALQRRFPIRLKIR
AsV-C1              LCQLISSQPFIVPKAELLSKGTQYRAKIVIATTNRTSFDTMVLSDTGALQRRFPIRLKIR
AsV-A1-HUN          MCQLISSQQFIVPKADLTSKGTLYNAKVVIATTNKNEFDSTVLNDSGALRRRFPIRLHVR
AsV-A1-USA-IN1      MCQLISSQQFIVPKADLTSKGTLYNAKVVIATTNKNEFDSTVLNDSGALRRRFPIRLHVR
AsV-B1-44C          LCQMISSASFIVPKADLNSKGTLYNSKVVIATTNRTSFDTQVLTTPDALRRRFPIELSIR
Orivirus-A1         VCNLISTSPFIVPKAELEAKGTYYNGRVVITTTNRMDFTSHKLFDPEALARRFPMSLHIR
Orivirus-A2         VCNLISTSPFIVPKAELEAKGTYYNGRLVITTTNRMDFTSHKLFDPEALARRFPLHLHIR
                    :*::**:  ******:* :*** *..::**:***: .* :  *  . ** ****: * :*

AsV-C1-45C          AHSFYTKADGTLDVALAMERKAFEDGSCWEINVGNDSRPCWQTLNWEVLTDEIDRMVATR
AsV-C1-HUN          AHSFYTKADGTLDVALAMERKAFEDGSCWEINVGNDSRPCWQTLNWEVLTDEIDRMVATR
AsV-C1              AHSFYTKADGTLDVALAMERKAFEDGSCWEINVGNDSRPCWQTLNWEVLTDEIDRMVATR
AsV-A1-HUN          PHSFYTTQDGRLDLNRAMKDGDIDPG-CWEIDVGS-GRSCWQTLNWDILIHEIEDELINR
AsV-A1-USA-IN1      PHSFYSTQDGRLDLNRAMKDGDIDPG-CWEINVGT-GRSCWQTLNWDILIHEVEDELINR
AsV-B1-44C          PHAFYCTLDGRLDMHKVMVEKAWDDCSCWEVNVSKDGRPCWQTINWDVLKDEISKQLATR
Orivirus-A1         PRPEYSTPKGHLDVVAAIKDK------KWNNVWEVKTDKGWCSLNVDFMLGKIMDEIEAR
Orivirus-A2         PRPEFTTPKGHLDVVAAIKEK------KWNNVWEIKTDKGWRSLNVDFLLGKVIDEIEAR
                    .:. : . .* **:  .:          *:          * ::* :.:  ::   :  *

AsV-C1-45C          SSIASLFNQGARCDLESDEVELIPESGPGSVNPKTMDKVKNWLNSLLSDALSWWERNKQW
AsV-C1-HUN          SSIASLFNQGARCDLESDEVELIPETGPGSVNPKTMDKVKNWLNSLLTDALSWWERNKQW
AsV-C1              SSIASLFNQGARCDLESDEVELIPETGPGSVNPKTMDKVKNWLNSLLSDALSWWERNKQW
AsV-A1-HUN          EHINKFFSQGA--IFESDEVEVVPEQGPGSVNKSTVTKLKNWINSLIDRAKSFFERNKCW
AsV-A1-USA-IN1      ENINKFFSQGA--IFESDEVEVIPEQGPGSVNKSTVTKLKNWINSLIDRAKSFFERNKCW
AsV-B1-44C          MSVLTFFSQGP------SLFECEEEEDEAPYSEQTKVRVSSWLRQLLDKARGFFERNKCW
Orivirus-A1         KEIVNIMDQGD-WIVKYDGQEVLFSVDEVEQQPQLSDKLKRYIDRAITAATNFVTQNKWA
Orivirus-A2         KEVINMMDQGD-WIAKYEDQQVIFSVDEVEPQPQLTQRLKKVIDRAIDAASNFITQNKWA
                      : .::.**       .  :   . .    . .   ::.  :   :  * .:  :**

AsV-C1-45C          LLLVSALSTLASLAIGAIPAYRAIQNQLYHGEPSAKPKDRVKRDFKPEGPN-------FH
AsV-C1-HUN          LLLVSALSTLASLAIGAIPAYRAIQNQLYHGEPSAKPKDRVKRDFKPEGPN-------FH
AsV-C1              LLLVSALSTLASLAIGAIPAYRAIQNQLYHGEPSAKPKDRVKRDFKPEGPN-------FH
AsV-A1-HUN          FYLGSALATLATLVTTALPAARSYLSNLYSGEPTRAKVTRVTREFQSEGPS-------YY
AsV-A1-USA-IN1      FYLGSALATLATLVTTALPAARNYLSNLYSGEPTRAKVTRVTREFQSEGPS-------YY
AsV-B1-44C          LVGISAVATLLSVAVSVAPKLFGNSS-VYEGSP-QQLKPKVYREFRSEGPN-------LF
Orivirus-A1         FVAFGALSALCATAAVYVTKYK-EDEGAYSGGAPRNPRPKQYRDMKAPVSNQNLLNSVVE
Orivirus-A2         FAAFGALSTLCAAAAVYVTKFK-EDEGAYSGGPIRNPRPKQYRDLKAPVSNQNLLNSVVE
                    :   .*:::* : .    .      .  * * .      :  *:::.  ..

AsV-C1-45C          SLKDRMVEIGTSHSTGLLLCDKKVLTFGHNTDCGFITHKD--QTFKVTSETYISVSGCDQ
AsV-C1-HUN          SLKDRMVEIGTSHSTGLLLCDKKVLTFGHNTDCGFITHKD--QTFKVTSETYISVSGCDQ
AsV-C1              SLKDRMVEIGTSHSTGLLLCDKKVLTFGHNTDCGFITHKD--QTFKVTSETYISVSGCDQ
AsV-A1-HUN          SLKDRLVEVGETGSTGLALGGKVVLSFGHNDDSKFIIYKD--QEHPVVKEENISVNNSPQ
AsV-A1-USA-IN1      SLKDRLVEVGETGSTGLALGGKVVLSFGHNDDSKFITYKD--QEHPVVKEENISVNNSPQ
AsV-B1-44C          HLKDRLVEVGSSGSTGLILGGKQVLTYGHNMDRSFIKHKD--IVYSVTKVEWIKVNDSEQ
Orivirus-A1         SAILEVVDVTGHRSTALAIGKKHLVSYSHGPELTRITFYKGPTSIPIEYAFNINYDGEPT
Orivirus-A2         NAIIEVVDITGHRSTALVIGKKHIVSYSHGPELVRISFYKGPCSIPIEYAYNVNYDGEPT
                        .:*::    **.* :  * ::::.*. :   * . .      :     :. ..

AsV-C1-45C          DLKILEVETPYQFKNCSHKIYSGNYKGDGNLIFLRNNQLIIKDVFRIREKQGIGTIDGTY
AsV-C1-HUN          DLKILEVETPYQFKNCSHKIYSGNYKGDGNLIFLRNNQLIIKDVFRIREKQGIGTIDGTY
AsV-C1              DLKILEVETPYQFKNCSHKIYSGNYKGDGNLIFLRNNQLIIKDVFRIREKQGIGTIDGTY
AsV-A1-HUN          DLALLTVQTPYQFKELRRKIYADVYRGDGFLLFLKKGTLIAHQVKRITPCDNIMTQQGHQ
AsV-A1-USA-IN1      DLALLTVQTPYQFKEIRRKIYADVYSGDGFLLFLKKGTLIAHQVKRITPCDNIMTQQGHQ
AsV-B1-44C          DLAILTIDTNLQFKQLVNKVYSGEYHGDGYLLYFRDNSLLATQVTGIRPFDPIATQEGHI
Orivirus-A1         DLVVYKVTGPTQLTSP-YTHFSDQLGQHPIMVSRRGGSLTVRPVDKIQPGGQITTLQGTT
Orivirus-A2         DLVVYKVTGPVQLSSP-YTHFSEHIGLHPIMVSRRSGTLSIRPVDKIQWGGQITTLQGTT
                    ** :  :    *:..   . ::     .  ::  : . *    *  *     * * :*
```

```
AsV-C1-45C          THSAYAYSARTGSGSCGGILVGYVSGNPIILGMHVAGNGDTGIAARLYPCFAQGVTMHKW
AsV-C1-HUN          THSAYAYSARTGSGSCGGILVGYVSGNPIILGMHVAGNGDTGIAARLYPCFAQGVTMHKW
AsV-C1              THSAYAYSARTGSGSCGGILVGYVSGNPIILGMHVAGNGDTGIAARLYPCFAQGVTMHKW
AsV-A1-HUN          TQFAYRYQVNSASGWCGGVLVGIVGGNPMILGMHVAGNGSHGIAARIFPNFSQGIVTQRM
AsV-A1-USA-IN1      TQFAYRYQVNSASGWCGGVLVGIVGGNPMILGMHVAGNGSHGIAARIFPNFSQGIVTQRM
AsV-B1-44C          TCRTYCYHAKTARGSCGGVLVGMVGGNPMVLGLHVAGNGHQGIAARVERYLWQSQGTVVK
Orivirus-A1         SSRTARYLGYNKEGDCGNVILTMHNGNYRILGIHTAGNGCVGYCNIVYGVVSQGQVLQRR
Orivirus-A2         SARTARYLGYNKEGDCGNIILTMHNGNYRFLGLHTAGNGCVGYCNIVYGVVCQGLVIQKR
                    :  :  *    .  * **.:::   .**  .**:*.****  * .  :   . *.

AsV-C1-45C          KQEQMFDTNYHQPRRSKFSPSCFFD-TGAQEPAILSNRDPRNPG-VADITKHNADKLTGN
AsV-C1-HUN          KQEQMFDTNYHQPRRSKFSPSCFFD-TGAQEPAILSNRDPRNPG-IEDITKHNADKLTGN
AsV-C1              KQEQMFDTNYHQPRRSKFSPSCFFD-TGAQEPAILSNRDPRNPG-IEDITKHNADKLTGN
AsV-A1-HUN          PN----TELYFQPRRSEIYPSPANDGTSNVEPPVLSNRDRRLETPIDDITKHNADRHKMN
AsV-A1-USA-IN1      PN----TELYFQPRRSEIYPSPANDGTSSVEPPVLSNRDKRLEEPIDDITKHNADRHKMN
AsV-B1-44C          IEP---GTVYHQPRRSRIVPSPVYC-DSALAPAVLSRADPRLEVPVEDITKRAAAKYVGN
Orivirus-A1         QS----DKVVYVPHQTNLIKSPCWVPSCELEPAALSGRDPRLEE-PRDLLVSNCDKYTGN
Orivirus-A2         ET----DKRVYVPHQTNLIKSPCWTPACDLEPAALSSRDPRLEE-PRDLLVANCAKYTGN
                              . *:::.:  *          *. **  * *      *:    . :   *

AsV-C1-45C          VFDPPEDAFALAKSRLIGSMSAHIEPEGQATFEEAVSSELLPIDWGTSPGDKYRGKTKAE
AsV-C1-HUN          VFDPPEDAFALAKSRLIGSMSVHVEPEGQATFEEAVSSELLPIDWGTSPGDKYRGKTKAE
AsV-C1              VFDPPEDAFALAKSRLIGSMSAHIEPEGQATFEEAVSSELLPIDWGTSPGDKYRGKTKAE
AsV-A1-HUN          RFNPPMDAFQVAKSNVISELASIVEPCYHMTYDQAVDSTLLPIVWTTSPGLEFKGKTKRQ
AsV-A1-USA-IN1      RFNPPMDAFQVAKANVISELASIVAPCYHMTYDQVVDSSLLPIDWTTSPGLEFKGKTKKQ
AsV-B1-44C          IFKPPEDCFIAAKAHVTRLLSTVVPPVGSLEYREAIDNSILPIDWSKSPGIKYKGMSKRQ
Orivirus-A1         IFEISTELCIDTVASVTSRLMEYGP---YAPVDYETSFTIVDMDWNTSPGHKYHNTCKQM
Orivirus-A2         VFDITTELITDTVATVTGKLMEYGP---FSPVDYETSFTILDMDWGTSPGHKYHNTTKDA
                     *. . :    : : :   :                 .  :: : * .*** :::.  *

AsV-C1-45C          LVDDKKFRADVHNLVKRFNGDPNREPVDVYFTCYLKDELRPKEKARACKTRVISAANWDY
AsV-C1-HUN          LVDDKKFRADVYNLVKRFNGDPDREPVDVYFTCYLKDELRPKEKARACKTRVISAANWDY
AsV-C1              LVDDKKFRADVYNLVKRFNGDPNREPVDVYFTCYLKDELRPKEKARACKTRVISAANWDY
AsV-A1-HUN          LVDDPGFKERVMKLYRSFAGG-NSAPPQVKYTTYLKDEVRIKEKVKKGATRTITASSFDY
AsV-A1-USA-IN1      LIDDPAFKERVMKLYKSFAGG-NSAPPQVKYTTYLKDEVRSKEKVKKGATRTITASSFDY
AsV-B1-44C          CVQDQSFKRDVLHLLL-------AQNPEVEFVTYLKDELRKLEKIKQGKTRSIEAASFDY
Orivirus-A1         LYESETFRSDVAKMLE---------VPTTYFVALLKDELRTREKVRKGKTRVIEAANFDY
Orivirus-A2         LHGSASFKQDVQDMLA---------VPSTYFVALLKDELRTKEKVRKGKTRVIEAANFDY
                       .  *:  * .:              . :.  ****:*  ** :   ** * *:.:**

AsV-C1-45C          TIATRMVAGPILRQLYAW-GREFGFGPGLNPYSHFDDLYDKILPFVICLDFKGFDGSLSS
AsV-C1-HUN          TIATRMVAGPILRQLYAW-GREFGFGPGLNPYSHFDDLYDKILPFVICLDFKGFDGSLSS
AsV-C1              TIATRMVAGPILRQLYAW-GREFGFGPGLNPYSHFDDLYDKILPFVICLDFKGFDGSLSS
AsV-A1-HUN          TIACRMIFGNIFRQLFGN-GLPAGFAPGMNPYTQFDELYDSCWLNVICLDYSKFDASLSK
AsV-A1-USA-IN1      TIACRMIFGNIFRQLFGN-GLPAGFAPGMNPYTQFDELYDSCRLNVICLDYSKFDASLSK
AsV-B1-44C          TIACRMLFGQIMMHLFVK-GREVGFGPGINPYTEFDELFDRLHPHCLEIDYSGFDGSLSR
Orivirus-A1         VVAYRMVMGEFLARVIEDPEKRAGICLGLNPYTDFSAIVNSLYQYNLCLDFKGFDGSLSE
Orivirus-A2         VVAYRMVMGEFLARVIEDPEKLAGICLGLNPYTDFSCMVNSLYEYNLCLDFKGFDGSLSE
                    .:* **: * :: ::        *:  *:***:.*. : :      : :*:. **.***

AsV-C1-45C          DLMFEAAQVIACFSTKPEAIMASAELTIGSTERVSDEVWYNYGGMPSGSPWTTTLNTICN
AsV-C1-HUN          DLMFEAAQVIACFSTKPEAIMASAELTIGSTERVSDEVWYNYGGMPSGSPWTTTLNTICN
AsV-C1              DLMFEAAQVIACFSTKPEAIMASAELTIGSTERVSDEVWYNYGGMPSGSPWTTTLNTICN
AsV-A1-HUN          DLMEHAIEVVSCFSEDPMSVIRAFQPTLISQERVSDELWEVRGSMPSGSPWTTMINTICN
AsV-A1-USA-IN1      ELMEHAIEVVACFSEDPMSVIRAFQPTLISQERVSDELWEVRGSMPSGSPWTTMINTICN
AsV-B1-44C          ELMIHCLDVLVSFHESPETCRKLAMLTIDSVERVSDEVWHVSGGMPSGSPLTTLMNTVCN
Orivirus-A1         GLMRAAVQCLANCSSNPELVVKIHEPTIVTTEIVRDEEWLVSGGMCSGSPSTTLLNCVCN
Orivirus-A2         GLMRAAVQCLANCSTDPELVVKIHEPTIVTTEIVRDEEWLVVGGMCSGSPSTTLLNCVCN
                     **  . : :     .*         *: : * * ** *   *.* **** ** :* :**
```

```
AsV-C1-45C          LLMCYTYLLDMGHCWSETFVVAYGDDVVISANIKHNLEGIENWFKTKFGATVTSSDKQSK
AsV-C1-HUN          LLMCYTYLLDMGHCWSDTYVVAYGDDVVISANIKHNLEGIENWFKTKFGATVTSSDKQSK
AsV-C1              LLMCYTYLLDMGHCWSETFVVAYGDDVVISANIKHNLEGIENWFKTKFGATVTSSDKQSK
AsV-A1-HUN          LLMCKTYLLDMGHDLTKTYVVCYGDDCVISVDQCHKLEGIEQWFMDKFGATVTPEDKSGK
AsV-A1-USA-IN1      LLMCKTYLLDMGHDITKTYVVCYGDDCVISVDQCHRLEGIEQWFMDKFGATVTPEDKSGK
AsV-B1-44C          MLMCYTWAFYQGYSCEEVFVAAYGDDVIISAKKKSNITDIVQCFKSWFGASITPAIKEGD
Orivirus-A1         IFVHTAFALVYN---LEFKVYCYGDDVIFSTKQQFDPRDYVEFMKEKFGMTVTSAQKTAE
Orivirus-A2         IFIHTAFALVYN---LDFKVYCYGDDVIFSTKQWFDPSVYVSFMAEKFGMTVTSAQKTSD
                    :::  :: :  .    .  * .**** ::*..         . :   ** ::*.  * ..

AsV-C1-45C          ITWTTKNNMEFLKRRPKELEFLPKIVGALDLDNMLQHLEWTKG--HIQDQLNSFYLELAL
AsV-C1-HUN          ITWTTKNNMEFLKRRPKELEFLPKIVGALDLDNMLQHLEWTKG--HIQDQLNSFYLELAL
AsV-C1              ITWTTKNNMEFLKRRPKELEFLPKIVGALDLDNMLQHLEWTKG--HIQDQLNSFYLELAL
AsV-A1-HUN          INWRFKNKLKFLKRTPMQLDWIPKIVGALDIDSMMDRIQWTKG--HFQEQLNCFYYELAL
AsV-A1-USA-IN1      IKWRFKNKLKFLKRTPMQLDWLPKIVGALDIDSMMDRIQWTKG--HFQEQLNCFYYELAL
AsV-B1-44C          ISWAPKHQVVFLKRRPKQLDFAPKIVGALDLQNMLDRIQWTTG--DFQSQLNSFYIELAL
Orivirus-A1         IAFVPPAEIEFLKRKP--TIFQGVTVGALSLDSLEHKIQWCRGLEAYKQQLKSFATELAL
Orivirus-A2         ITFVPPYEIEFLKRKP--VSFSGVTVGALSLESLENKIQWCRGLDAYKQQLRSFATELAF
                    * :    :: **** *    :    ****.::.: .:::*  *    :.**..*  ***:

AsV-C1-45C          HGREKYEEIRAKLAPRAPQLVHPTYACAKATITPMVAIL---------
AsV-C1-HUN          HGREKYEEIRAKLAPRAPQLVHPTYACARATITPMVAIL---------
AsV-C1              HGREKYEEIRAKLAPRAPQLVHPTYACAKATITPMVAIL---------
AsV-A1-HUN          HGEDTYNEARKSIAFRCPELVHPTYQCALQTIKPMVSLM---------
AsV-A1-USA-IN1      HGEDTYNEARRSIAFRCPELVHPTYHCALETIKPMVSLM---------
AsV-B1-44C          HGRETYNSVRAFLANKAPHCVHPTYDTAVLTVQPIVGFL---------
Orivirus-A1         HGRDQYTLLTSKLGIDIPWGAAKAWAKALLSSVIEGLDPGPPDRIVSP
Orivirus-A2         HGRSQYELTTDILGVDIPWGAAHAWARALLSSVTEGLDPGPPDRVVSP
                    **.. *      :.   *  .  ::  *  :
```
